# Hexokinase 2 confers radio-resistance in hepatocellular carcinoma by promoting autophagy-dependent degradation of AIMP2

**DOI:** 10.1038/s41419-023-06009-2

**Published:** 2023-08-01

**Authors:** Yilin Zheng, Yizhi Zhan, Yuqin Zhang, Yaowei Zhang, Yang Liu, Yuwen Xie, Yining Sun, Junying Qian, Yanqing Ding, Yi Ding, Yuan Fang

**Affiliations:** 1 Department of Radiation Oncology, Nanfang Hospital, Southern Medical University, Guangzhou, Guangdong Province China; 2 Department of General Surgery, Nanfang Hospital, Southern Medical University, Guangzhou, Guangdong Province China; 3 Department of Pathology, Nanfang Hospital, Southern Medical University, Guangzhou, Guangdong Province China; 4 Department of Pathology, School of Basic Medical Sciences, Southern Medical University, Guangzhou, Guangdong Province China; 5 Guangdong Province Key Laboratory of Molecular Tumor Pathology, Guangzhou, Guangdong Province China

**Keywords:** Radiotherapy, Liver cancer

## Abstract

With technological advancements, radiotherapy (RT) has become an effective non-surgical treatment for hepatocellular carcinoma (HCC), comprehensively improving the local control rate of patients with HCC. However, some patients with HCC still experience radio-resistance, cancer recurrence, and distant metastasis following RT. Our previous study has revealed that hexokinase 2 (HK2), a potent oncogene, was overexpressed in radio-resistant HCC cell lines; however, its role in HCC radio-resistance remains elusive. Here, we confirmed the upregulation of HK2 in HCC tissue, which is related to unfavorable prognosis in patients with HCC, and demonstrated that HK2 exerts a radio-resistant role by attenuating apoptosis and promoting proliferation in HCC cell lines. HK2 downregulation combined with ionizing radiation showed an excellent synergistic lethal effect. Mechanistically, HK2 alleviated ionizing radiation-mediated apoptosis by complexing with pro-apoptotic protein aminoacyl tRNA synthetase complex interacting multifunctional protein 2 (AIMP2) while enhancing its autophagic lysosomal-dependent degradation, thereby increasing radio-resistance of HCC. Pharmacologically, ketoconazole, an FDA-approved antifungal drug, served as an inhibitor of HK2 and synergistically enhanced the efficacy of RT. Our results indicated that HK2 played a vital role in radio-resistance and could be a potential therapeutic target for improving RT efficacy in HCC.

## Introduction

Hepatocellular carcinoma (HCC) is a major global health challenge and the fourth leading cause of cancer-related deaths worldwide [1]. Radiation therapy (RT) has become an indispensable part of HCC treatment since the development of stereotactic body radiotherapy (SBRT). However, the efficiency of RT is limited owing to inherent or acquired resistance, leading to local recurrence and metastasis [2]. Approximately 20% of patients reported local recurrence after receiving SBRT treatment, indicating resistance to ionizing radiation (IR) [3]. Thus, an urgent need to investigate molecular mechanisms underlying the radio-resistance of HCC has risen.

Resistance to IR is poly-modal and associated with biological alterations in both the innate tumor and surrounding microenvironment [4]. Increased resistance to apoptosis is one of the hallmarks of cancer and has been regarded as a determinant of radio-resistance [2]. Sensitization to apoptosis directly or indirectly was indicated as a prospective strategy to ameliorate the efficacy of RT [2]. Conversely, autophagy, a pro-survival pathway that enables the degradation and recycling of proteins and organelles to cope with stress, promotes progression in multiple types of cancers [5]. Several studies have indicated that the autophagy response of cancerous cells to RT is an indispensable pathway that, in contrast with cell death causing apoptosis, may culminate in cellular survival [6]. Simultaneously, inhibition of autophagy may elevate sensitivity to RT [7]. Thus, enhancing apoptosis in concert with constraining autophagy is a promising strategy for combating radio-resistance.

Hexokinase 2 (HK2), a key enzyme in glycolysis, could catalyze the following rate-limiting step in aerobic glycolysis: glucose phosphorylation and production of glucose-6-phosphate [8]. In pancreatic cancer, cervical cancer, and glioblastoma, HK2 is associated with radio-resistance as it promotes local recurrence and metastasis [9–11]. Among isoforms of hexokinases, HK4 is mainly expressed in hepatocytes, whereas HK2 is only expressed in HCC cells [12]. To better delineate the mechanism underlying radio-resistance, we constructed acquired ionizing radiation resistant (IR-R) HCC cell lines characterized by high levels of HK2 [13]. Additionally, we observed an upregulation of HK2 expression over time within the inherently radio-resistant cell line but not in the radio-sensitive cell lines. Hence, we speculated that HK2 might be a biomarker of radio-resistance in HCC. However, whether and how HK2 modulates the radiation response of HCC remains ambiguous. In this study, we aimed to investigate the function of HK2 and the mechanism underlying its modulation of HCC radio-resistance.

## Results

### High HK2 expression contributes to radio-resistance and predicts poor prognosis in HCC

**Fig. 1:**
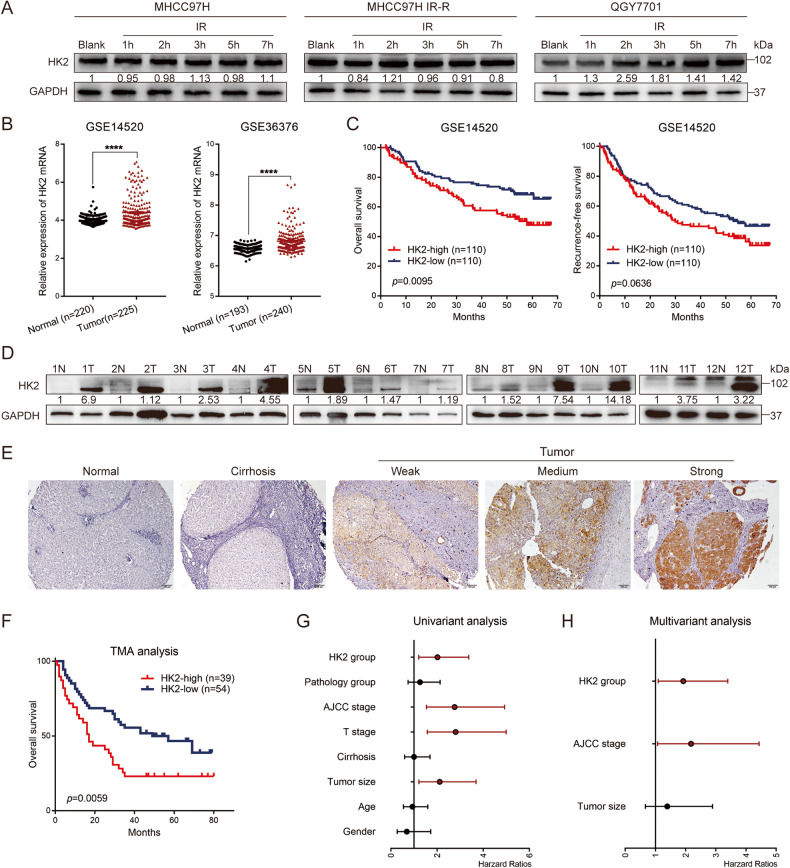
High expression of HK2 was associated with poor prognosis in Hepatocellular carcinoma. **A** Western blotting of HK2 in radio-sensitive and radio-resistant HCC cells at indicated time point upon 6 Gy radiation. **B** mRNA expression of HK2 obtained from GEO databases GSE14520 (normal group contains 220 samples and tumor group contains 225 samples), GSE36376 (normal group contains 193 samples and tumor group contains 240 samples). **C** The overall survival and recurrence-free survival comparison of HCC patients exhibiting high or low HK2 in GSE14520 using Kaplan–Meier analysis (each group contains 110 samples). **D** Western blotting of HK2 in 12 pairs of HCC tissues, GAPDH was loaded as a control. **E** Representative images of HK2 staining on tissue microarray (TMA). Scale bar 100 μm. **F** The overall survival rate of HCC patients according to high or low HK2 protein expression on TMA (HK2-high group contains 39 samples and HK2-low group contains 54 samples). **G**, **H** Univariant and multivariant analysis for the prognosis of HCC based on TMA analysis.

In the present study, we observed that HK2 protein levels were elevated over time in inherent IR-R (QGY7701) HCC cell lines, whereas they were maintained in radio-sensitive HCC cell lines MHCC97H and MHCC97L. Additionally, HK2 was not upregulated over time in acquired IR-R cell lines (Fig. 1A and Supplementary Fig. 1A), possibly because continuous irradiation during the construction of IR-R cell lines enabled the survival of a high HK2-expressing cell population. Therefore, we hypothesized that HK2 is a radio-resistance gene in HCC.

To assess HK2 expression in patients with HCC, we analyzed public Gene Expression Omnibus (GEO) databases (accession GSE14520 and GSE36376). Increased HK2 mRNA levels were observed in HCC tissues compared with that in adjacent normal tissues (Fig. 1B). Kaplan–Meier survival analysis validated that higher HK2 mRNA expression predicted poor overall survival (OS). Although no statistical difference, there was a trend toward shorter recurrence-free survival (RFS) in patients with high HK2 expression (Fig. 1C). Western blotting revealed that HCC samples from patients exhibited higher protein levels of HK2 (Fig. 1D), consistent with results of database analysis. Immunohistochemical staining (IHC) of HK2 in a liver cancer tissue microarray (TMA) containing 93 liver cancer samples and 87 matched normal liver tissues or cirrhosis tissues revealed that high HK2 expression was predictive of shorter OS (Fig. 1E, F). A Cox risk regression model of TMA was constructed using various clinicopathological indicators and HK2 expression. Univariate and multivariate analysis revealed that high HK2 expression was an independent risk factor for the poor prognosis of patients with HCC (Fig. 1G, H). These data verified that HK2 upregulated in HCC tissues at the mRNA and protein levels, was positively correlated with an unfavorable prognosis in patients with HCC.

### HK2 causes significant radio-resistance in HCC cells

**Fig. 2:**
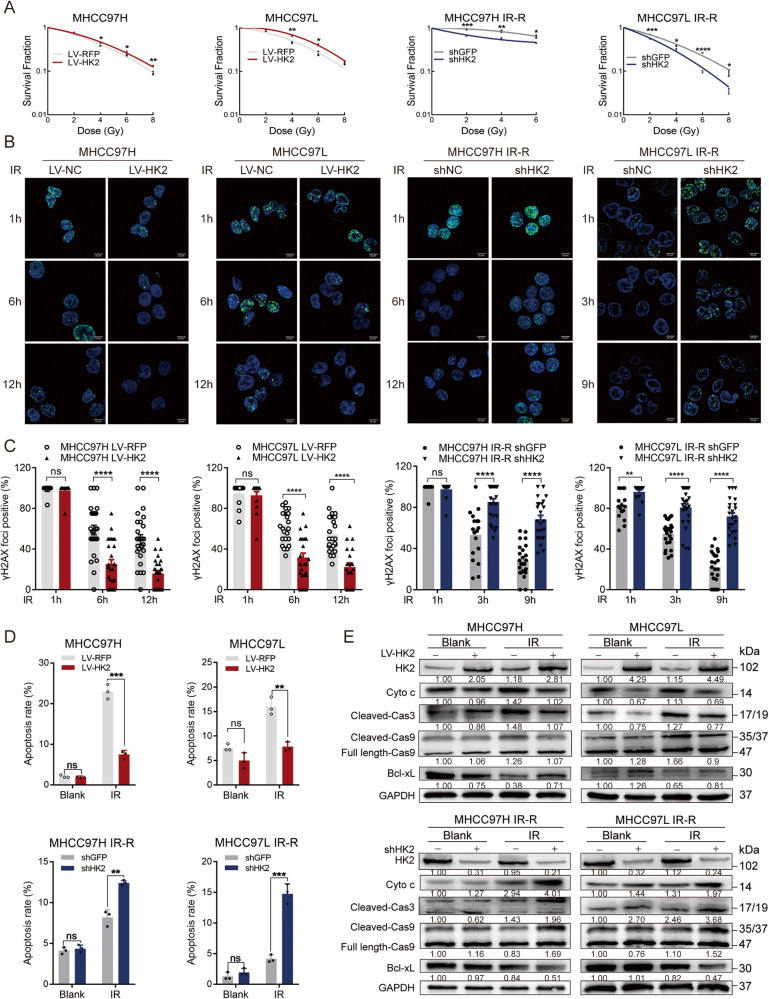
HK2’s role in proliferation and IR-induced apoptosis. **A** Colony formation assays of cells with different HK2 expressions after exposure to indicated doses of radiation. Error bars are the SEM of at least three independent replicates. **B**, **C** Cells were treated with 8 Gy radiation and then stained and quantified at the indicated times with antibodies to pH2AX-Ser139. Error bars are the SEM of at least ten independent replicates. Scale bar 20 μm. **D** Statistics of apoptosis in indicated cell lines with different HK2 status with or without 8 Gy radiation, respectively (*n* = 3). *p < 0.05, **p < 0.01, ***p < 0.001, ****p < 0.0001, based on Students’ *t*-test. **E** Western blotting of HK2, Cyto c, Cleaved Caspase 3, Caspase/Cleaved Caspase 9, and Bcl-xL in indicated groups.

To further investigate HK2-mediated radio-resistance, we constructed stable HK2-overexpression cells in radio-sensitive cell lines (MHCC97H and MHCC97L) and stable HK2-knockdown cells in radio-resistant cell lines (MHCC97H IR-R, MHCC97L IR-R, and QGY7701). Western blotting verified transfection efficiency (Supplementary Fig. 1B). CCK8 and colony formation in these edited cell lines were assessed. LV-HK2 or shHK2 did not consistently affect cell proliferation without IR (Supplementary Fig. 1C). However, after IR, LV-HK2 significantly promoted the growth of radio-sensitive cell lines, whereas shHK2 significantly reduced the growth of radio-resistant cell lines (Fig. 2A and Supplementary Fig. 1D).

To evaluate whether HK2 regulates cellular response to IR damage, an immunofluorescence (IF) assay of the phosphorylation of histone 2 A (pH2AX-Ser139), an indicator of effective DNA damage repair, was performed at the indicated time. The nuclear pH2AX-Ser139 focal point dissipated faster in LV-HK2 cells than in parallel control cells, whereas shHK2 in radio-resistant cells exerted the opposite effect (Fig. 2B, C and Supplementary Fig. 1E).

Because IR can lead to cell death, flow cytometry (FCM) of apoptosis was performed on cells with different levels of HK2. Although HK2 expression did not significantly affect apoptosis in the absence of IR, LV-HK2 could reduce IR-induced apoptosis (Fig. 2D and Supplementary Fig. 1F). Consistently, shHK2 combined with IR synergistically promoted apoptosis in radio-resistant cells compared with that in parallel cells (Fig. 2D and Supplementary Fig. 1F, G). Western blotting further confirmed that levels of apoptosis-related proteins, including Cyto c, Cleaved Caspase 3, and Cleaved Caspase 9, decreased with the overexpression of HK2, while the levels of the anti-apoptotic protein Bcl-xL increased with the overexpression of HK2, which was consistent with the results of FCM (Fig. 2E and Supplementary Fig. 1H, I). These results indicate that HK2 plays an indispensable role in regulating radio-resistance in vitro in HCC.

### HK2 serves as a radio-resistant gene in vivo

**Fig. 3:**
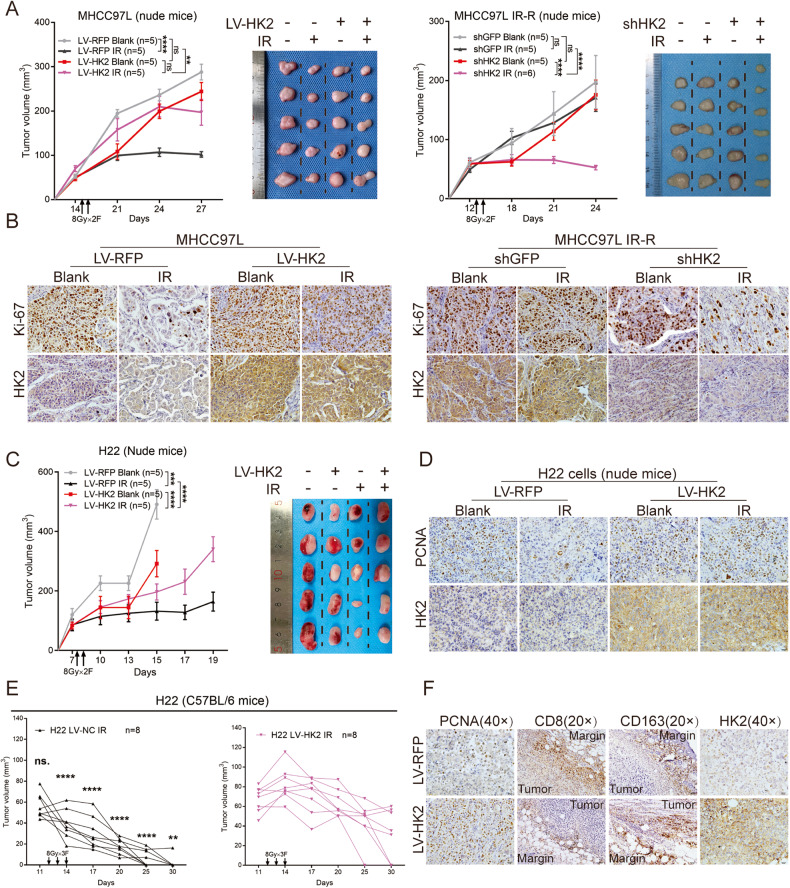
HK2’ s role of radio-resistance in vivo. **A** Subcutaneous xenograft nude mice model to twice 8 Gy radiation responsiveness among human HCC cells with different HK2 status in MHCC97L, MHCC97L IR-R. Tumor growth curves and images upon necropsy were presented. Error bars are the SEM of at least five independent replicates. **B** Representative IHC staining of Ki-67 and HK2 in the formalin-fixed tumor sections from blank and IR groups. Scale bar 20 μm. **C** Subcutaneous xenograft nude mice model to exhibit radiation responsiveness among mouse HCC cells with different HK2 status. Tumor growth curves and images upon necropsy were presented (*n* = 5). **D** Representative IHC staining of PCNA and HK2 in the formalin-fixed tumor sections from indicated treatment groups. Scale bar 20 μm. **E** Subcutaneous xenograft model in C57BL/6 mice to twice 8 Gy radiation responsiveness among H22 cells with different HK2 status. Tumor volumes were presented (*n* = 8). **F** Representative IHC staining of PCNA, CD8, CD163, and HK2 in the formalin-fixed tumor sections from indicated treatment groups. Scale bar 40 μm. Data were represented as mean ± SEM. *p < 0.05, **p < 0.01, ***p < 0.001, ****p < 0.0001.

Based on in vitro results, we investigated the role of HK2 in vivo by establishing subcutaneous xenograft models in nude mice. No significant difference in proliferation was observed between subcutaneous tumors with shHK2 and those with LV-HK2. However, treatment with IR showed that HK2 overexpression could effectively enhance radio-resistance, whereas knockdown of HK2 elevated radio-sensitivity and inhibited tumor growth (Fig. 3A and Supplementary Fig. 2A). We observed that IR significantly suppressed cell proliferation in parallel groups by IHC assays of Ki-67. However, in HK2-overexpression groups, the inhibitory effect of IR on proliferation was not obvious (Fig. 3B and Supplementary Fig. 2B–D). In radio-resistant group, IR exerted minor effects on the proportion of Ki-67, whereas in the shHK2 group, IR vitally suppressed the proportion of Ki-67 in subcutaneous tumors (Fig. 3B and Supplementary Fig. 3B). Transfection efficiency of HK2 was analyzed by IHC (Supplementary Fig. 2E).

We further incorporated murine-derived H22 HCC cells into subcutaneous xenograft models in nude and C57BL/6 mice. In nude mouse models, the growth rate of the HK2-overexpression group was higher than that of the parallel control group after IR (Fig. 3C). By examining the proportion of proliferating cell nuclear antigen (PCNA)-positive cells, we found that LV-HK2 increased radio-resistance and maintained proliferation after IR (Fig. 3D and Supplementary Fig. 2F). In C57BL/6 mice with subcutaneously inoculated H22 LV-RFP, tumors regressed faster and more frequently (7/8, 87.5%) than in those treated with H22 LV-HK2 (3/8, 37.5%) (Fig. 3E). Subsequent IHC of PCNA indicated that H22 LV-HK2 cells underwent more active proliferation after IR than H22 LV-RFP cells (Fig. 3F and Supplementary Fig. 2G). Numerous studies have reported that HK2 in cancer cells promote immune evasion [14]. Therefore, we focused on the infiltration of CD8+ T cells and M2 macrophages. Our data showed reduced infiltration of CD8+ T cells and an increase in M2 macrophages (CD163+) in mice inoculated with H22 LV-HK2 cells compared with that in mice inoculated with H22 LV-RFP cells (Fig. 3F and Supplementary Fig. 2G), implying that HK2 may act synergistically with IR to inhibit the immune system, thus contributing to HCC radio-resistance.

### HK2 complexes with AIMP2 protein and inhibits it by regulating lysosome-dependent autophagy

**Fig. 4:**
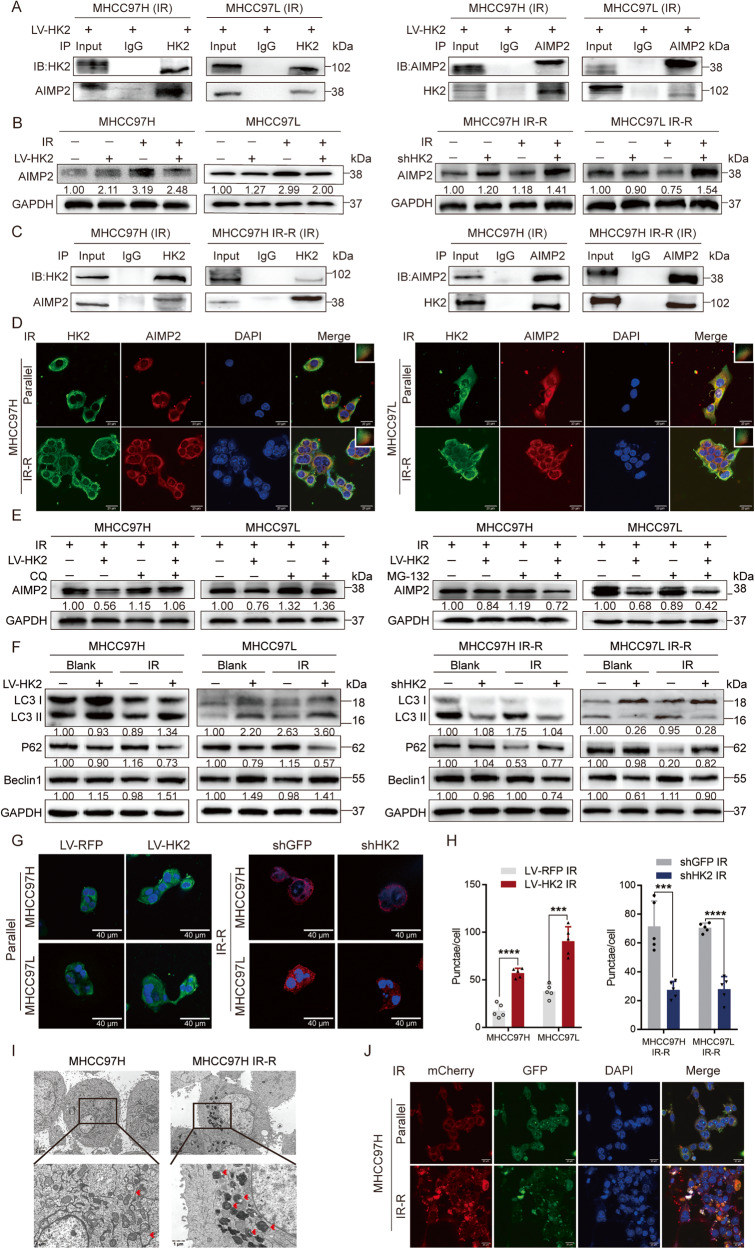
HK2 co-localizes with AIMP2 post IR. **A** Co-immunoprecipitation (Co-IP) identified the interaction between HK2 and AIMP2 in MHCC97H LV-HK2 and MHCC97L LV-HK2 post 8 Gy radiation. **B** Protein levels of AIMP2 in HCC cell lines with different HK2 expressions by facilitating western blotting post 8 Gy radiation. **C** Co-IP identified the interaction between HK2 and AIMP2 in MHCC97H and MHCC97H IR-R post 8 Gy radiation. **D** IF analysis of the co-localization of HK2 (green) and AIMP2 (red) in MHCC97H, MHCC97L, MHCC97H IR-R, and MHCC97L IR-R post 8 Gy radiation. Scale bar 20 μm. **E** MG-132 (20 μM, 24 h) and CQ (5 μM, 24 h) individually treatment in MHCC97H and MHCC97L cells with or without HK2 overexpression post 8 Gy radiation. **F** Western blotting of autophagy-related protein including Beclin1, LC3 I/II, P62 in indicated groups with different HK2 and AIMP2 status post 8 Gy radiation. **G**, **H** IF analysis and quantification of the LC3 II (green) in MHCC97H and MHCC97L cells with or without HK2 overexpression, and LC3 II (red) in MHCC97H IR-R, MHCC97L IR-R cells with or without HK2 knockdown post 8 Gy radiation after 48 h (*n* = 5). Scale bar 40 μm. **I** TEM of indicating cells post 8 Gy radiation after 48 h. **J** IF analysis of autophagosomes in indicated post-IR cells which transfected with a plasmid expressing mCherry-GFP-LC3 II. Scale bar 20 μm. Data were represented as mean ± SEM. *p < 0.05, **p < 0.01, ***p < 0.001, ****p < 0.0001.

To explore molecular mechanisms underlying HK2-mediated radio-resistance, immunoprecipitation (IP), and silver staining assays were carried out by comparing anti-HK2 products with anti-IgG IP products in lysates prepared from post-IR MHCC97H LV-RFP and LV-HK2 cell lines (Supplementary Fig. 3A). AIMP2 proteins were obtained using mass spectrometry of specific antigen bands (Supplementary Fig. 3B). As shown in Fig. 4A, these results were confirmed in post-IR MHCC97H LV-HK2 and MHCC97L LV-HK2 cells through co-immunoprecipitation (Co-IP) and western blotting that contrasted anti-HK2 or anti-AIMP2 IP product with the levels of anti-IgG IP product from each cell type (Fig. 4A).

Aminoacyl tRNA synthetase complex interacting with multifunctional protein 2 (AIMP2), a multifunctional tumor suppressor, can prompt apoptosis and repress cell stemness [15–17]. Western blotting results showed AIMP2 was significantly downregulated in HK2-overexpression cell lines but upregulated in HK2-knockdown cell lines after IR treatment (Fig. 4B and Supplementary Fig. 3C, D). IHC of AIMP2 in liver cancer tissues of aforementioned nude mice demonstrated similar results (Supplementary Fig. 3E, F). However, no difference in mRNA level were observed post-IR (Supplementary Fig. 3G). According to a previous study, HK2 does not act as a transcription factor [18]. Therefore, we inferred that HK2 mediated AIMP2 downregulation via post-transcriptional modification.

To confirm the relationship between HK2 and AIMP2 in unedited cells, Co-IP was performed. We observed that HK2 and AIMP2 were co-localized at the protein level only after IR (Fig. 4C and Supplementary Fig. 3H, I). Simultaneously, IF was performed on IR-R and parallel cells. HK2 signals overlapped with AIMP2 signals in the cytoplasm after IR while not in the absence of IR (Fig. 4D and Supplementary Fig. 3J, K). These results verified the co-localization of HK2 and AIMP2 at cellular and protein levels.

Because AIMP2 protein levels were inversely regulated by HK2, and HK2 may mediate AIMP2 downregulation via post-transcriptional modification, we pretreated radio-sensitive LV-RFP and LV-HK2 cells with autophagy inhibitor chloroquine (CQ) or proteasome inhibitor MG-132 followed by IR. CQ treatment successfully reversed AIMP2 downregulation, whereas MG-132 treatment did not (Fig. 4E and Supplementary Fig. 4A). Previous reports have shown that HK2 facilitates autophagic flux and could maintain cellular energy homeostasis [15]. However, whether the role of HK2 in regulating autophagy could be affected by IR to mediate AIMP2 downregulation remains unclear.

To further investigate the participation of autophagy in AIMP2 degradation, the expression of classic autophagy-related proteins, including LC3, P62, and Beclin1, were evaluated by western blotting. When combined with IR, LV-HK2 upregulated Beclin1 levels, enhanced LC3 II lipidation, and downregulated P62 levels compared with those in LV-RFP groups; while shHK2 had the opposite effects (Fig. 4F and Supplementary Fig. 4B). IF of LC3 II also exhibited a positive correlation with HK2 expression (Fig. 4G, H). Transmission electron microscope (TEM) images of autophagic vacuoles revealed that radio-resistant cell lines generated more autophagic vacuoles than radio-sensitive cell lines after IR, further demonstrating the relationship between HK2 and autophagy (Fig. 4I and Supplementary Fig. 4C). Simultaneously, mCherry-GFP-labeled LC3 II were transfected, higher number of autophagic vacuoles and upregulation of autophagic flux were observed in radio-resistant cell lines (Fig. 4J and Supplementary Fig. 4D–F). These results implied that HK2 upregulated autophagy in combination with IR and further reduced AIMP2 protein levels.

### Radio-resistant effect of HK2 is partly caused by AIMP2 degradation

**Fig. 5:**
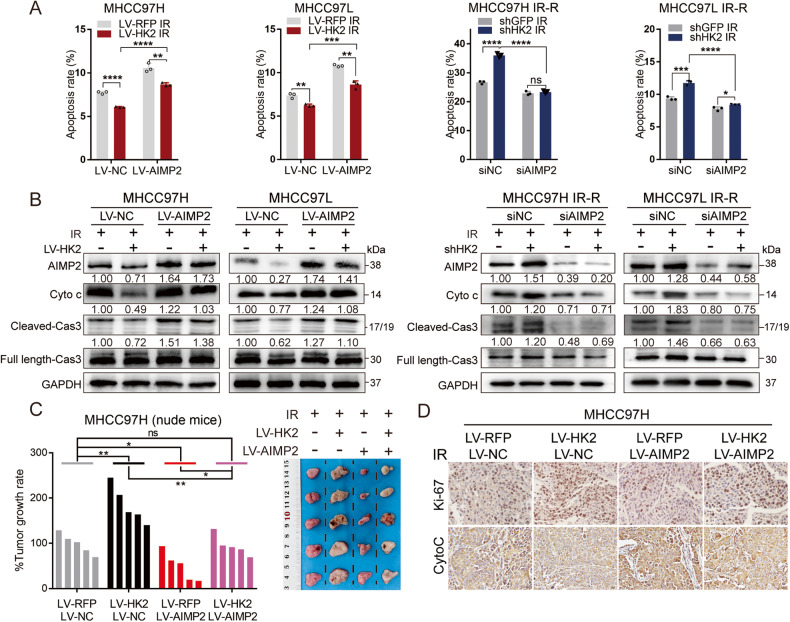
The function role of HK2 is dependent on AIMP2. **A** Apoptosis statistics in indicated cell lines with different HK2 and AIMP2 status after 8 Gy radiation, respectively (*n* = 3). **B** Western blotting of apoptosis-related protein including Cyto c, Caspase/Cleaved Caspase 3 in indicated groups post 8 Gy radiation. **C** Subcutaneous xenograft nude mice model to twice 8 Gy radiation responsiveness among human HCC cells with different HK2 and AIMP2 status in MHCC97H. Tumor growth rate and images upon necropsy were presented. **D** Representative IHC staining of Ki-67 and Cyto c in the formalin-fixed tumor sections from indicated groups, Scale bar 20 μm. Data were represented as mean ± SEM. *p < 0.05, **p < 0.01, ***p < 0.001, ****p < 0.0001.

We hypothesized that the role of HK2 in radio-resistance was partly mediated by impaired AIMP2 functions. Thus, we constructed HCC cell lines with different AIMP2 and HK2 statuses. Firstly, we screened the most efficient siRNA segments and transfected them into shGFP and shHK2 cell lines (Supplementary Fig. 5A). Western blotting and FCM revealed that siAIMP2 was capable of partially reversing the upregulation of apoptosis caused by shHK2 after IR (Fig. 5A, B and Supplementary Fig. 5B–E). Correspondingly, AIMP2-expressing recombinant lentivirus (LV-AIMP2) or control vectors (LV-NC) were introduced into LV-RFP & LV-HK2 cell lines. LV-AIMP2 partially enhances apoptosis that is reduced due to LV-HK2 after IR (Fig. 5A, B and Supplementary Fig. 5B, E).

Considering that HK2 is influenced by multiple signaling pathways, including P53, NF-κb, and Wnt/β-catenin pathways [19–21], and AIMP2 prompts apoptosis through the aforementioned pathways [16, 22, 23], we validated the expression of critical factors after IR. However, HK2 overexpression suppressed the expression of anti-apoptosis-related genes (*BCL2*, *BCL2L1*, *IER3*, and *XIAP*) of the NF-κb pathway at the mRNA level but not at the protein level after IR (Supplementary Fig. 5F, G). The P53 (*PUMA, PPM1D, PHLDA3, PMAIP1*, *and BAX*) and Wnt/β-catenin signaling (*AXIN2, C-myc, CD44*, and *NKD1*) pathways did not show consistent trends at mRNA or protein levels (Supplementary Fig. 5H–J) [16]. These results suggest that the downregulation of AIMP2 facilitated by HK2 may not be regulated by the aforementioned signaling pathways.

In vivo, subcutaneous xenograft models were established in nude mice. In MHCC97H LV-HK2 and LV-RFP cells after IR, AIMP2 overexpression decelerated tumor growth. In LV-HK2 group, high AIMP2 levels led to the retention of increased tumor volume caused by HK2 overexpression compared to the control group (Fig. 5C and Supplementary Fig. 6A). IHC of HK2 and AIMP2 was used to verify transfection efficiency (Supplementary Fig. 6B). IHC of Cyto c and Ki-67 elucidated that high AIMP2 expression enhanced apoptosis and partially suppressed the increased proliferation caused by HK2 overexpression in LV-HK2 cells (Fig. 5D and Supplementary Fig. 6C). These results revealed that the radio-resistant function of HK2 is partly due to AIMP2 degradation in vivo.

### Pharmacological inhibition of HK2 significantly alleviates its radio-resistant effect

**Fig. 6:**
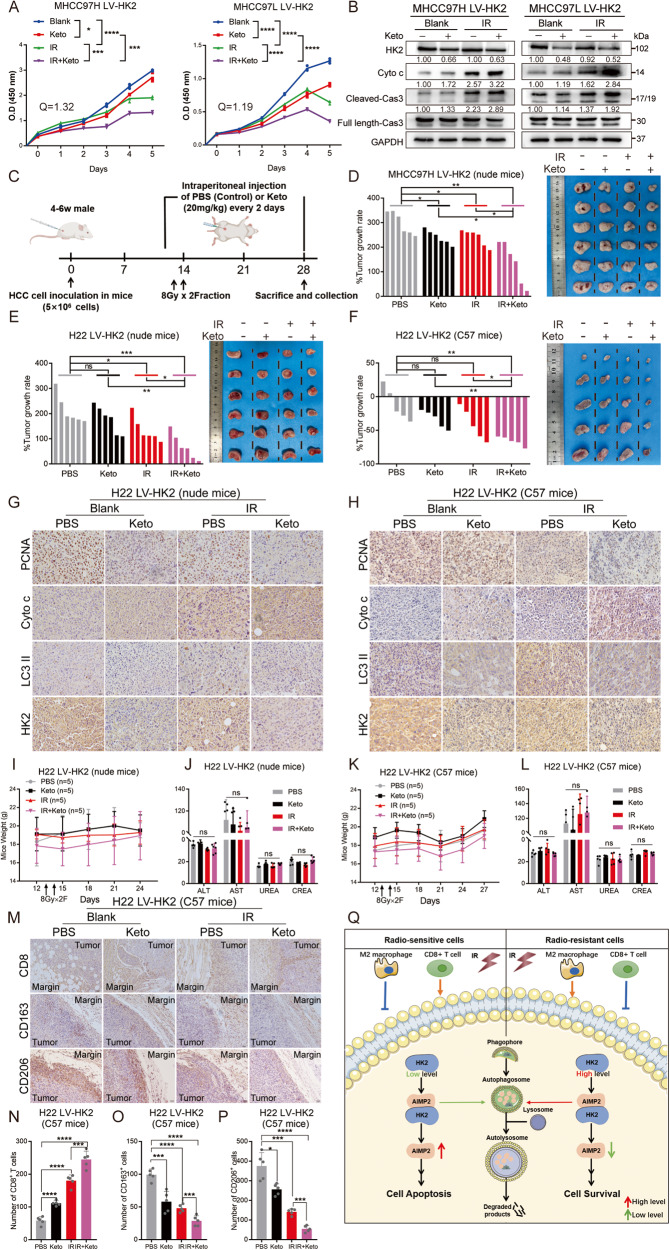
Targeting HK2 could alleviate radio-resistance. **A** CCK8 assays of Keto (20 μM, 48 h) with or without 8 Gy radiation and and the Q-value of the combination index of IR and Keto, respectively (*n* = 3). **B** Western blotting of apoptosis-related protein including Cyto c, Cleaved Caspase 3, and Caspase 3 in indicated groups with or without 8 Gy radiation. **C** Diagram of mice subcutaneous xenograft models with IR or Keto. **D**–**F** Tumor growth rate and tumor images of subcutaneous xenograft models in indicated mice with indicated cells and treatments. **G**, **H** Representative IHC staining of PCNA, Cyto c, LC3 II, and HK2 in the formalin-fixed tumor sections from nude mice and C57 mice with H22 LV-HK2 groups. Scale bar 20 μm. **I**–**L** Mice weight and indicators of mice liver and kidney in nude mice (*n* = 6) and C57 mice (*n* = 5) with H22 LV-HK2 groups. The units of ALT and AST are U/L. The units of UREA and CREA are Mmol/L and μmol/L, respectively. **M**–**P** Representative IHC staining and qualification of CD8, CD163, and CD206 in the formalin-fixed tumor sections from C57 mice with H22 LV-HK2 groups (*n* = 5). Scale bar 40 μm. **Q** Schematic diagram summarizing our working model, in brief, HK2 could interact with and prompt the lysosomal-dependent autophagy of AIMP2, thereby attenuating radiation-induced apoptosis, while high HK2 expression promotes immunosuppression, both of which accelerate the progression of HCC. Data are represented as mean ± SEM. *p < 0.05, **p < 0.01, ***p < 0.001, ****p < 0.0001.

Considering the predominant expression of HK2 in HCC, we speculated that HK2 inhibition might be highly selective for HCC. Ketoconazole (Keto), a commonly used FDA-approved antifungal drug, inhibits glioblastoma cell proliferation by targeting HK2 [24]. To verify whether Keto could participate in IR sensitization, we divided LV-HK2 cells into the following treatment groups for pretreatment: (i) control; (ii) Keto treatment; (iii) IR treatment (8 Gy); and (iv) IR + Keto treatment groups. CCK8 assays revealed that Keto treatment partially inhibited cell survival, whereas combination treatment of Keto and IR further promoted a synergistic lethal effect by calculating the combination index (Q) (Fig. 6A). Western blotting showed that Keto partially enhanced apoptosis levels in LV-HK2 cells and integrated with IR to further increase apoptosis (Fig. 6B).

Subsequently, subcutaneous xenograft models of nude mouse with MHCC97H LV-HK2 cells or H22 LV-HK2 were established. Keto was injected intraperitoneally at the beginning of the day before IR treatment and maintained every two days at a concentration of 20 mg/Kg (Fig. 6C). Although not significantly, both Keto and IR treatments partially reduced tumor volume and inhibited tumor cell multiplication, whereas combined therapies synergistically inhibited tumor volume and growth rate (Fig. 6D, E and Supplementary Fig. 6D, E). IHC assays revealed that Keto integrated with IR significantly restrained proliferation and autophagy and elevated apoptosis more than Keto or IR alone (Fig. 6G and Supplementary Fig. 6F–H). Mice weight and indicators of liver and kidney were not influenced by Keto treatment (Fig. 6I, J and Supplementary Fig. 6I, J). Similarly, the tumor growth rate of combination treatment in H22 LV-HK2 subcutaneous xenograft C57BL/6 mice was more restricted than other treatments (Fig. 6F and Supplementary Fig. 6K). IHC assays revealed the same results (Fig. 6H and Supplementary Fig. 6L). Similarly, mice weight and vitality were not influenced (Fig. 6K, L). Moreover, in the combination group, more infiltrated CD8+ T and fewer M2 macrophages were observed in the tumor, indicating that combination treatment demonstrated better reversal of immunodeficiency (Fig. 6M–P). These in vitro and in vivo results imply that pharmacological targeting of HK2 might be an effective adjunct to IR in HCC and has great clinical translation potential.

## Discussion

RT combined with systemic therapy has become the first-line treatment for patients with advanced HCC [25]. However, approximately 10–20% of the patients have reported local recurrence and distant metastasis, indicating that radio-resistance has become a substantial hurdle [26]. Radio-resistance involves changes in multiple biological traits, including apoptosis, autophagy, DNA repair, and cellular energy [4]. Exploring target genes for radio-resistance that are indicative of poor prognosis after IR is an urgent clinical priority.

HK2 is commonly upregulated in various cancers and exerts pro-cancer properties [27]. We observed that HK2 levels were elevated in inherently radio-resisitant HCC cell lines post-IR while not in radio-sensitive cell lines. In a hepatocarcinogenesis mice model, liver-specific depletion of HK2 decreased tumor incidence [28]. In colorectal cancer, gallbladder cancer, and acute myeloid leukemia, HK2 may accelerate cancer proliferation and DNA damage repair [19, 29, 30]. Growing evidence indicates that targeted glycolysis exerts a tumor-killing effect and synergizes the efficacy of chemotherapy and radiotherapy [31, 32]. HK is the initial rate-limiting enzyme of glycolysis, based on its function of catalyzing the phosphorylation of glucose into glucose-6-phosphate [33]. The occurrence of chemo- and radio-resistance resulting from high expression of HK2 was associated with glycolysis, generating more energy and promoting proliferation [31]. lornitamine, 3-Bromopyruvate, and 2-deoxyglucose inhibit glycolysis by targeting HK2 in glioblastoma and hepatocellular carcinoma and promoting chemo-sensitivity [34–36]. In liver tissues, HK2 is mainly expressed in HCC cells, whereas HK4 is mainly expressed in normal hepatocytes, making targeted inhibition of HK2 highly selective against HCC cells [12]. Moreover, during hepatocarcinogenesis, the expression of HK can be switched from the isoenzyme HK4 to HK2 [37]. HK2 is influenced by multiple signals, including those in the PI3k/AKT, NF-κB, C-myc and P53 pathways, owing to its role in glycolysis [19–21, 27]. In addition, similar to other glycolysis-related genes, such as *PKM2* and *LDHA*, HK2 could perform functions other than regulation of glycolysis [38, 39]. HK2 prevents apoptosis by binding to VDAC in the outer membrane of mitochondria and inhibits the release of pro-apoptotic factors, such as Cyto c [12, 33]. HK2 also exerts a vital role in immune evasion by suppressing the recruitment of CD8+ T cells and affecting the polarization of M2 macrophages [14, 40, 41]. Clinically, HK2 serves as a non-invasive preclinical diagnosis of urine and pleural effusions, and can be used to identify circulating tumor cells in multiple cancers [42, 43]. HK2 is associated with radio-resistance in cervical cancer and glioblastoma, but in HCC radio-resistance remains unclear [11, 44].

Our investigation revealed that HK2 levels were elevated in HCC tissues and predicted unfavorable prognosis in patients with HCC. HK2 overexpression in radio-sensitive HCC cells significantly elevated proliferation and reduced apoptosis, whereas HK2 knockdown in IR-R HCC cells induced sensitivity to IR both in vitro and in vivo. These results strongly supported the function of HK2 in radio-resistance in HCC.

IR damages double-stranded DNA, leading to cell apoptosis [45]. The promotion of apoptosis has emerged as a promising strategy for radio-resistance in HCC [4]. AIMP2, a multifunctional tumor suppressor, was proposed as a potential downstream protein of HK2 by IP and mass spectrometry [16]. AIMP2 could elevate cell apoptosis by activating P53 and inhibiting the TRAF2-dependent NF-κb pathway [22, 46]. In colorectal cancer, AIMP2 participates in the regulation of adenoma initiation via Wnt/β-catenin signaling [16]. We observed that HK2 interacted with and modulated the degradation of the AIMP2 protein only post-IR, thus suppressing apoptosis levels and facilitating radio-resistance both in vivo and in vitro after IR. Given that HK2 and AIMP2 are regulated and influenced by multiple pathways, including NF-kb, Wnt/β-catenin, and P53 pathways, we evaluated related factors, but no further results were obtained from the study.

Considering that HK2 downregulates the protein but not the mRNA level of AIMP2, we hypothesized that HK2 may mediate the degradation of the AIMP2 protein. Rescue tests using the autophagy inhibitor CQ and the proteasome inhibitor MG-132 clarified that HK2 upregulated autophagy and, thus, the degradation of AIMP2 after IR. Lysosomal autophagy, a highly conserved process of self-digestion, mediates cellular homeostasis by degrading and recycling damaged organelles and proteins in response to stress [5]. IR can disrupt the mitochondrial function of tumor cells and induce DNA damage while inducing protective autophagy in tumor cells to repair DNA damage and limit further apoptosis [47]. Promotion of autophagy reduces radio-sensitivity in pancreatic cancer and glioblastoma [47, 48]. Moreover, HK2 drives glycolysis and autophagy, thus conferring cellular protection in endometrial cancer [49]. Autophagy was elevated after IR treatment in HK2 overexpression cells. However, how HK2 mediates AIMP2 degradation through autophagy after IR remains uncertain.

HK2 was a significant target for anti-tumor therapy. 2-deoxyglucose (2-DG), an indirect pharmacological inhibitor of HK2, has entered phase II clinical trials [50]. Phase I/II clinical trials have already investigated the synergistic effect of radiotherapy and 2-DG on glioblastoma [51]. Our in vitro and in vivo experiments with Keto, an FDA-approved antifungal drug used as an HK2 inhibitor in glioblastoma, demonstrated that HK2 blockage, in combination with IR, effectively halted cancer cell proliferation and autophagy. Meanwhile, these combinations also could reverse immune deficiency, recruit a variety of CD8+ T cells, and restrict M2 macrophage polarization. However, the underlying mechanism warrants further exploration. In conclusion, HK2 inhibition might be an effective adjuvant for IR treatment and enhancement of clinical translation potential.

This study sheds light on the role of HK2 in HCC radio-resistance (Fig. 6Q). Targeting HK2 synergistically enhanced the efficacy of IR and HK2 could further be a potential therapeutic target in HCC radiotherapy.

## Materials and methods

### Patient samples

Twelve pairs of HCC and non-tumor-adjacent samples from patients undergoing curative surgery obtained from Hepatobiliary Surgery of Nanfang Hospital were used for western blot analysis. A liver cancer tissue microarray (TMA) containing 93 liver cancer samples and 87 matched normal liver tissues or cirrhosis tissues purchased from Shanghai Xinchao were used for immunohistochemistry (IHC) and prognosis analysis. IHC data of TMA were sorted from highest to lowest. The top 60% of patients were designated as the HK2-high group (*n* = 54) and the latter 40% of patients were designated as the HK2-low group (*n* = 39). The datasets involved were downloaded from the public GEO (Gene Expression Omnibus) databases. The GSE14520 dataset contains paired tissue data from 220 patients, and tumor tissue data were sorted according to the mRNA expression. The first 50% of patients were designated as the HK2-high group (*n* = 110), and the second 50% were designated as the HK2-low group (*n* = 110).

### Cell culture and cell construction

The HCC cell lines were purchased from Shanghai Cell Bank. Acquired radio-resistant cell lines MHCC97H IR-R and MHCC97L IR-R were constructed as follows [13]. These cells were cultured in DMEM medium supplemented with 10% fetal bovine serum (Gbico, America) and maintained at 37 °C and 5% CO_2_. Lentiviral vectors and short hairpin RNA were designed and constructed by WZ Biotech (Jinan, China). Flag-tagged HK2 overexpression vectors (LV-HK2) and control vectors (LV-RFP) were transferred into MHCC97H, MHCC97L, and H22. Consistently, HK2 shRNA (shHK2) and control short hairpin RNA (shGFP) were transfected into MHCC97H IR-R, MHCC97L IR-R, and QGY7701. AIMP2 overexpression vectors (LV-AIMP2) and control vectors (LV-NC) were transferred into MHCC97H (LV-RFP&LV-HK2) and MHCC97L (LV-RFP&LV-HK2) cells. The small interfering RNA (siRNA) oligonucleotides constructed by Ribobio (Guangzhou, China) contain siAIMP2 (three targets) and siControl (siNC). The plasmid of mCherry-GFP-LC3 II was constructed by Hanyi Biotech (Guangzhou, China). Both were transfected into cells using Lipofectamine 3000 (Thermo Fischer Scientific) transfection reagent following the supplier’s recommendations. Ketoconazole, chloroquine (CQ) and MG-132 were all purchased from Selleck and dissolved in DMSO.

### Subcutaneous xenograft model

Stably transfected cells (5 × 10^6^) were implanted subcutaneously into the backside of nude mice or C57BL/6 mice (4–6 weeks old, male); IR facilitation was performed on days 12 and 13 after implantation. When the volume reached 200 mm^3^, mice were randomly divided into four groups for further treatment. Ketoconazole (purchased from Selleck) was dissolved in 30% propylene glycol, 5% Tween 80, and 65% D5W and injected intraperitoneally at the beginning of the day before IR and maintained every two days at a concentration of 20 mg/Kg [13]. Tumor volume (mm^3^) was measured and calculated as follows: tumor volume = L × W × H/2, where L is length, W is width, and H is height. When the volume reached 1000 mm^3^, tumors were harvested and dipped into formalin immediately for further analysis. We were blinded to the group during the experiment.

### IR treatment and CCK8 and colony formation assays

The mice and cells were exposed under different irradiation doses from 0 to 8 Gy using a linear accelerator (Varian Clinac 23EX Linear Accelerator, USA). For the CCK8 assay, cells were plated on 96-well plates 24 h before the IR and measured with cell counting kit-8 (DOJINDO Laboratories) at 450 nm for 5–6 days. Synergistic killing effects of IR and Keto were calculated using the formula: Q = E_(A+B)_/(E_A_ + E_B_ − E_A_ × E_B_), where Q = 0.85–1.15 means additive, Q > 1.15 means synergism and Q < 0.85 means antagonism. E_(A+B)_ is the cell killing rate of combination therapy, E_A_ and E_B_ indicate cell killing rates of A and B, respectively [34, 35]. For colony formation assay, cells were planted in six-well plates in the meantime setting up replicate sets, irradiated at specific doses from 0 to 8 Gy on the second day, and returned to the incubator for around 2–3 weeks. Discarded medium and washed cells with phosphate buffer solution (PBS) twice, fixed with methanol, and stained with 0.5% crystal violet. Colonies in each well were counted by Image J software. Surviving fraction at different doses was calculated by using the formula described previously: [(number of surviving colonies in dose X)/(number of cells seeded for dose X (average colonies arising from the nonirradiated cells (0 Gy)/number of nonirradiated cells seeded)] [13]. The dose survival curve was then plotted using GraphPad Prism 7.0 software, and survival curves were plotted according to the single-hit multi-target model SF = 1-(1-e^-D/D0^) ^N^, where SF is the surviving fraction and D is the IR dose [52].

### Western blotting analysis

Total proteins were extracted by RIPA buffer (Beyotime, China), lysed from cells or tissues with the addition of protease inhibitors (1:100, CWBIO) and phosphatase inhibitors (1:100, CWBIO), separated by 10% SDS PAGE or 12% SDS PAGE, and then transferred onto PVDF membranes. After incubation with primary antibodies overnight at 4 °C, membranes were washed three times with PBST. These blots were incubated with HRP-conjugated secondary antibody (Proteintech #SA00001-2, America) for 1.5 h at 4 °C. The blots were washed three times with PBST and visualized by enhanced chemiluminescence (Tanon ECL, China). Primary antibodies used for western blot were listed as follows: GAPDH (Proteintech #10494-1-AP, China), HK2 (Proteintech #22029-1-AP, China), AIMP2 (Proteintech #10424-1-AP, China), Cyto c (Proteintech #10993-1-AP, China), Caspase 3/p17/p19 (Proteintech #19677-1-AP, China), Bcl-xL (Abcam #ab32370, America), LC3 (Proteintech #14600-1-AP, China), P62 (Proteintech #18420-1-AP, China), Becline1 (Proteintech #11306-1-AP, China).

### Immunohistochemistry (IHC)

IHC staining of tumors was performed as described previously [13]. Primary antibodies were HK2 (Proteintech #22029-1-AP, China), HK2 (Proteintech #66974-1-Ig, China), AIMP2 (Proteintech #10424-1-AP, China), Ki-67 (Cell Signaling Technology #9449, America), Cyto c (Proteintech #10993-1-AP, America), PCNA (Abclonal #a0264, China), LC3 II (Proteintech #18725-1-AP, China), CD8 (Abcam #ab217344), CD163 (Proteintech #16646-1-AP, China), CD206 (Cell Signaling Technology #24595, American). Staining intensity was independently evaluated by two senior pathologists. The immunohistochemical score was calculated based on the positive reaction area and the intensity of staining. Each group quantified at least five different views.

### Quantitative real-time PCR (qRT-PCR)

Total RNA from cells was derived using TRIzol reagent (AG, China) and reverse transcribed into cDNA using Evo M-MLV RT Master Mix (AG, China) according to the manufacturer’s instructions. Subsequently, mRNA expression was analyzed by using SYBR® Green Premix Pro Taq HS qPCR Kit (Rox Plus) (AG, China) under a LightCycler 96 detection system (Roche). GAPDH was selected for normalization. Primer sequences were listed in Supplementary Table 1.

### Flow cytometry of apoptosis

Cells were pretreated with the corresponding experimental design, then harvested and stained according to the manufacturer’s instructions (KeyGEN), followed by flow cytometry for apoptosis analysis [13], the apoptosis rate included early apoptosis and late apoptosis.

### Co-immunoprecipitation assay

Total protein was extracted using cell lysis buffer with the addition of protease inhibitors (1:100, CWBIO) and phosphatase inhibitors (1:100, CWBIO). Then the lysate (2000 μg protein) was incubated overnight at 4 °C with anti-HK2 (1:50, Proteintech), anti-AIMP2 (1:50, Proteintech), or IgG (as a negative control, 1:100, Abclonol). The protein-antibody complex was incubated with protein A/G magnetic beads for 6 h at 4 °C. Immunoprecipitation was then collected by centrifugation at 2000×*g* for 5 min at 4 °C, and the beads complex was washed five times with PBS. Then, protein A/G magnetic beads were eluted by boiling in 5X SDS sample buffer before western blot.

### Silver staining

Silver staining was performed following the protocol provided by Beyotime Technology. Briefly, the gels were fixed in 50% ethanol/10% acetic acid for 40 min after electrophoresis and washed in 30% ethanol for 10 min and Milli-Q water for another 10 min. The gels were incubated with silver staining sensitizer for 2 min and then with silver nitrate for 10 min. Afterward, the gels were put in Milli-Q water for 1 min, removed, and in the developing solution. When clear staining was achieved, gels were transferred to the stop solution for 10 min. Stained gels were stored in Milli-Q water at 4 °C [19].

### Immunofluorescence (IF)

Cells were grown in confocal dishes for 48–72 h and proliferated for two to three generations, then fixed using 4% paraformaldehyde for 30 min, washed three times with PBS, and permeabilized using 0.3% Triton X-100 for 8 min. Then blocked with goat serum, followed by the appropriate antibody. Anti-HK2 antibodies (Proteintech #66974-1-Ig, America), anti-AIMP2 (Proteintech #10424-1-AP, America), anti-LC3B (Proteintech #18725-1-AP, America) antibodies were used herein, followed by Alexa-488-conjugated goat anti-Mouses secondary antibody (1:200, Bioss) or Alexa-555-conjugated goat anti-Rabbits secondary antibody (1:200, Bioss) for imaging. The cells were then stained with DAPI as a nuclear indicator and imaged with a confocal laser scanning microscope (Olympus FV1000). Autophagy was measured by an observer blinded to experimental conditions.

### Transmission electron microscope characterization of cells

Cells were digested and collected after 48 h of exposure to 8 Gy irradiation, then fixed by special solution for TEM characterization. Samples were prepared on nickel or aluminum TEM grids.

### Statistics

The results were shown as mean ± SEM, and all data followed a normal distribution with homogenous variance. Survivals related to HK2 expression in patients with HCC were calculated by the Kaplan–Meier survival analysis. A two-tailed, unpaired, or paired Student’s *t*-test was utilized to compare variables of two groups, and one-way or two-way ANOVA was used for multi-group comparisons. Co-localization of HK2, AIMP2, and the LC3 II punctae in IF staining were calculated with Image-Pro Plus 6.0. Significant differences were showed with *p < 0.05, **p < 0.01, ***p < 0.001, and ****p < 0.0001. Statistical details are included in the respective figure legends.

## Supplementary information

Extended Figure1 (Figure1 merge file)

Extended Figure2 (Figure2 merge file)

Extended Figure3 (Figure3 merge file)

Extended Figure4 (Figure4 merge file)

Extended Figure5 (Figure5 merge file)

Extended Figure6 (Figure6 merge file)

Supplementary Figure

Supplementary Table 1

Supplementary Figure1

Supplementary Figure2

Supplementary Figure3

Supplementary Figure4

Supplementary Figure5

Supplementary Figure6

Extended Supplementary Figure1 (Supplementary Figure1 merge file)

Extended Supplementary Figure2 (Supplementary Figure2 merge file)

Extended Supplementary Figure3 (Supplementary Figure3 merge file)

Extended Supplementary Figure4 (Supplementary Figure4 merge file)

Extended Supplementary Figure5 (Supplementary Figure5 merge file)

Extended Supplementary Figure6 (Supplementary Figure6 merge file)

Original Western Blots

aj-checklist

## Data Availability

The datasets used and/or analyzed during the current study are available from the corresponding author on reasonable request.
