# Supplementary material for: Hexokinase 2 confers radio-resistance in hepatocellular carcinoma by promoting autophagy-dependent degradation of AIMP2
Source: Cell Death Dis. 2023 Aug 1;14(8):488. doi: 10.1038/s41419-023-06009-2 (PMC10390495; doi:10.1038/s41419-023-06009-2)
Supplement: Supplementary file 1 — Extended Figure1 (Figure1 merge file) [file 41419_2023_6009_MOESM1_ESM.pdf]

A

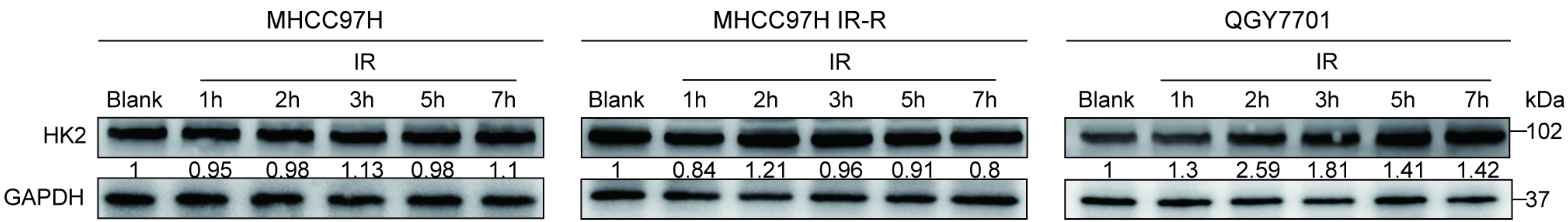
MHCC97H
MHCC97H IR-R
QGY7701
IR
Blank
1h
2h
3h
5h
7h
kDa
HK2
GAPDH
1
0.95
0.98
1.13
0.98
1.1
1
0.84
1.21
0.96
0.91
0.8
1
1.3
2.59
1.81
1.41
1.42
102
37

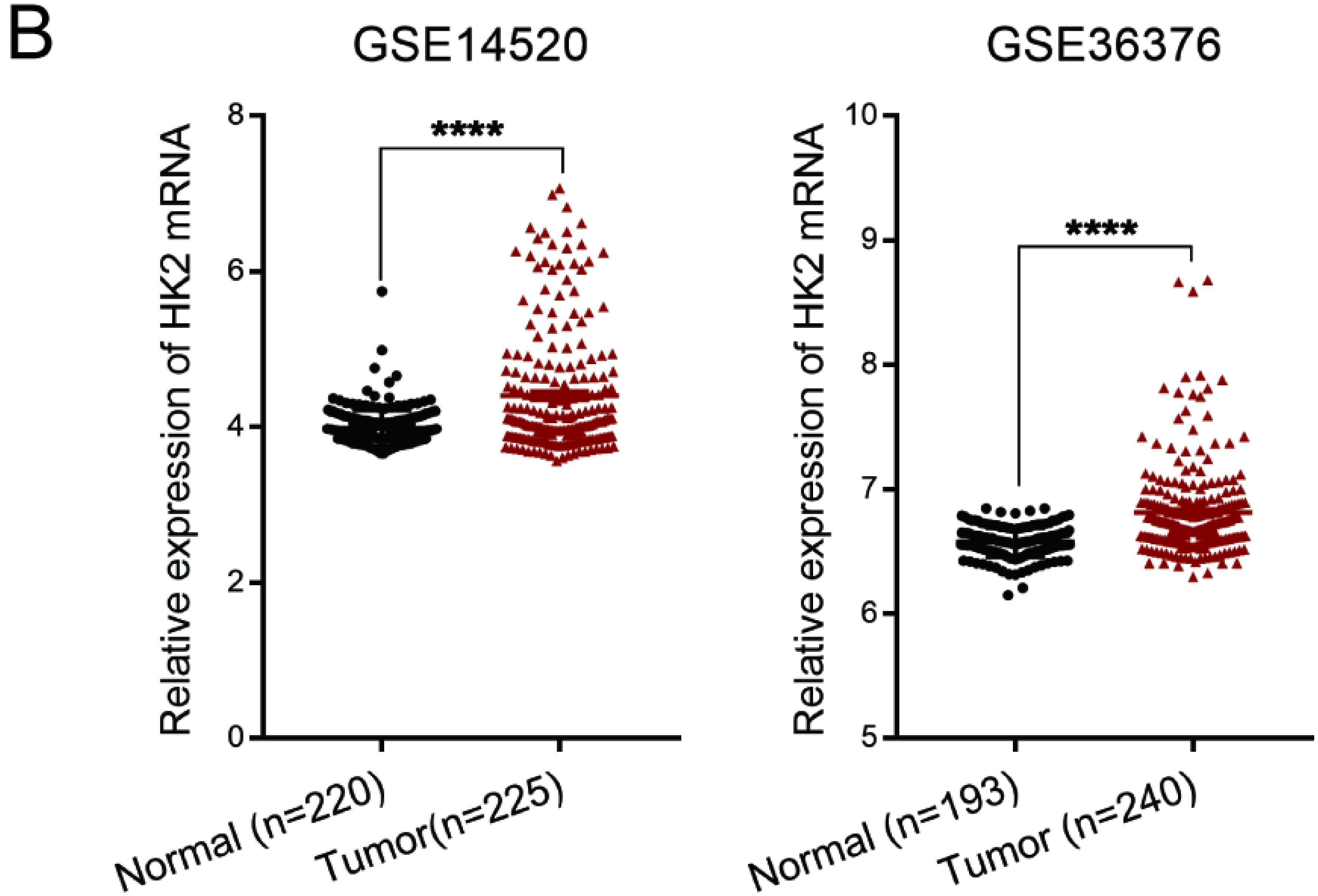
B
GSE14520
GSE36376
Relative expression of HK2 mRNA
****
****
0
2
4
6
8
5
6
7
8
9
10
Normal (n=220)
Tumor(n=225)
Normal (n=193)
Tumor (n=240)

C

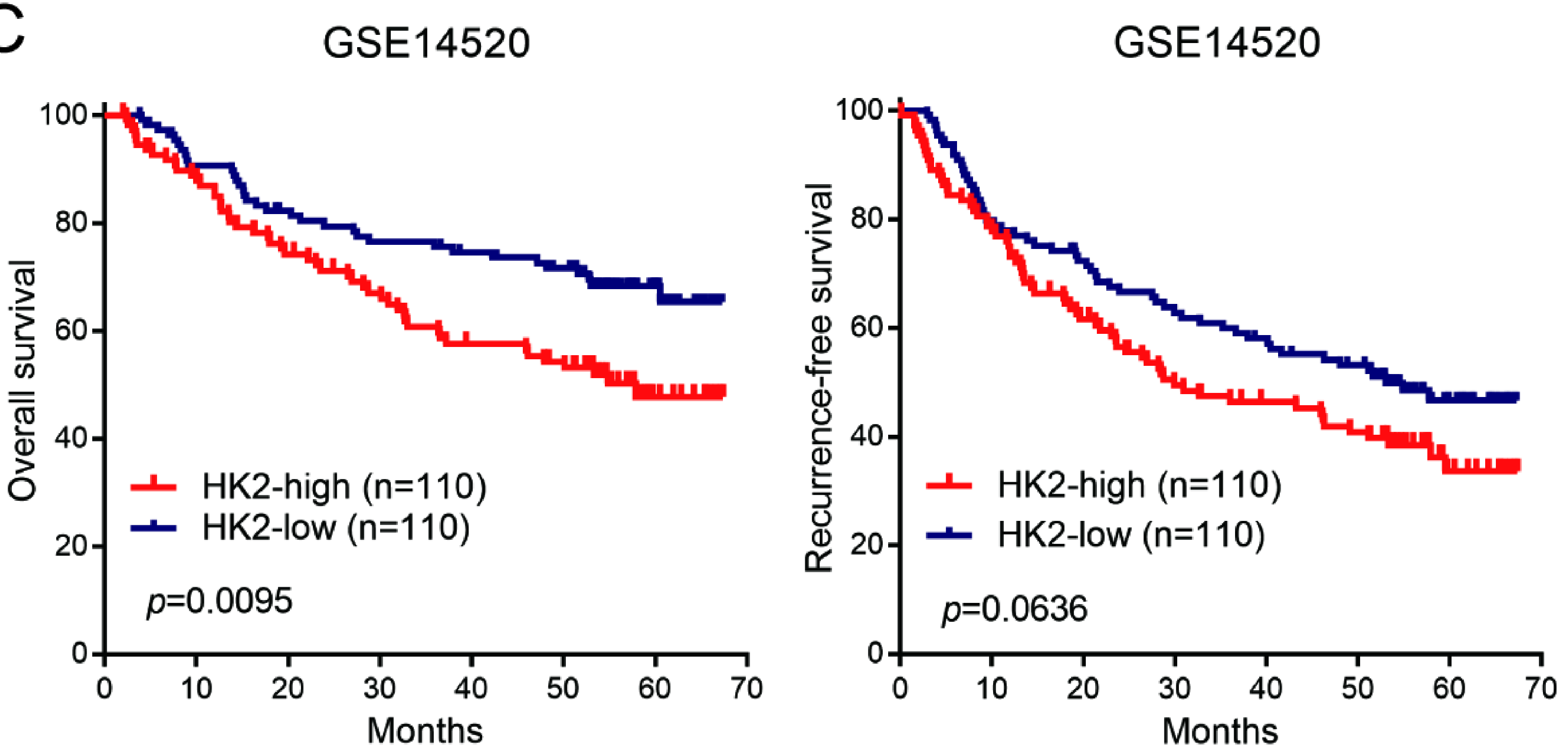
GSE14520
Overall survival
100
80
60
40
20
0
0
10
20
30
40
50
60
70
Months
HK2-high (n=110)
HK2-low (n=110)
p=0.0095
GSE14520
Recurrence-free survival
100
80
60
40
20
0
0
10
20
30
40
50
60
70
Months
HK2-high (n=110)
HK2-low (n=110)
p=0.0636

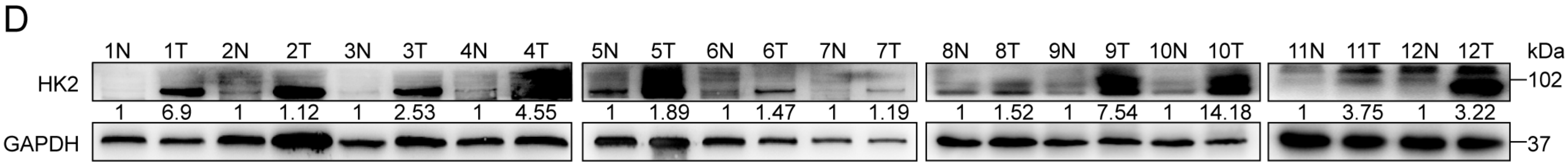
D
1N 1T 2N 2T 3N 3T 4N 4T 5N 5T 6N 6T 7N 7T 8N 8T 9N 9T 10N 10T 11N 11T 12N 12T
kDa
HK2
102
1 6.9 1 1.12 1 2.53 1 4.55 1 1.89 1 1.47 1 1.19 1 1.52 1 7.54 1 14.18 1 3.75 1 3.22
GAPDH
37

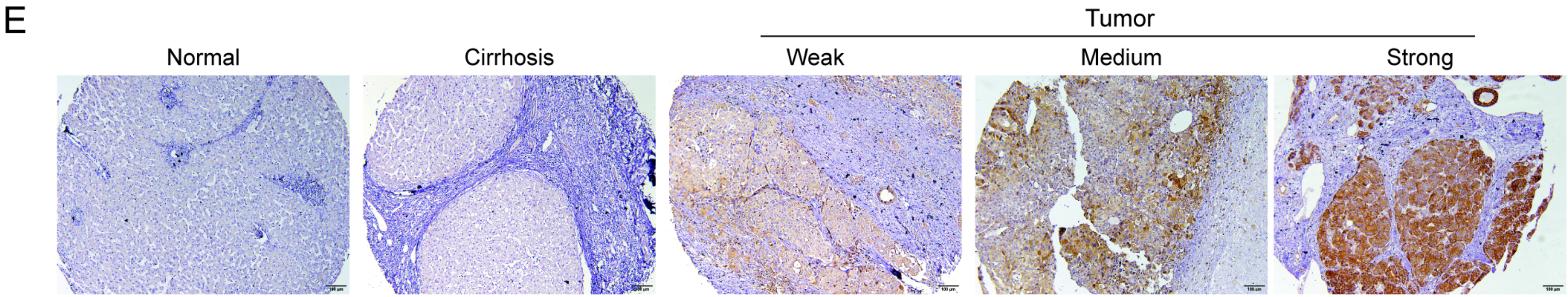
E
Tumor
Normal
Cirrhosis
Weak
Medium
Strong

F

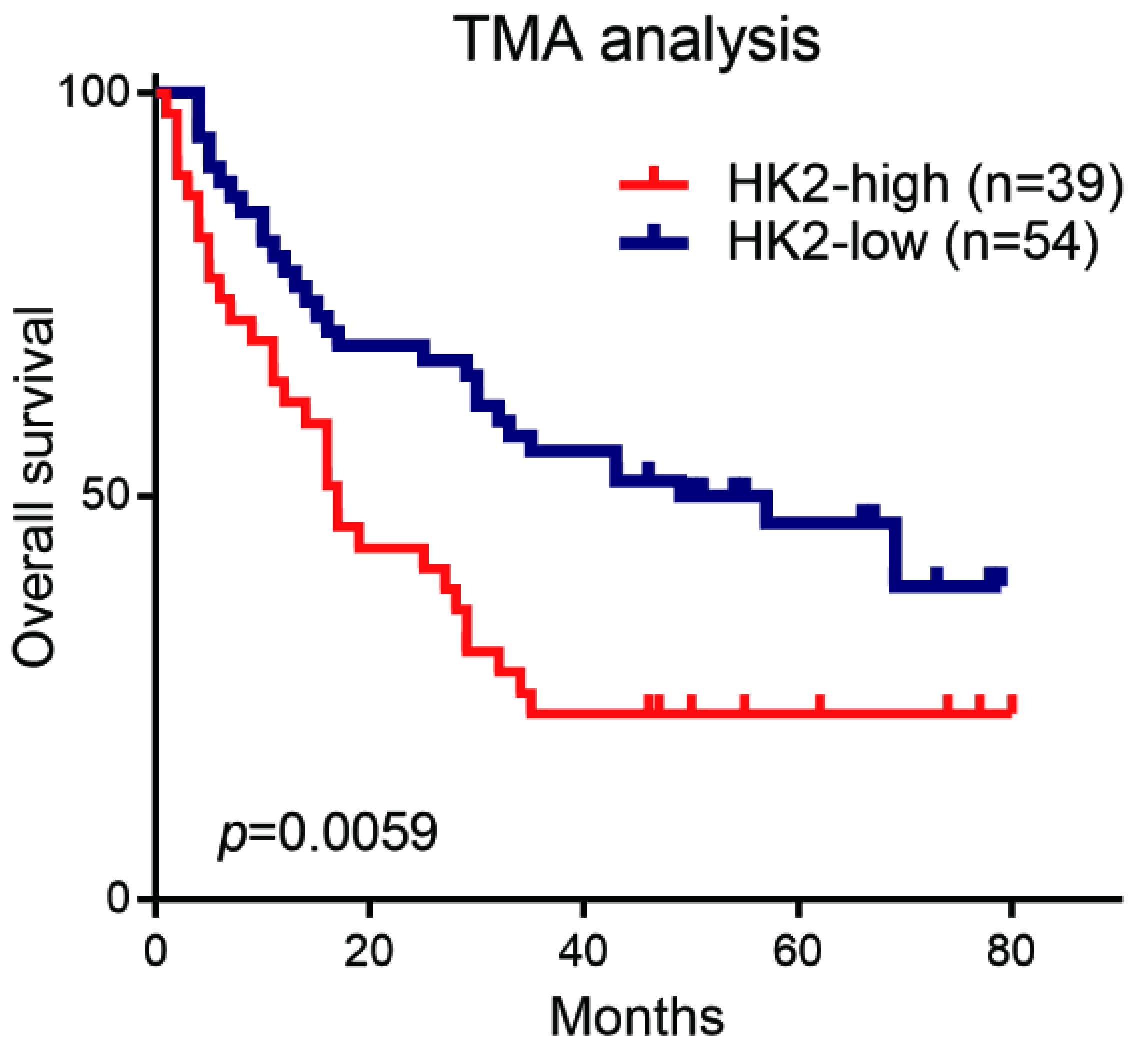
TMA analysis
100
50
0
Overall survival
HK2-high (n=39)
HK2-low (n=54)
p=0.0059
0
20
40
60
80
Months

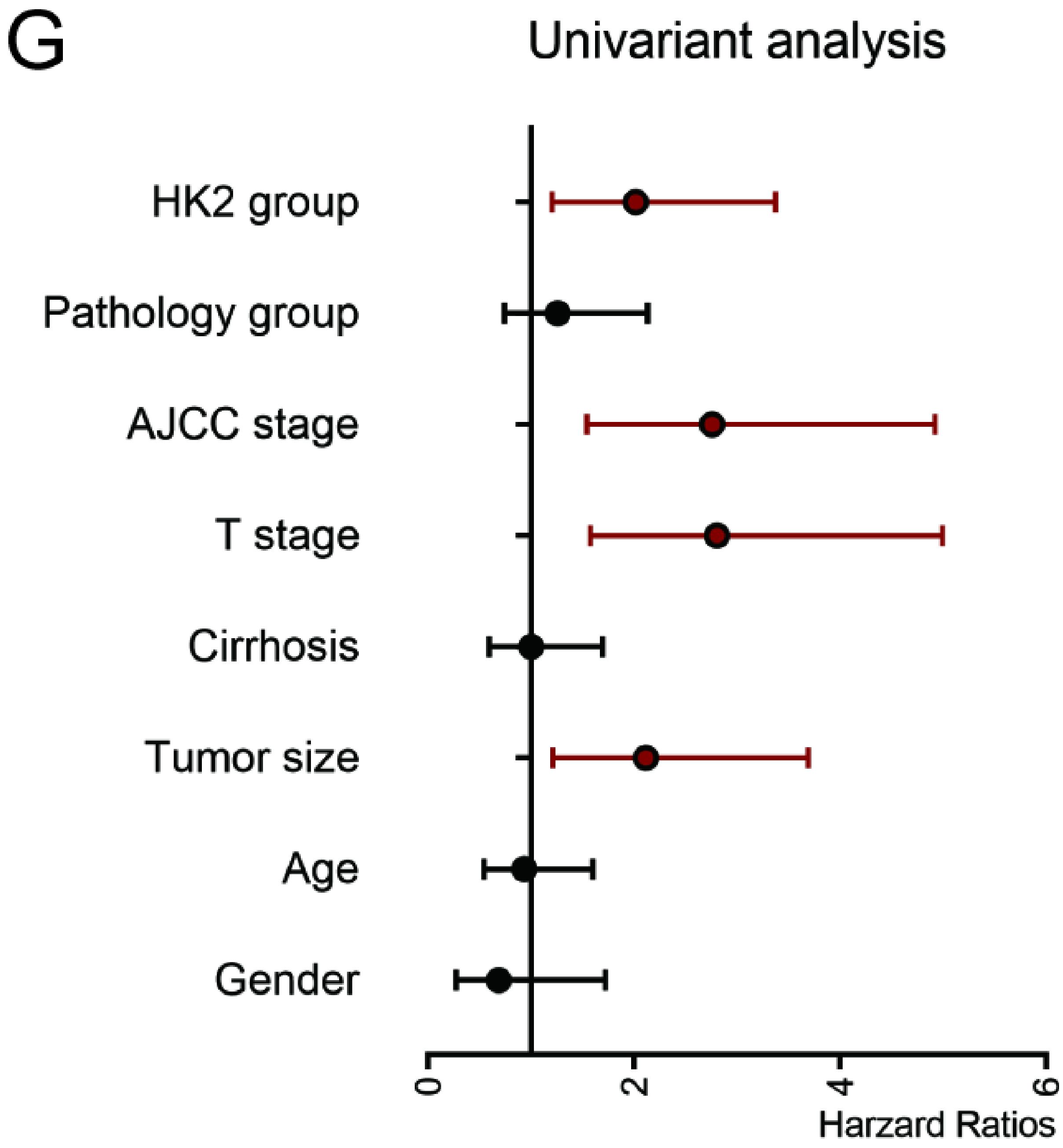
G
Univariant analysis
HK2 group
Pathology group
AJCC stage
T stage
Cirrhosis
Tumor size
Age
Gender
0
2
4
6
Harzard Ratios

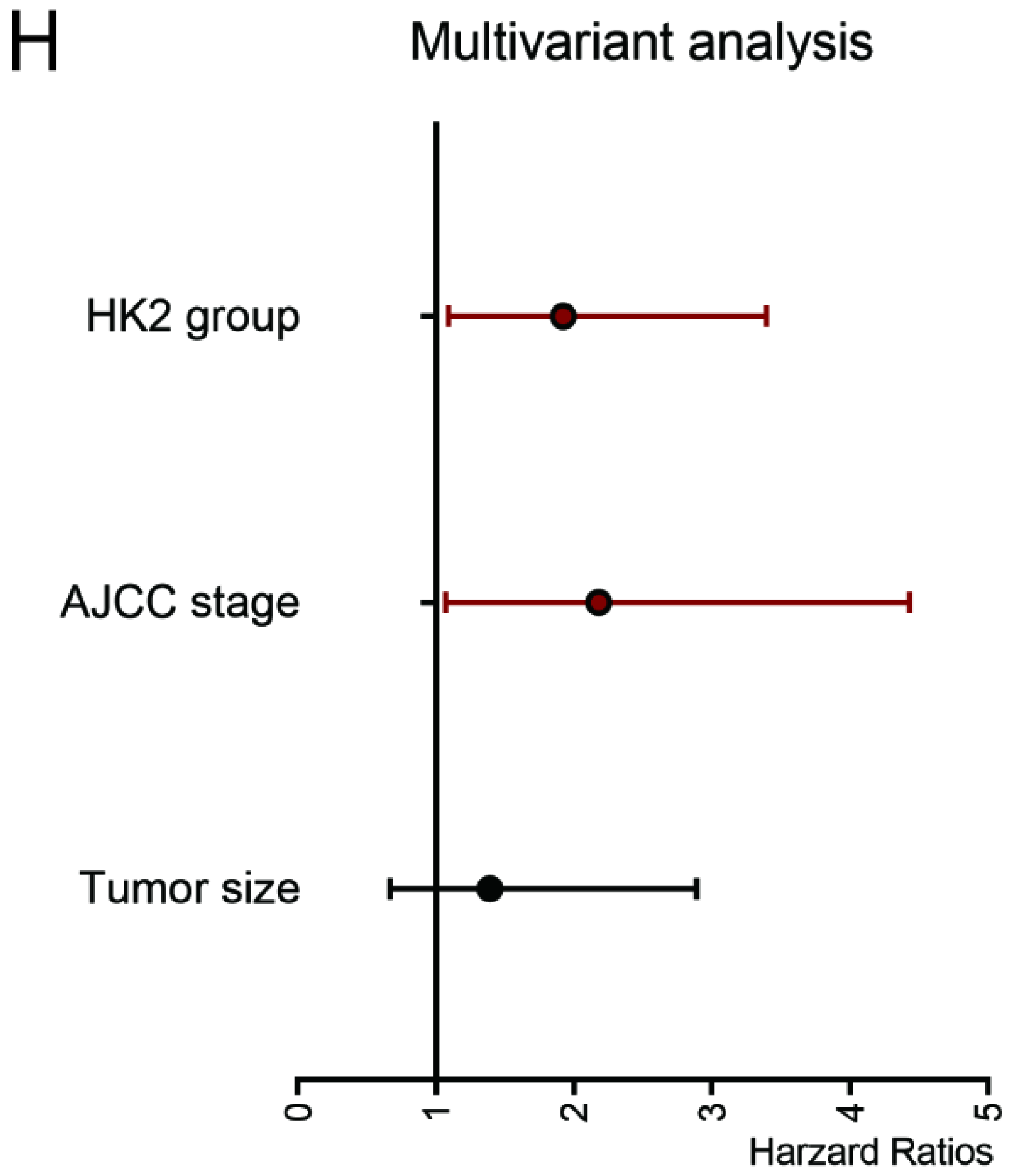
H
Multivariant analysis
HK2 group
AJCC stage
Tumor size
0
1
2
3
4
5
Harzard Ratios
