# Supplementary figures and images for: Hexokinase 2 confers radio-resistance in hepatocellular carcinoma by promoting autophagy-dependent degradation of AIMP2
Source: Cell Death Dis. 2023 Aug 1;14(8):488. doi: 10.1038/s41419-023-06009-2 (PMC10390495; doi:10.1038/s41419-023-06009-2)

A

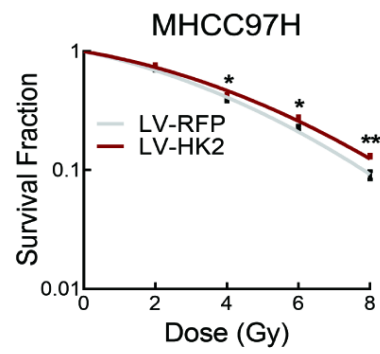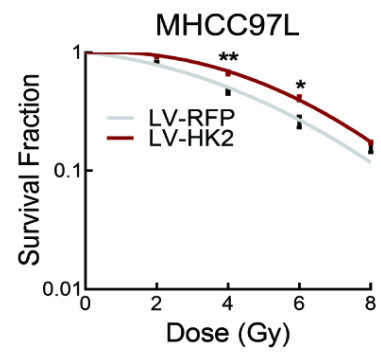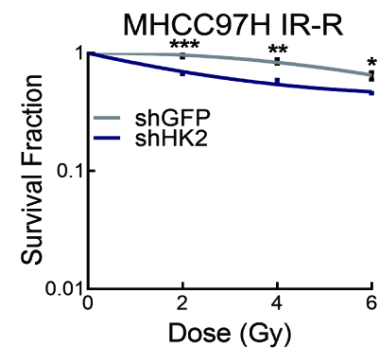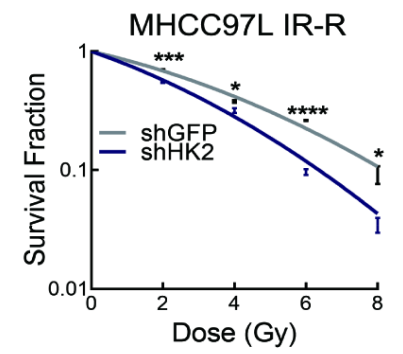

B

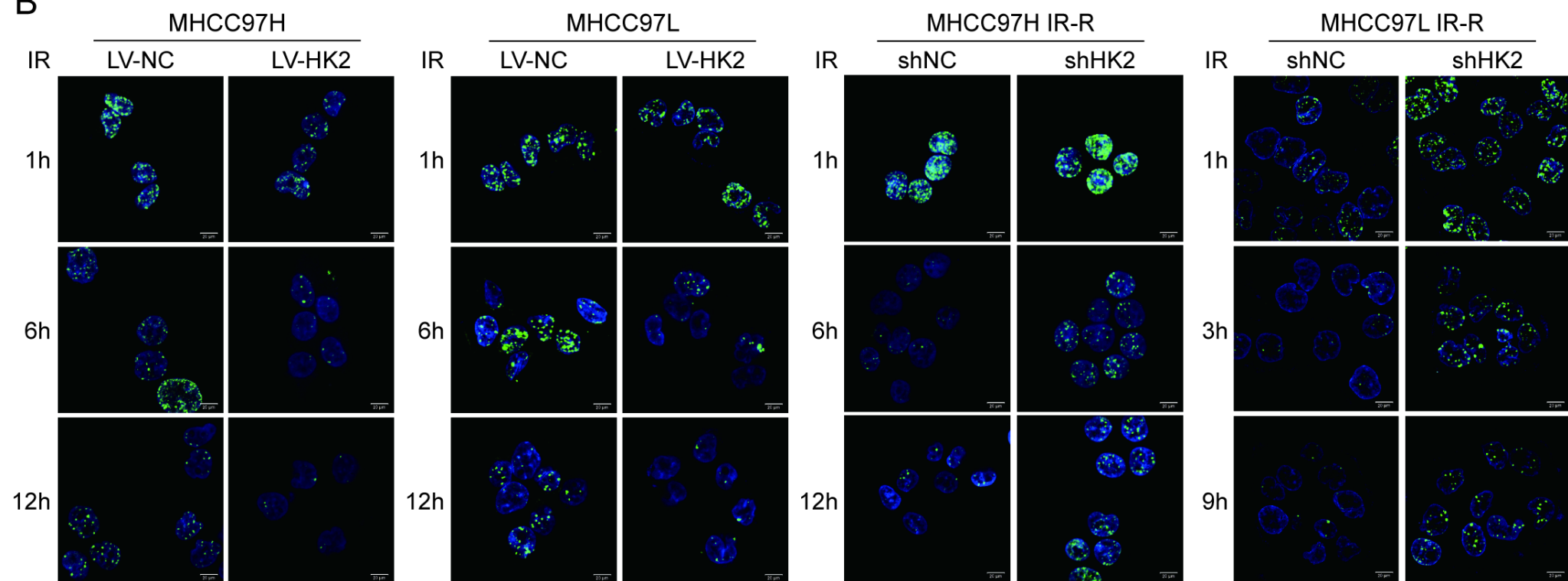

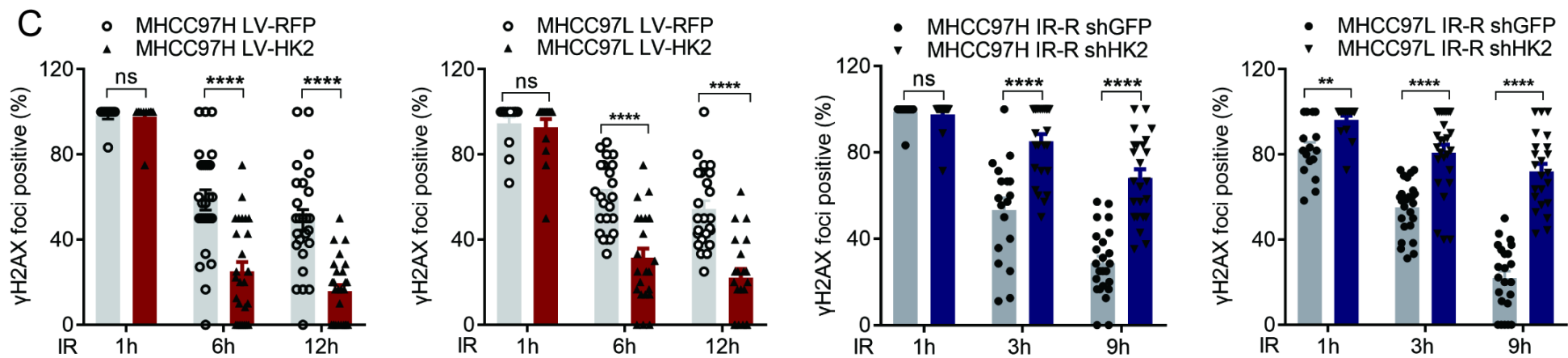

D

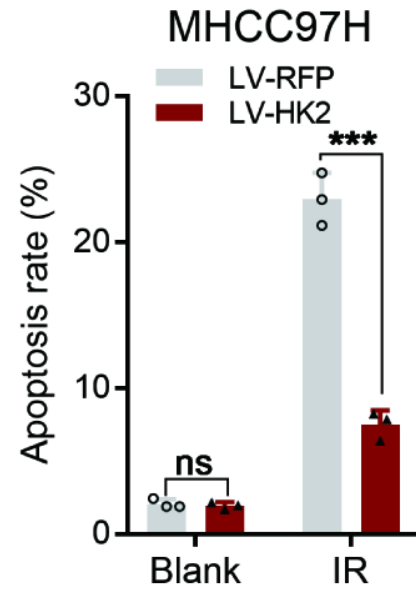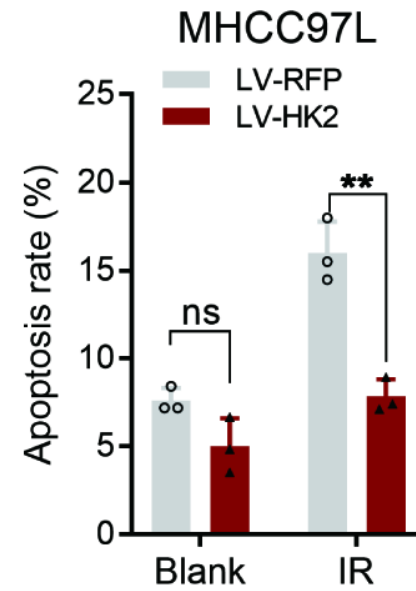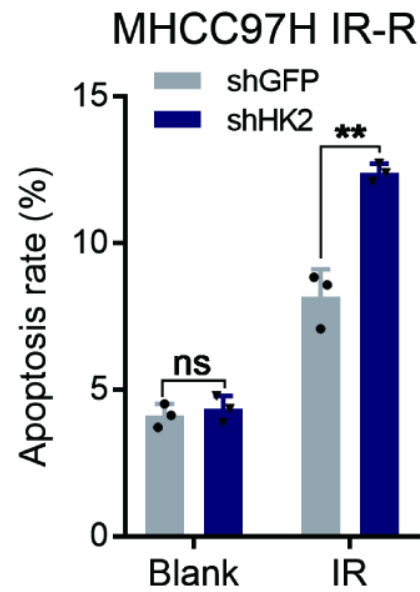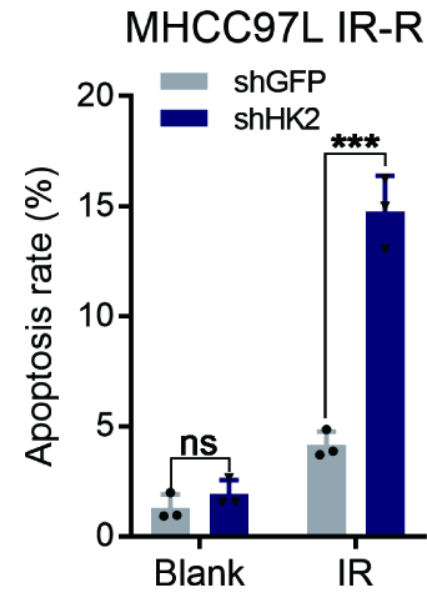

E

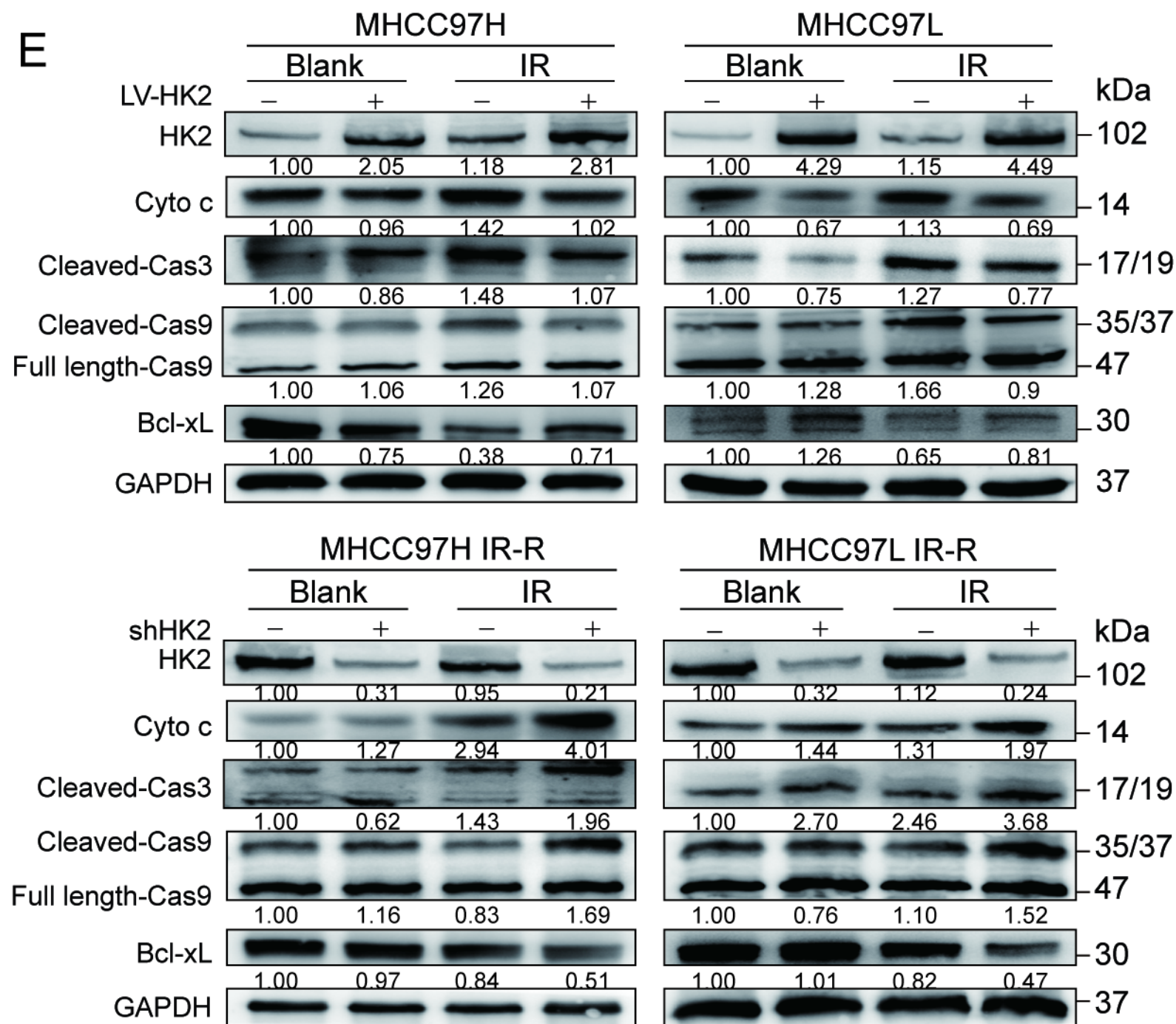

Supplement: Supplementary file 2 — Extended Figure2 (Figure2 merge file) [file 41419_2023_6009_MOESM2_ESM.pdf]

A

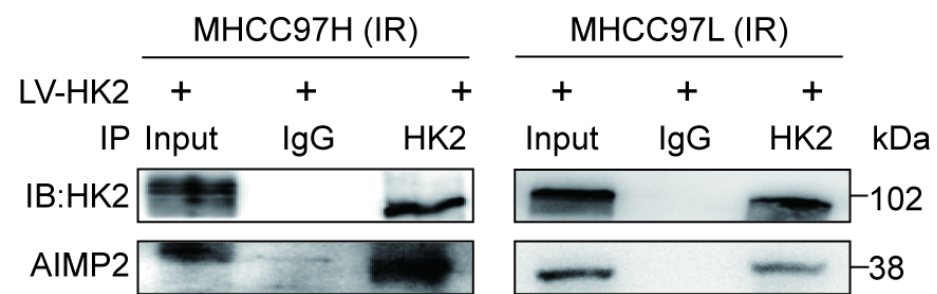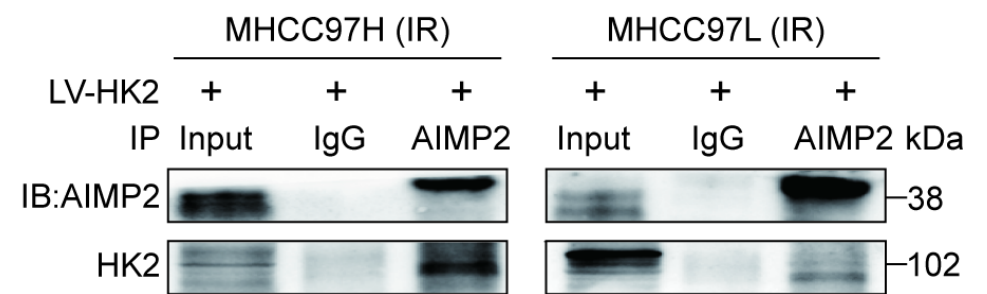

B

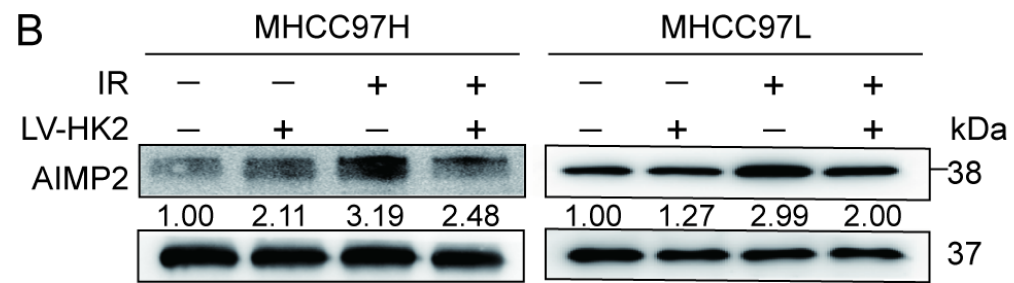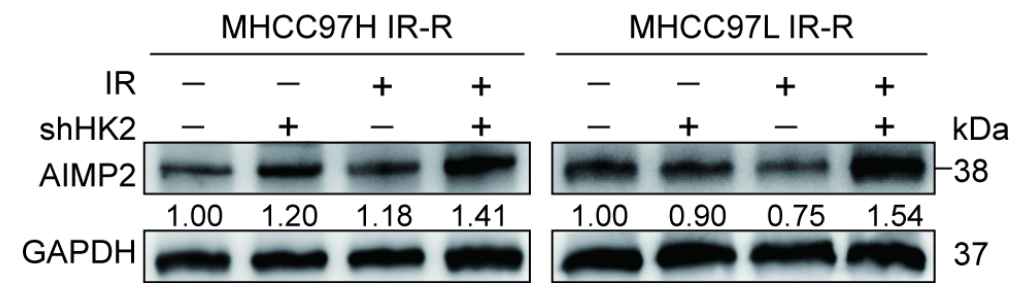

C

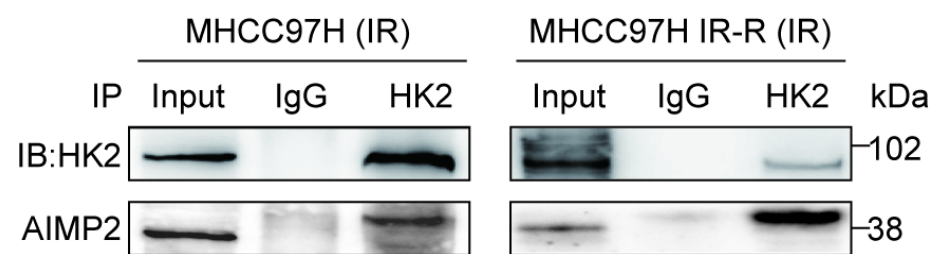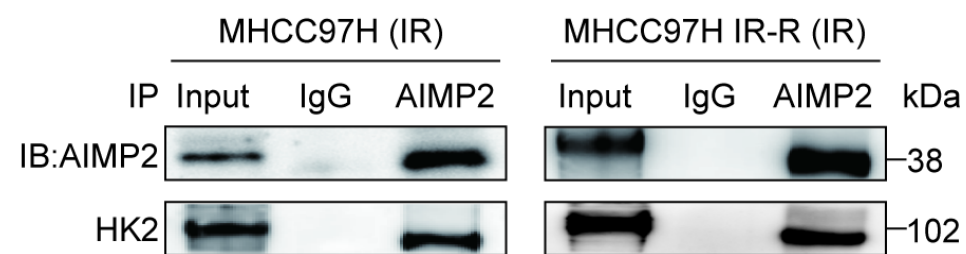

D

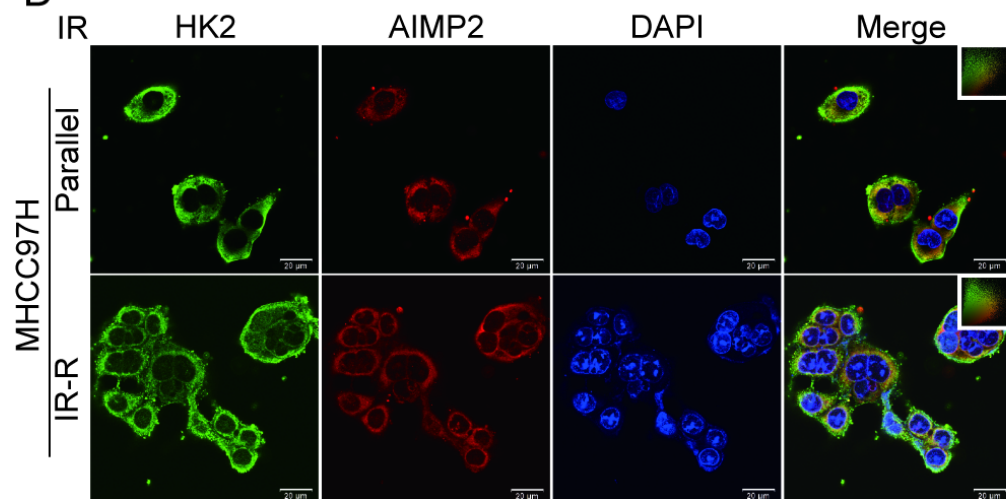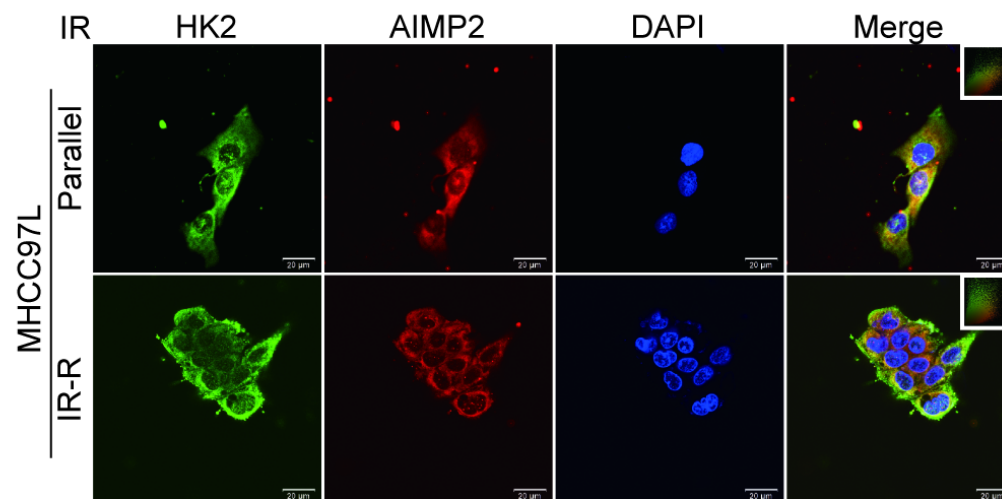

E

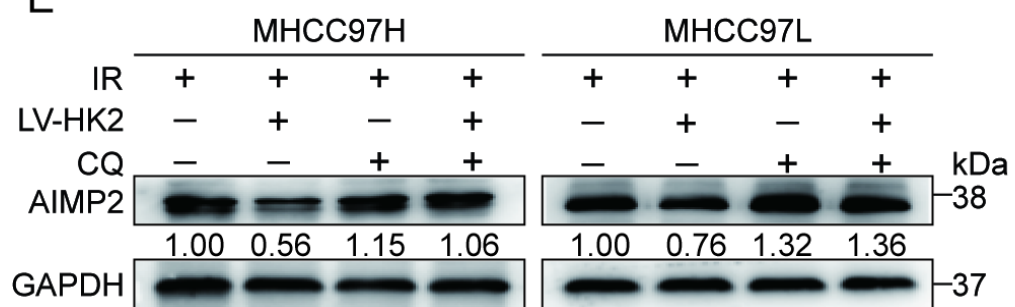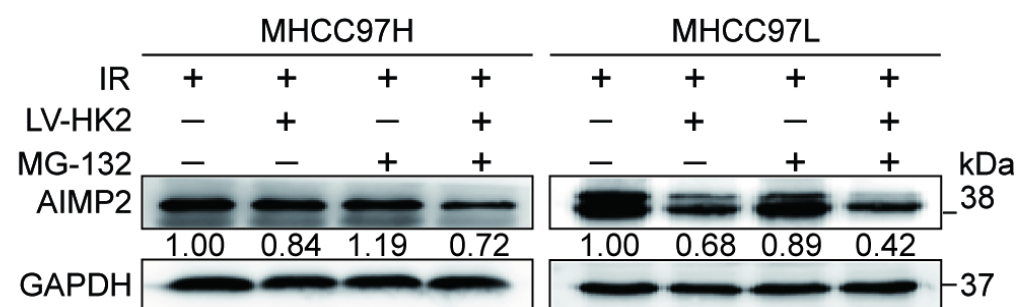

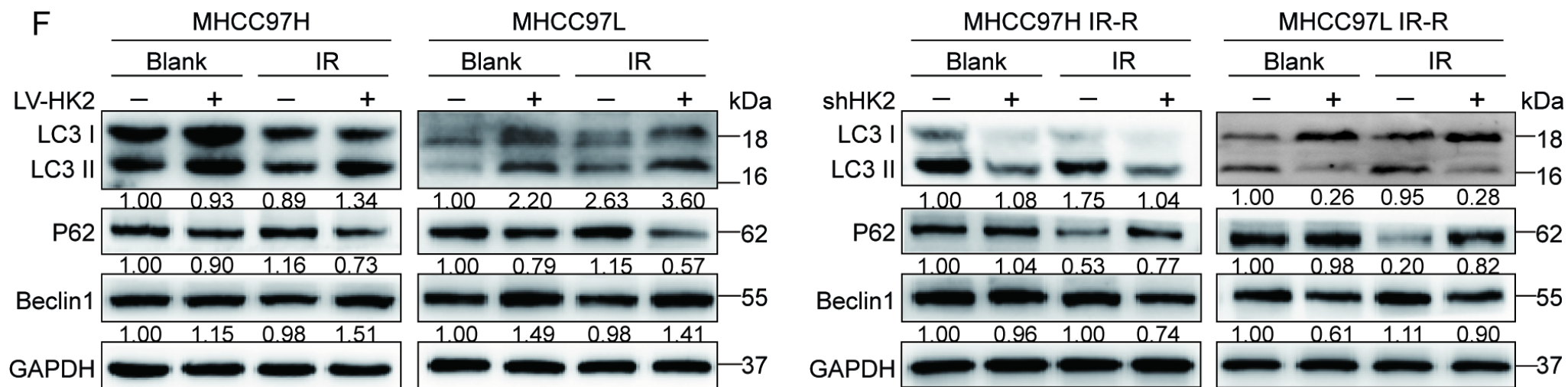

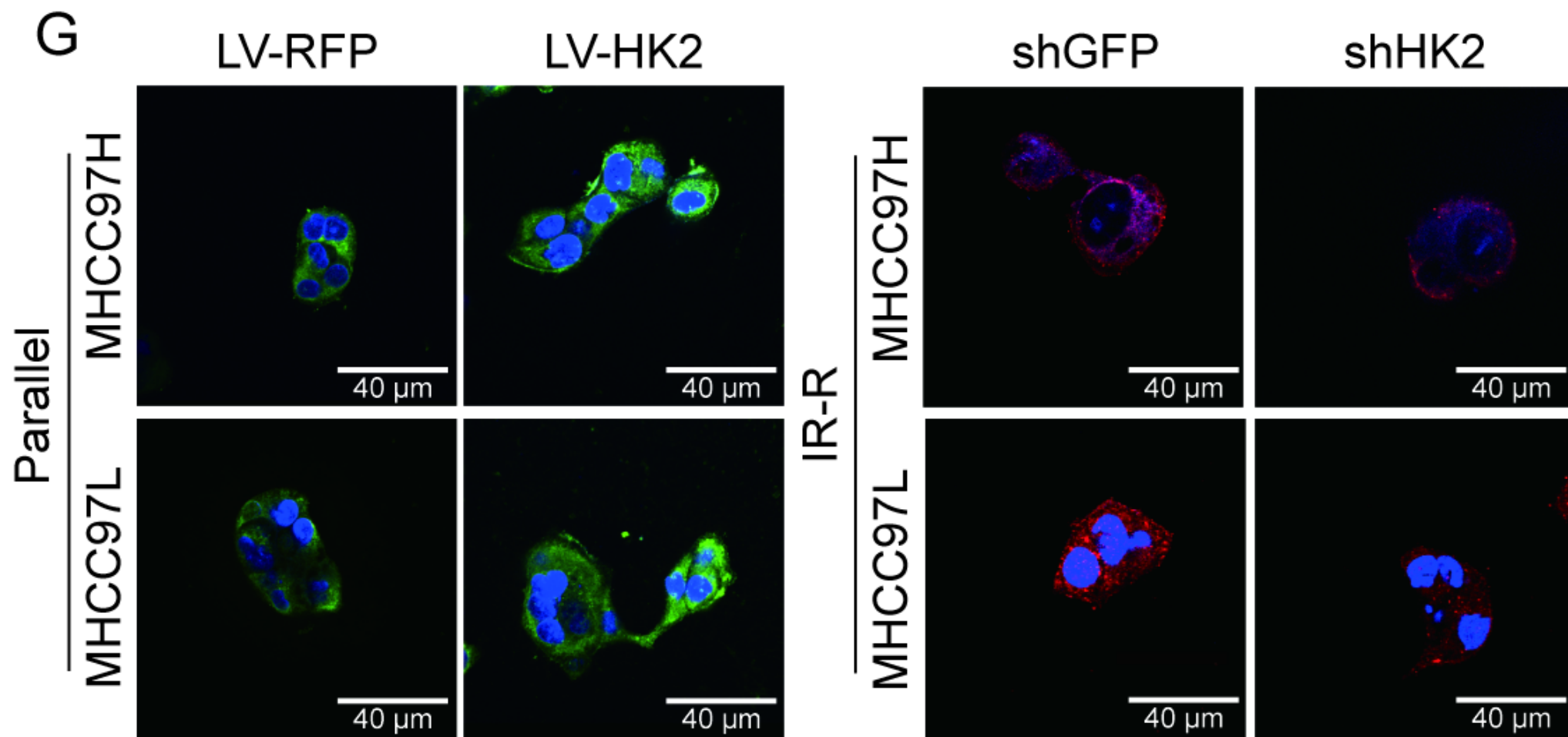

H

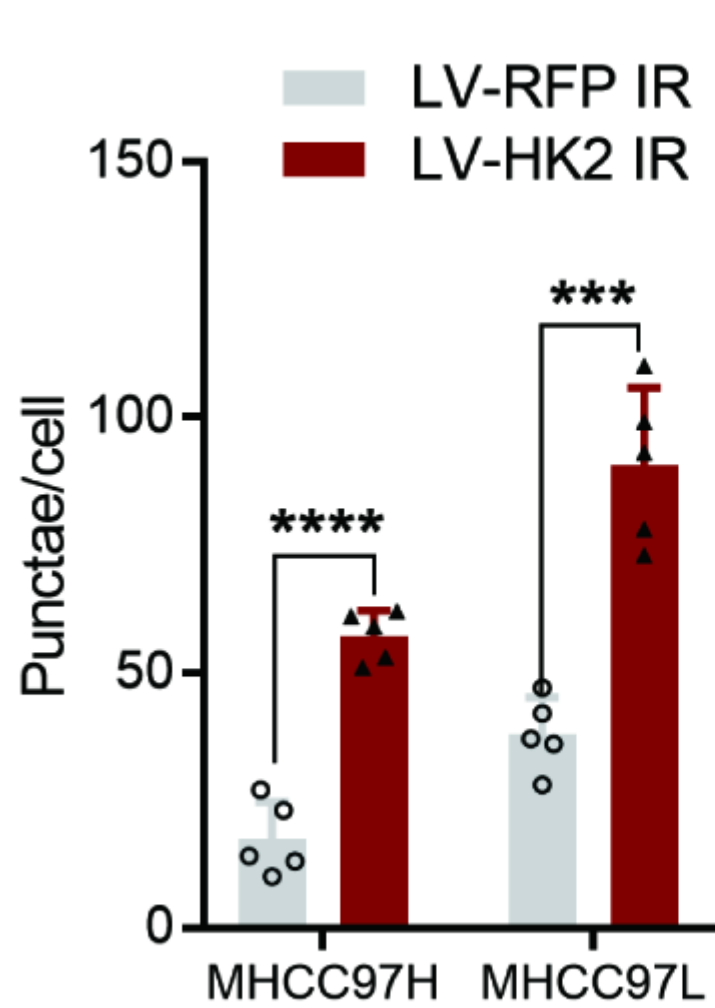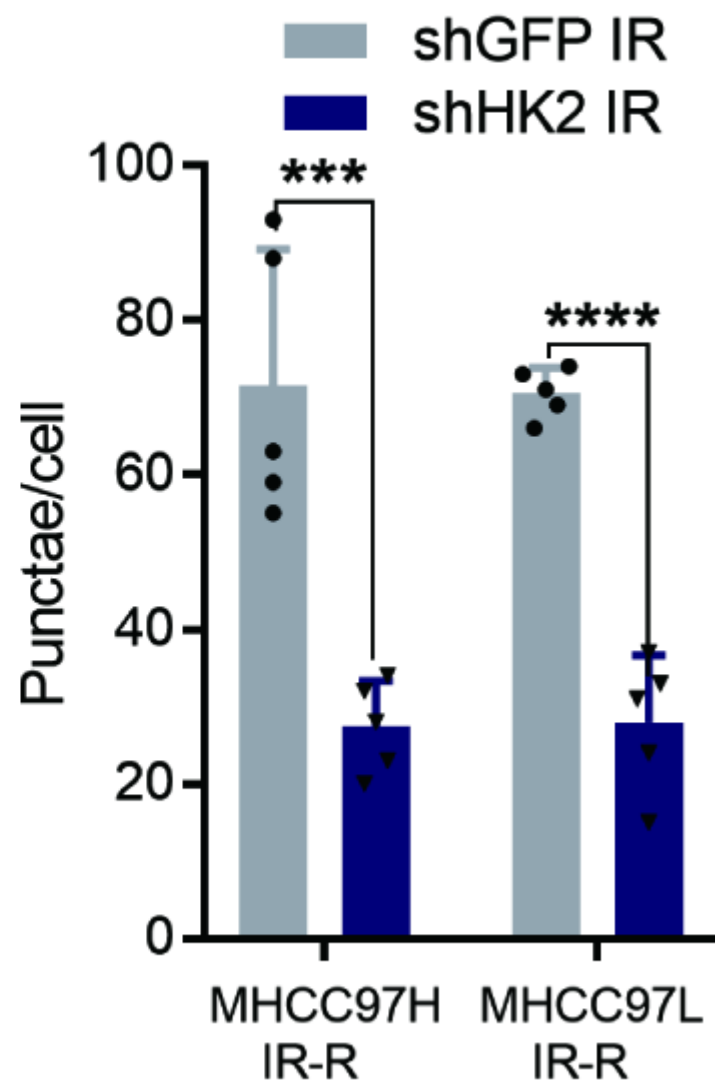

I

MHCC97H

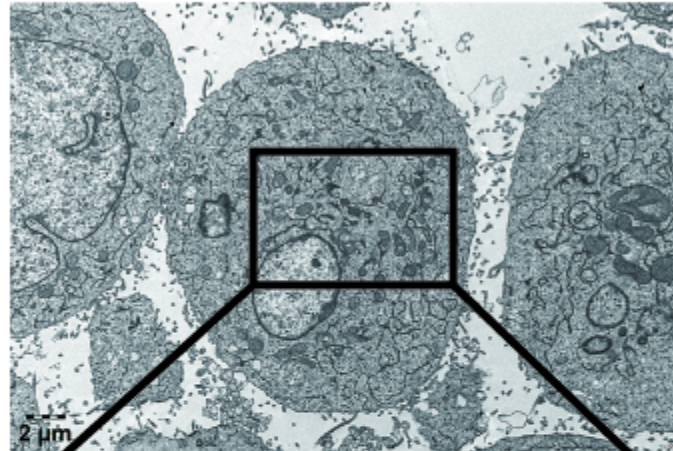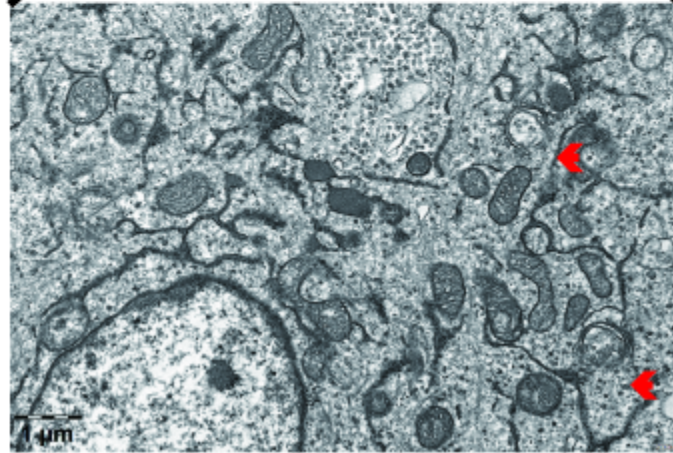

MHCC97H IR-R

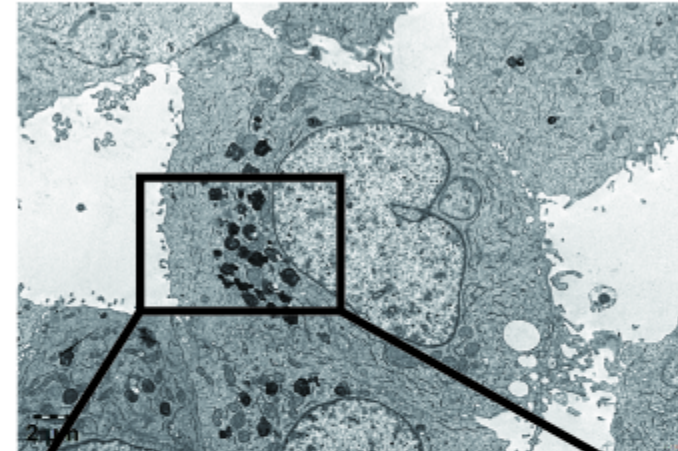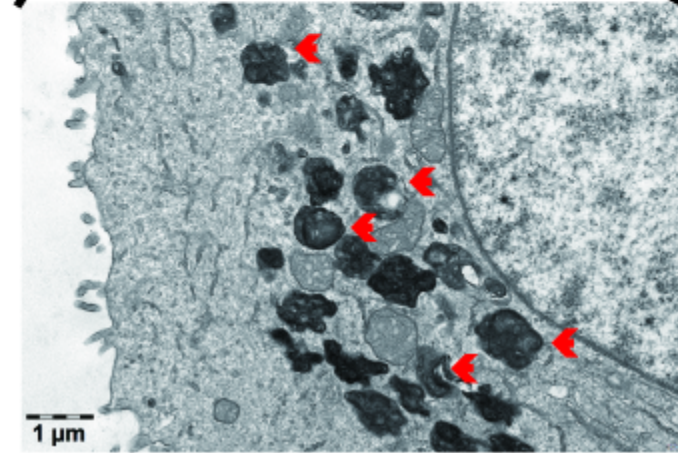

J

MHCC97H

IR

mCherry

GFP

DAPI

Merge

Parallel

IR-R

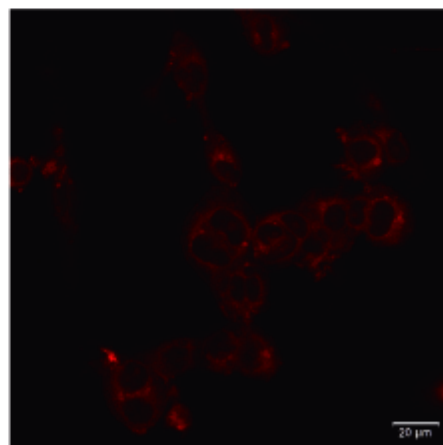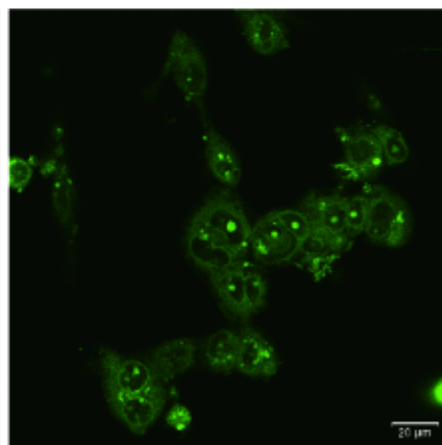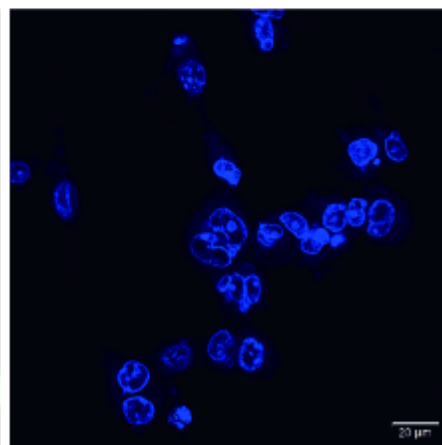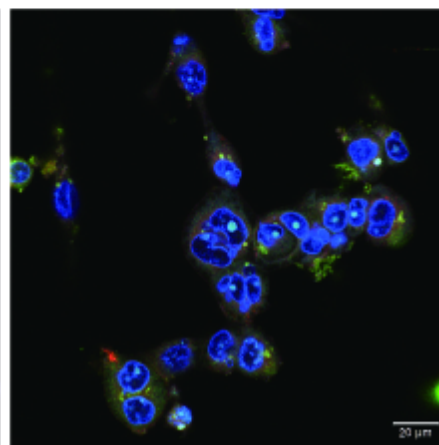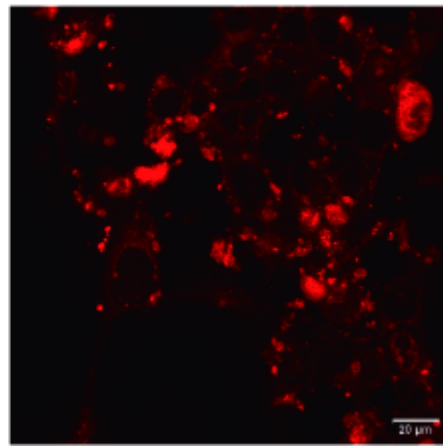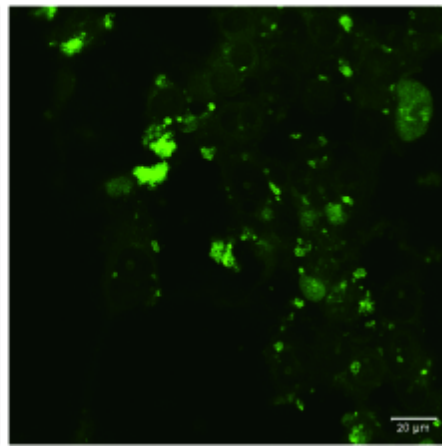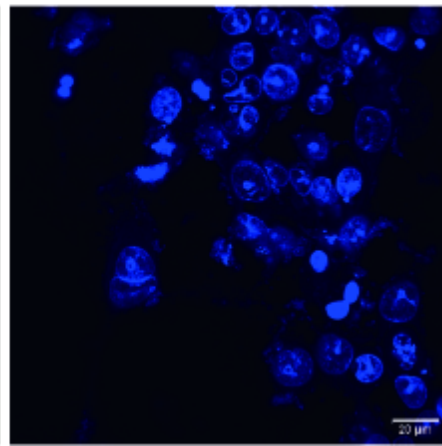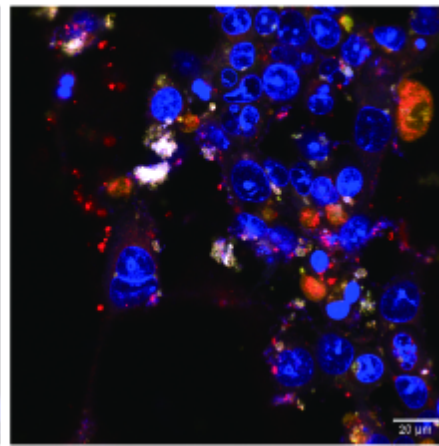

Supplement: Supplementary file 4 — Extended Figure4 (Figure4 merge file) [file 41419_2023_6009_MOESM4_ESM.pdf]

A

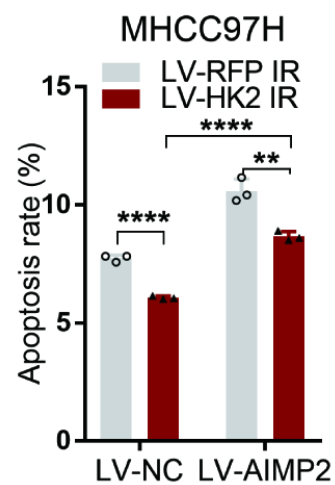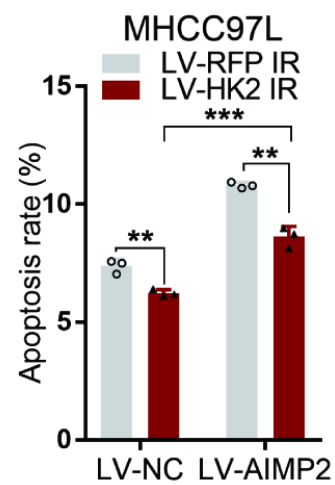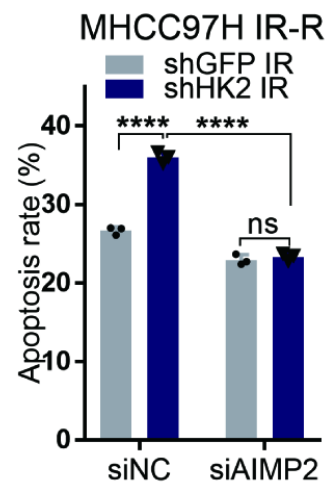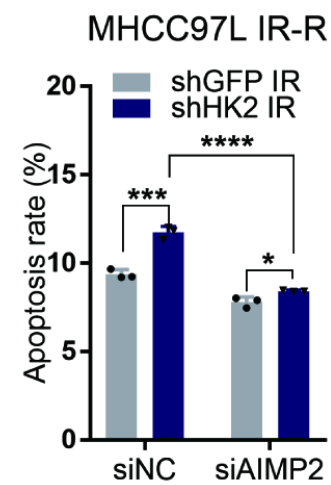

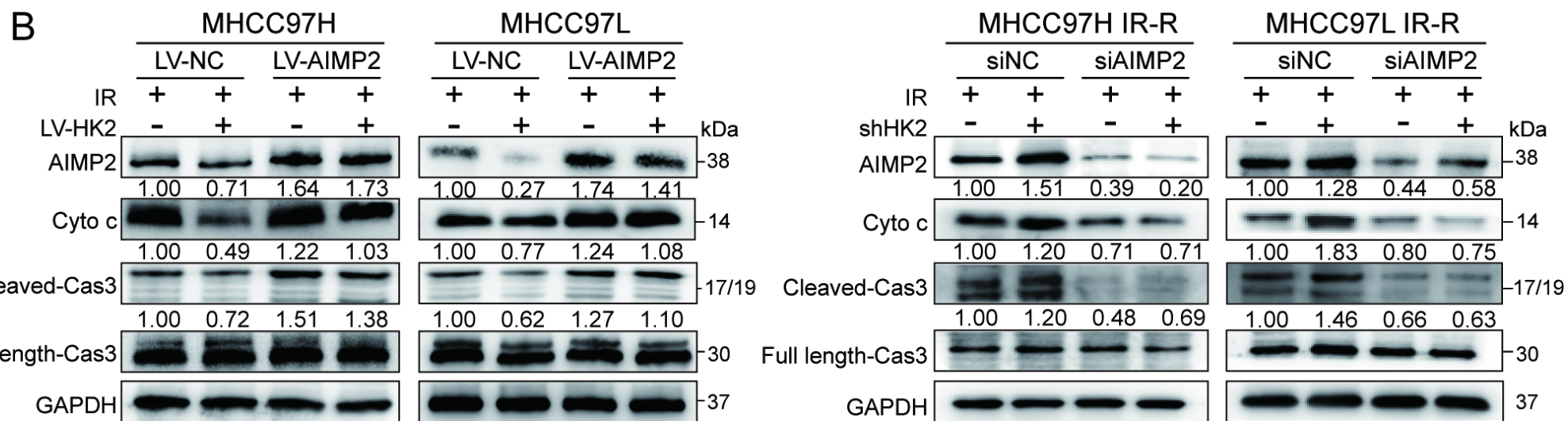

C

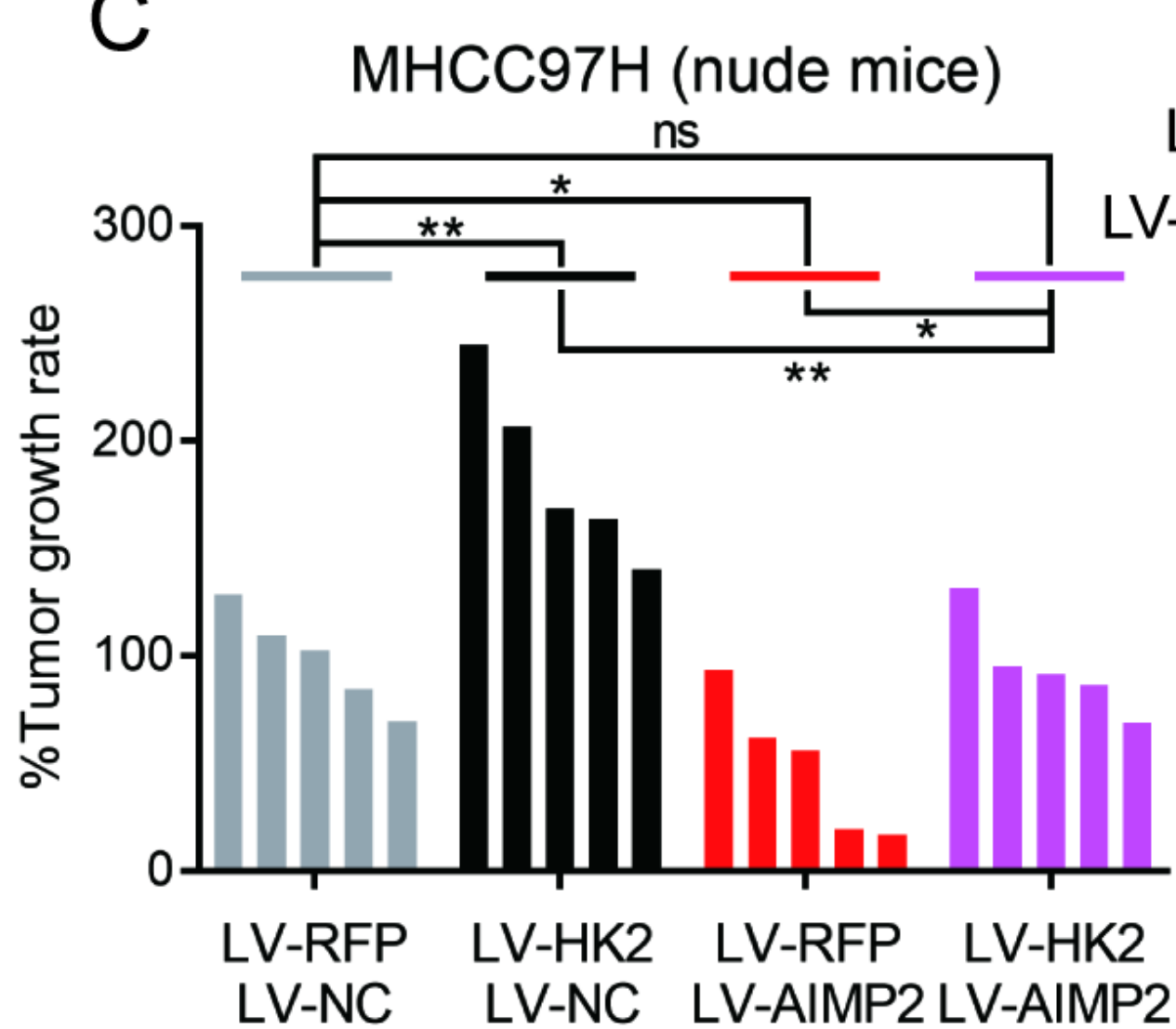

|          |   |   |   |   |
|----------|---|---|---|---|
| IR       | + | + | + | + |
| LV-HK2   | - | + | - | + |
| LV-AIMP2 | - | - | + | + |

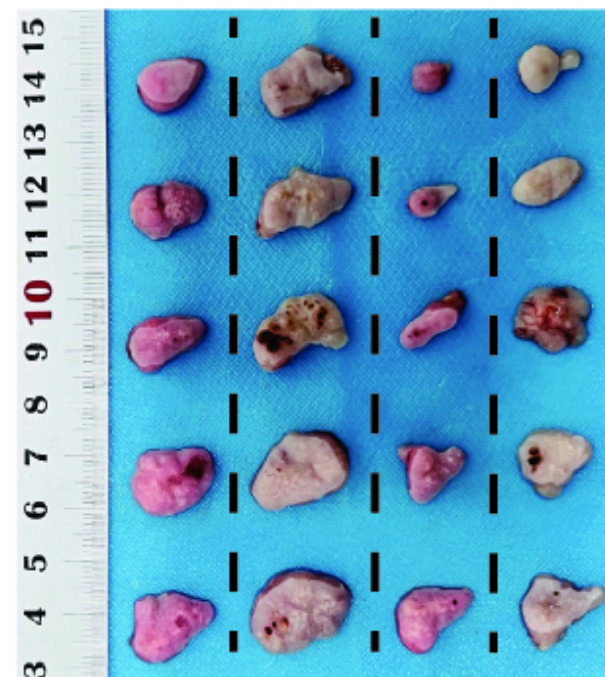

D

MHCC97H

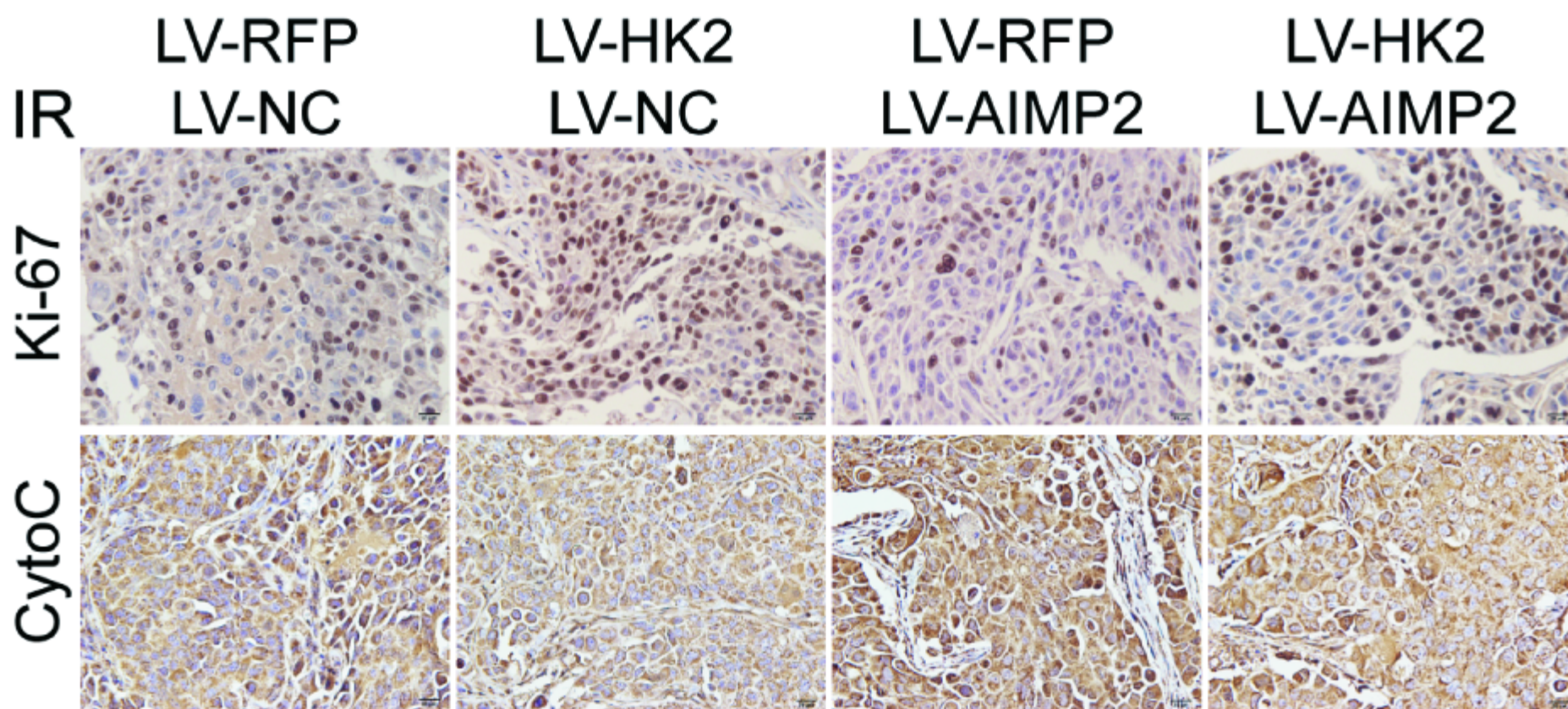

Supplement: Supplementary file 5 — Extended Figure5 (Figure5 merge file) [file 41419_2023_6009_MOESM5_ESM.pdf]

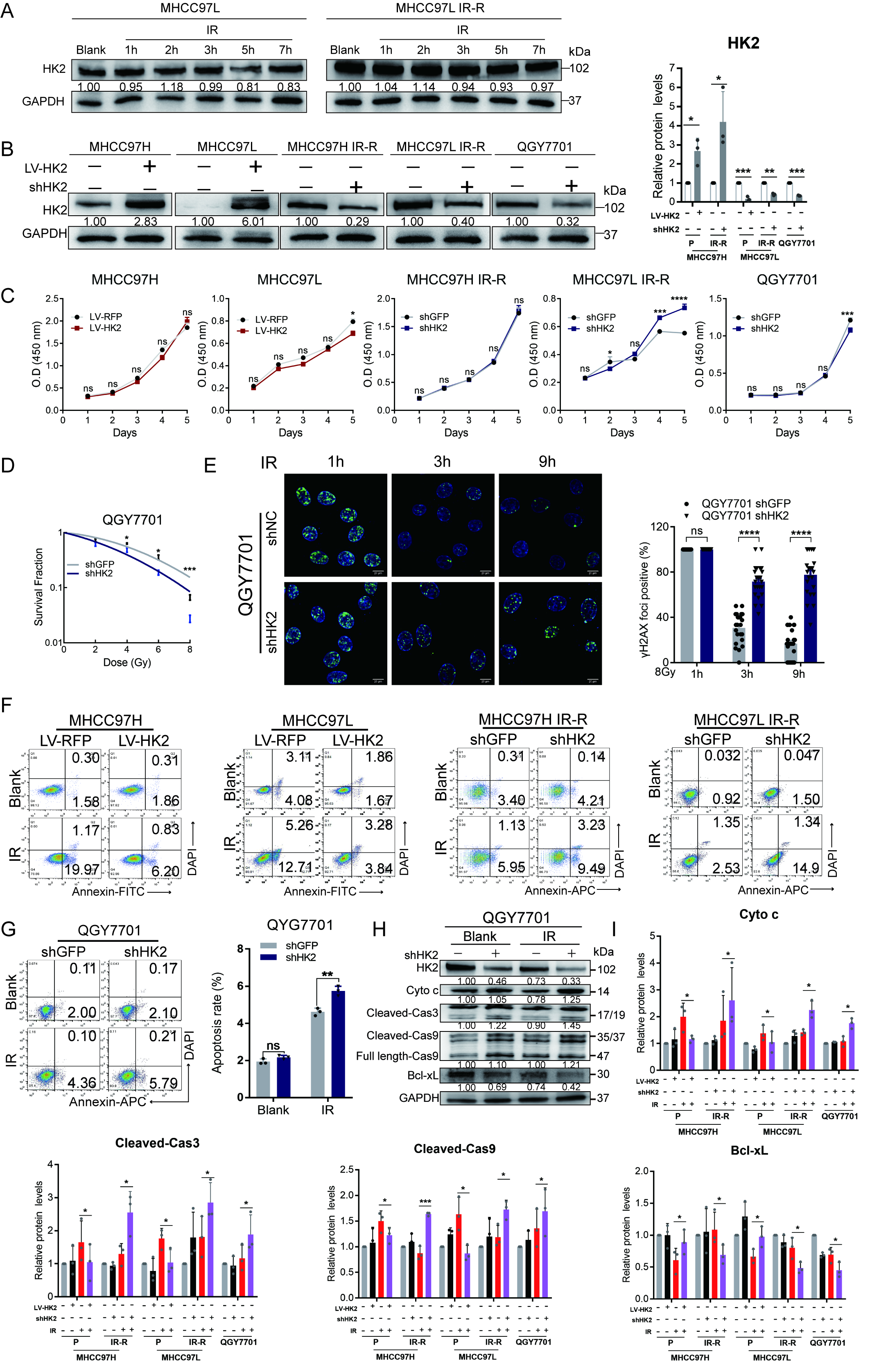

Supplement: Supplementary file 9 — Supplementary Figure1 [file 41419_2023_6009_MOESM9_ESM.tif]

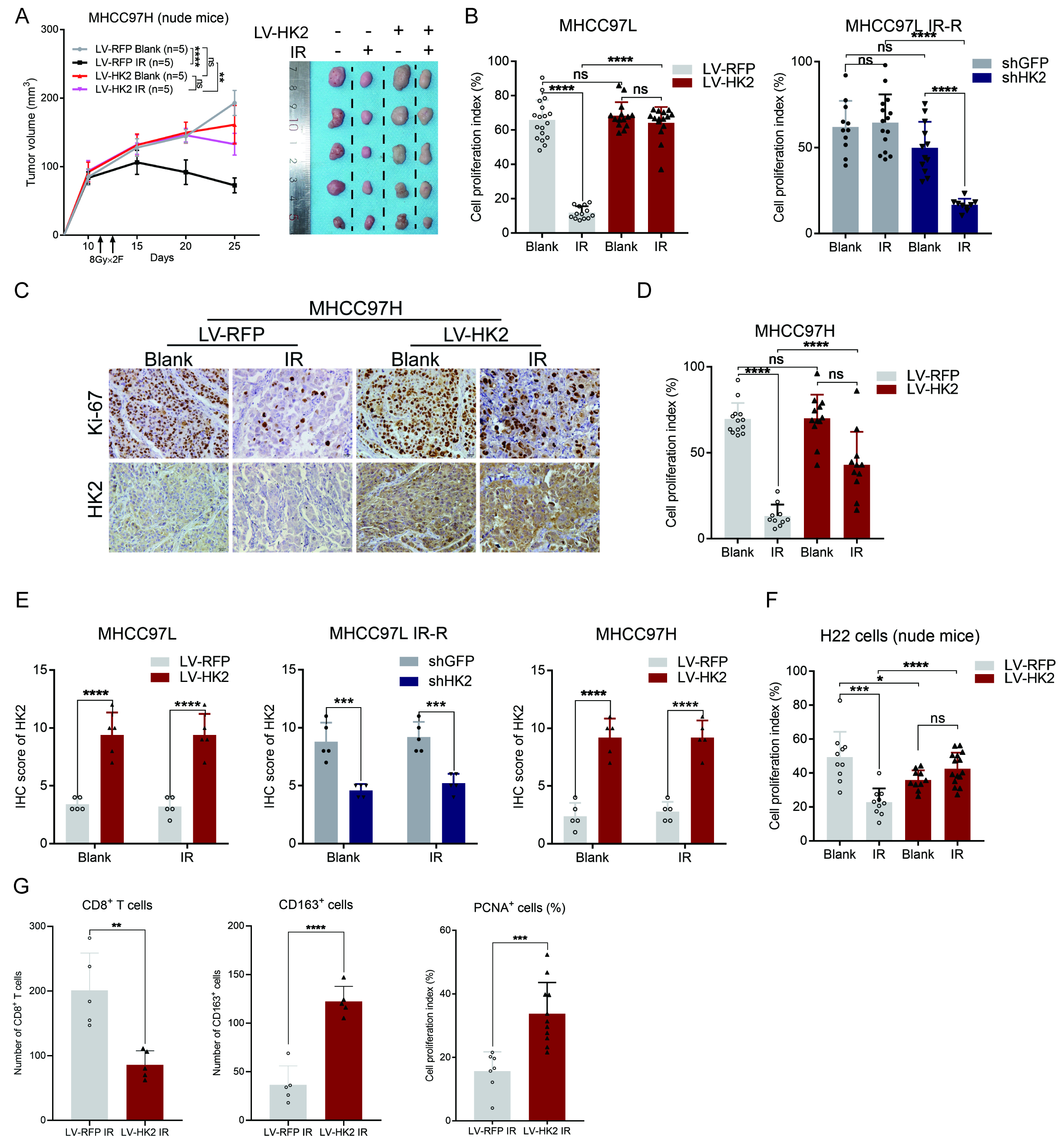

Supplement: Supplementary file 10 — Supplementary Figure2 [file 41419_2023_6009_MOESM10_ESM.tif]

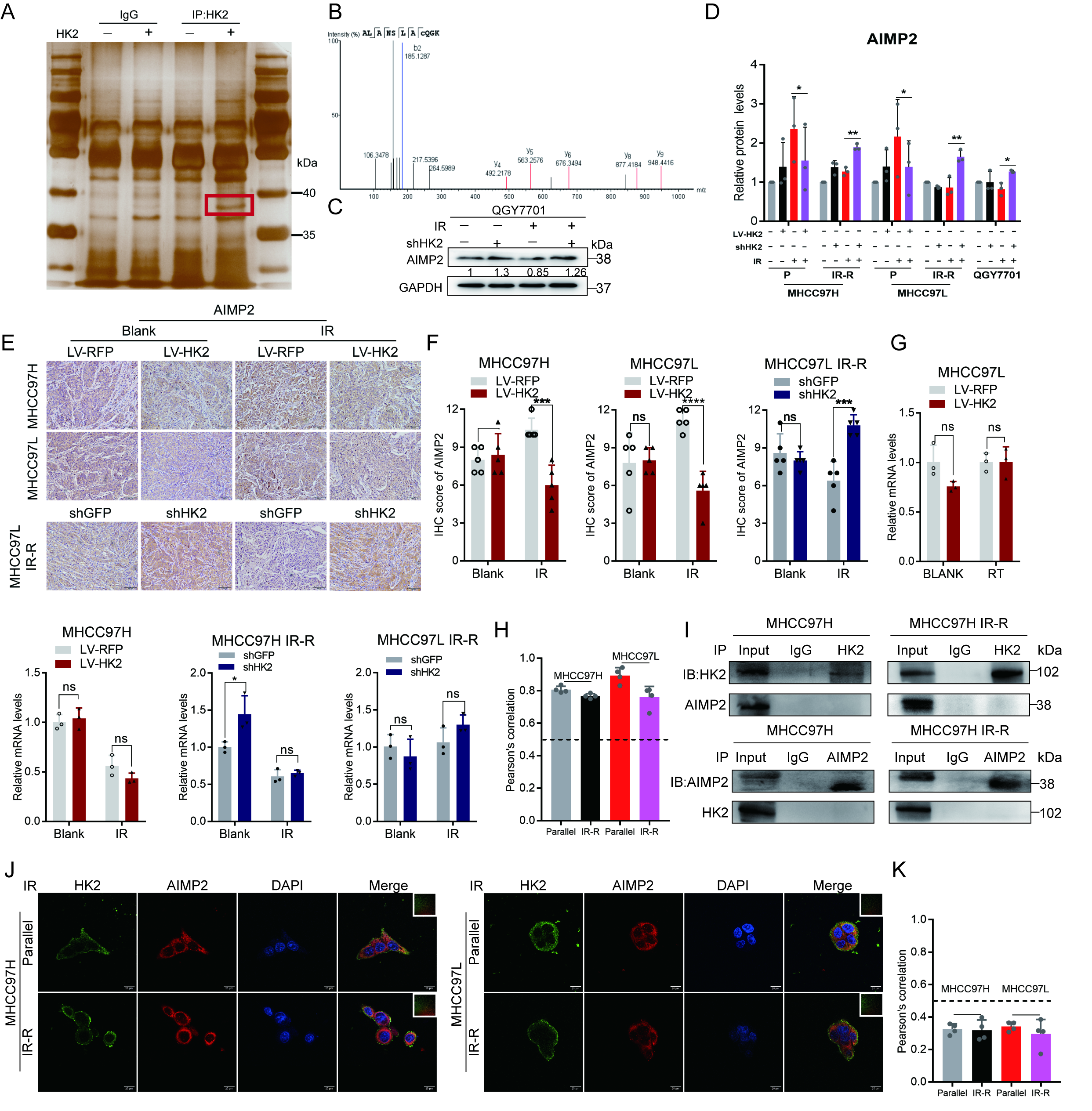

Supplement: Supplementary file 11 — Supplementary Figure3 [file 41419_2023_6009_MOESM11_ESM.tif]

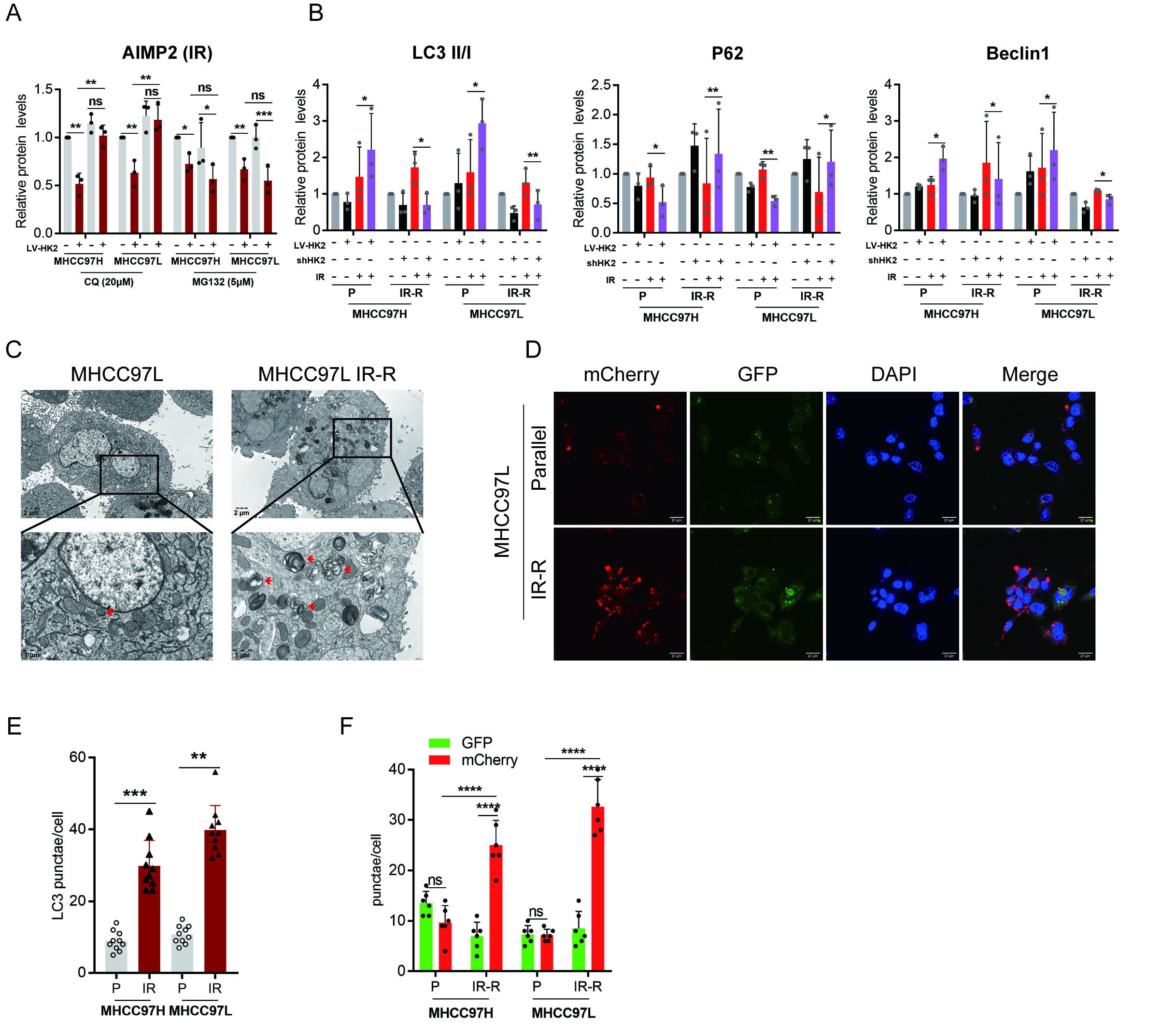

Supplement: Supplementary file 12 — Supplementary Figure4 [file 41419_2023_6009_MOESM12_ESM.tif]

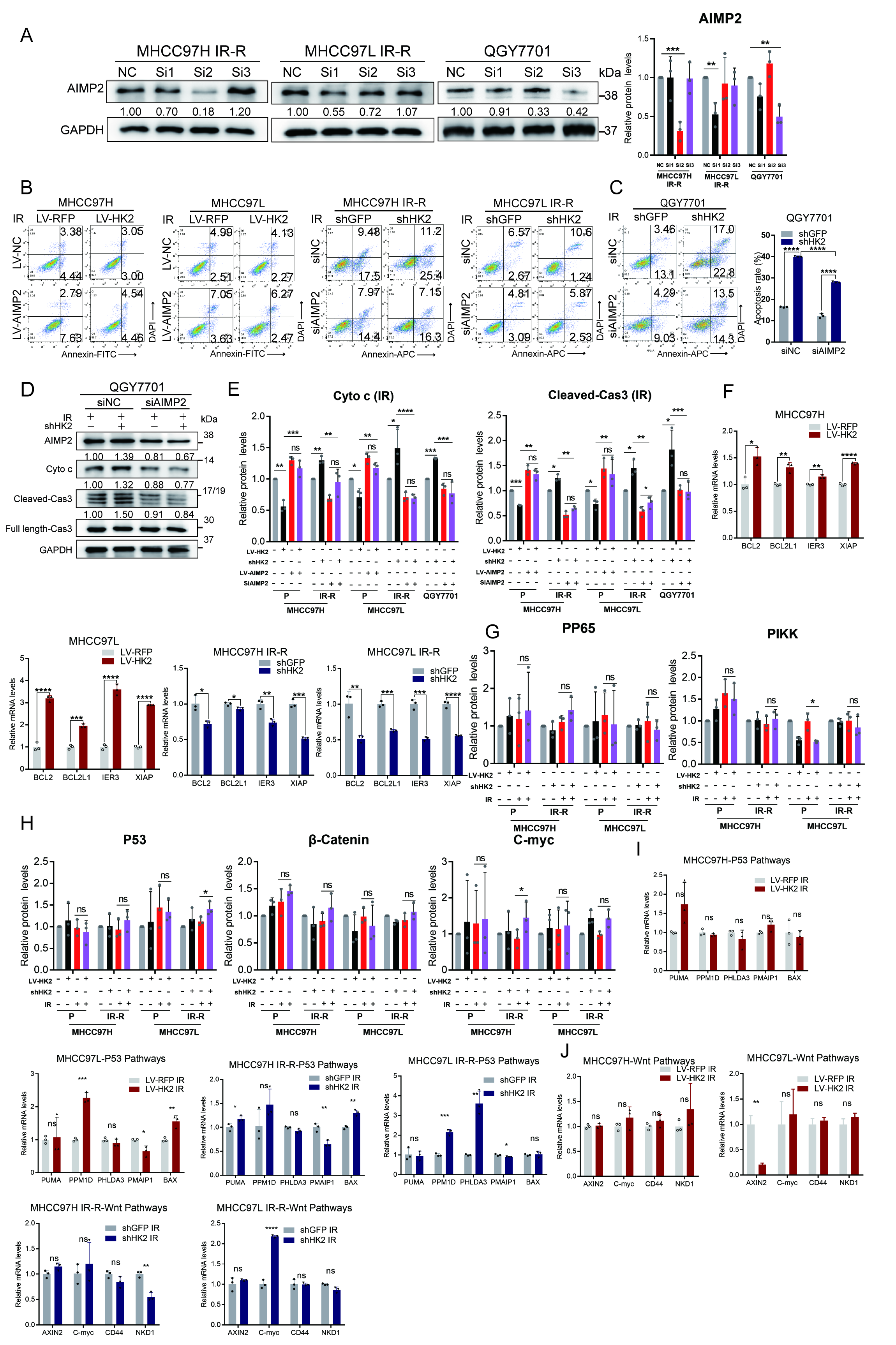

Supplement: Supplementary file 13 — Supplementary Figure5 [file 41419_2023_6009_MOESM13_ESM.tif]

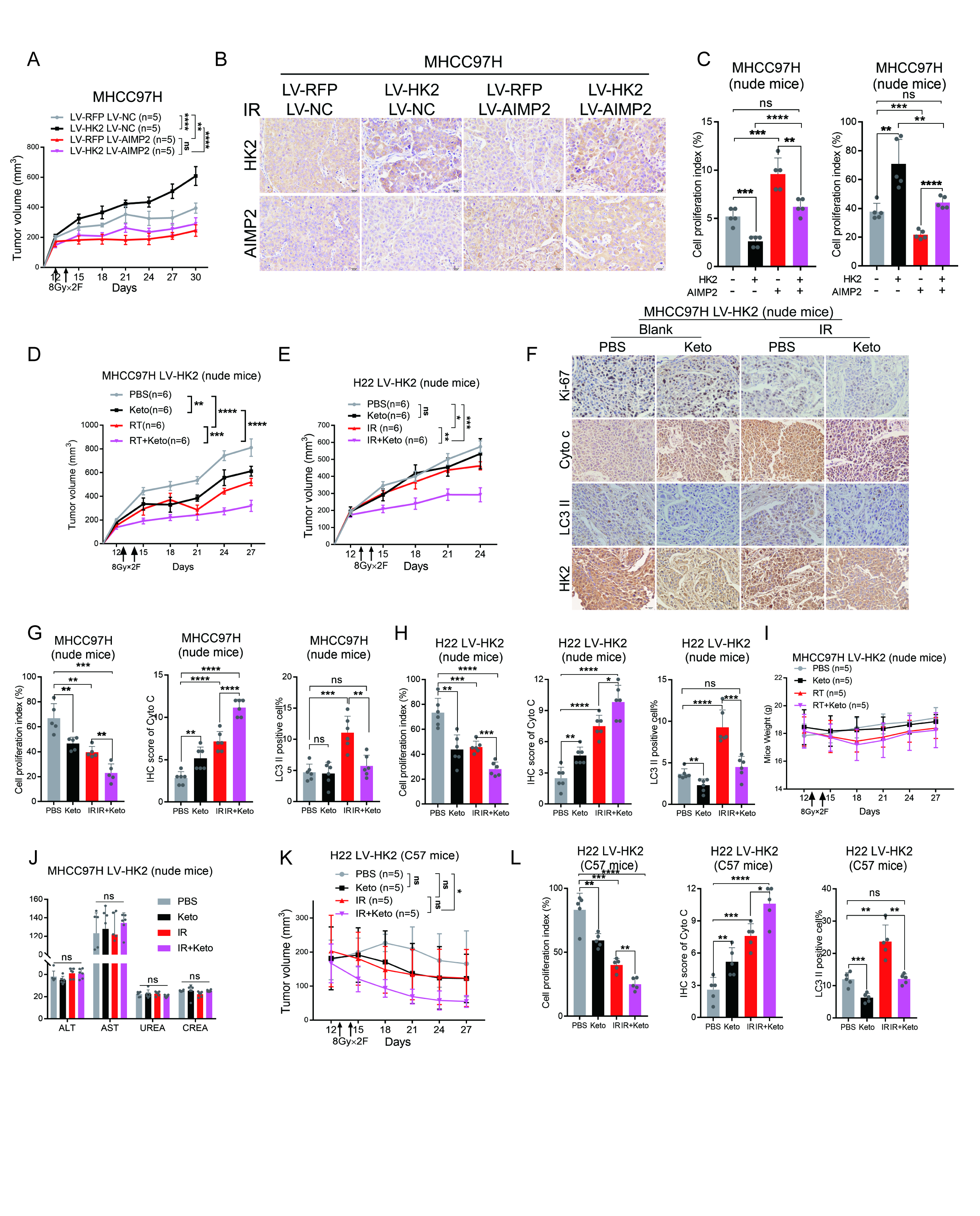

Supplement: Supplementary file 14 — Supplementary Figure6 [file 41419_2023_6009_MOESM14_ESM.tif]

A

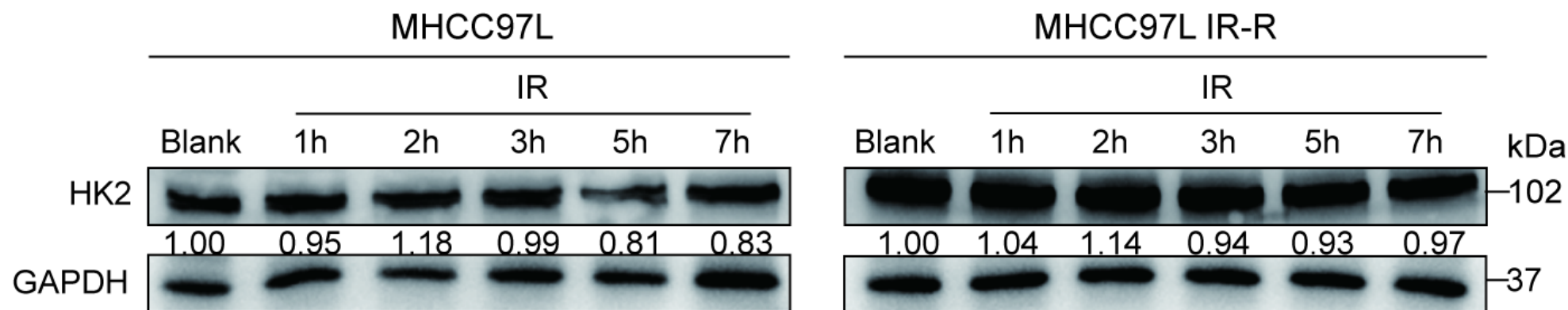

**B**

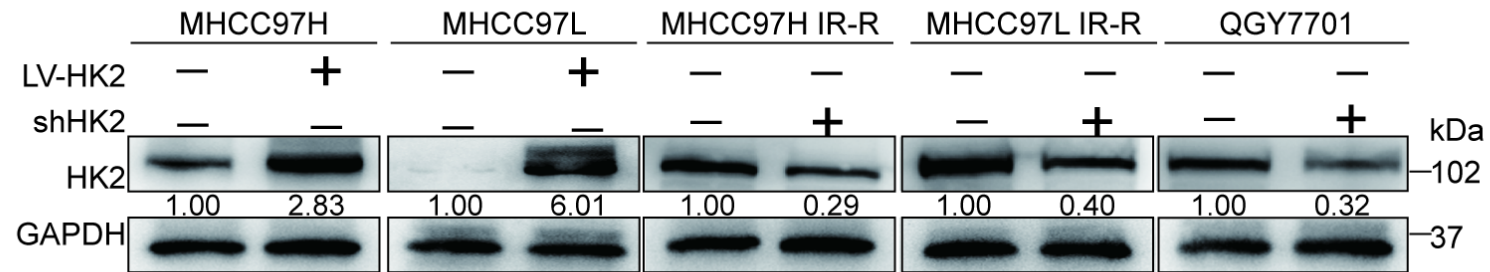

**HK2**

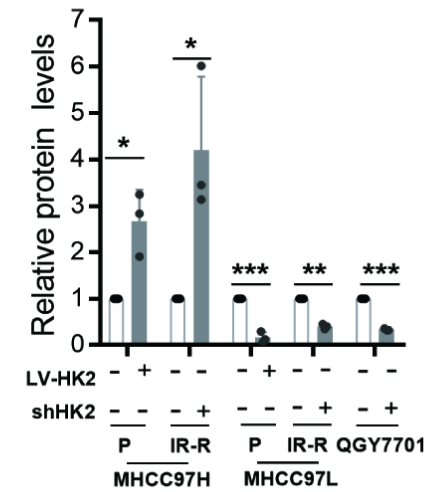

C

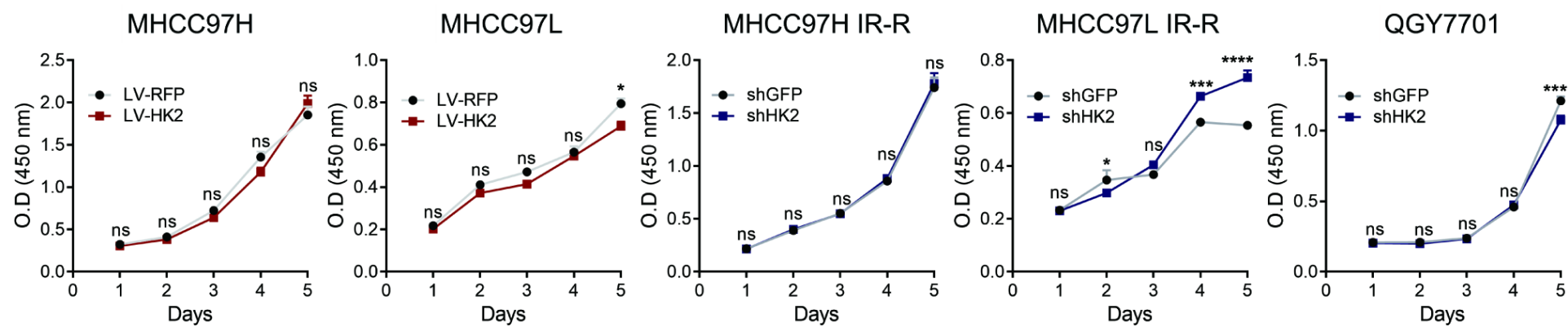

D

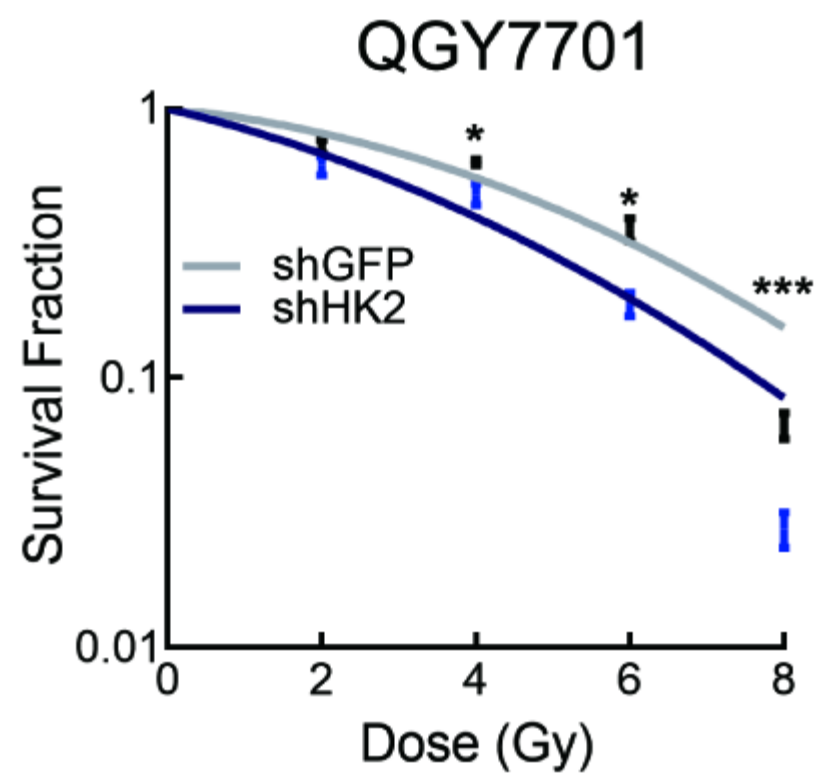

E

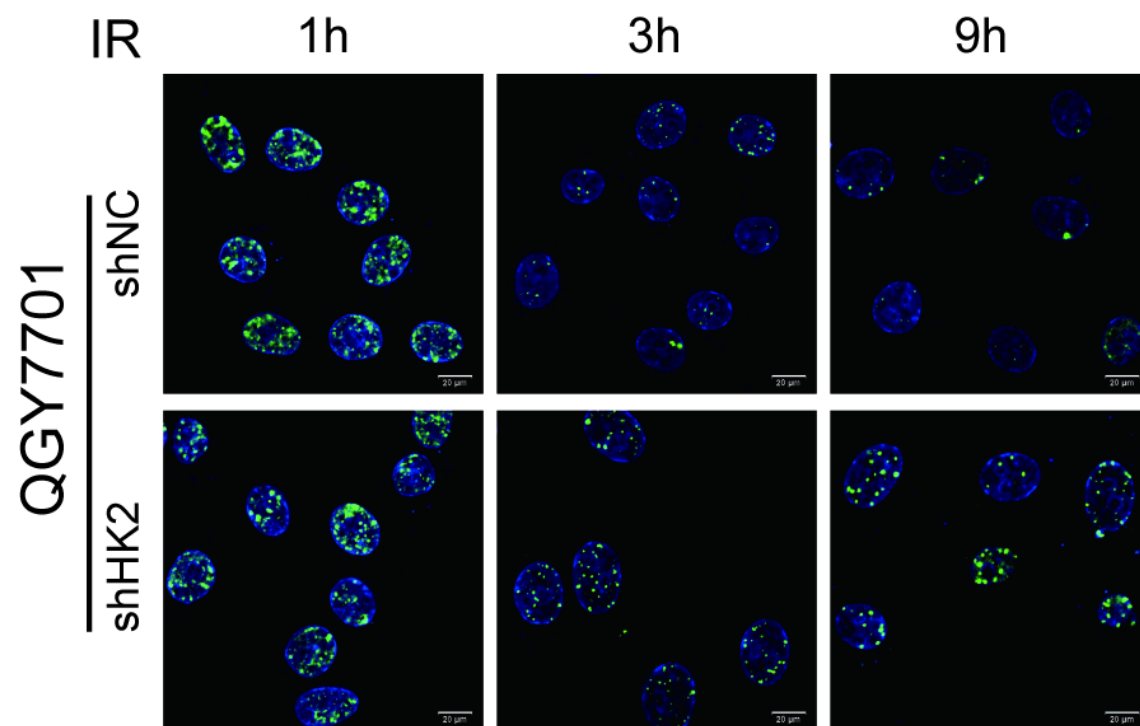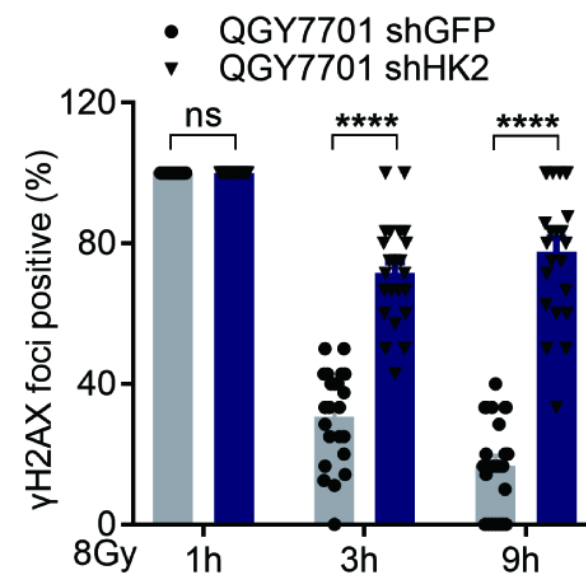

F

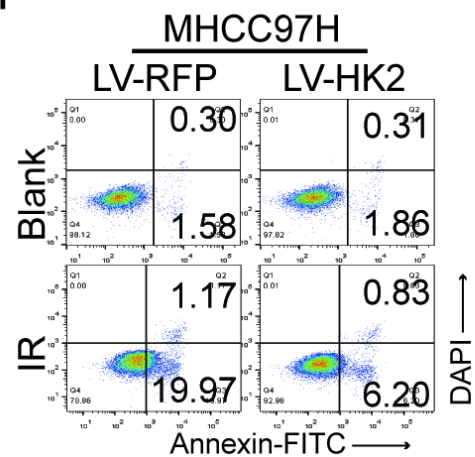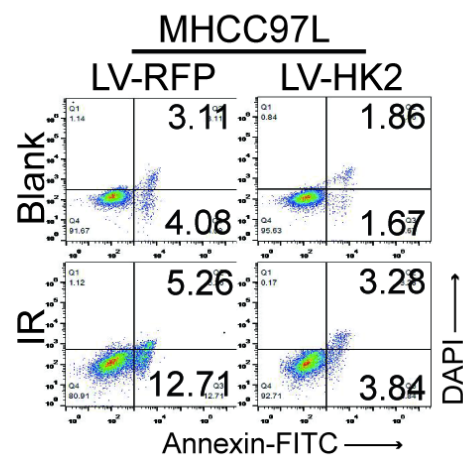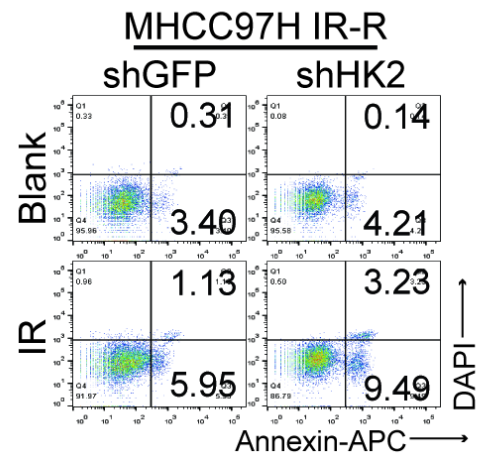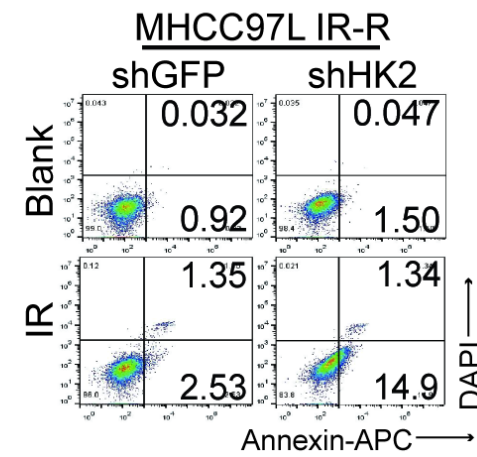

G

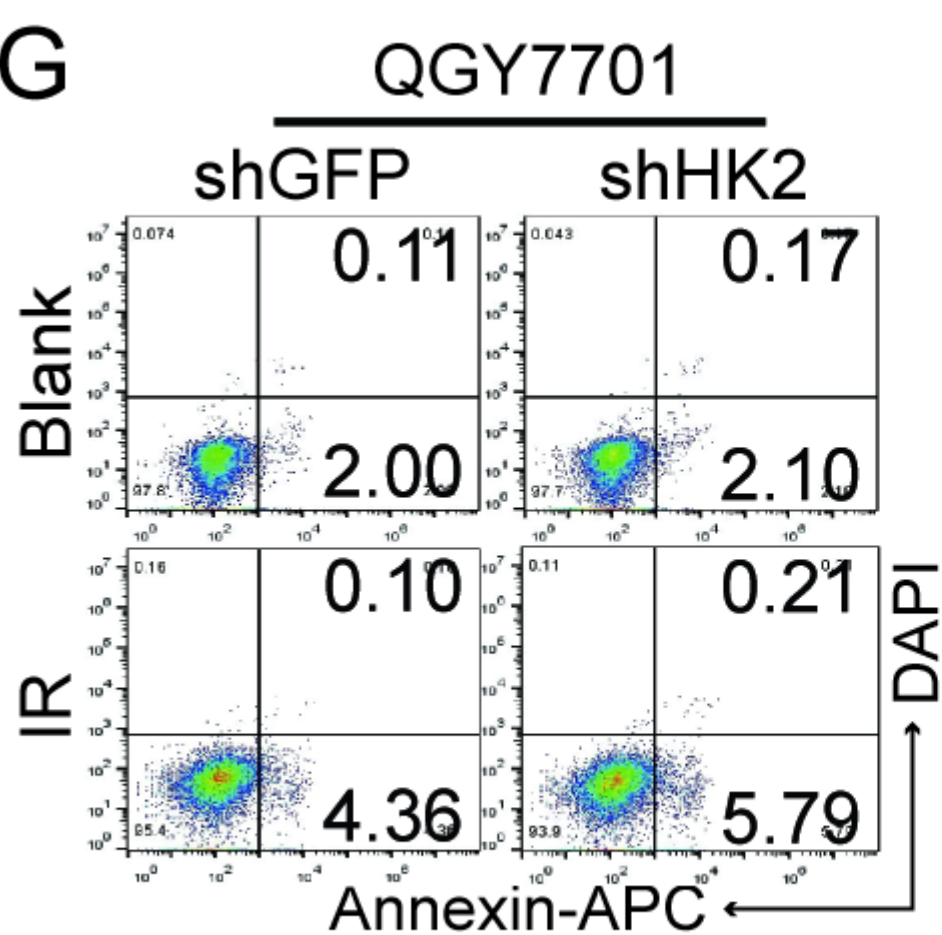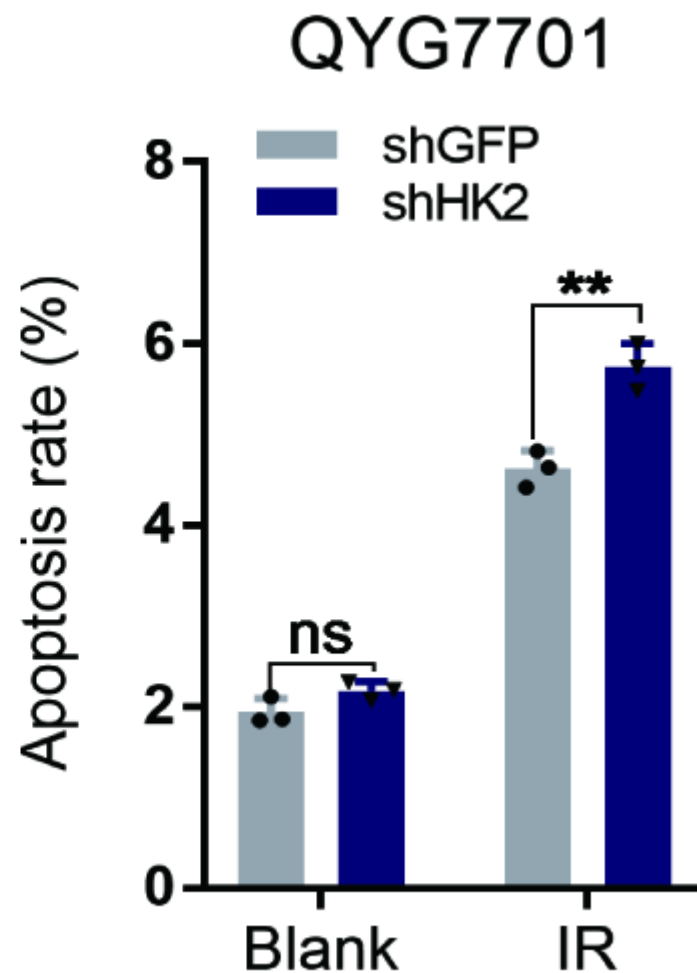

H

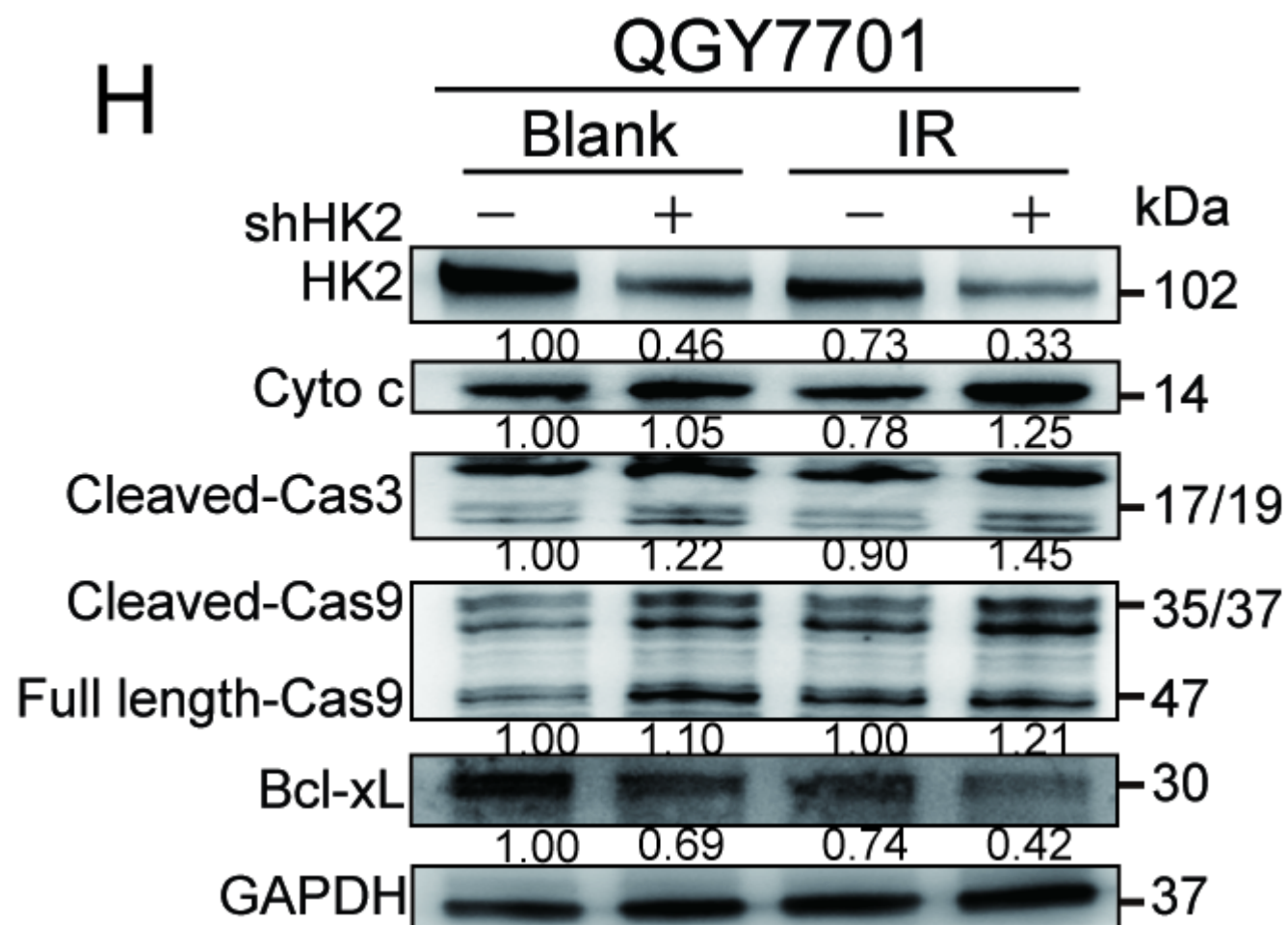

I

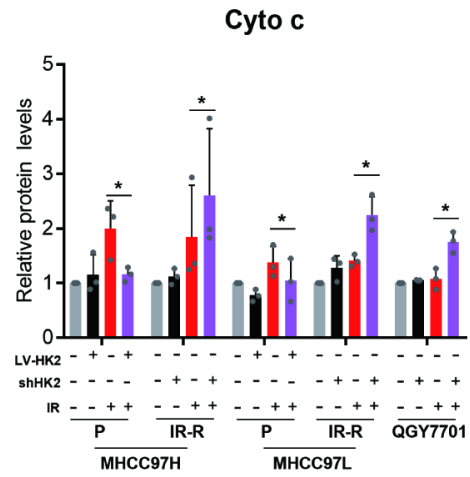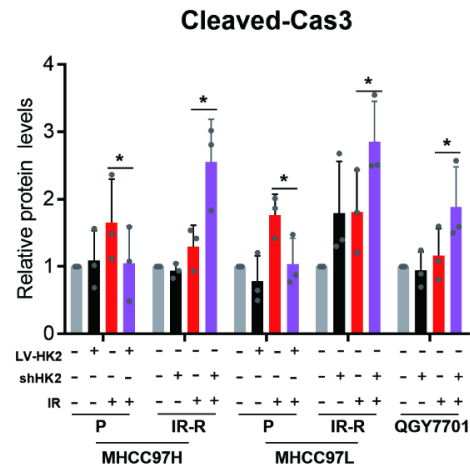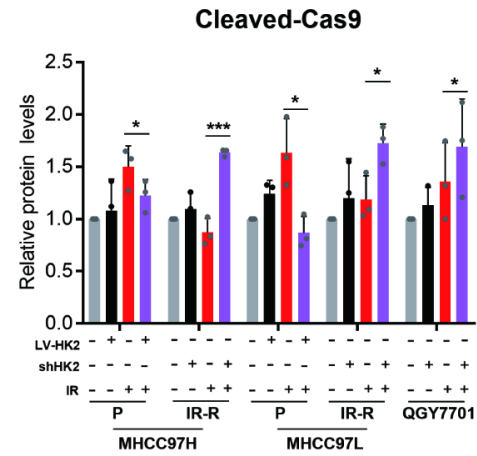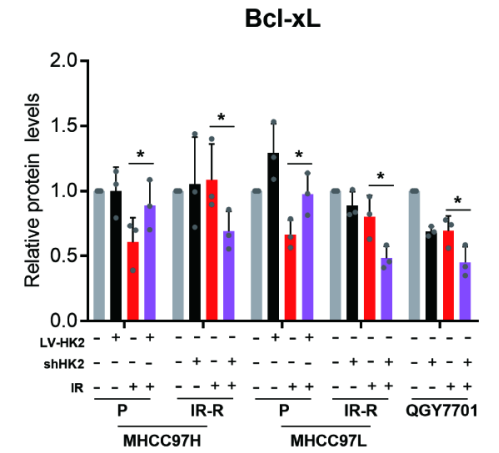

Supplement: Supplementary file 15 — Extended Supplementary Figure1 (Supplementary Figure1 merge file) [file 41419_2023_6009_MOESM15_ESM.pdf]

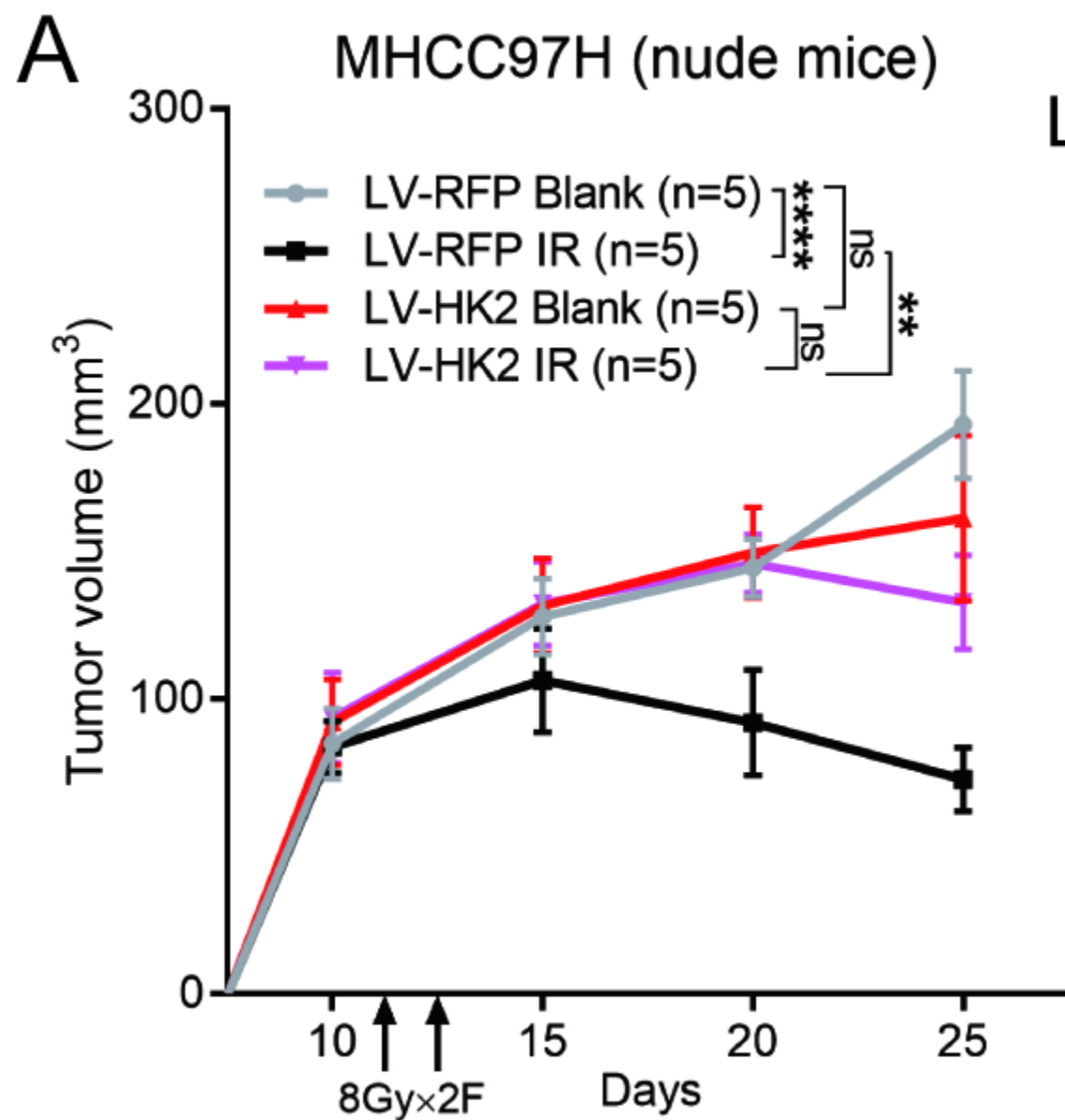

| LV-HK2 | - | - | + | + |
|--------|---|---|---|---|
| IR     | - | + | - | + |

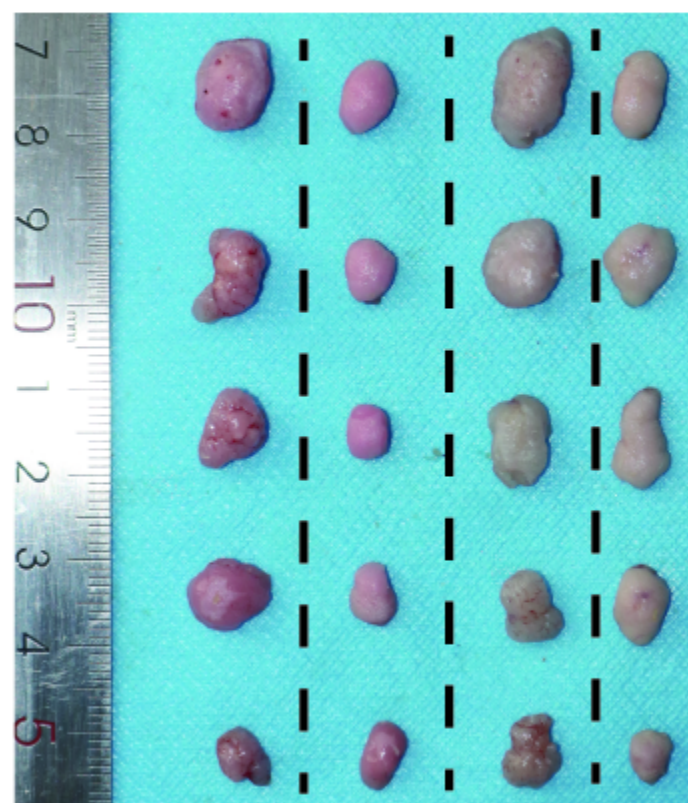

B

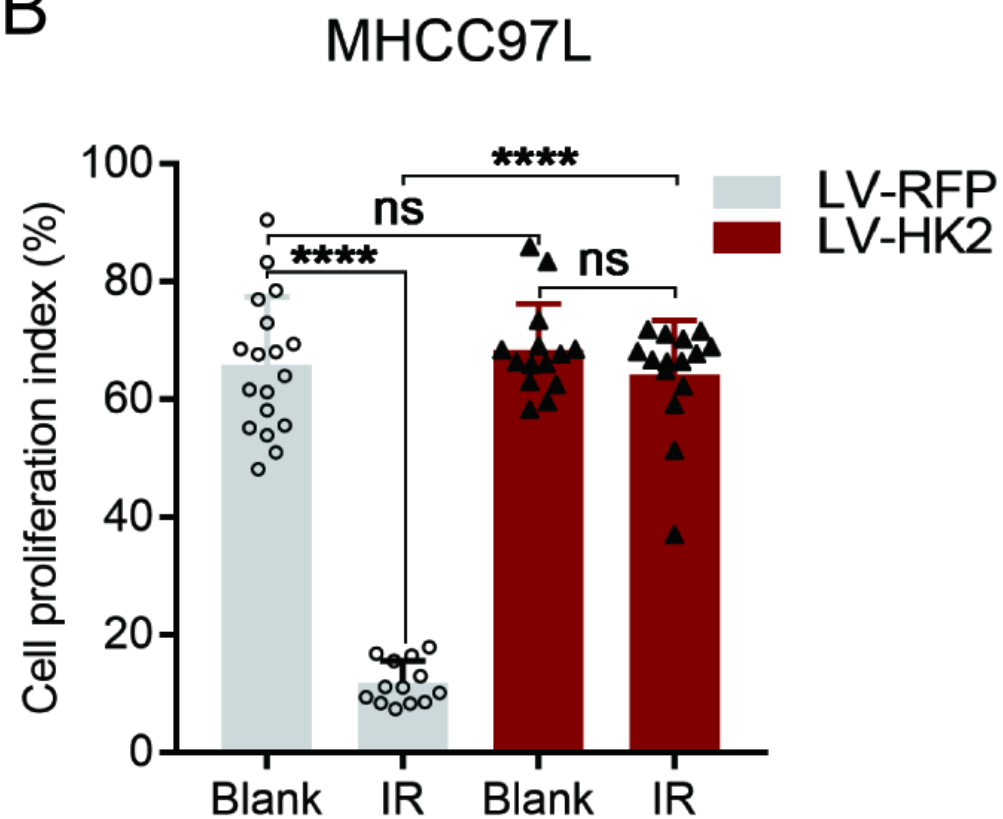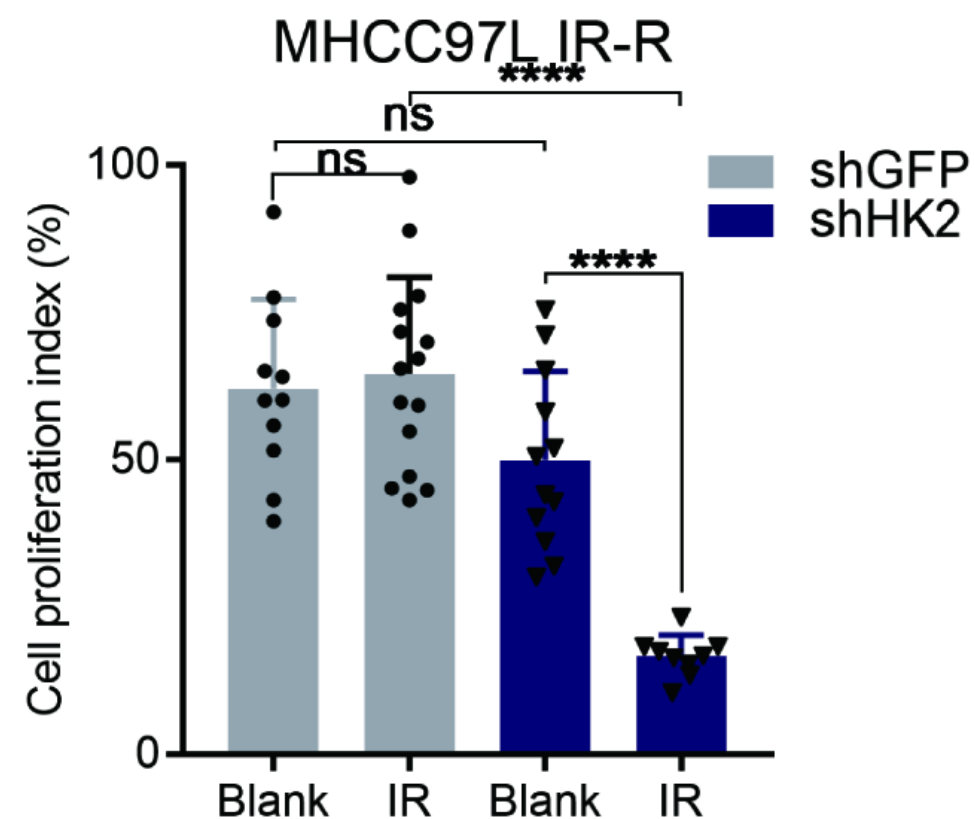

C

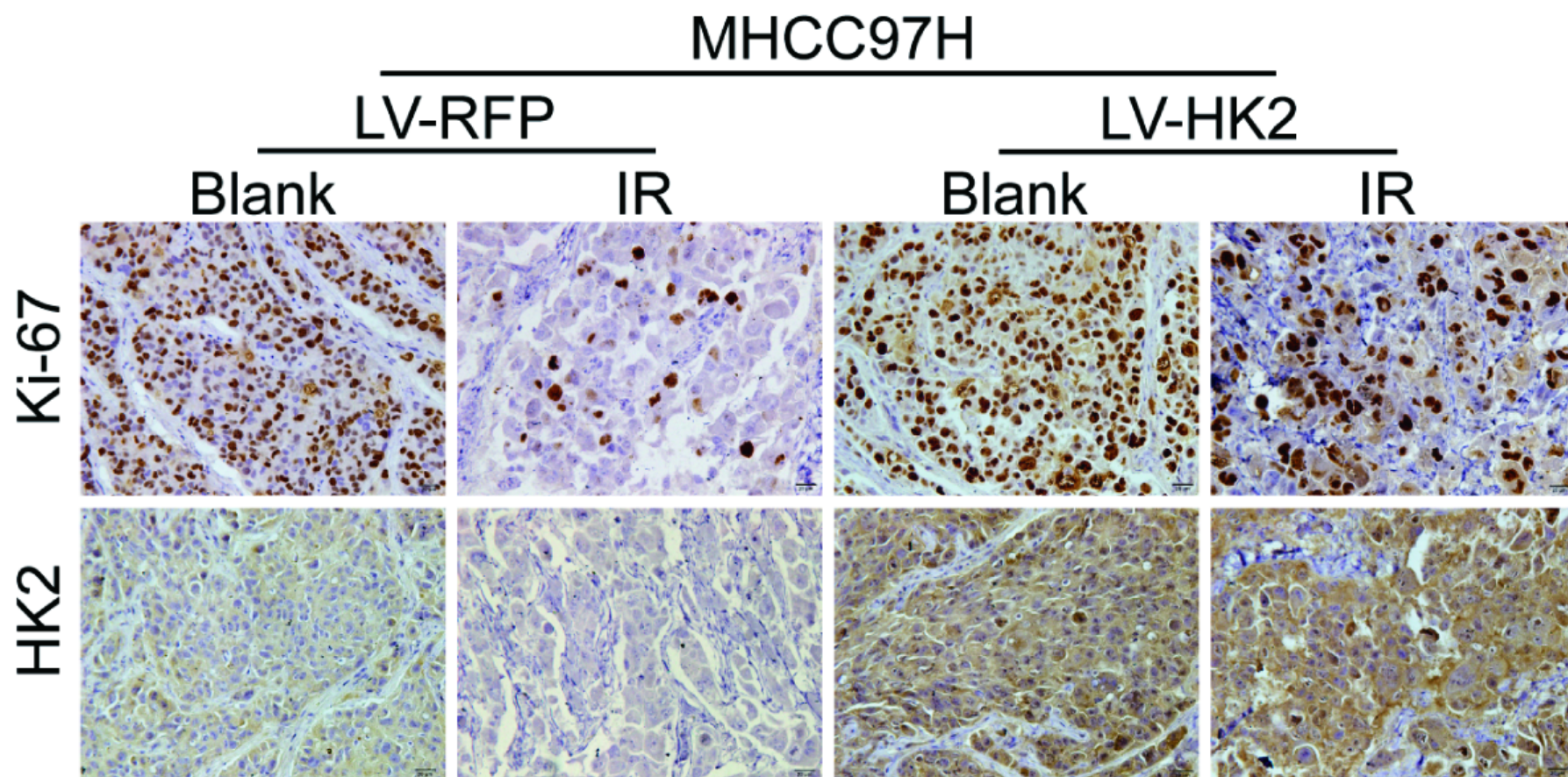

D

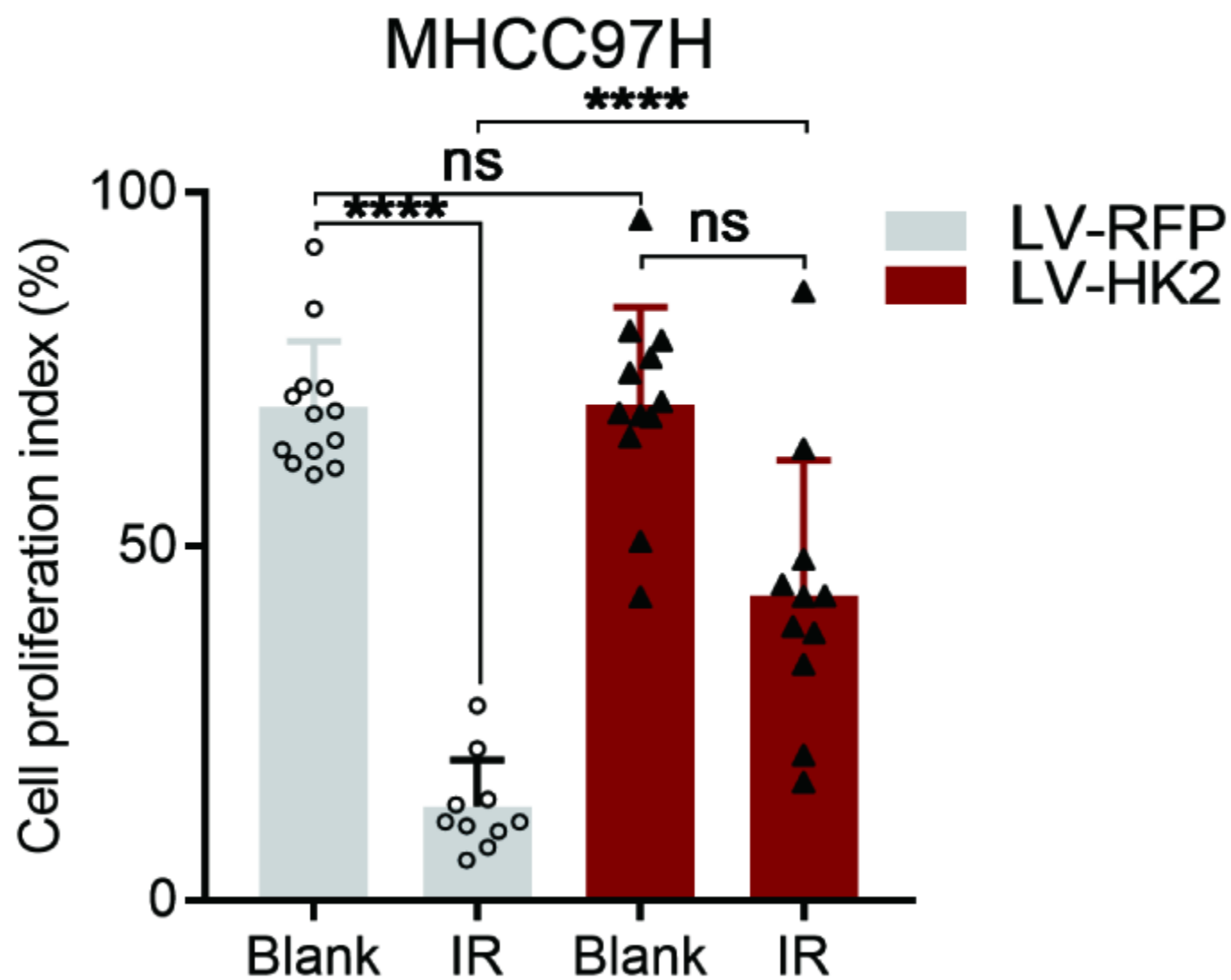

E

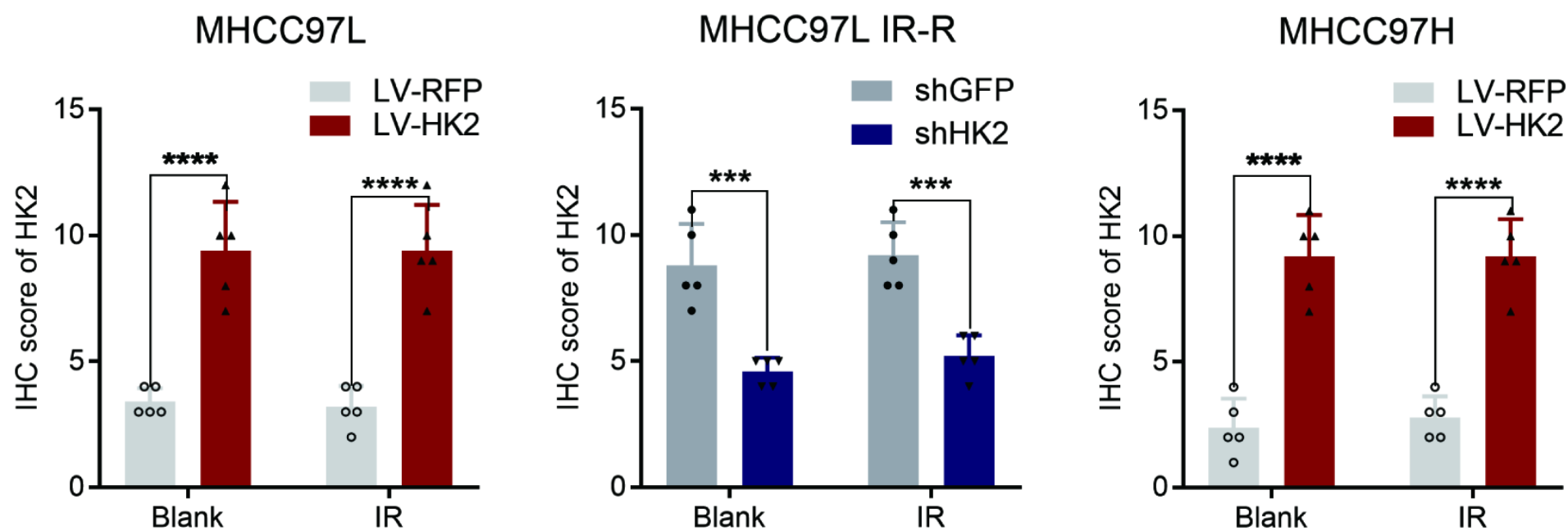

F

H22 cells (nude mice)

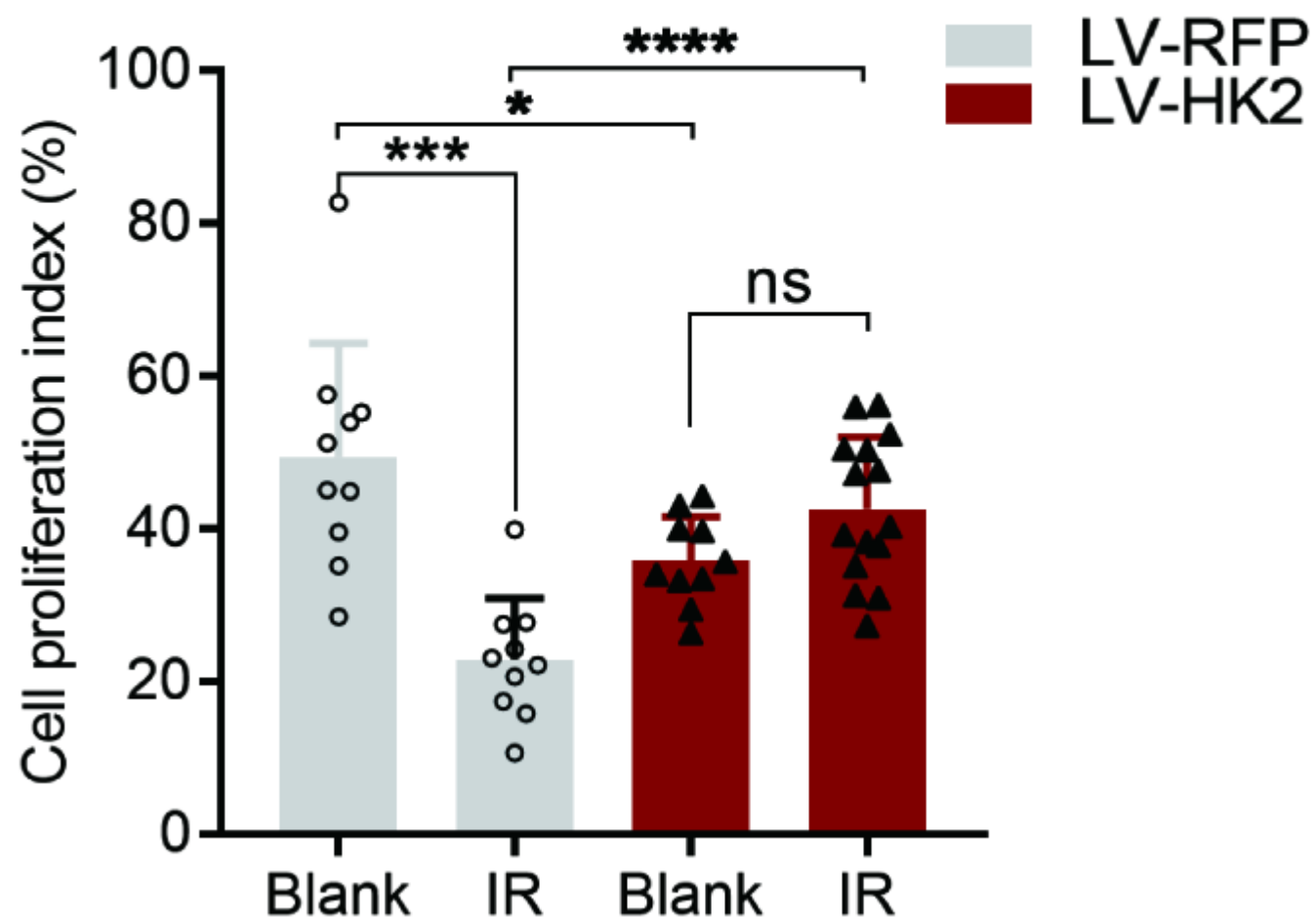

G

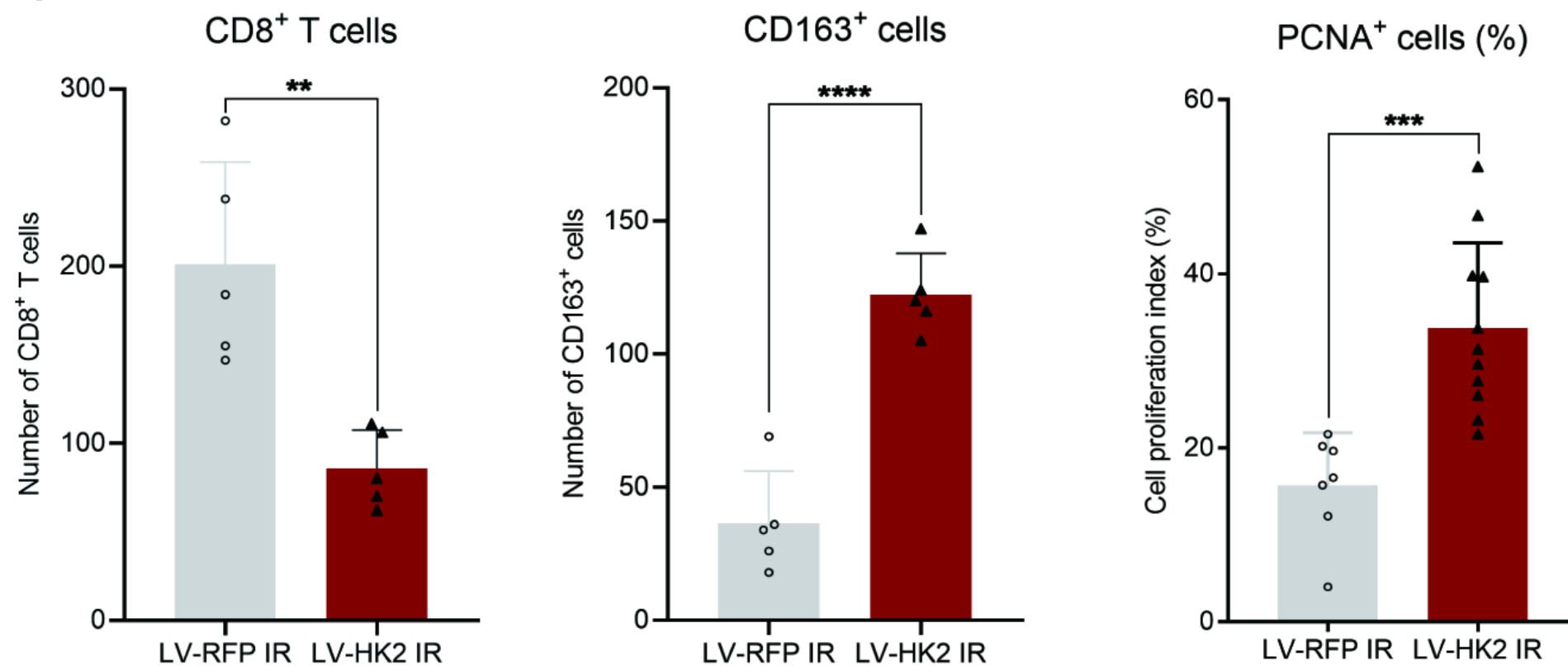

Supplement: Supplementary file 16 — Extended Supplementary Figure2 (Supplementary Figure2 merge file) [file 41419_2023_6009_MOESM16_ESM.pdf]

A

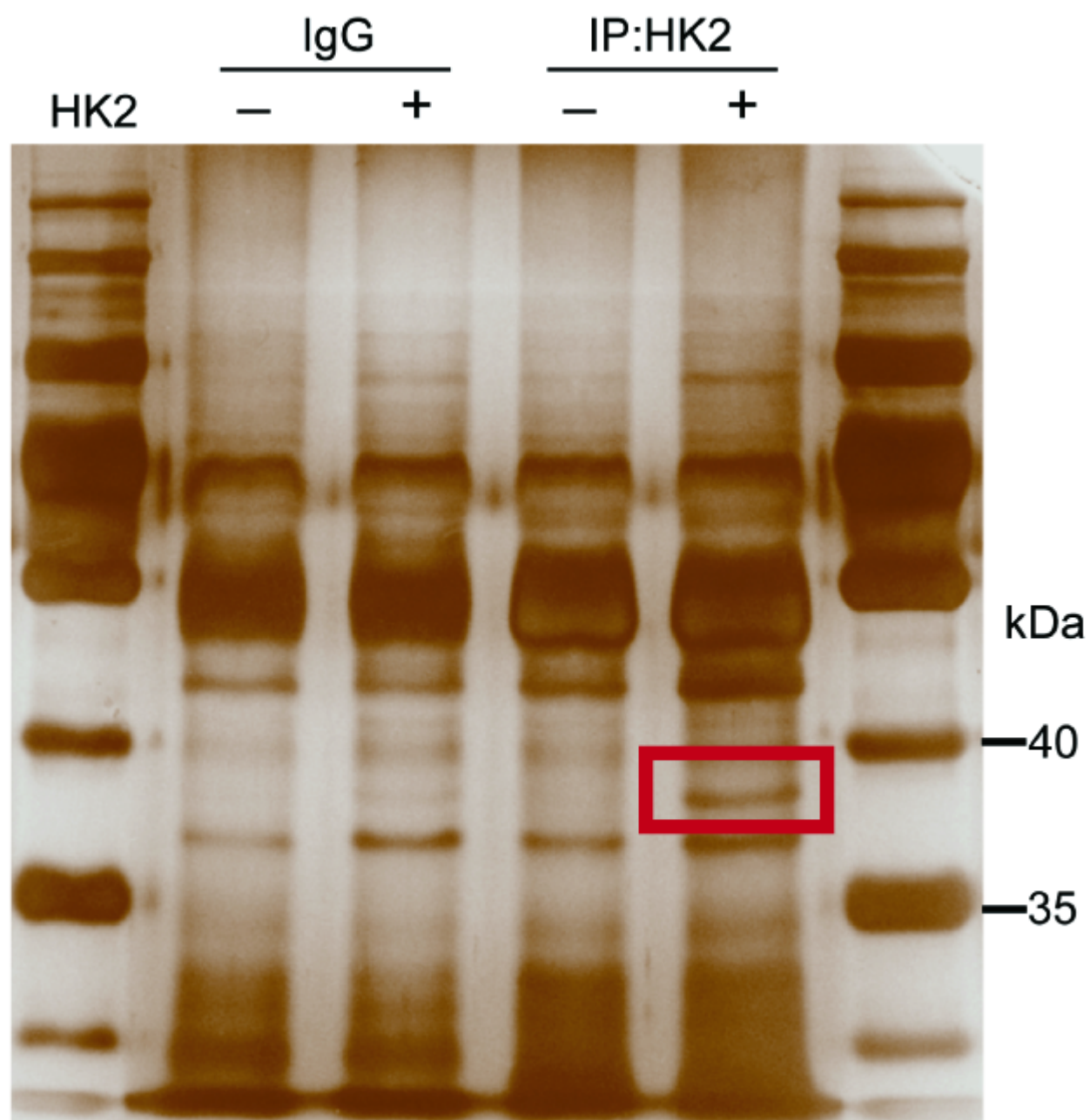

# B

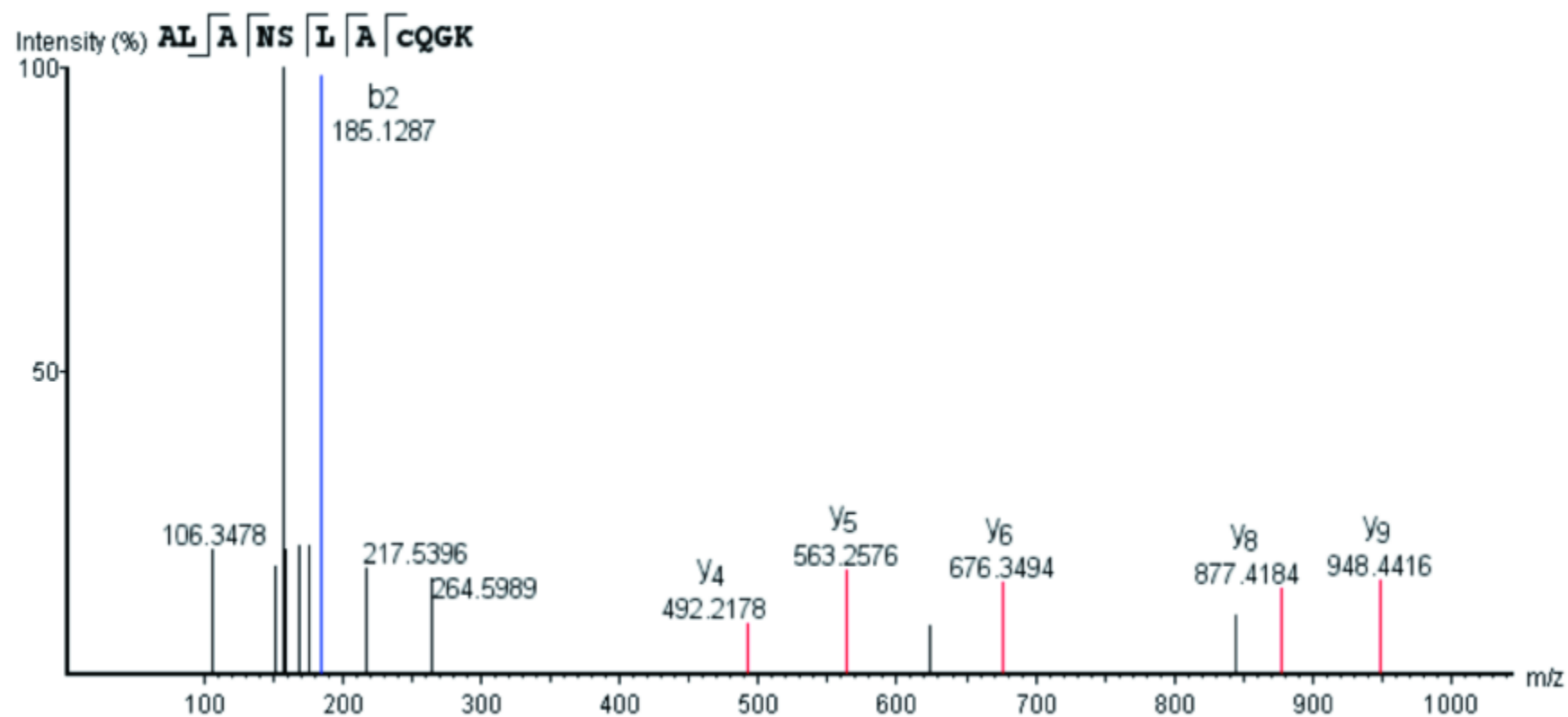

C

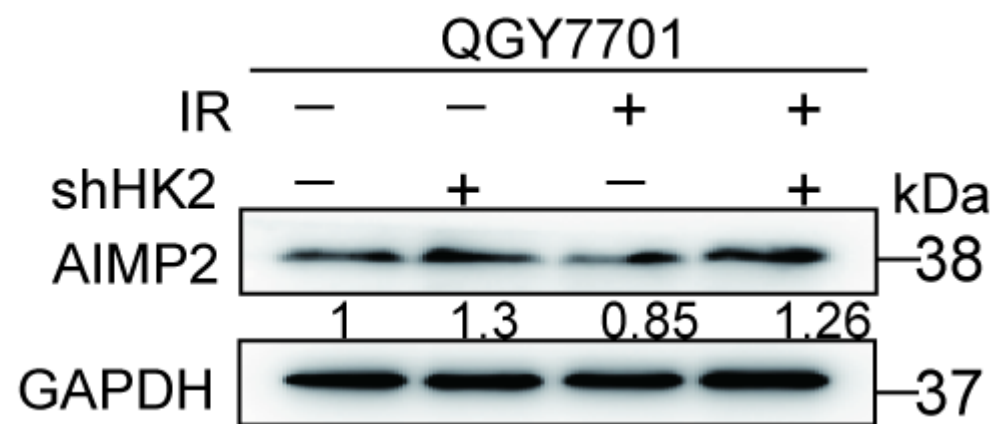

D

# AIMP2

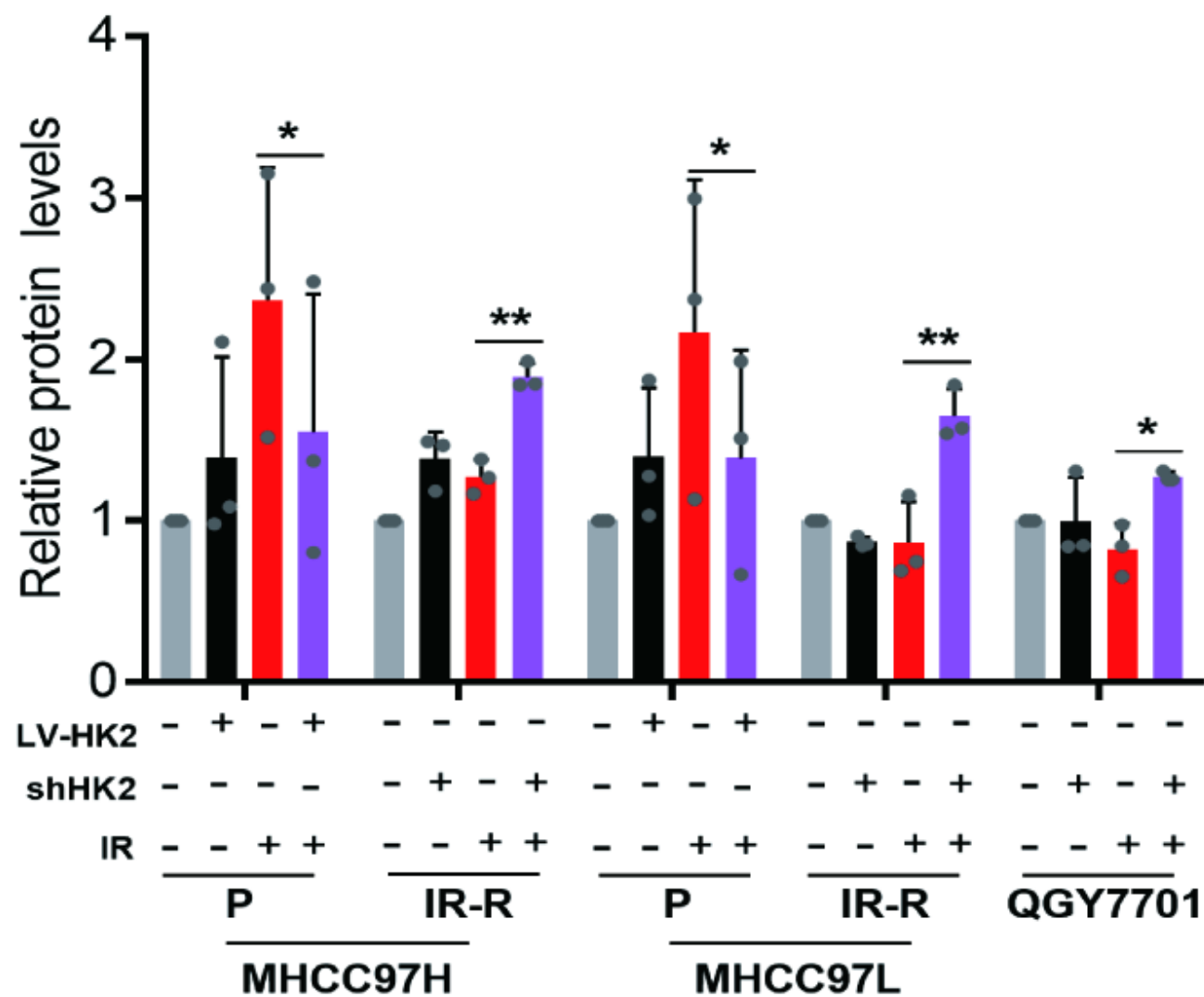

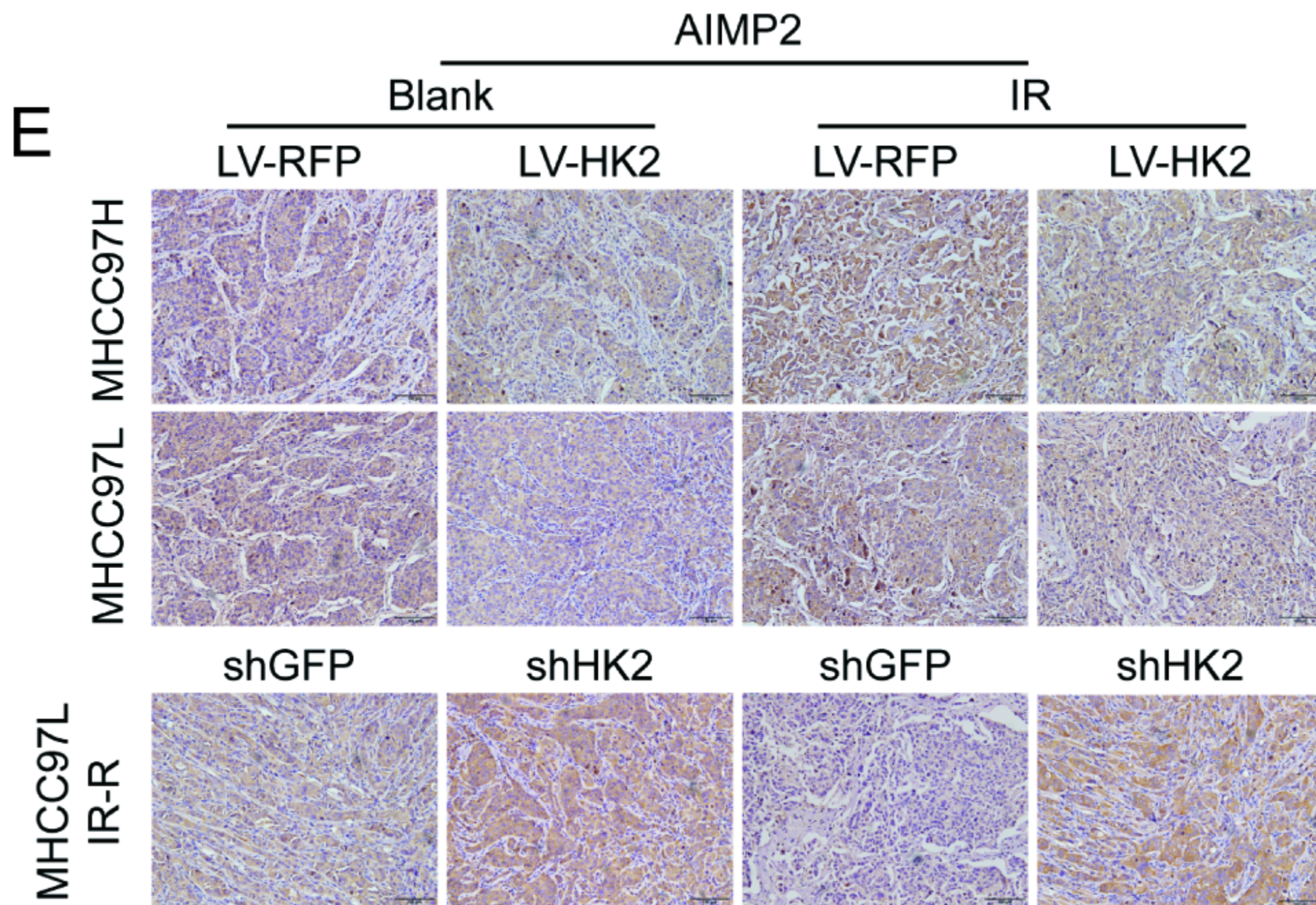

F

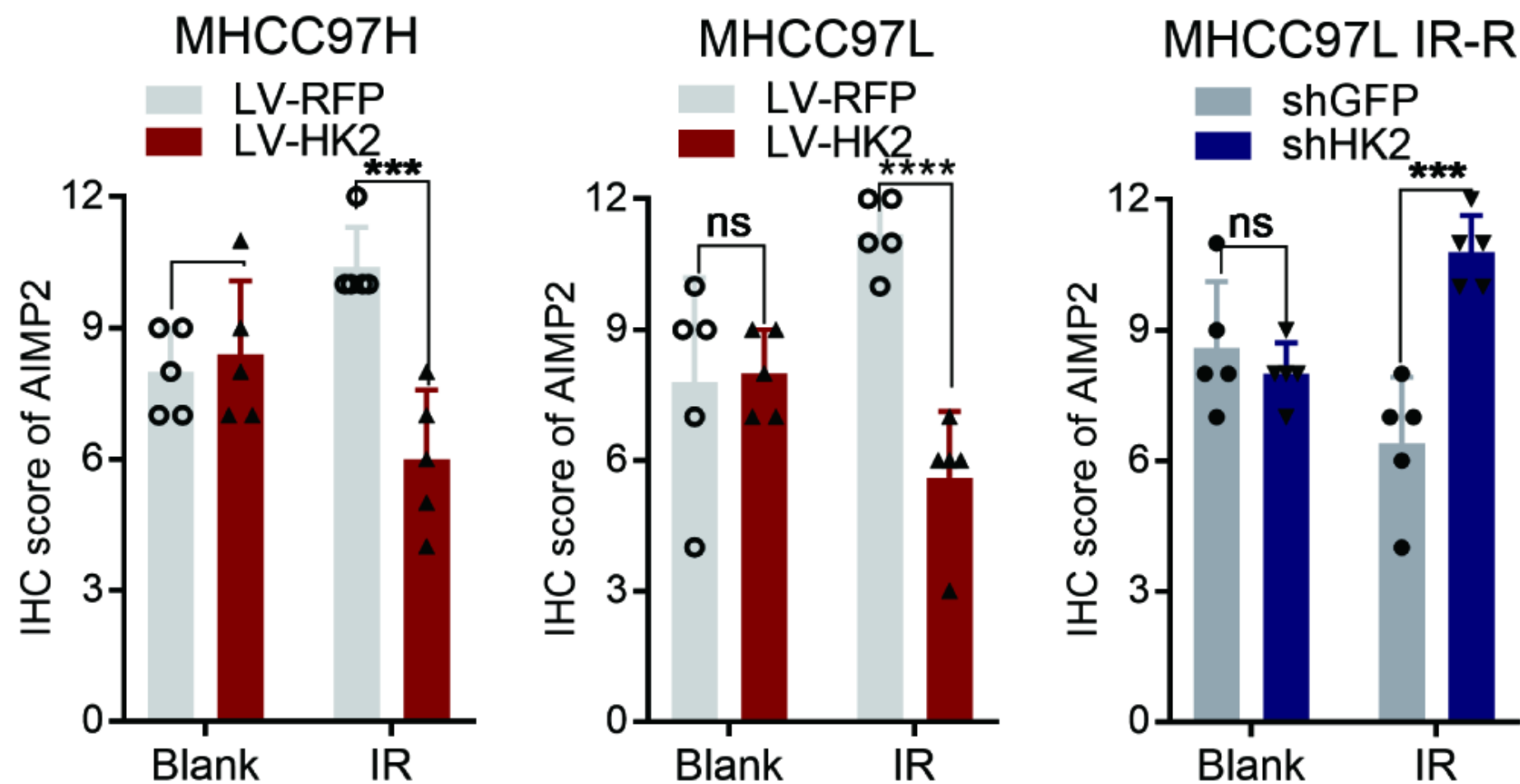

G

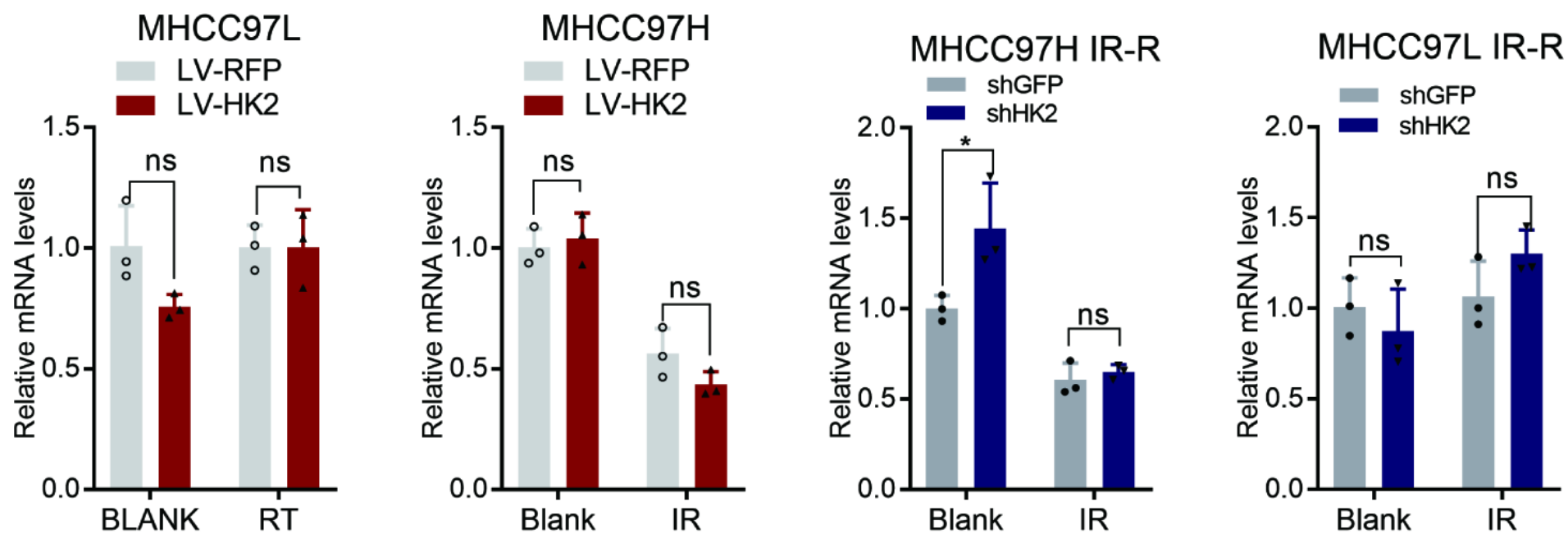

H

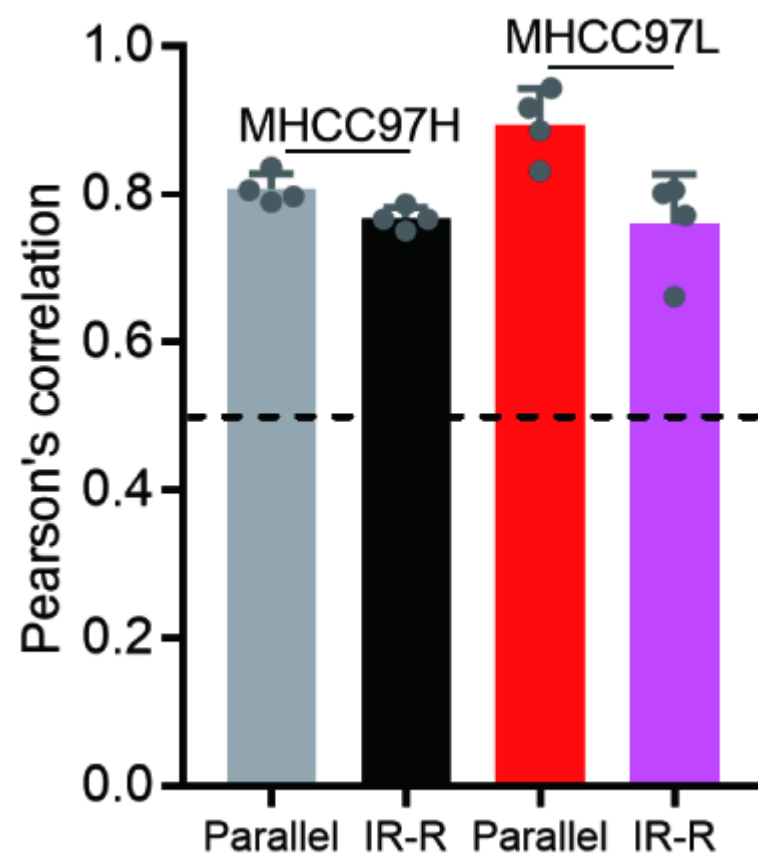

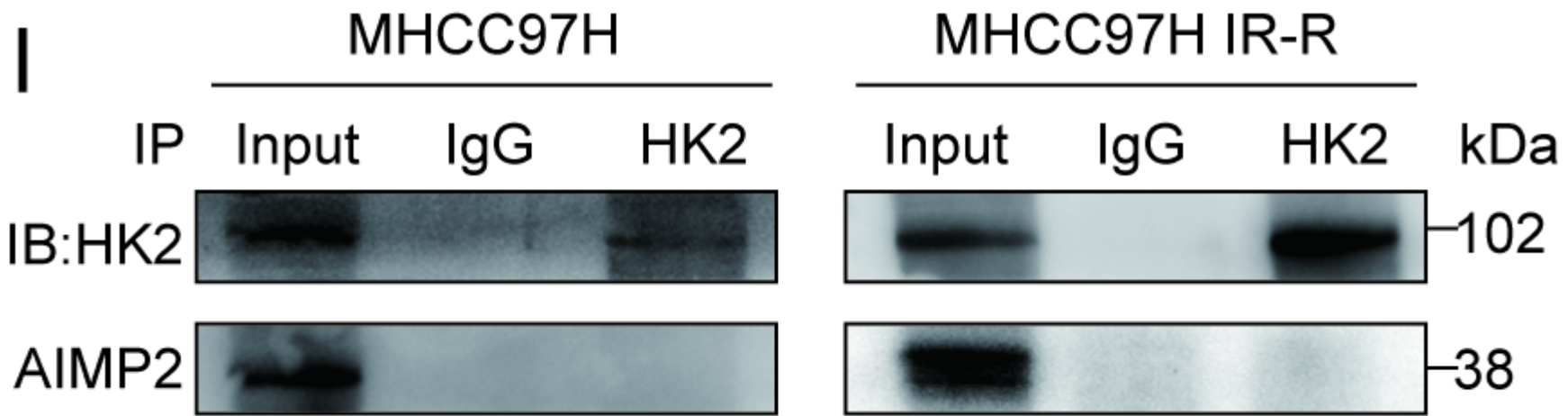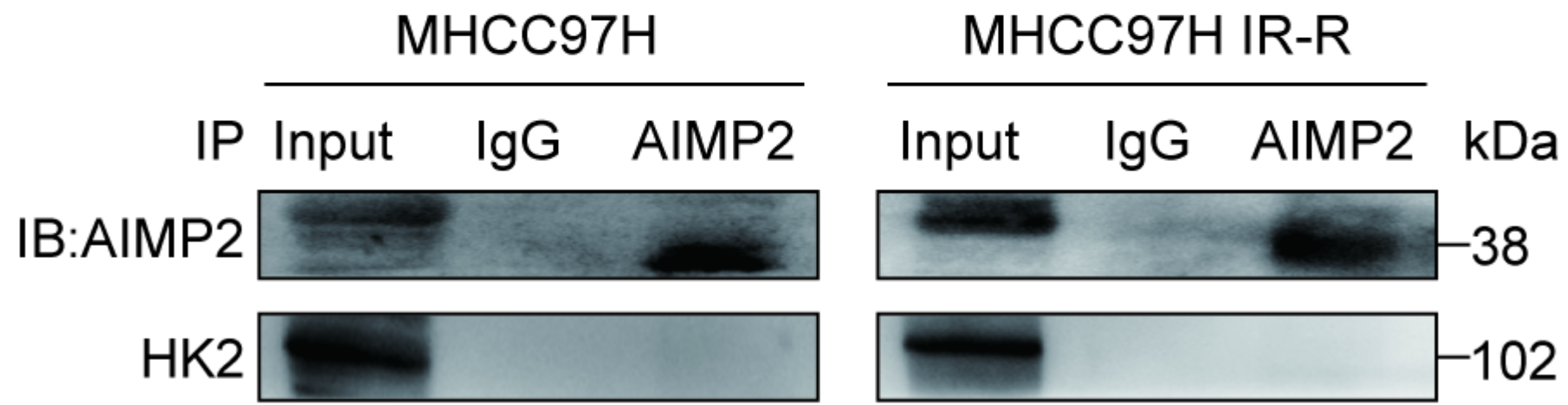

J

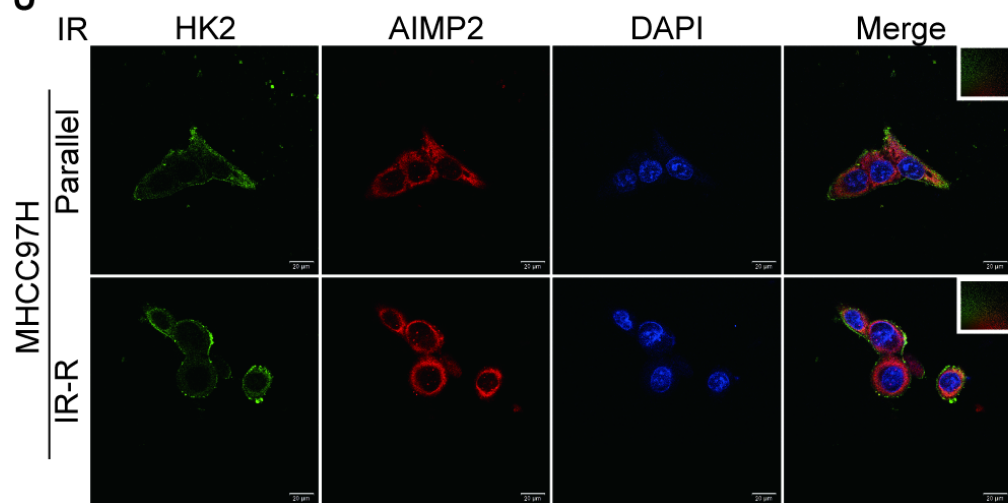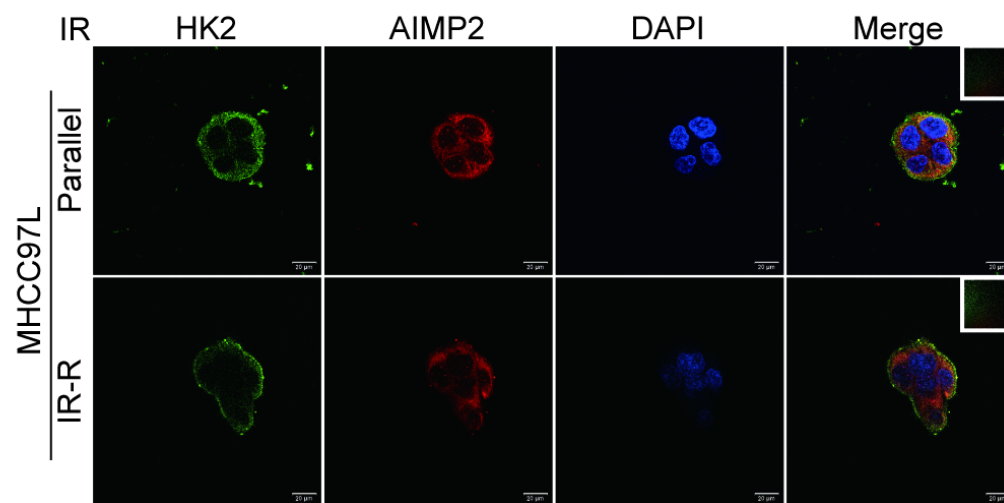

K

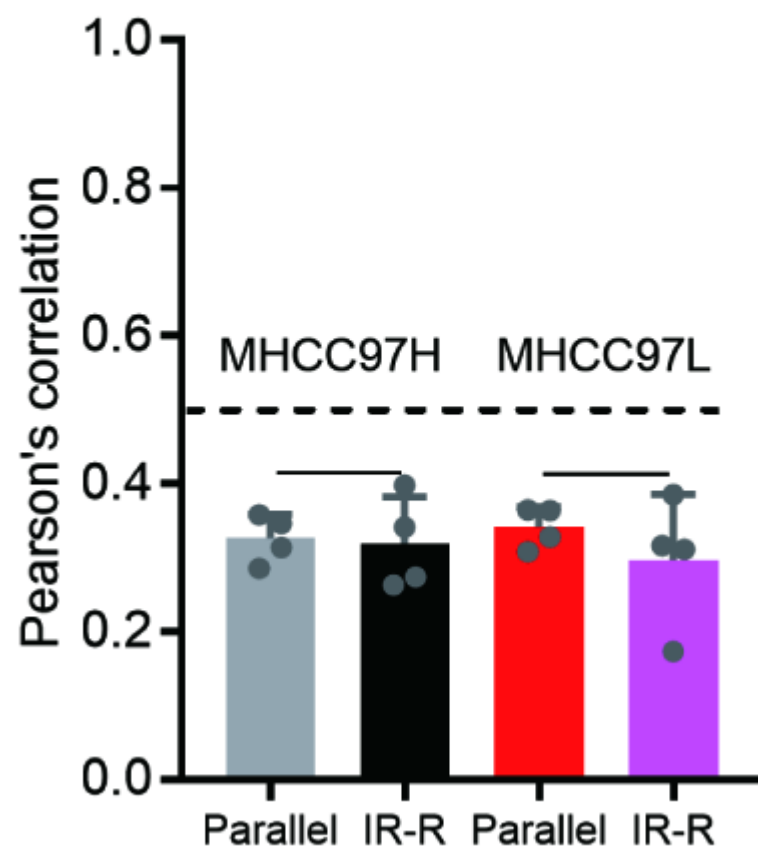

Supplement: Supplementary file 17 — Extended Supplementary Figure3 (Supplementary Figure3 merge file) [file 41419_2023_6009_MOESM17_ESM.pdf]

A

## AIMP2 (IR)

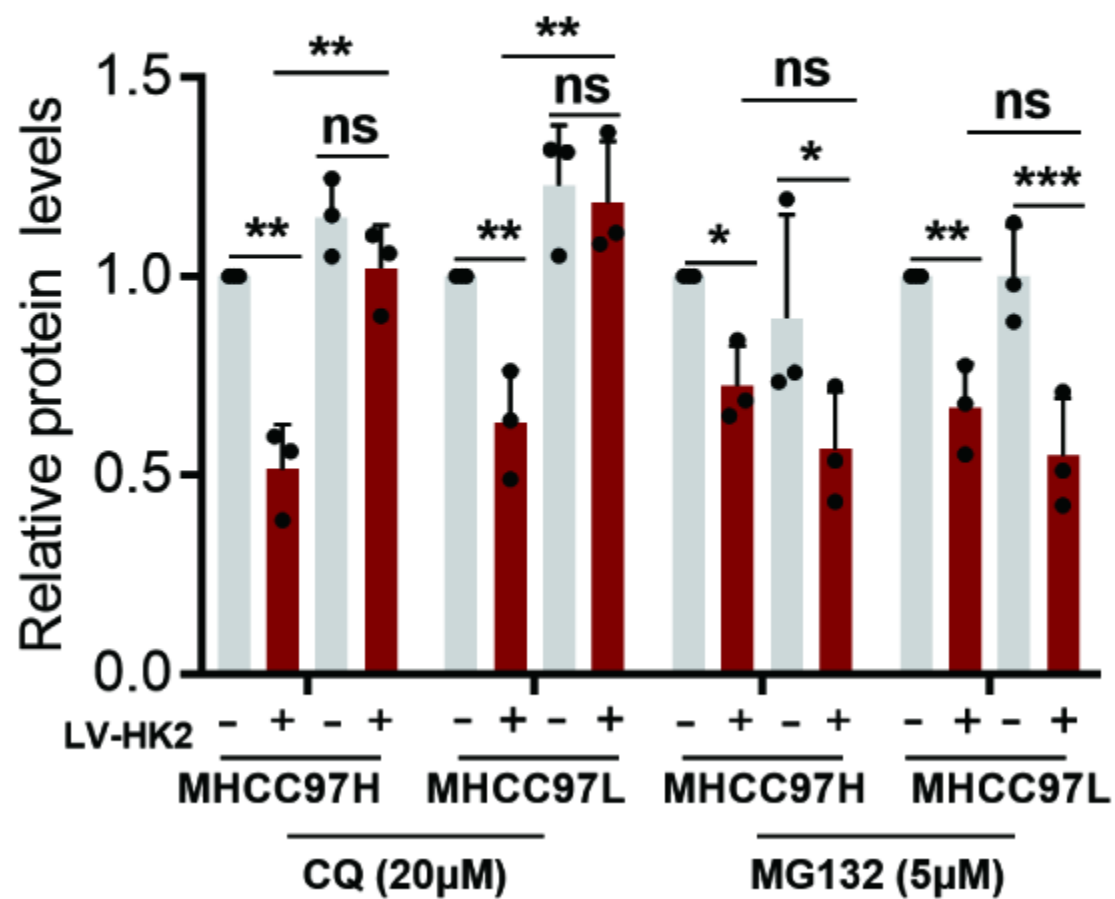

B

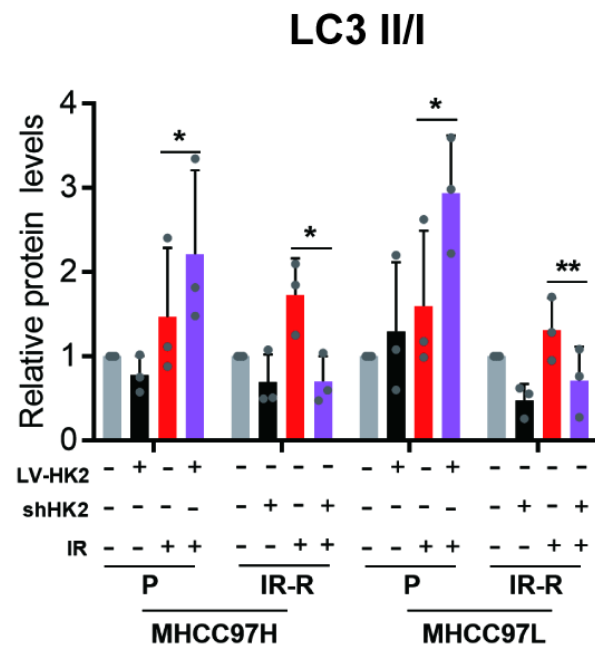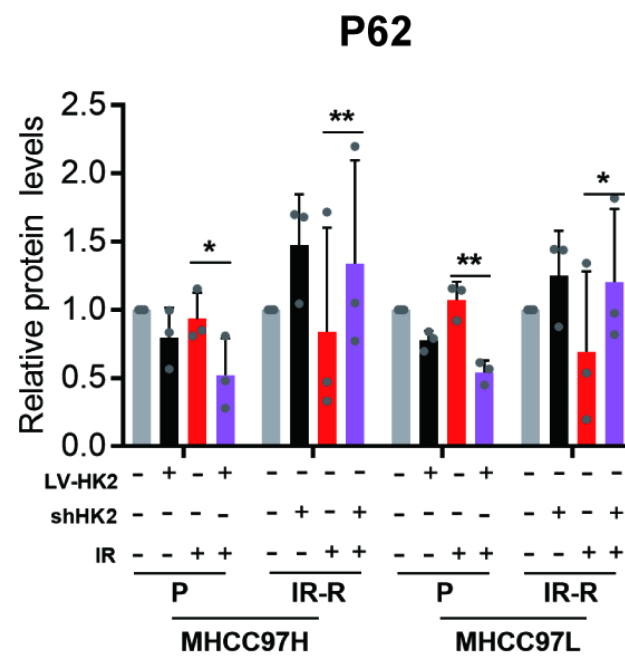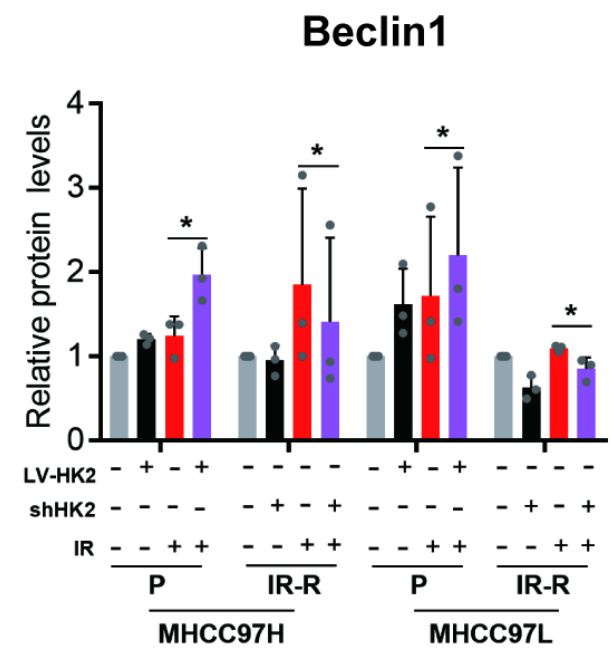

C

MHCC97L

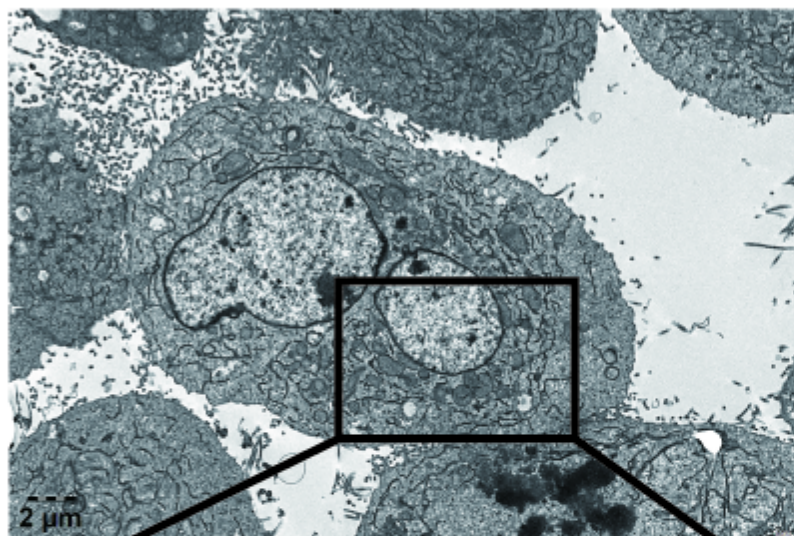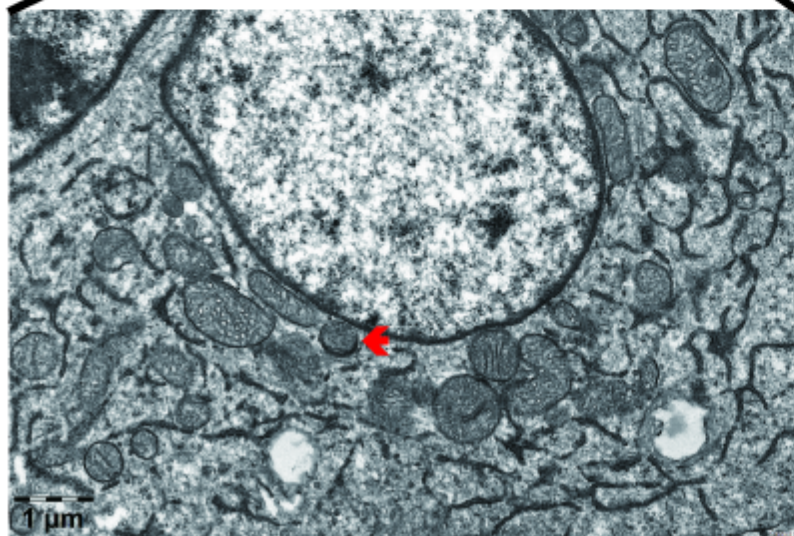

MHCC97L IR-R

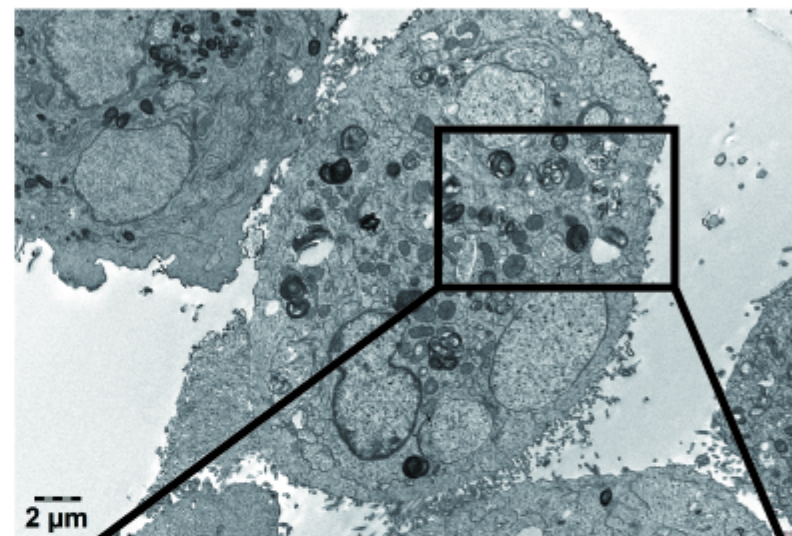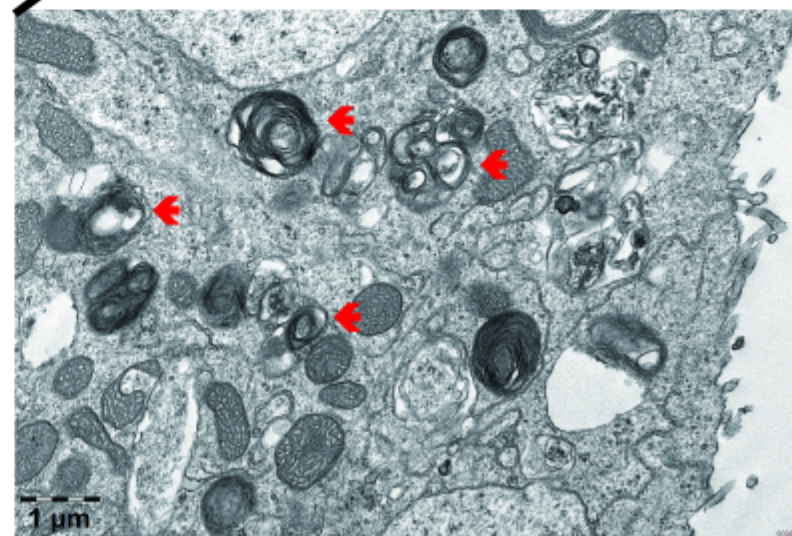

MHCC97L

D

Parallel

IR-R

mCherry

GFP

DAPI

Merge

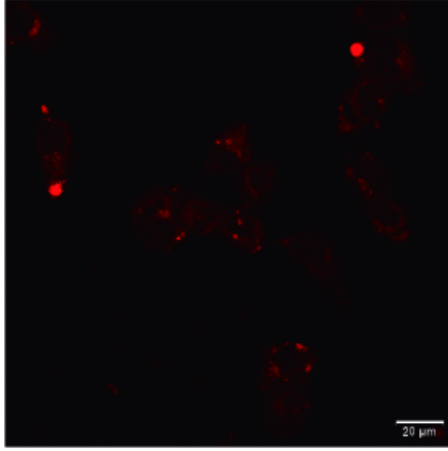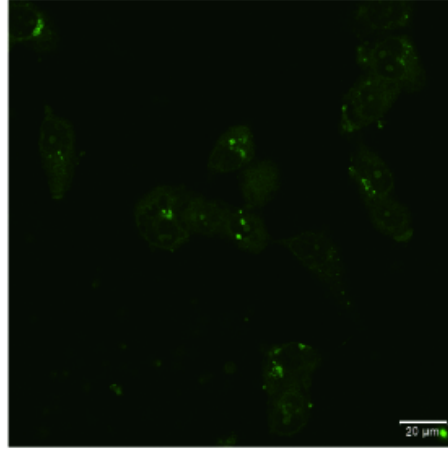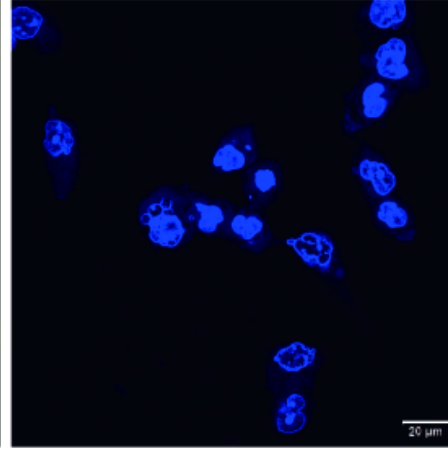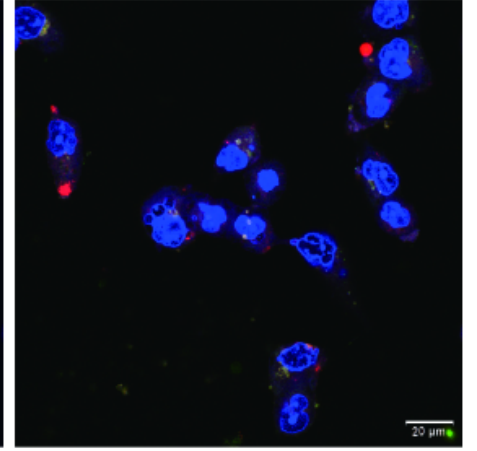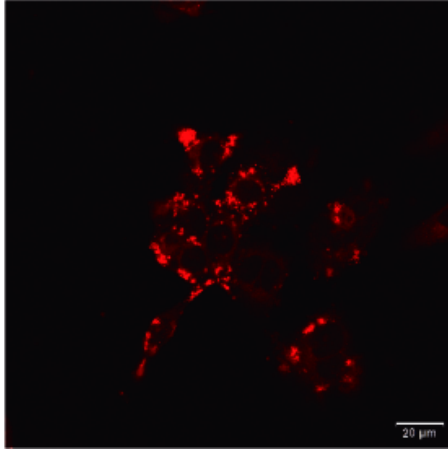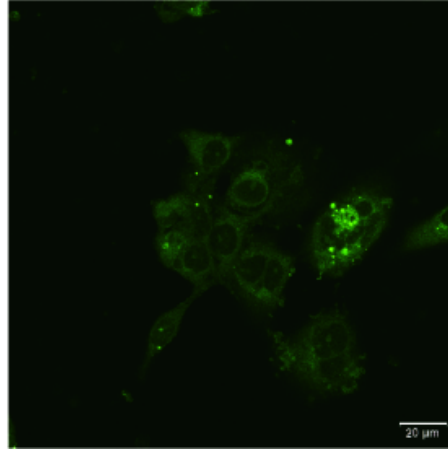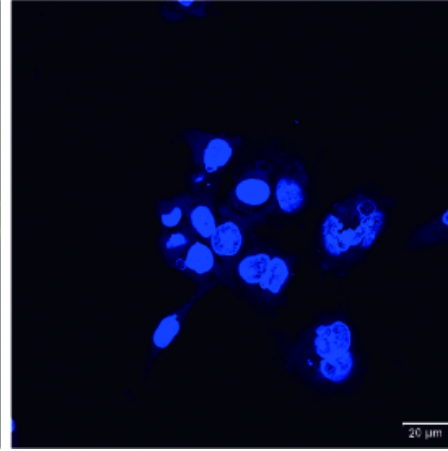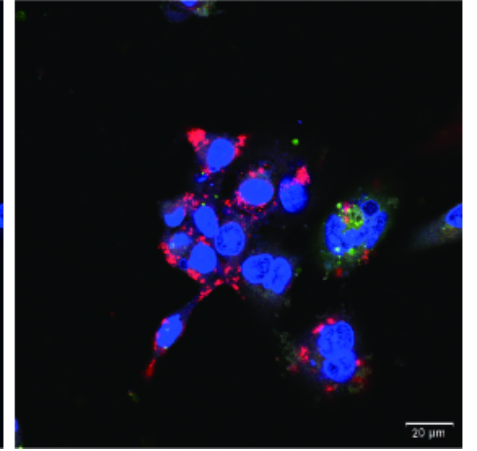

E

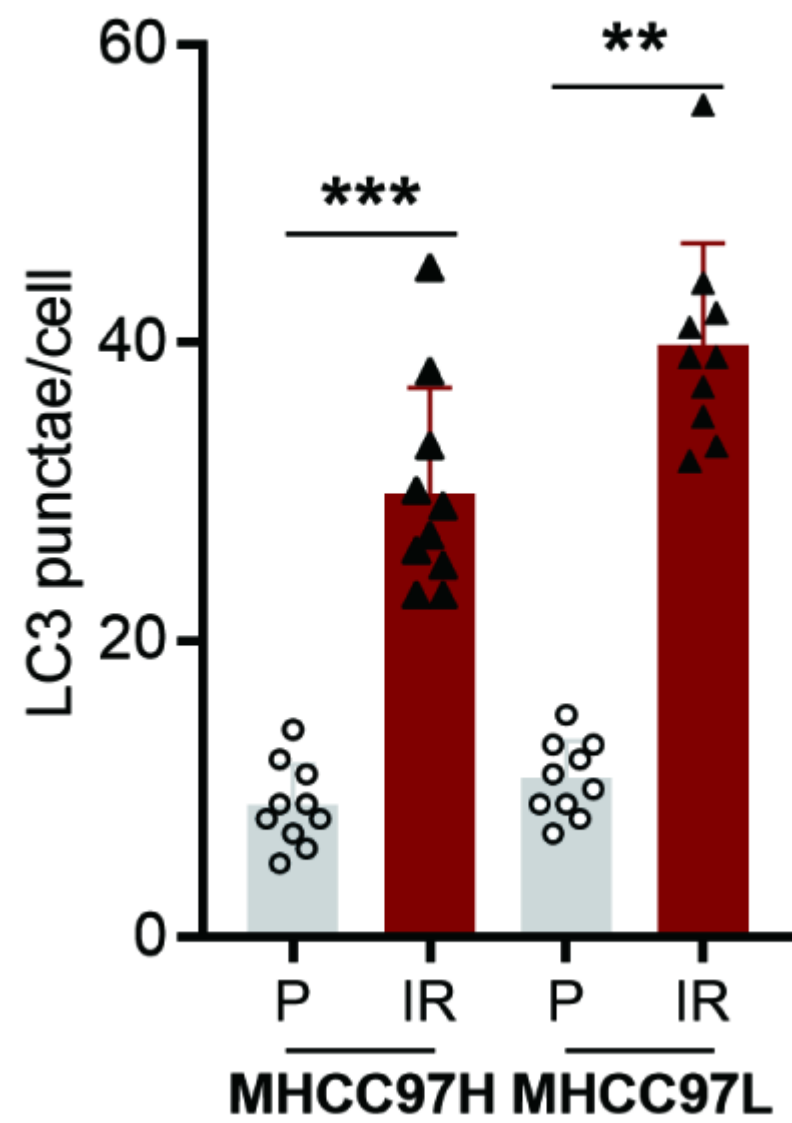

F

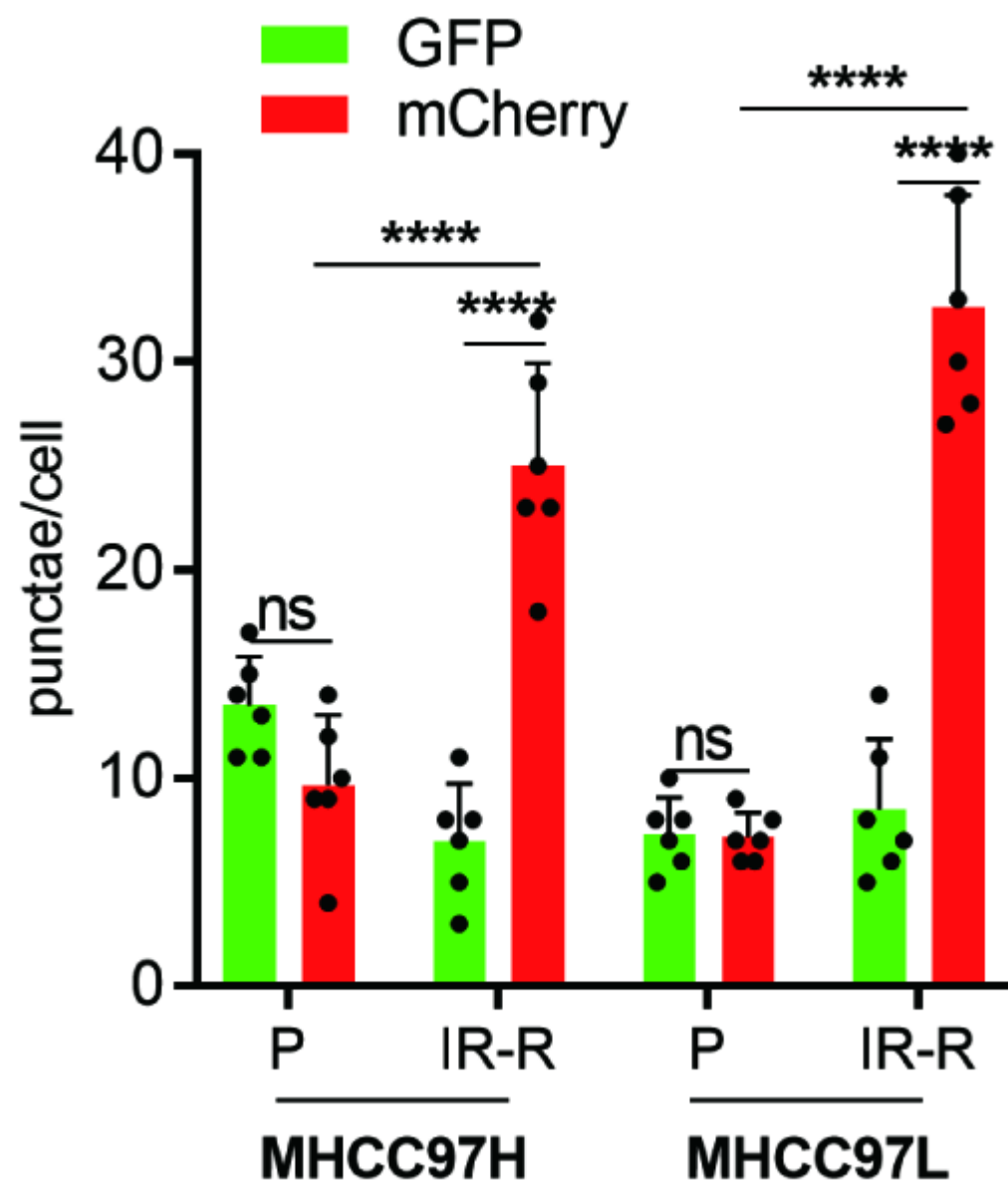

Supplement: Supplementary file 18 — Extended Supplementary Figure4 (Supplementary Figure4 merge file) [file 41419_2023_6009_MOESM18_ESM.pdf]

FIG1

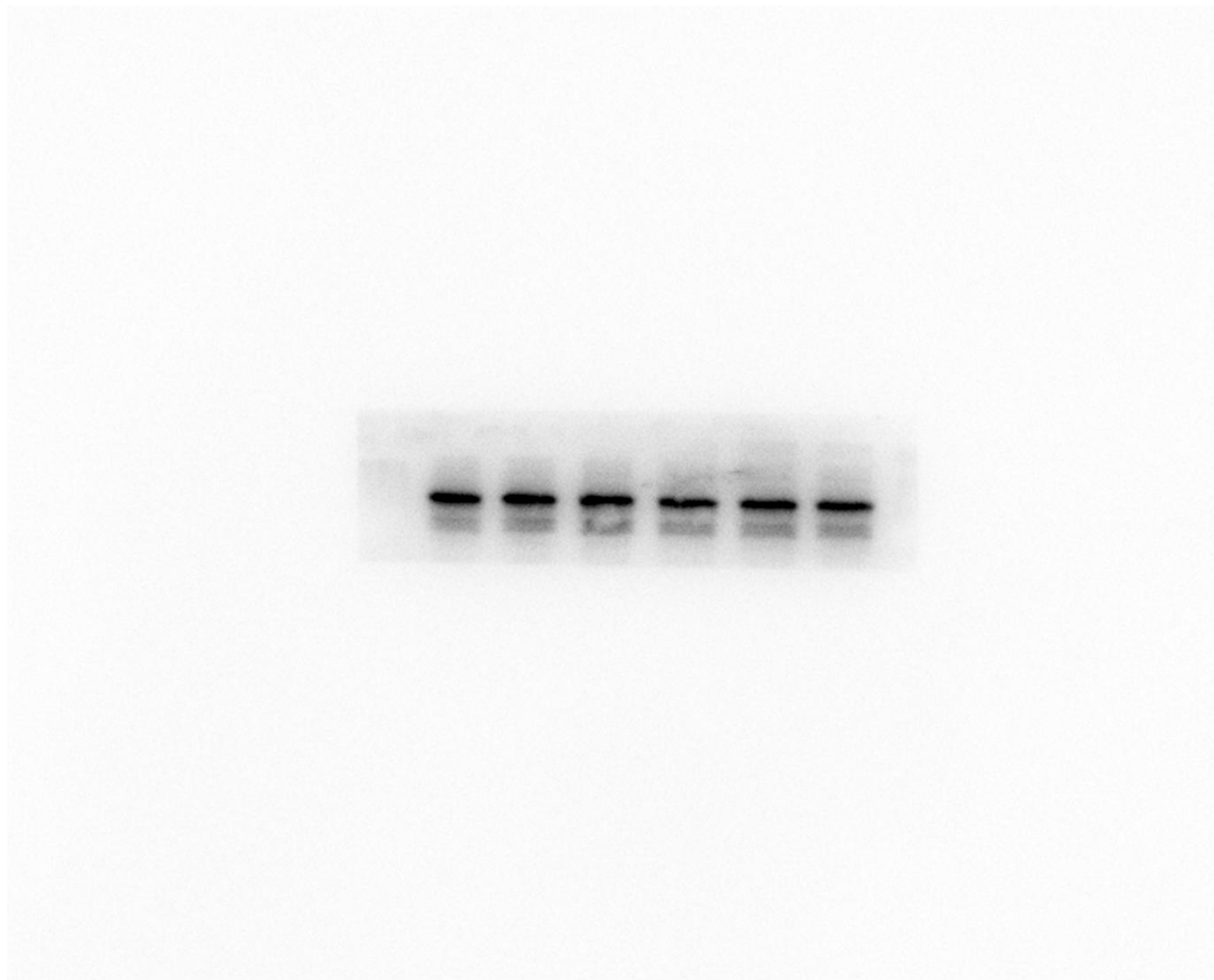

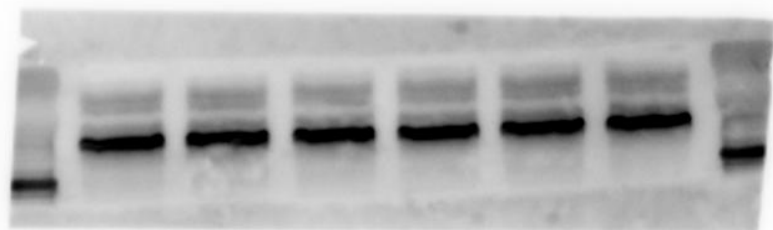

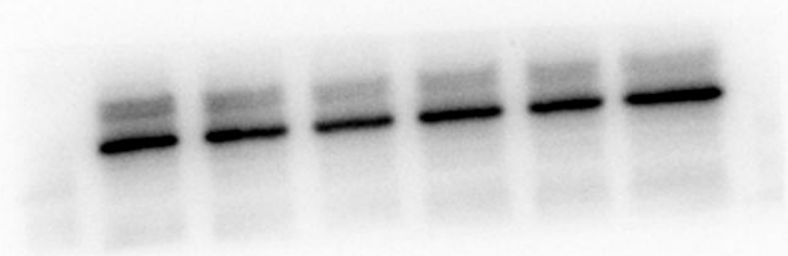

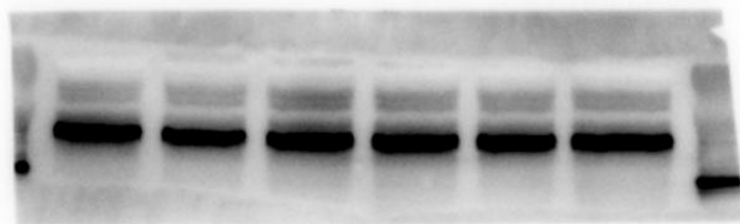

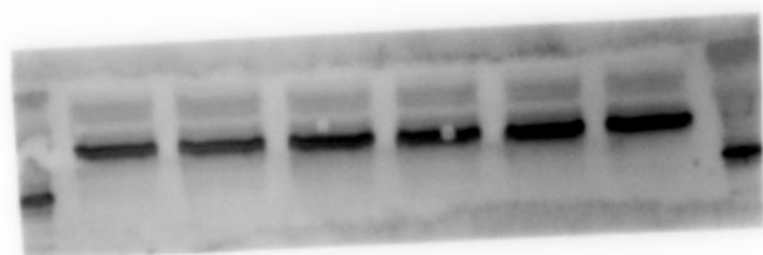

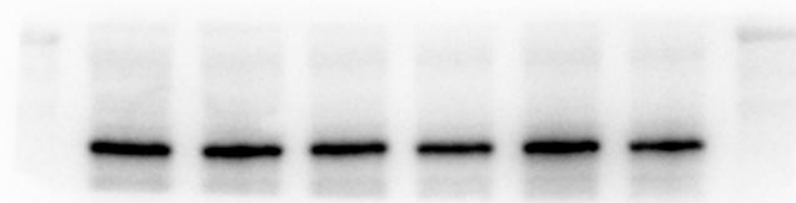

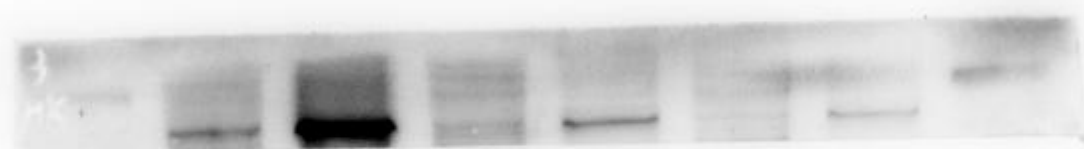

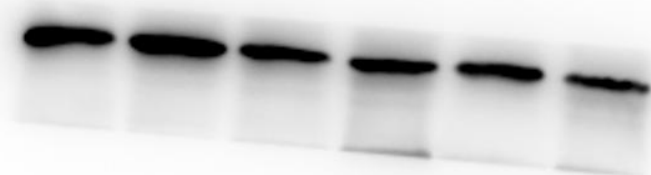

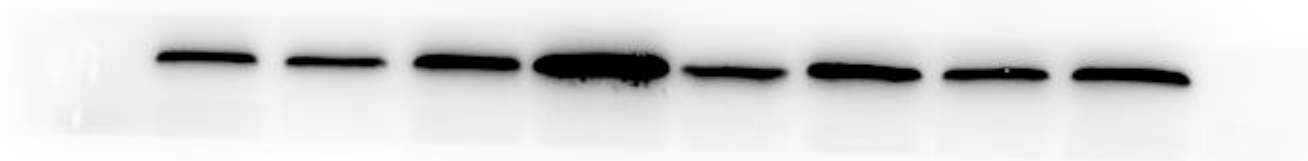

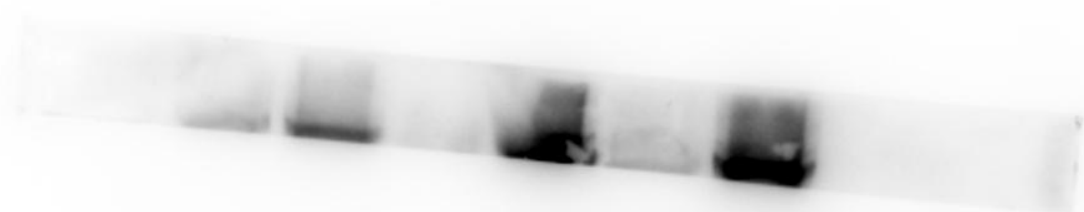

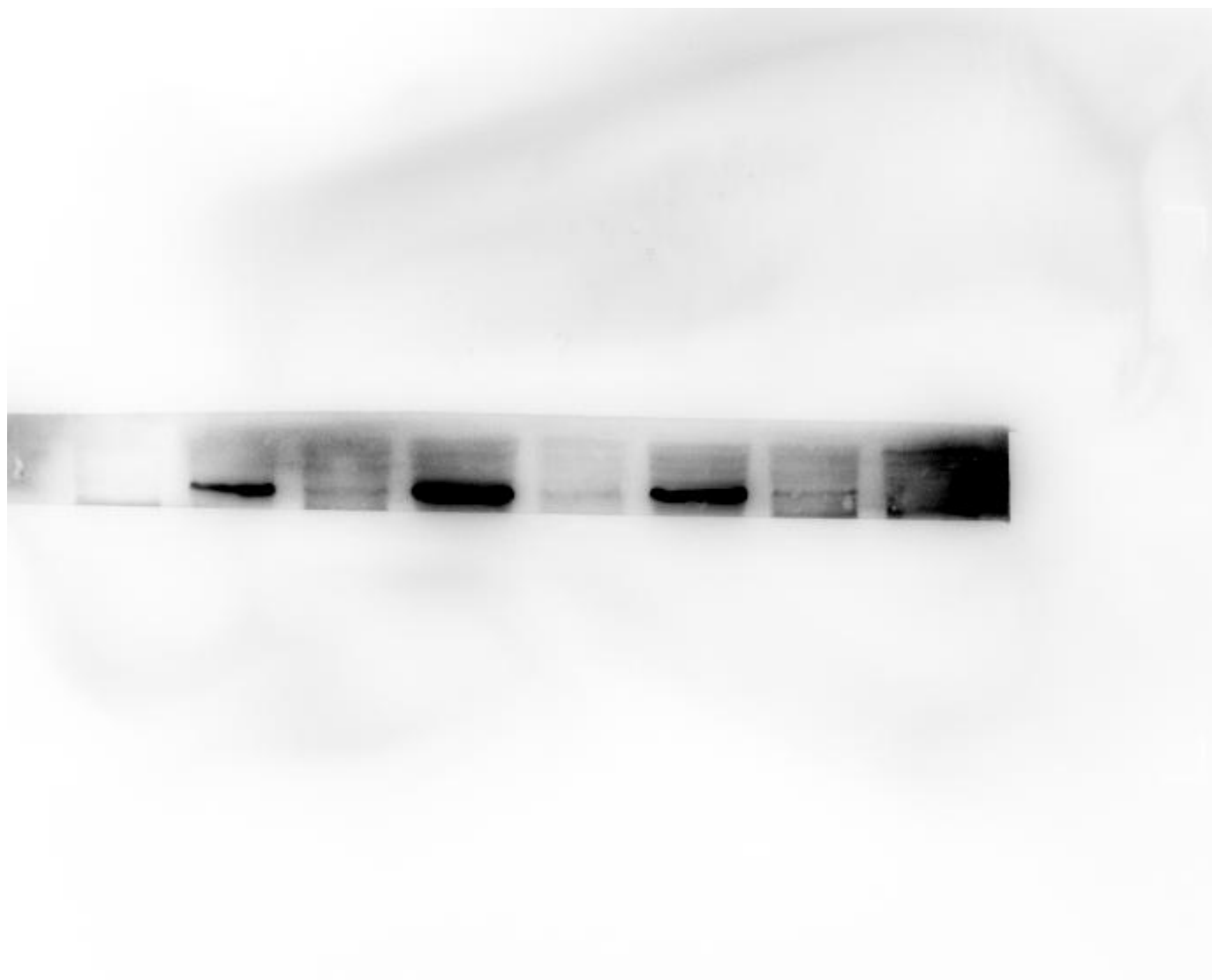

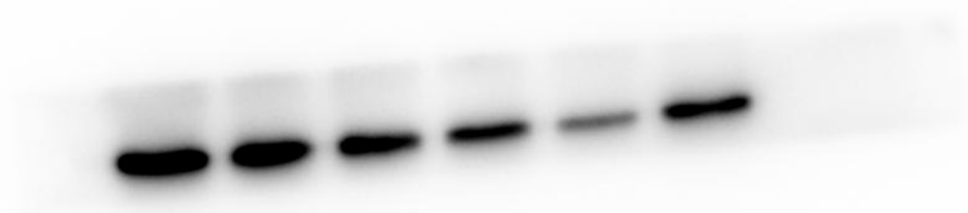

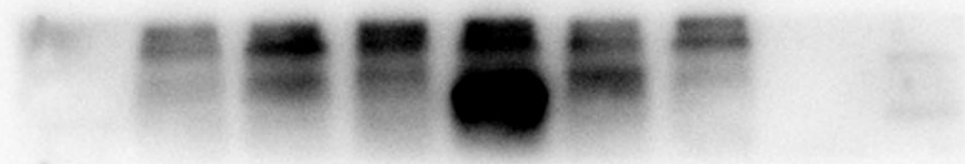

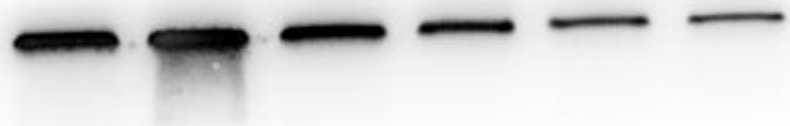

FIG2

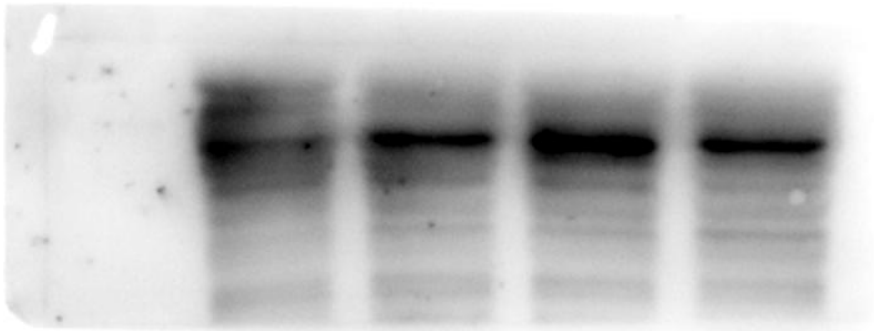

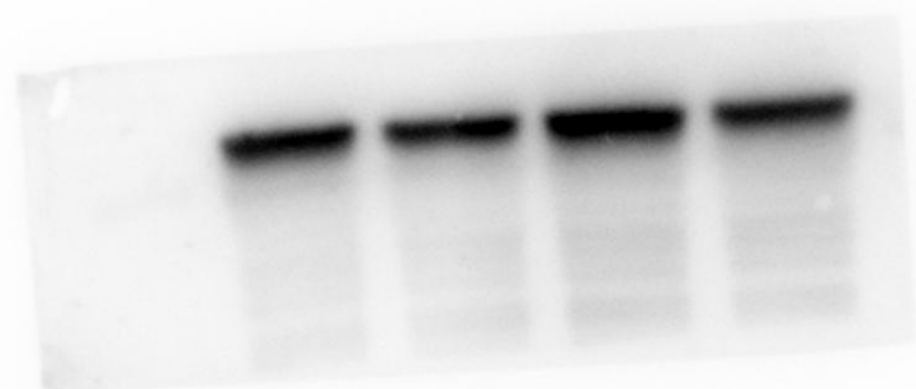

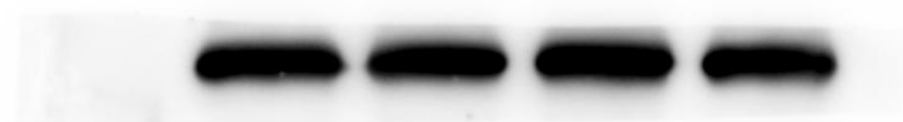

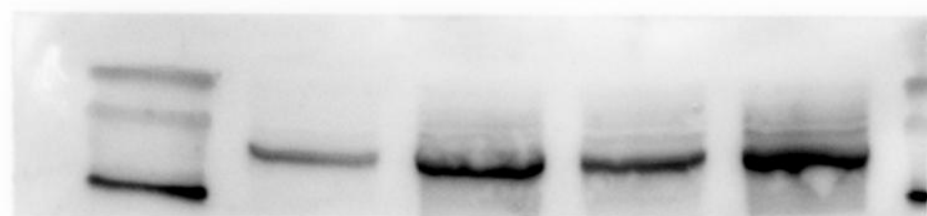

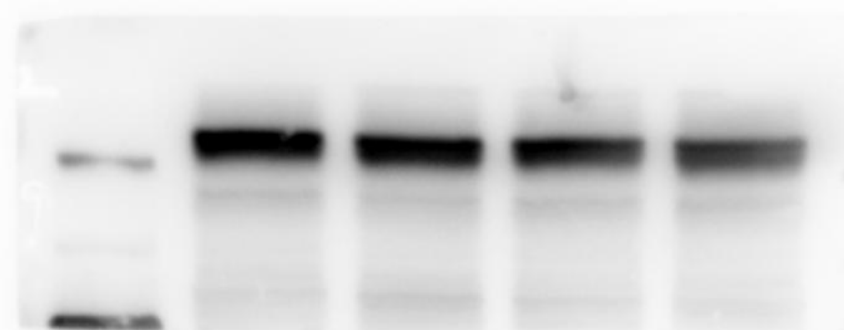

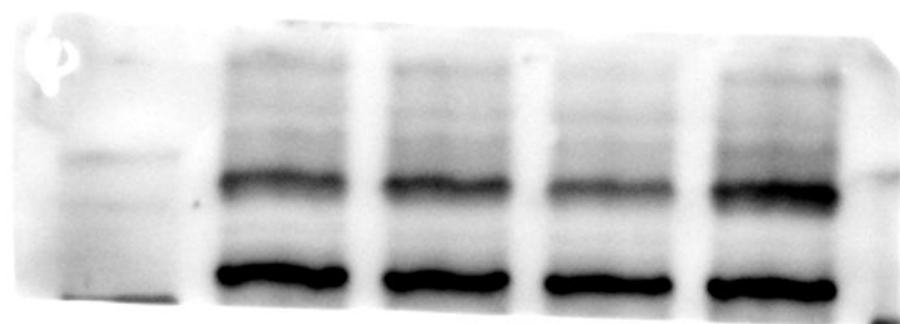

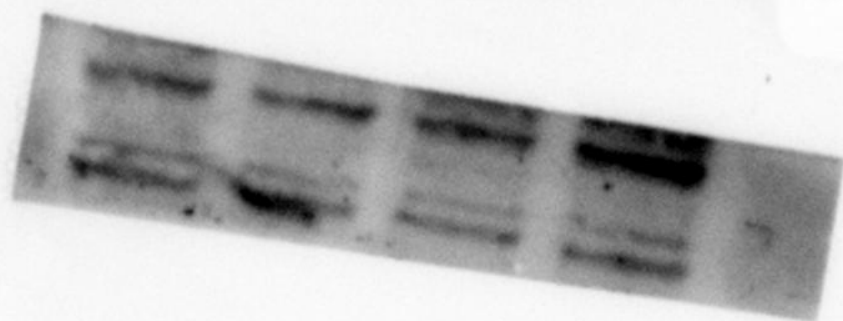

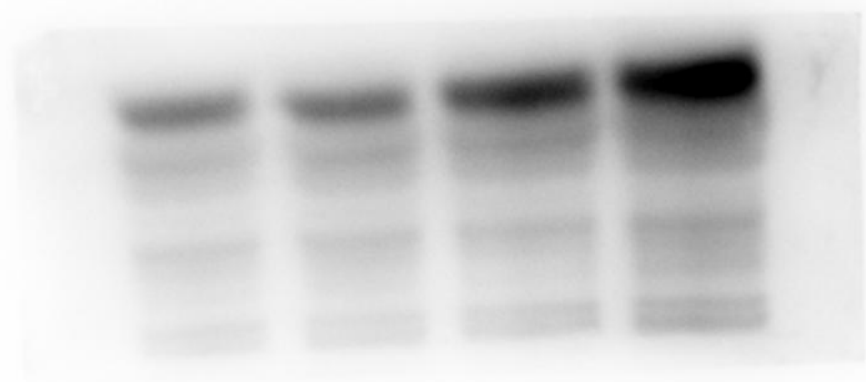

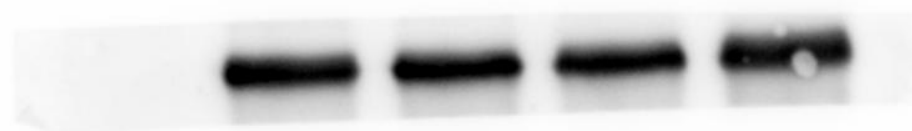

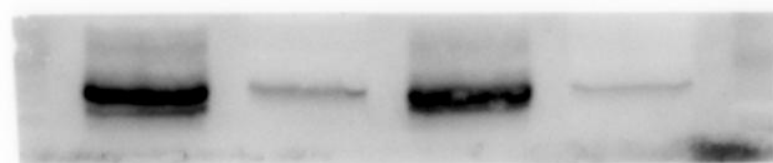

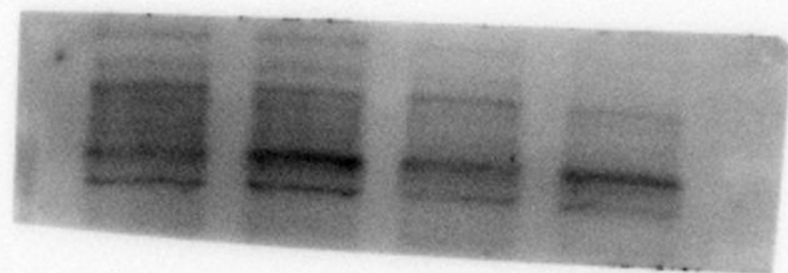

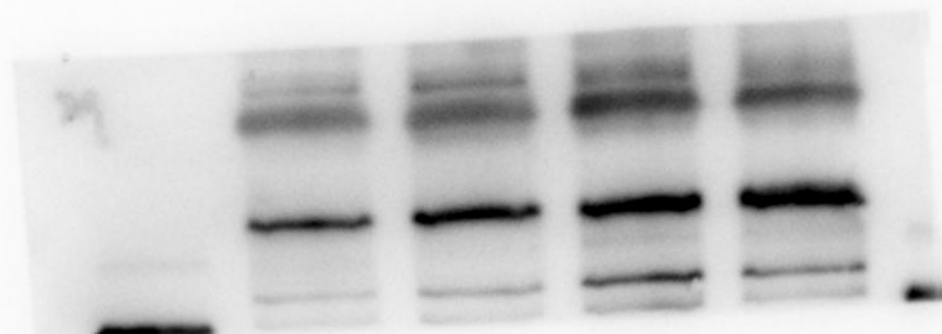

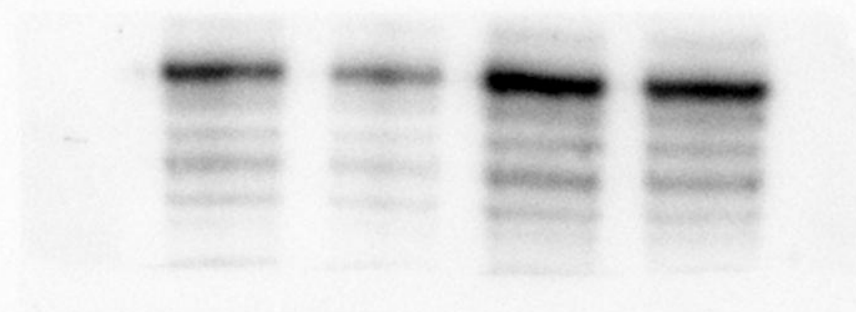

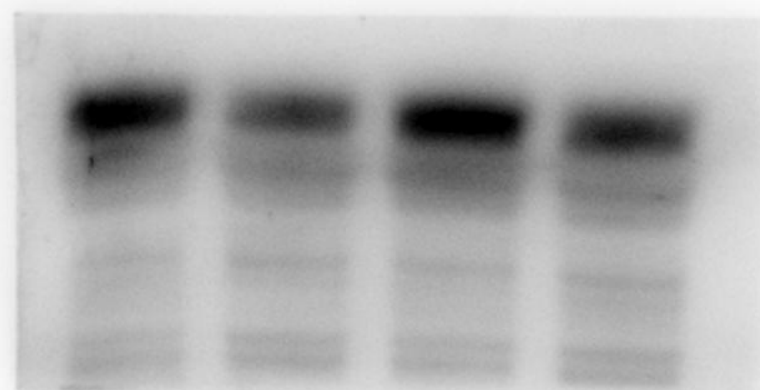

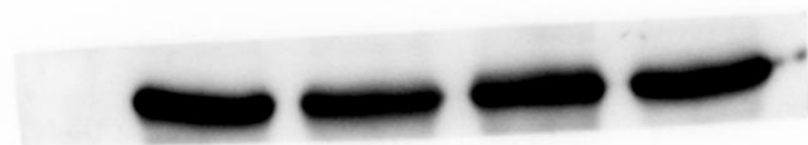

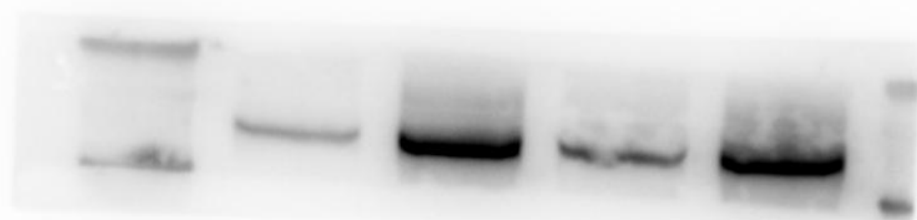

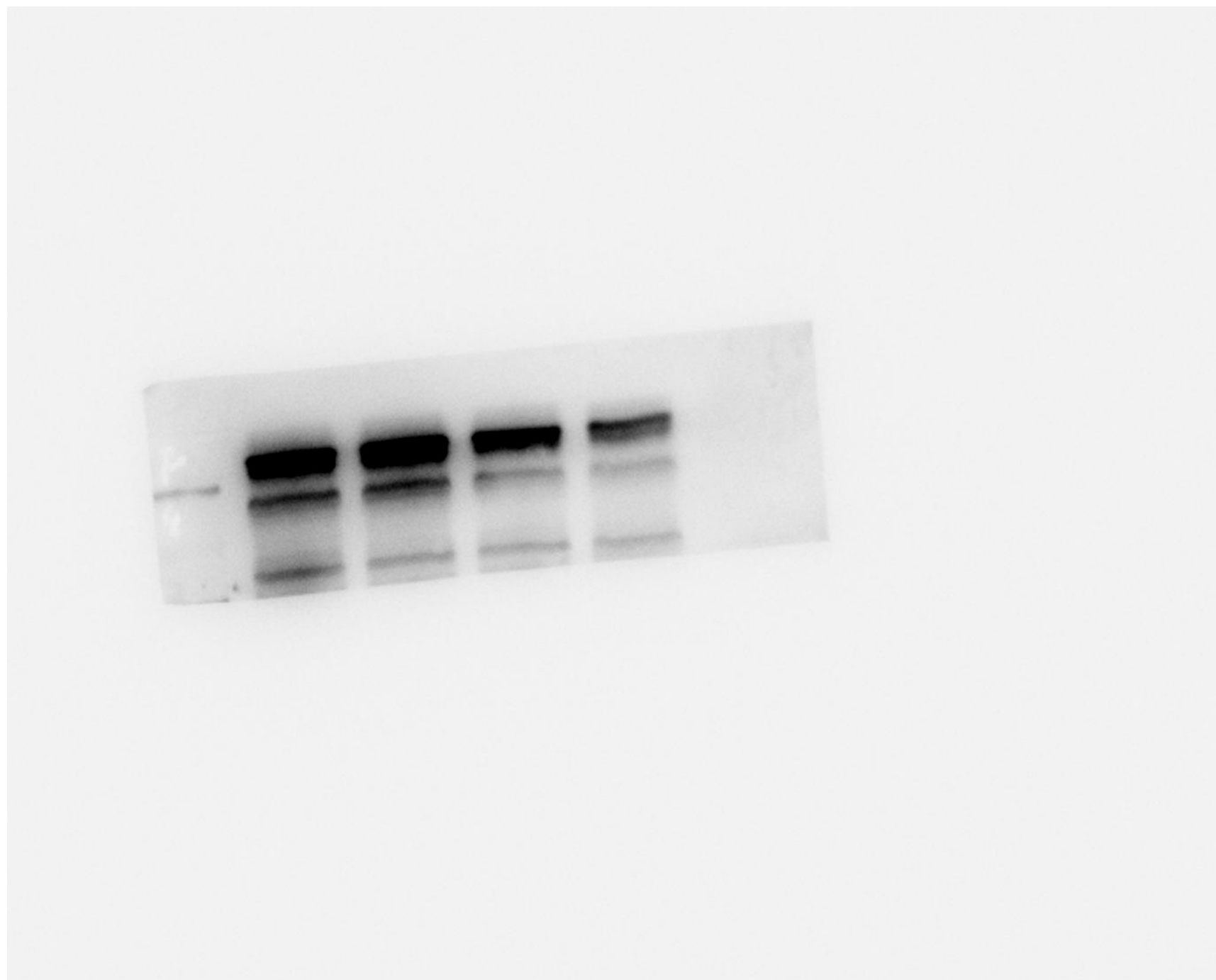

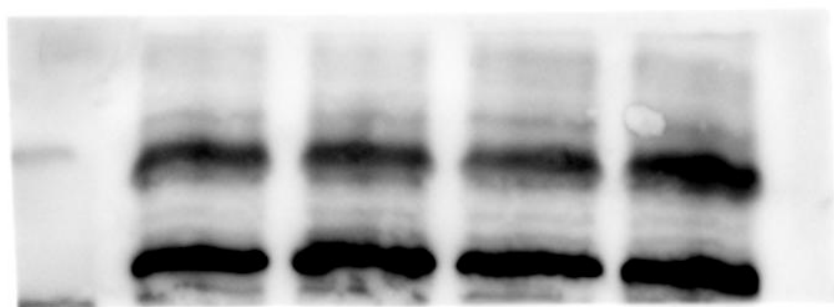

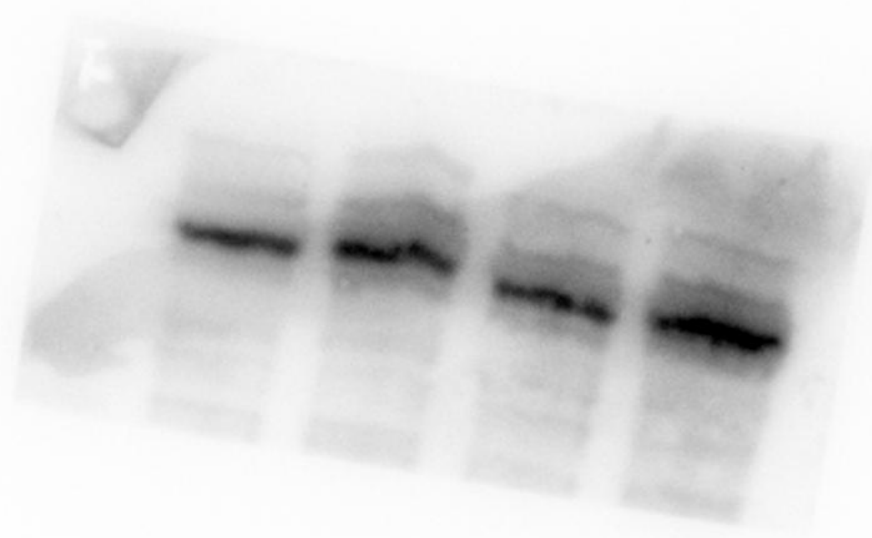

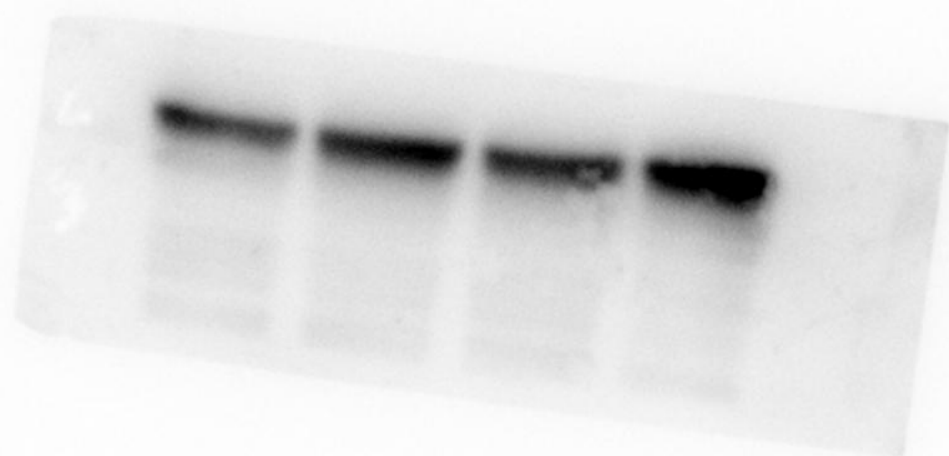

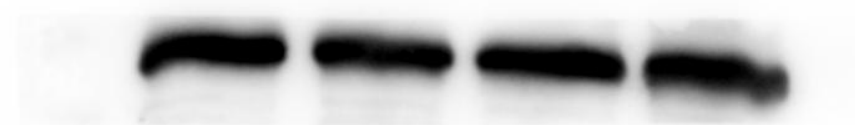

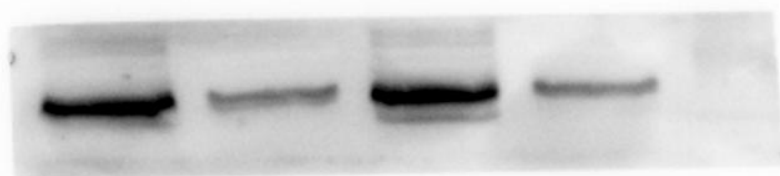

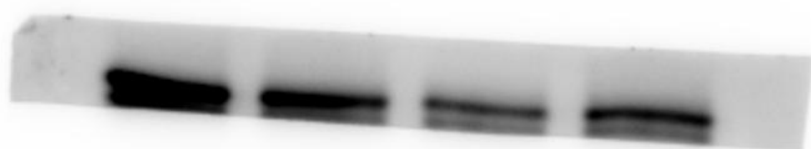

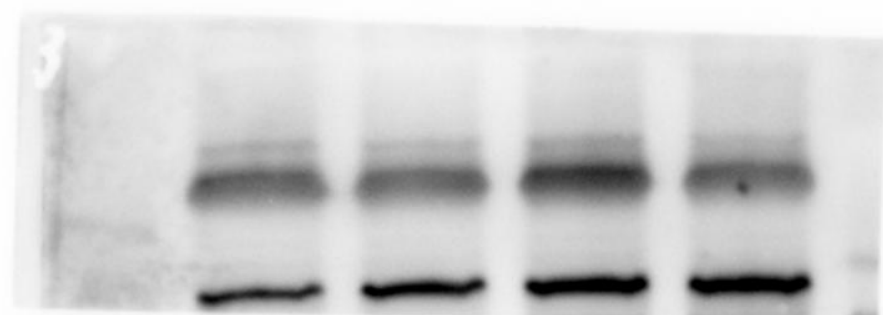

FIG4

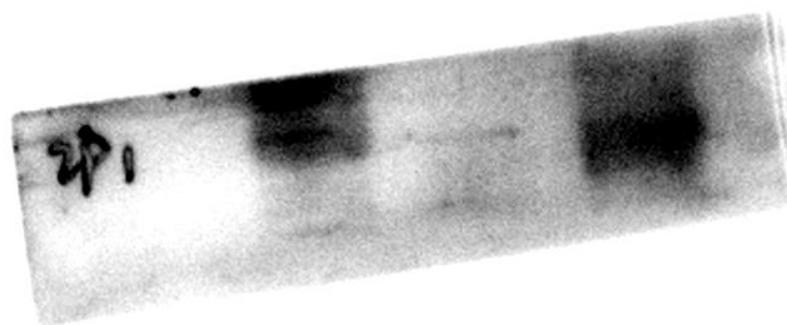

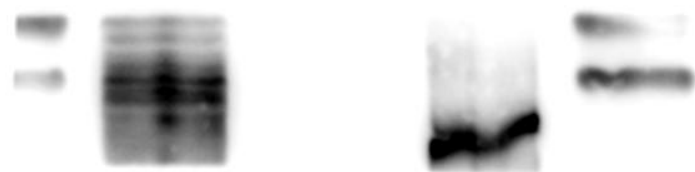

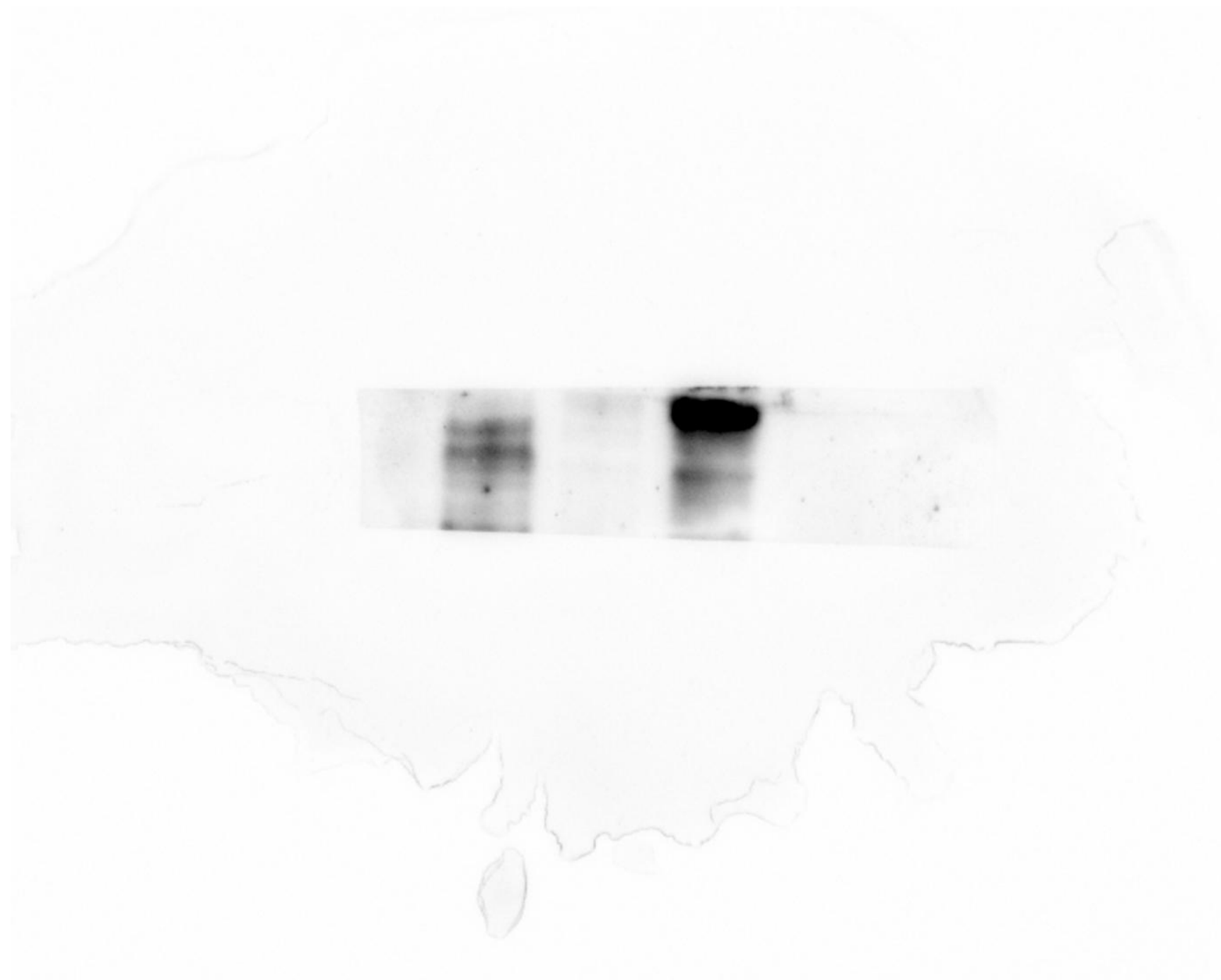

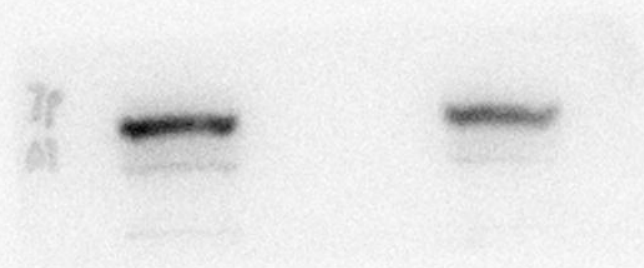

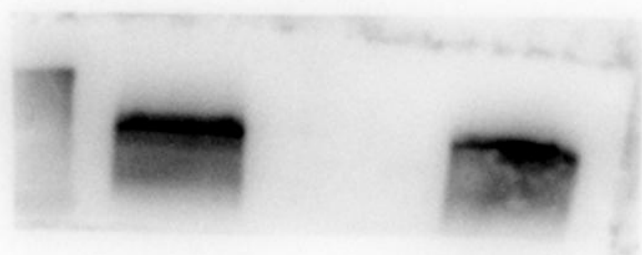

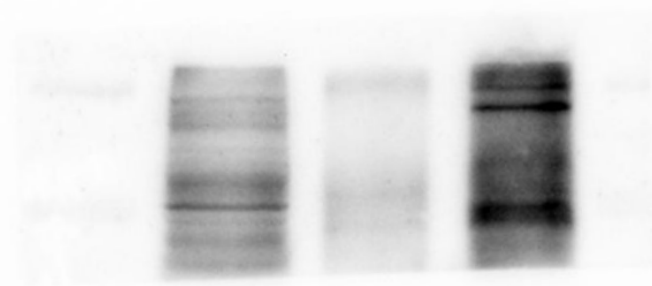

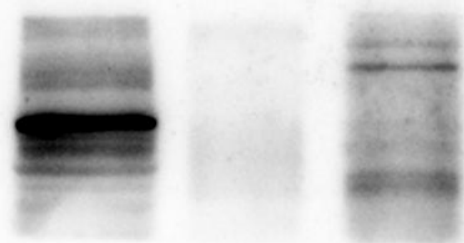

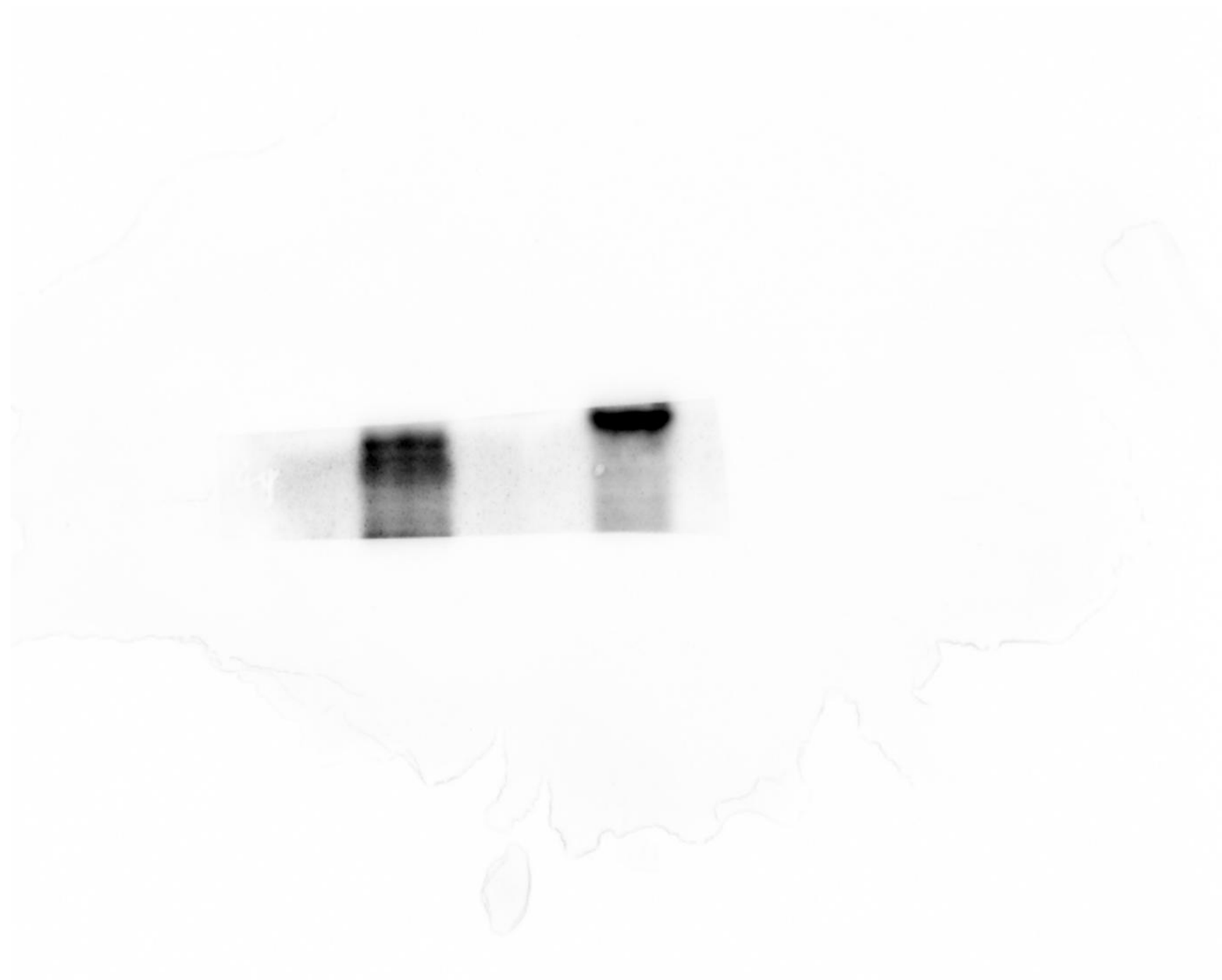

— — — —

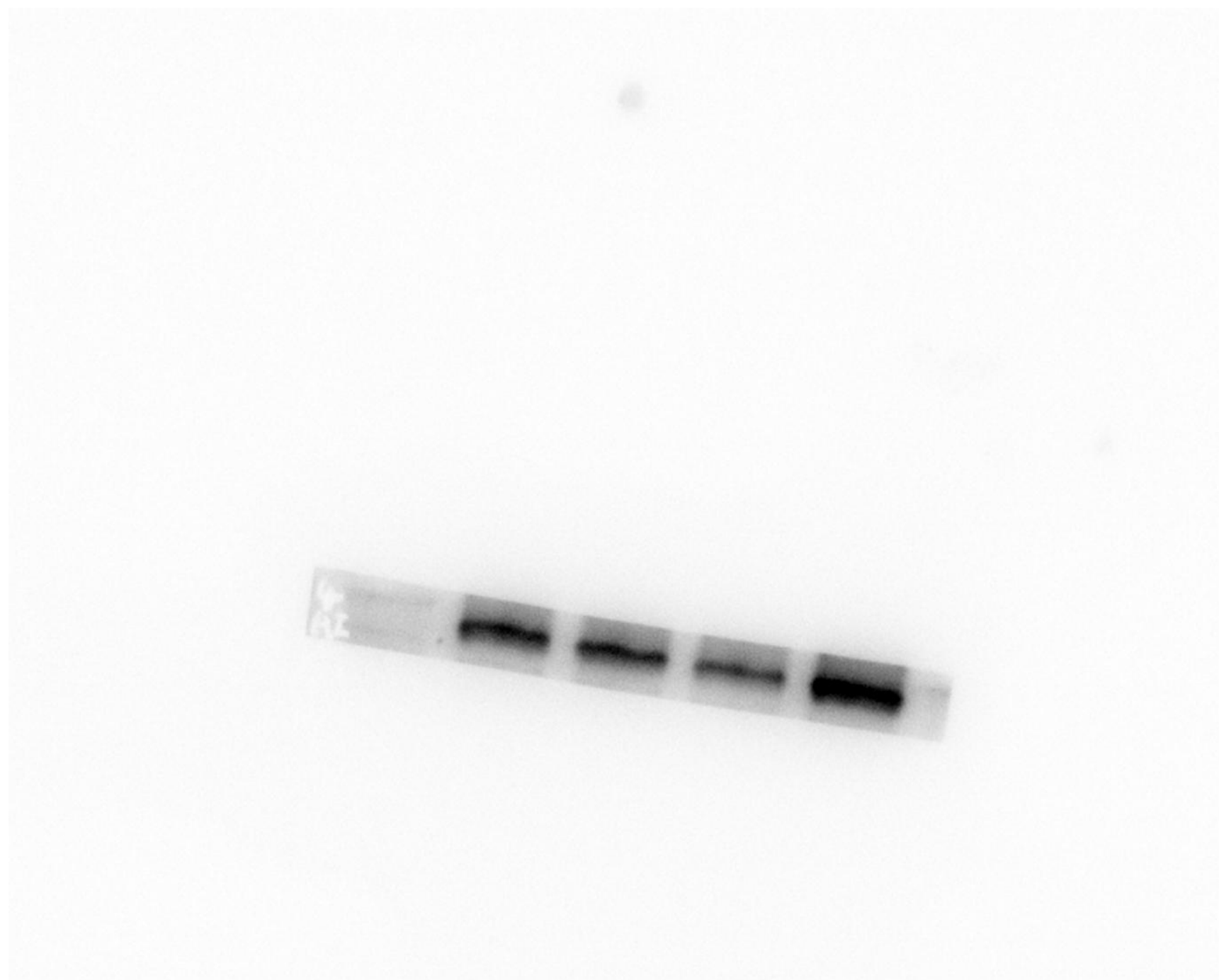

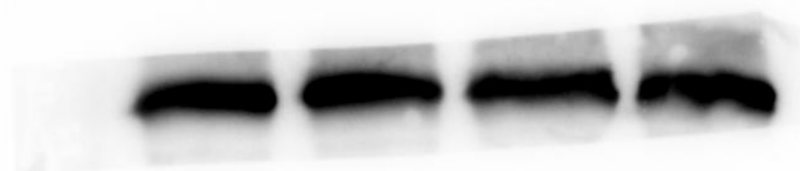

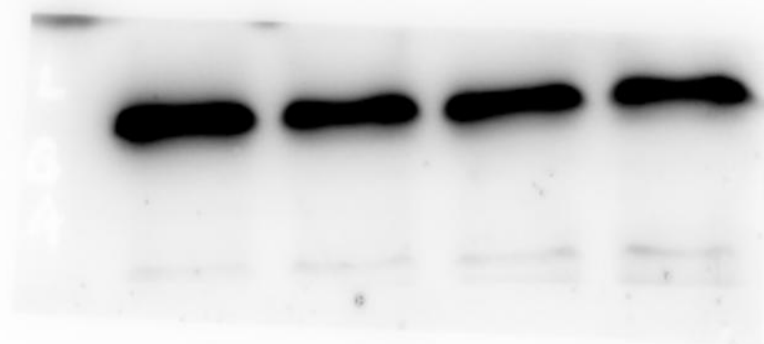

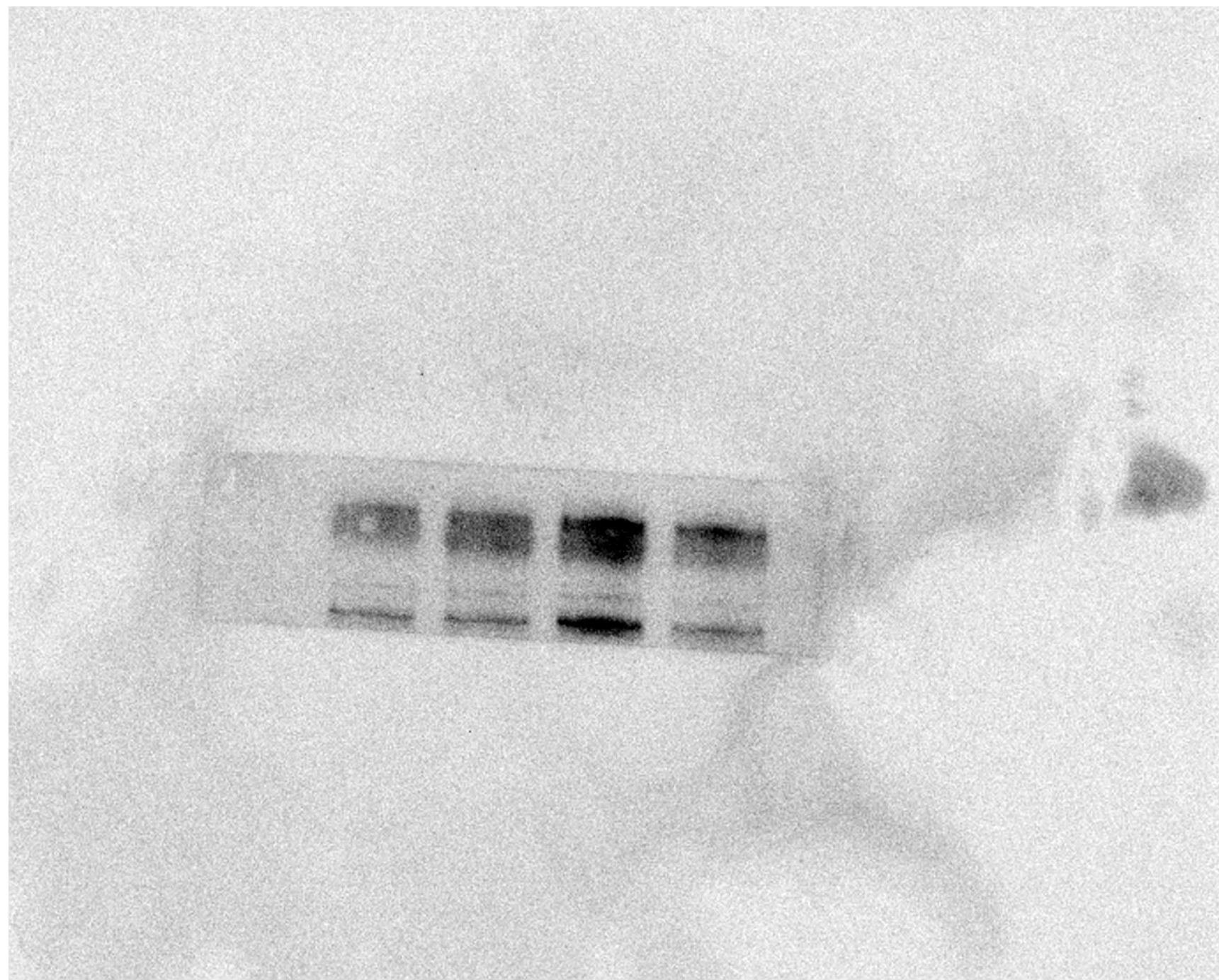

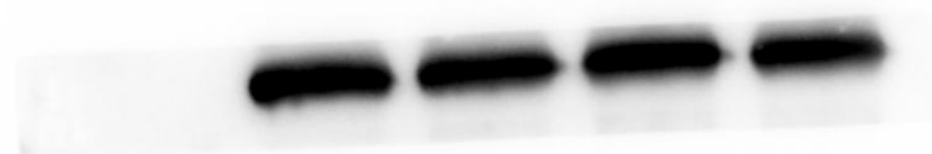

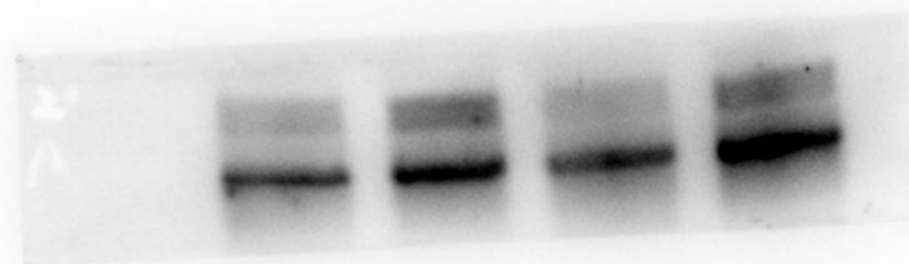

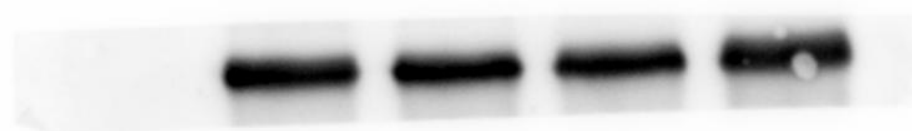

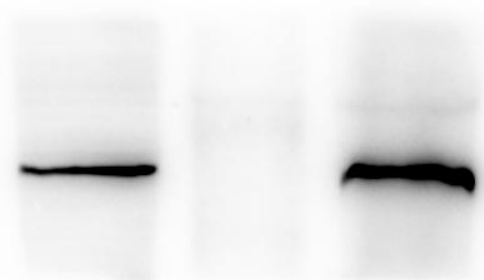

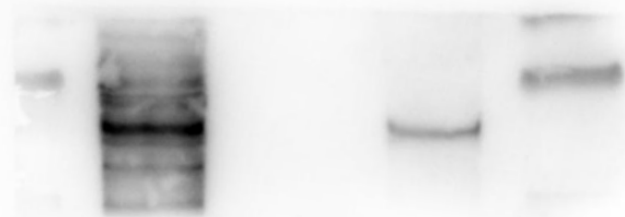

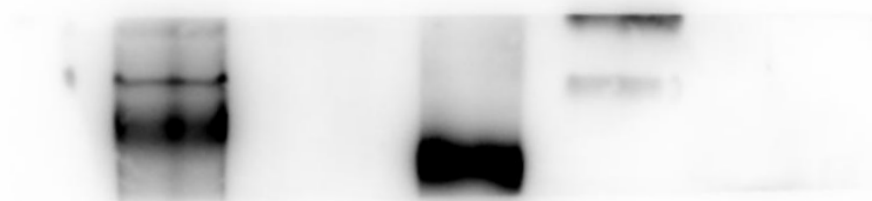

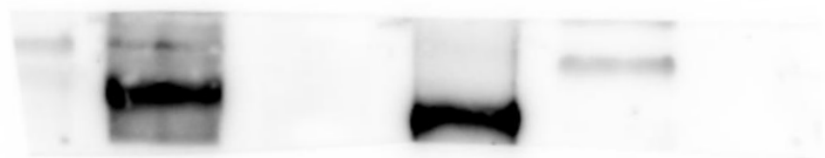

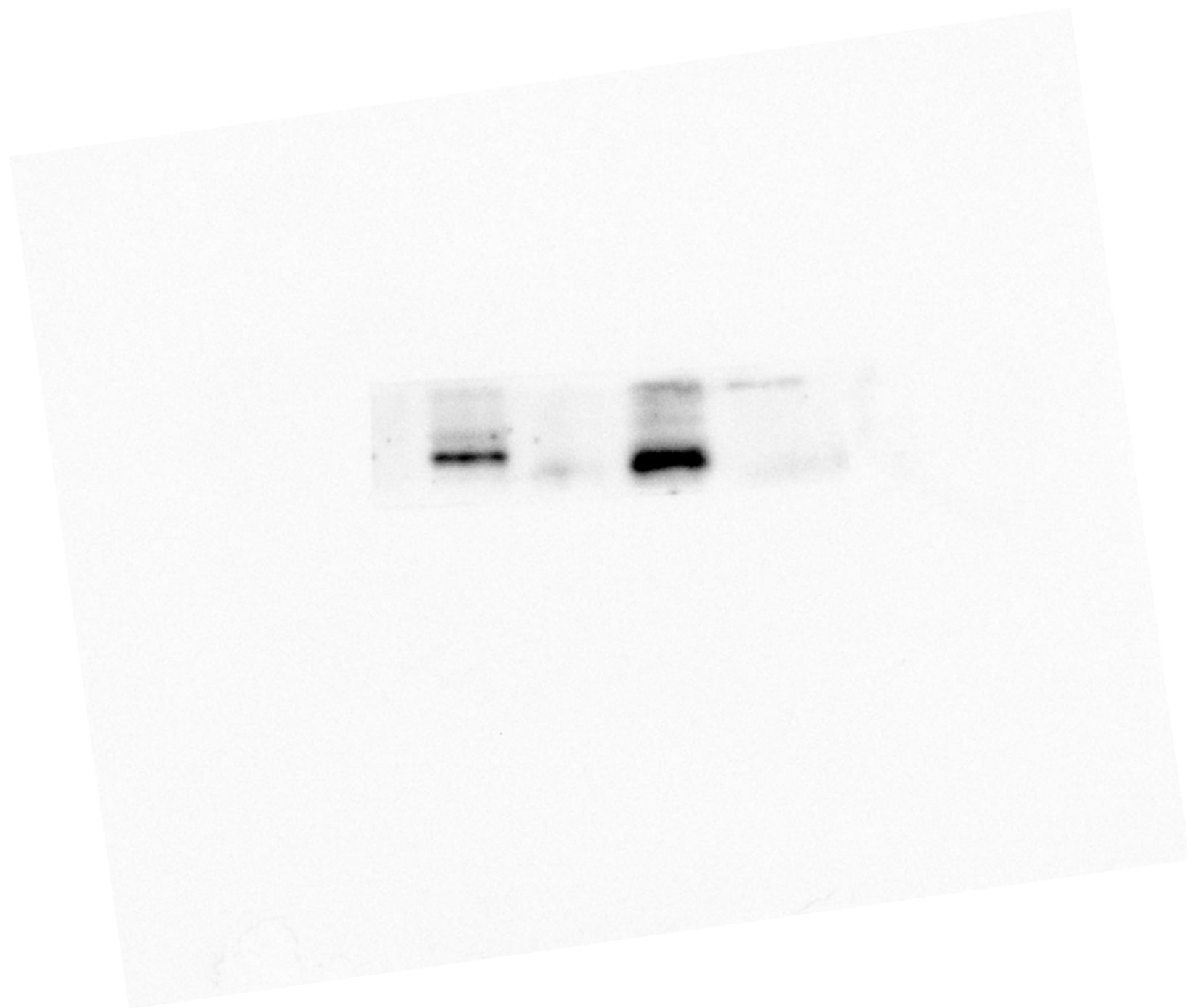

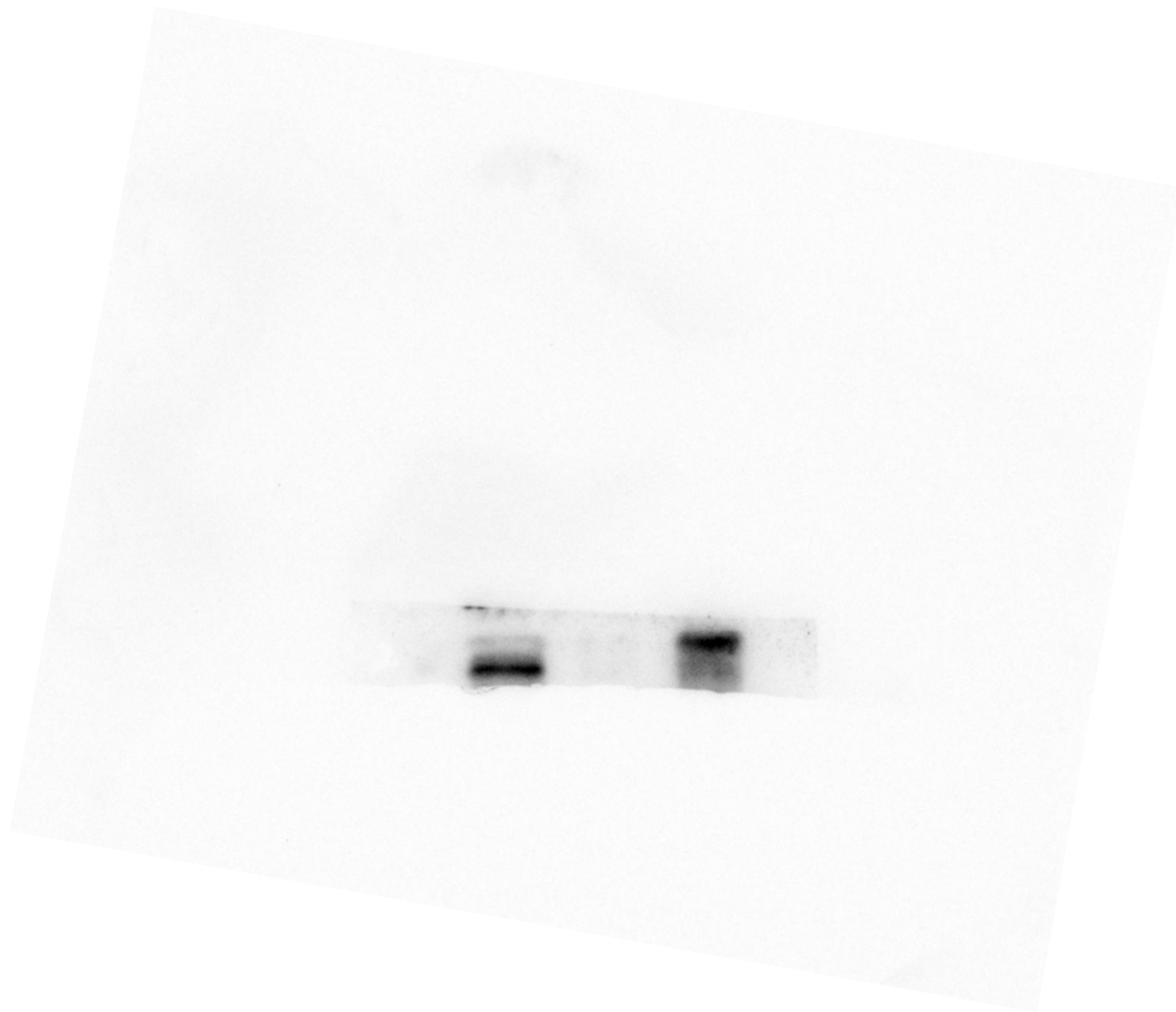

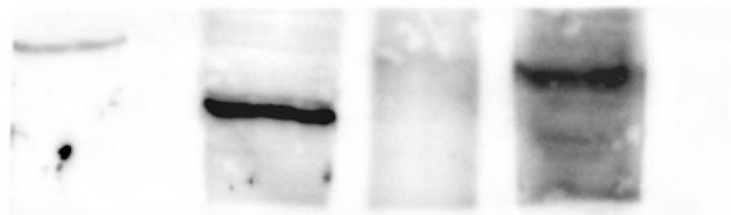

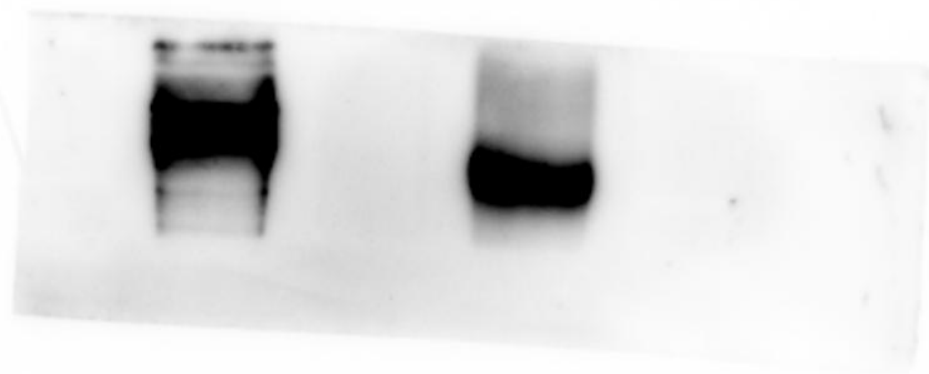

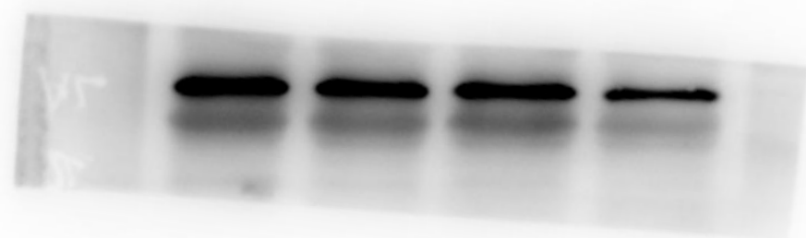

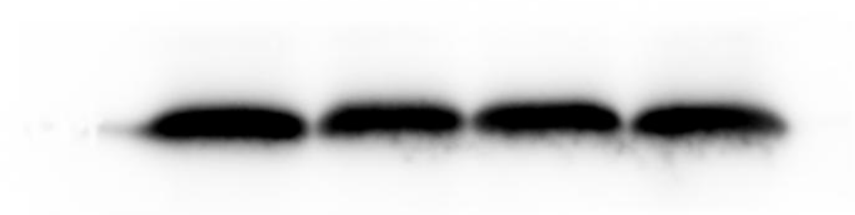

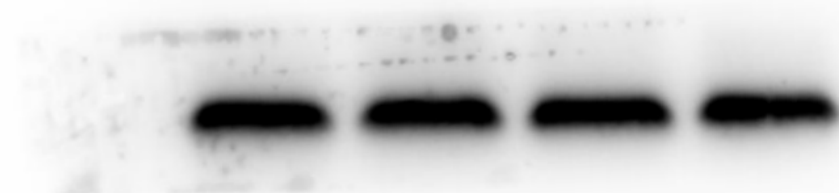

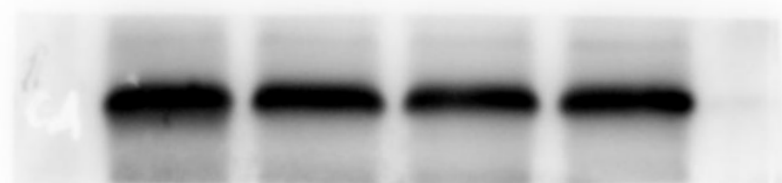

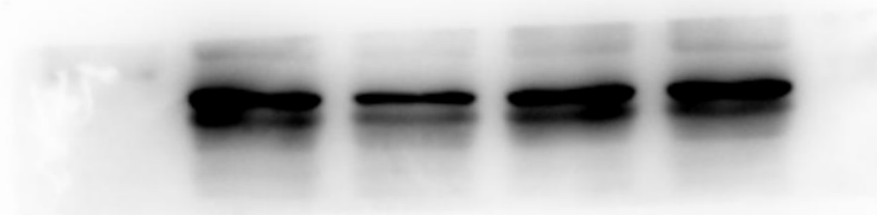

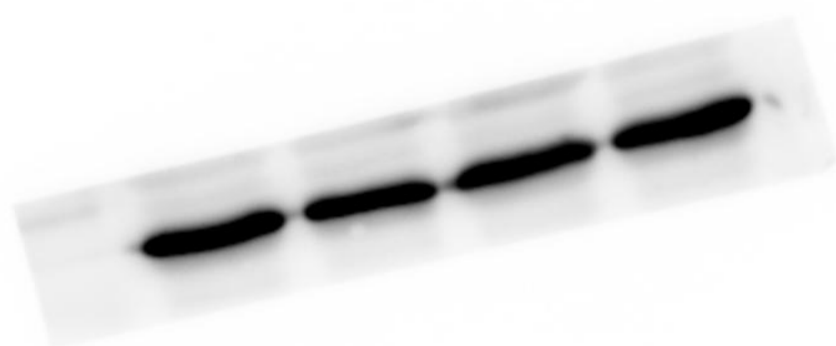

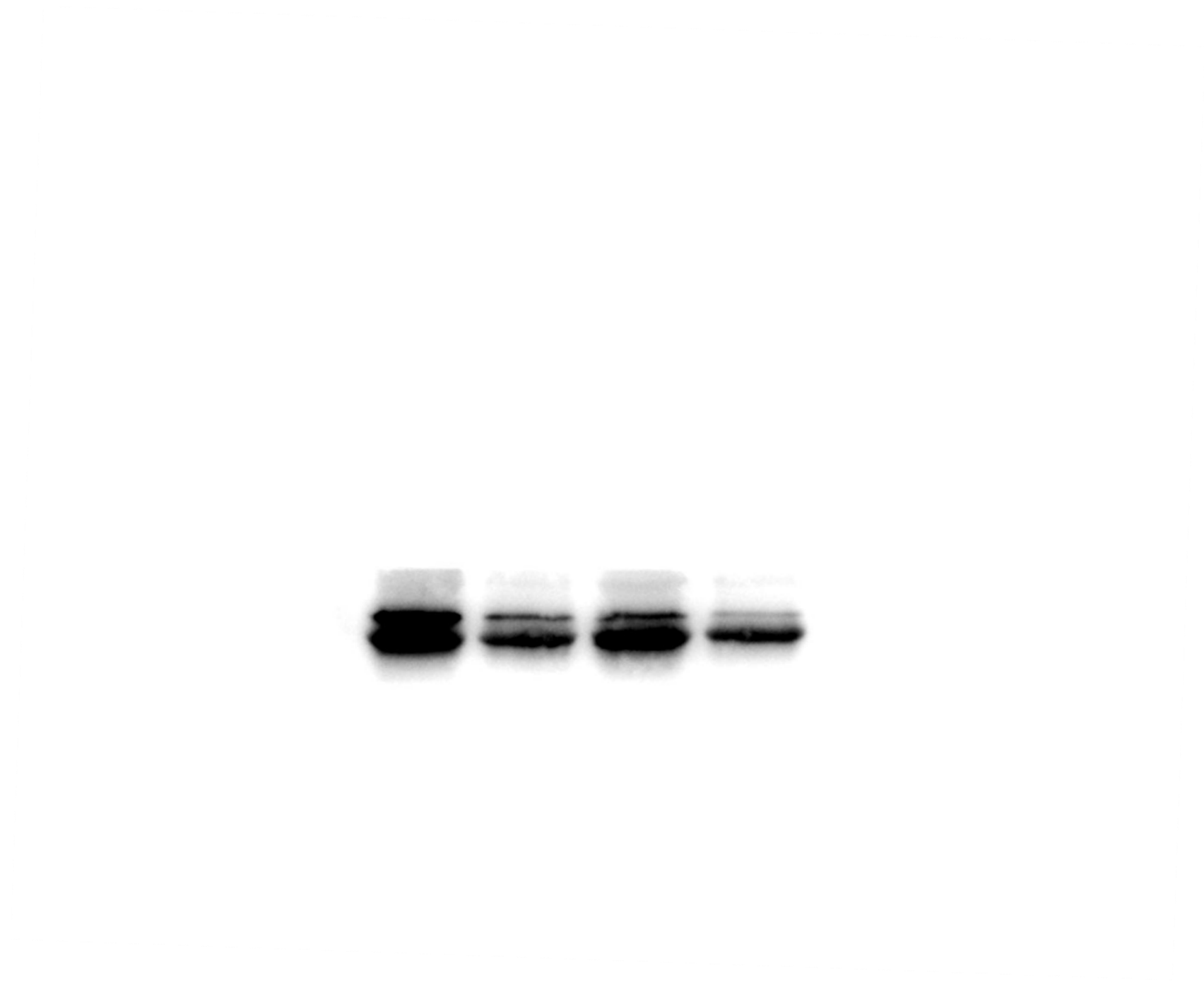

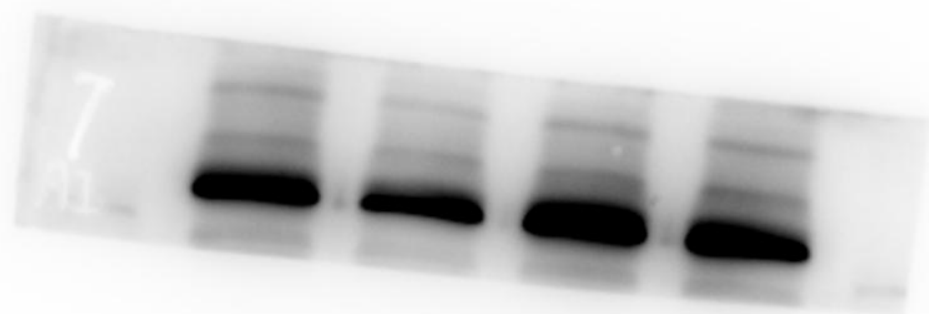

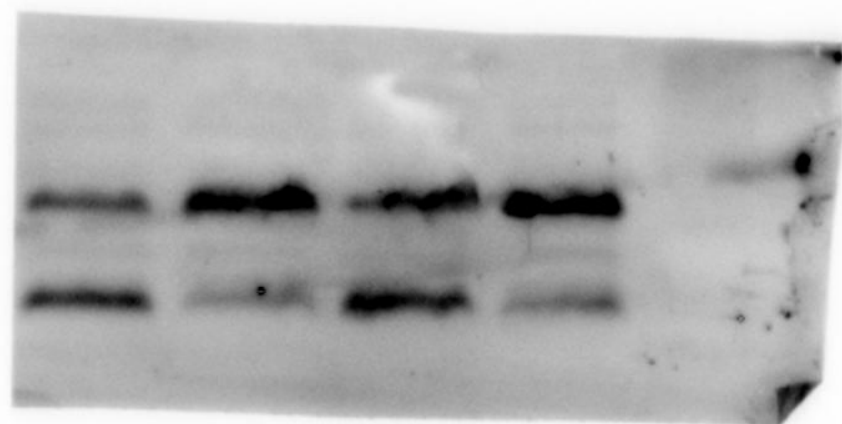

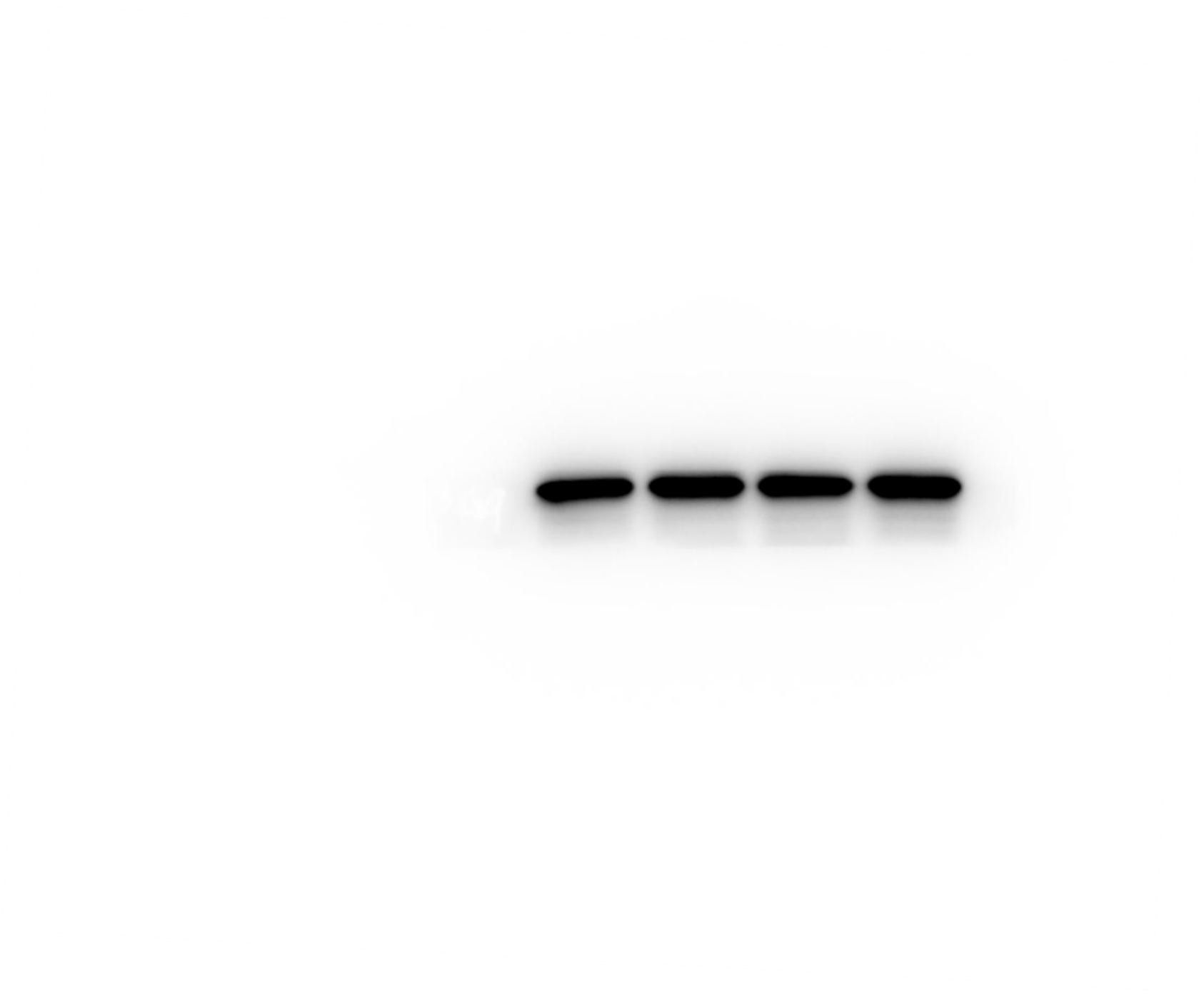

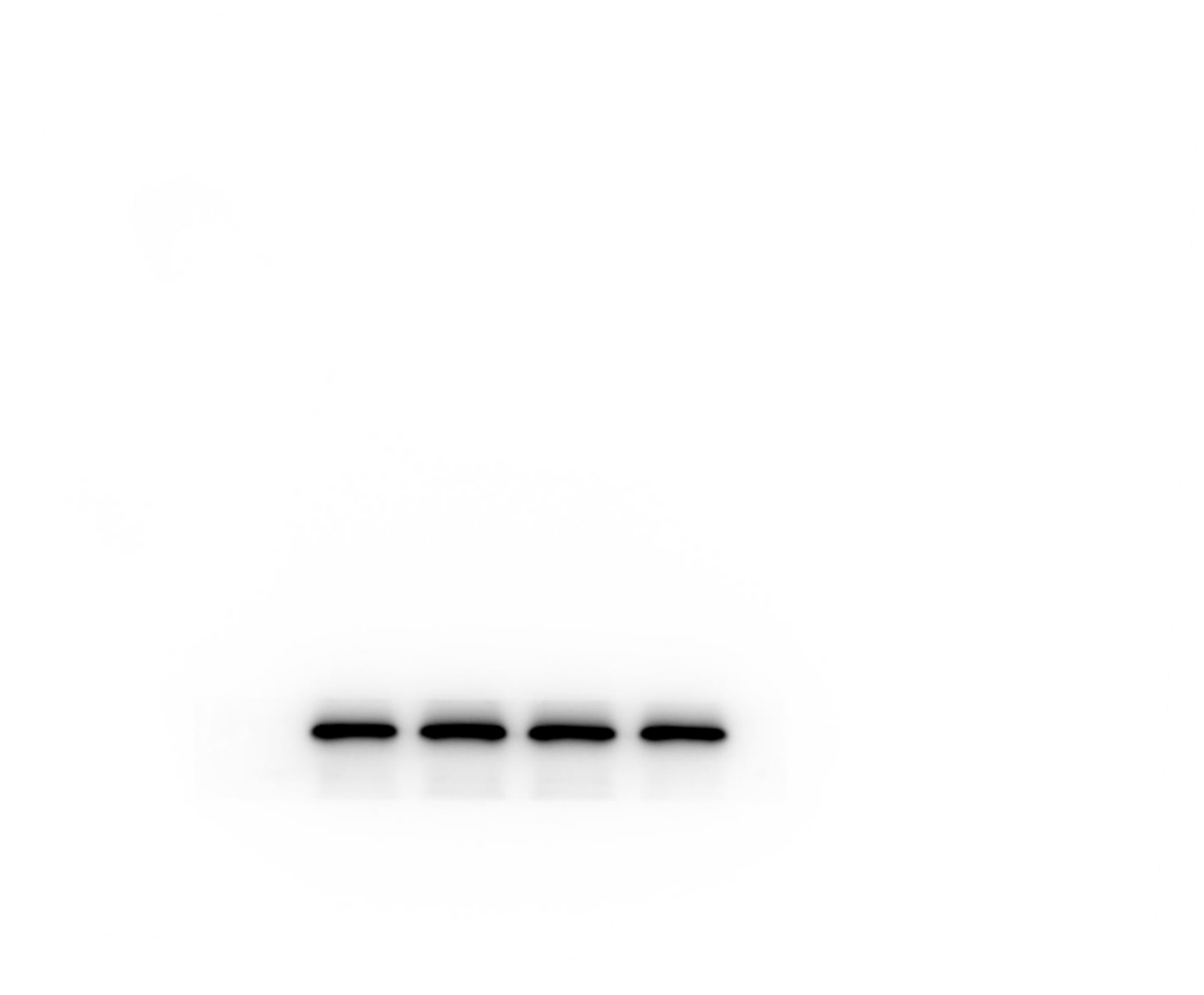

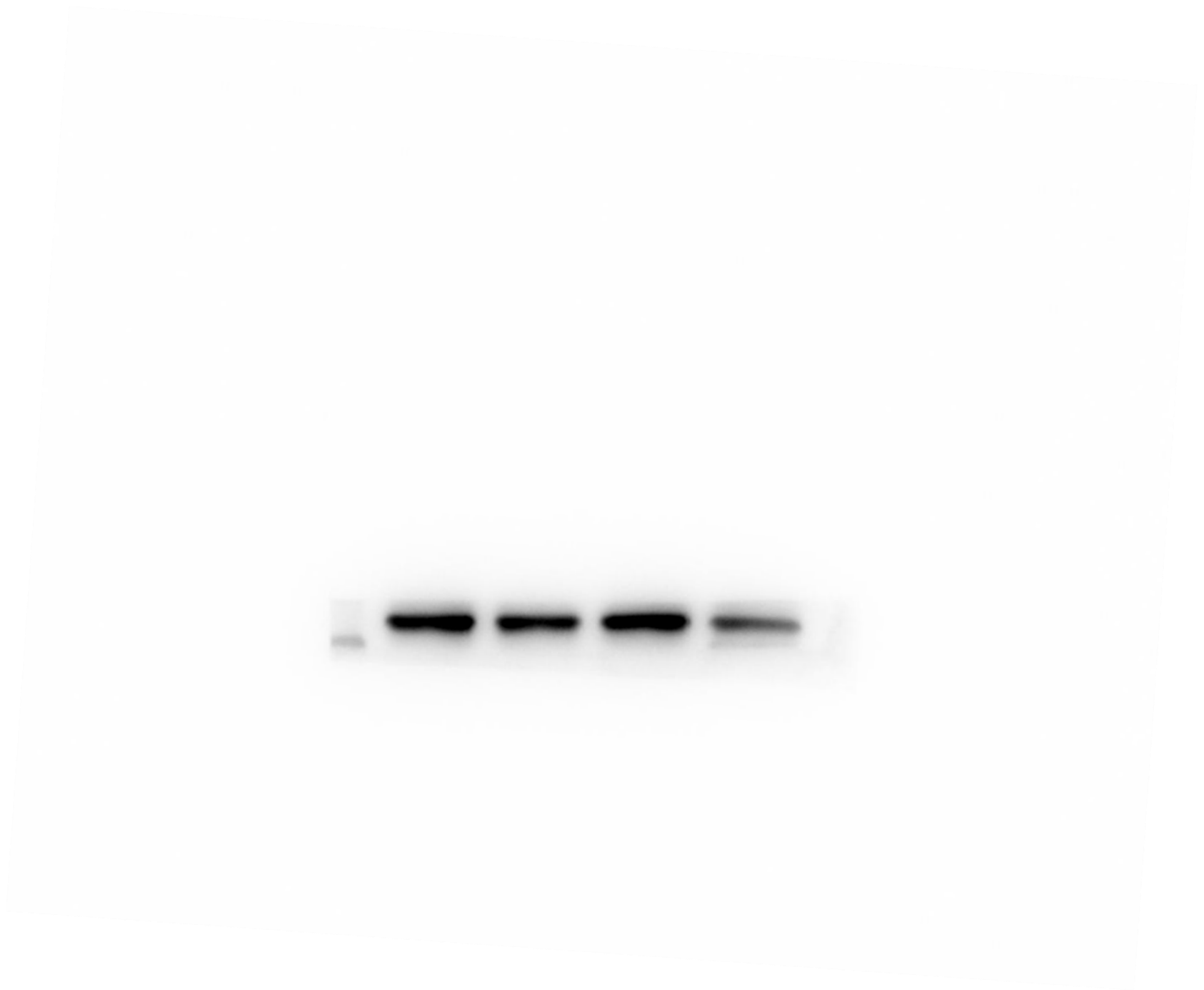

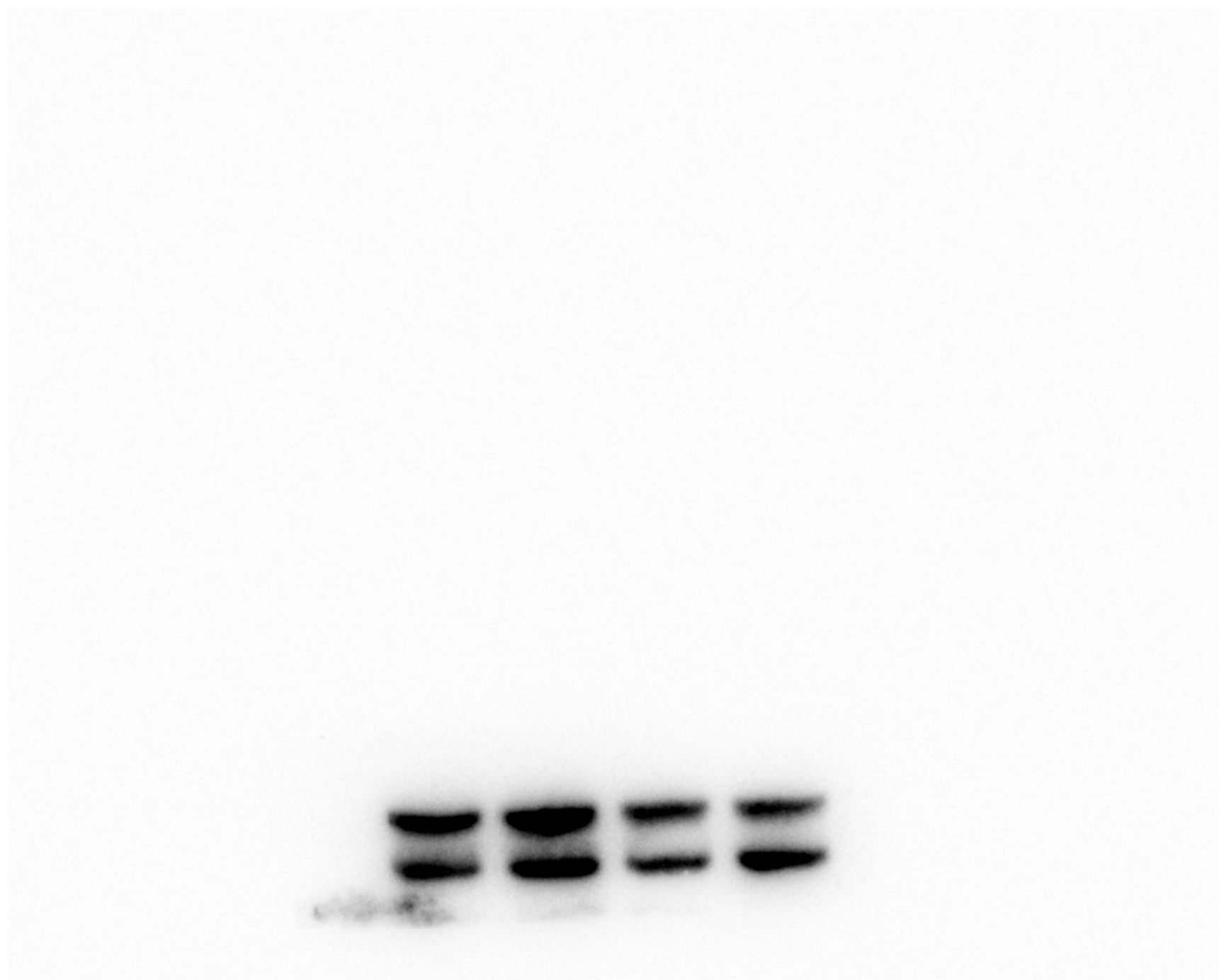

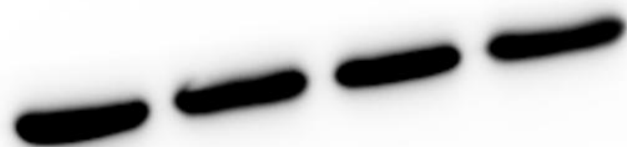

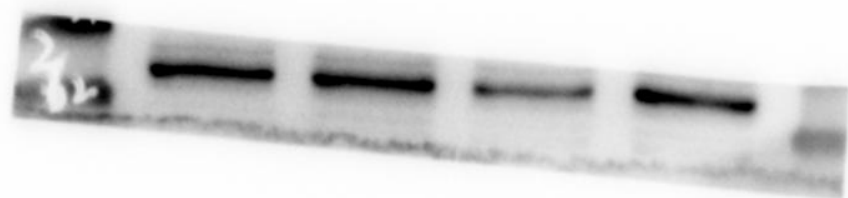

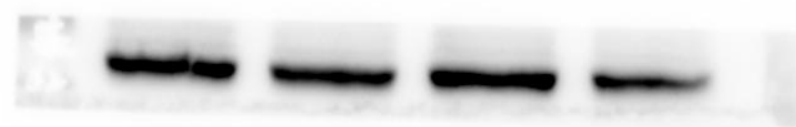

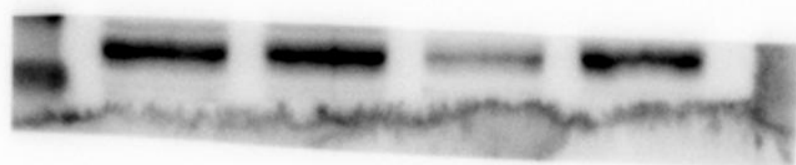

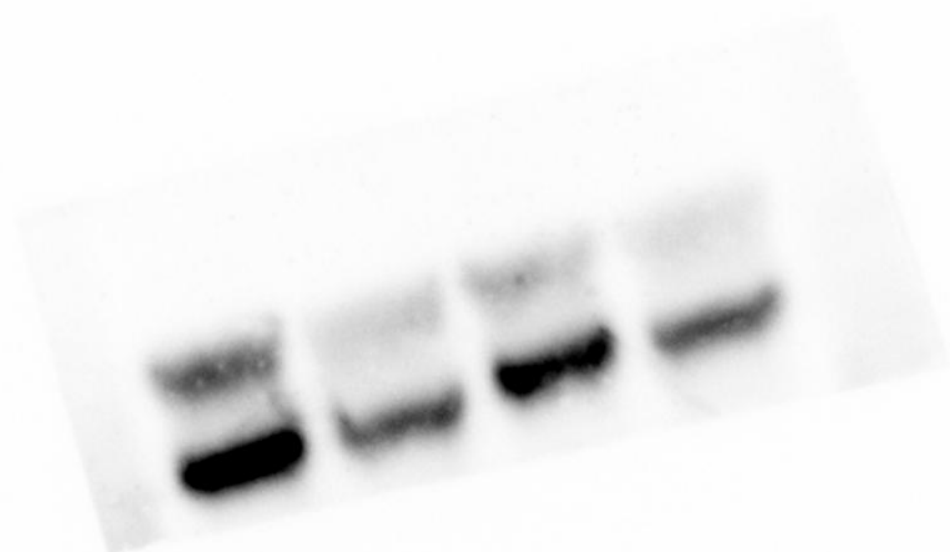

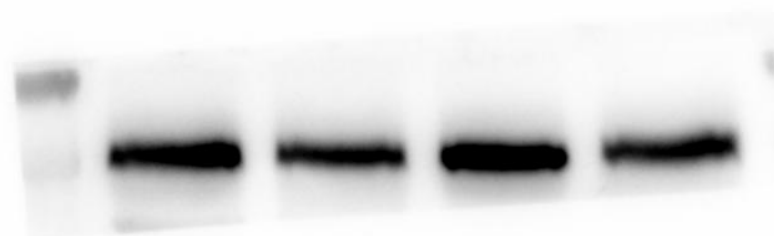

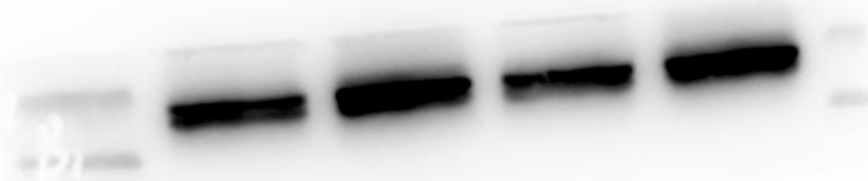

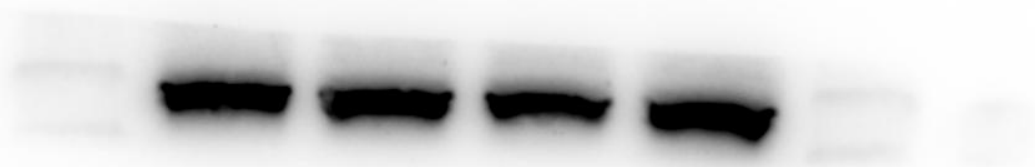

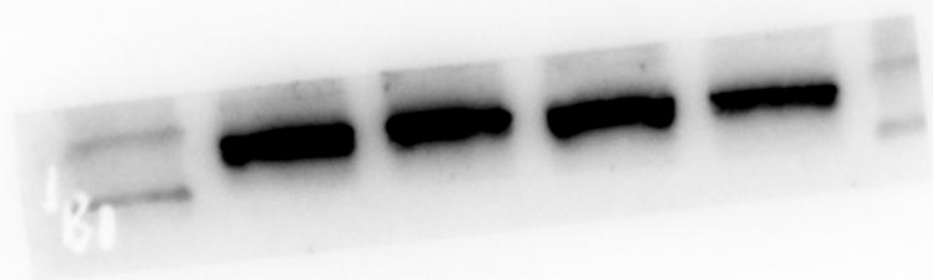

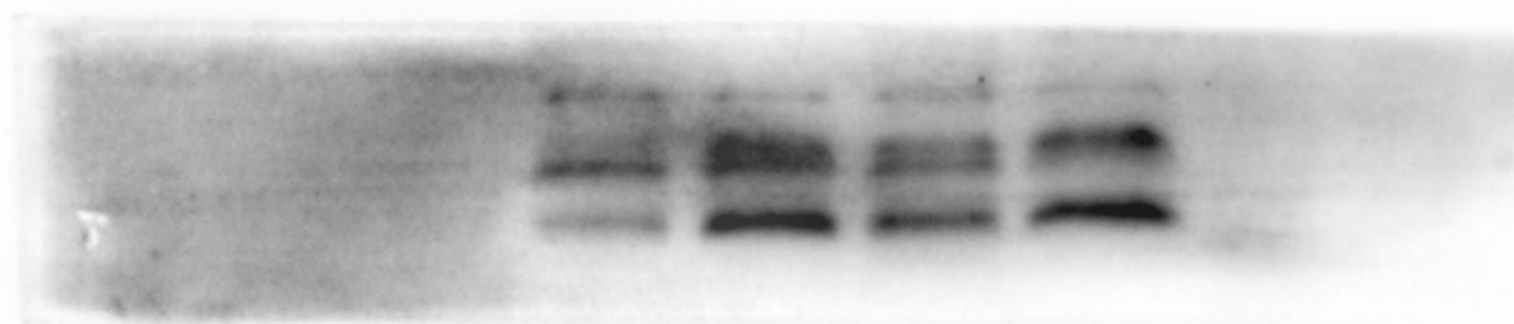

FIG5

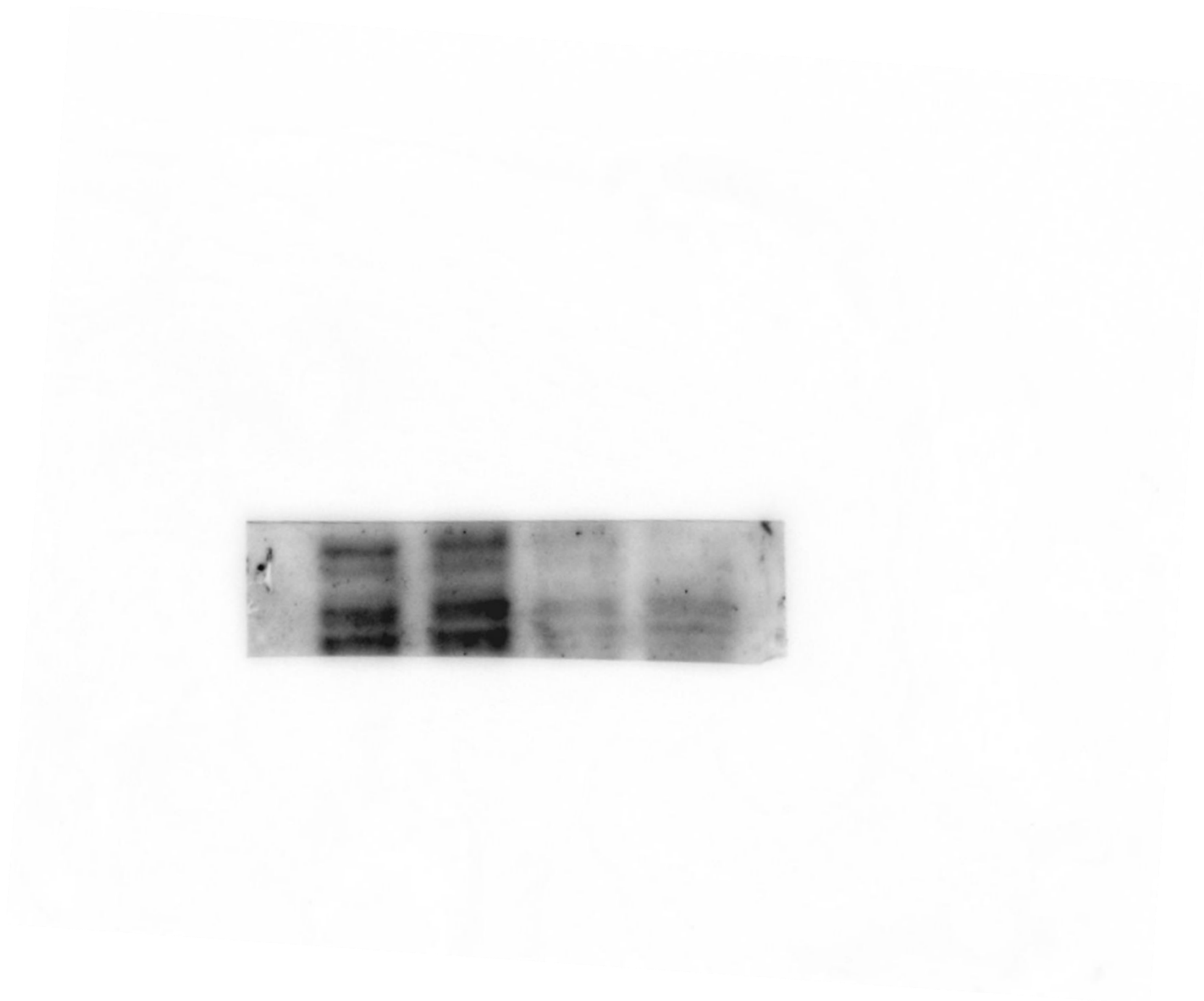

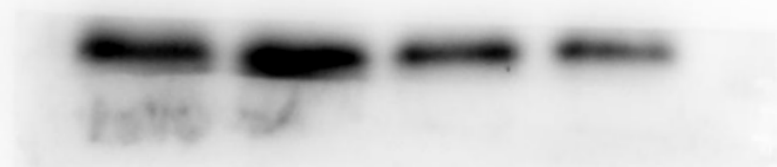

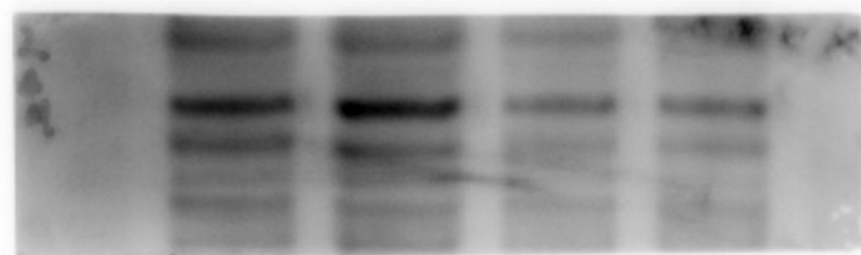

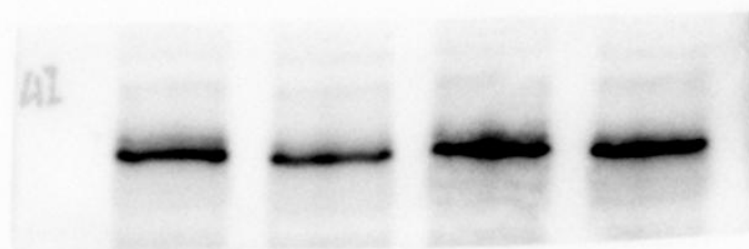

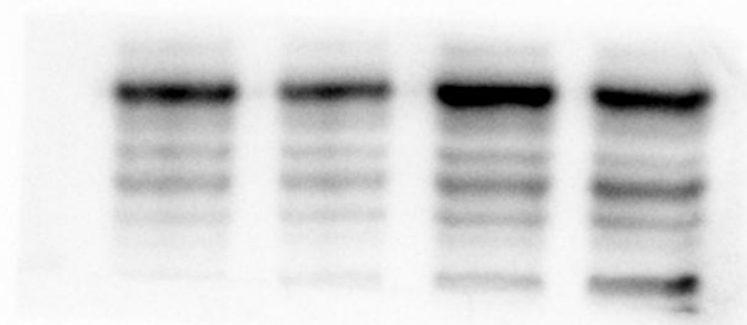

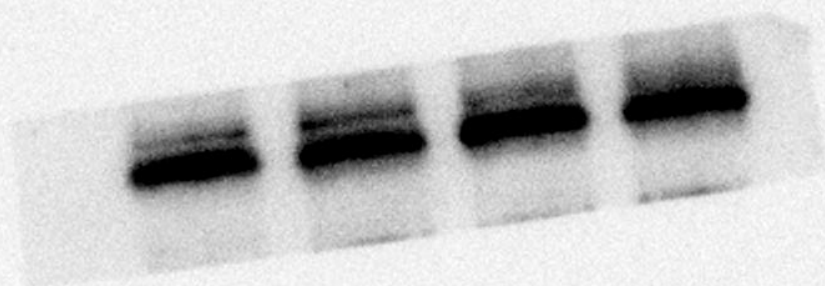

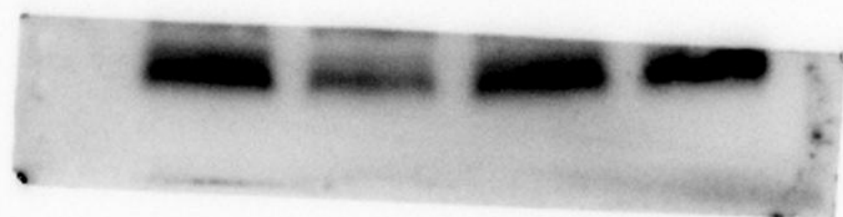

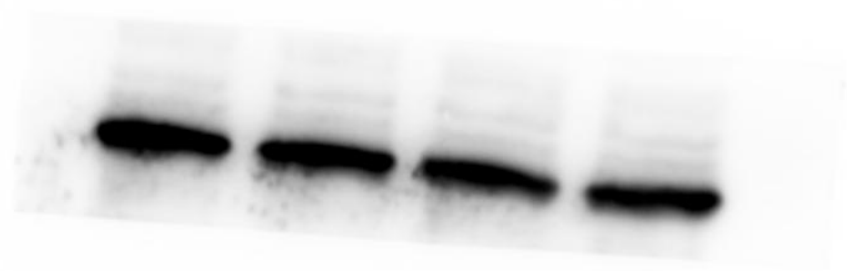

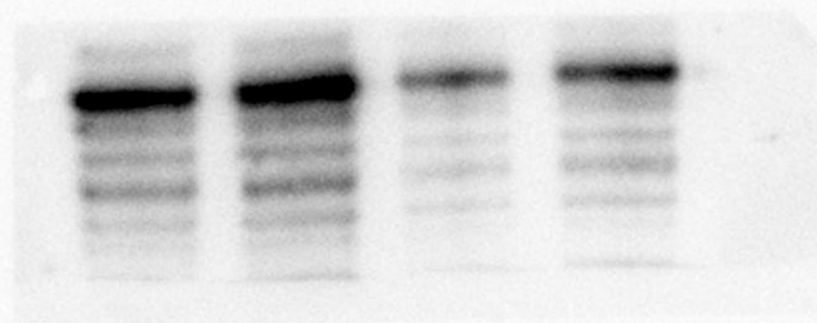

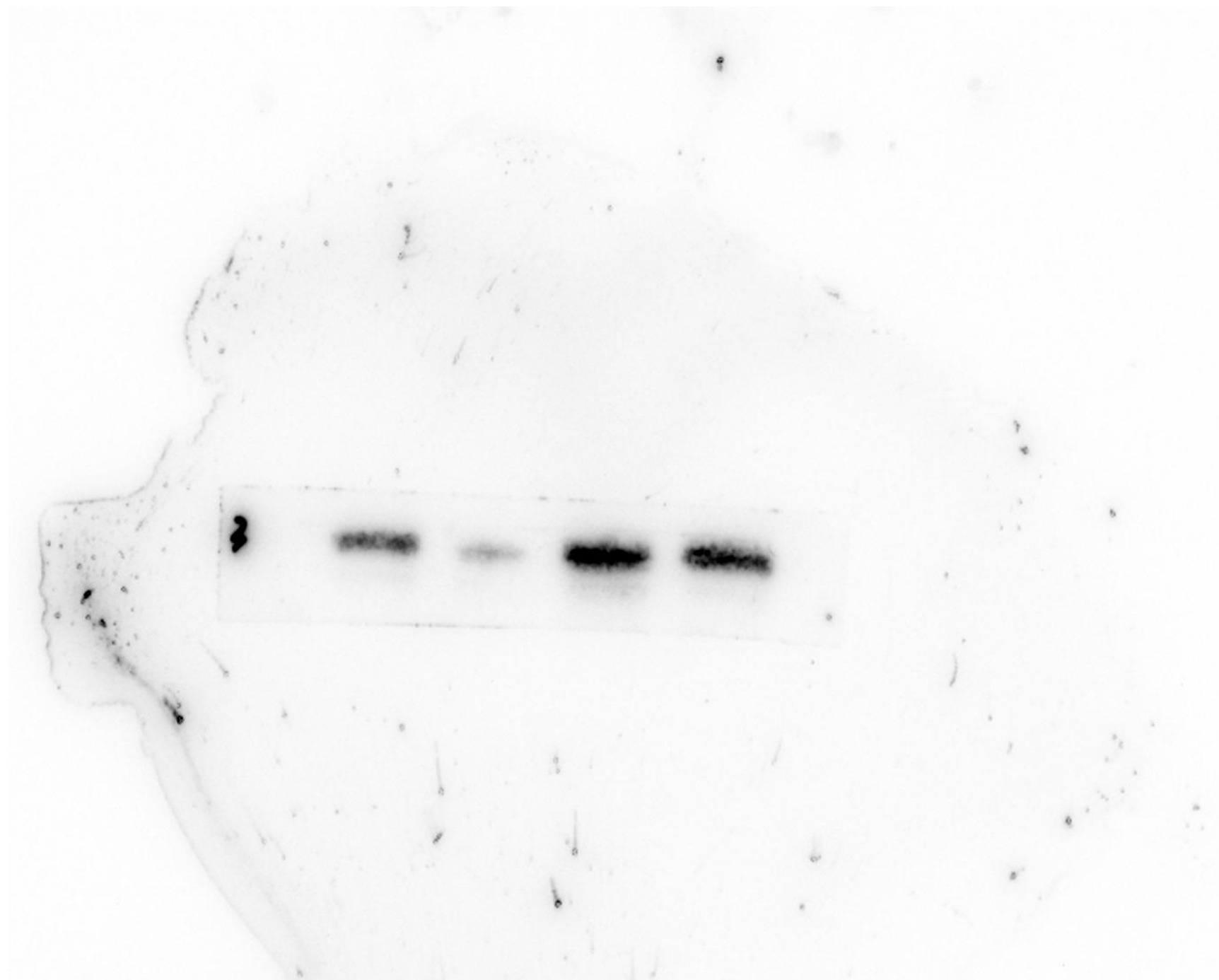

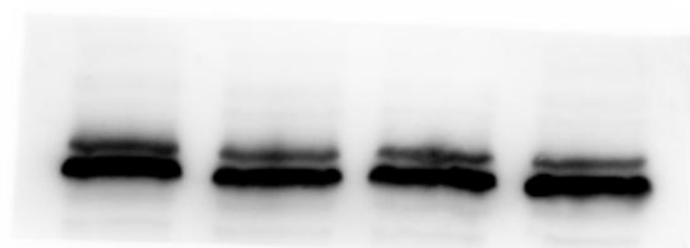

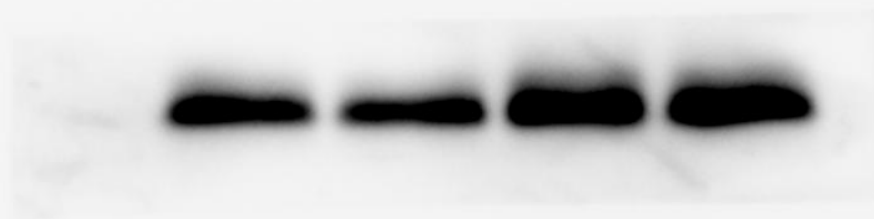

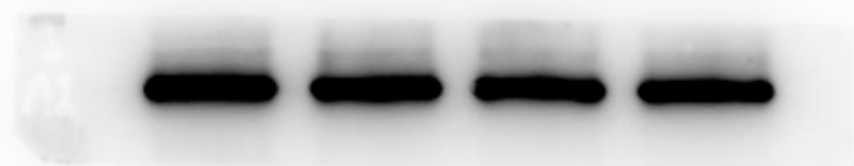

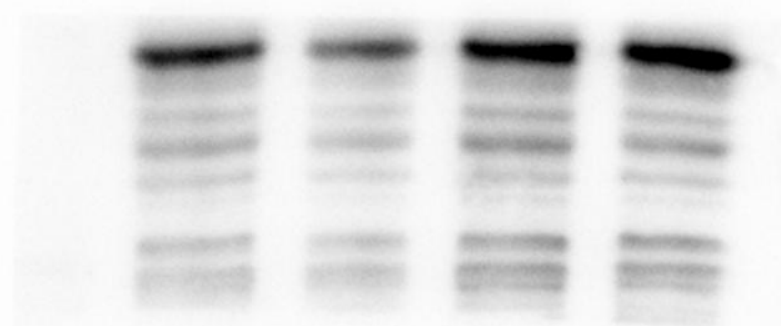

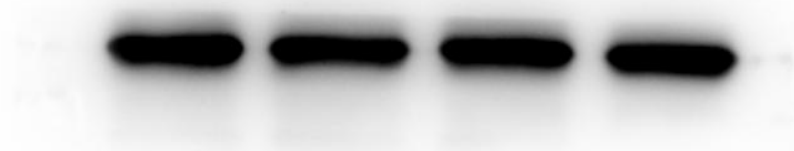

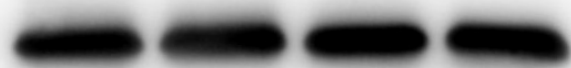

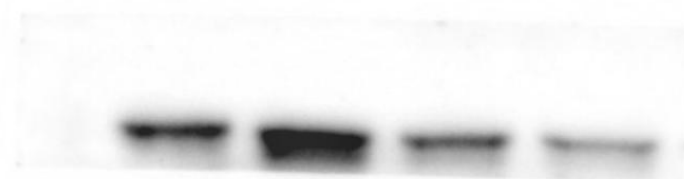

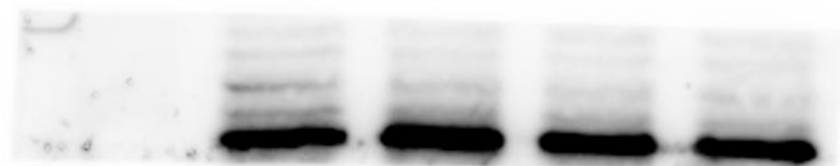

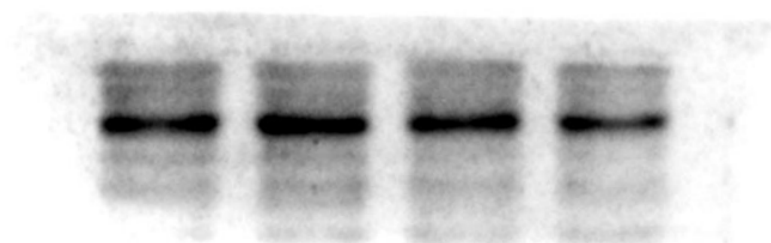

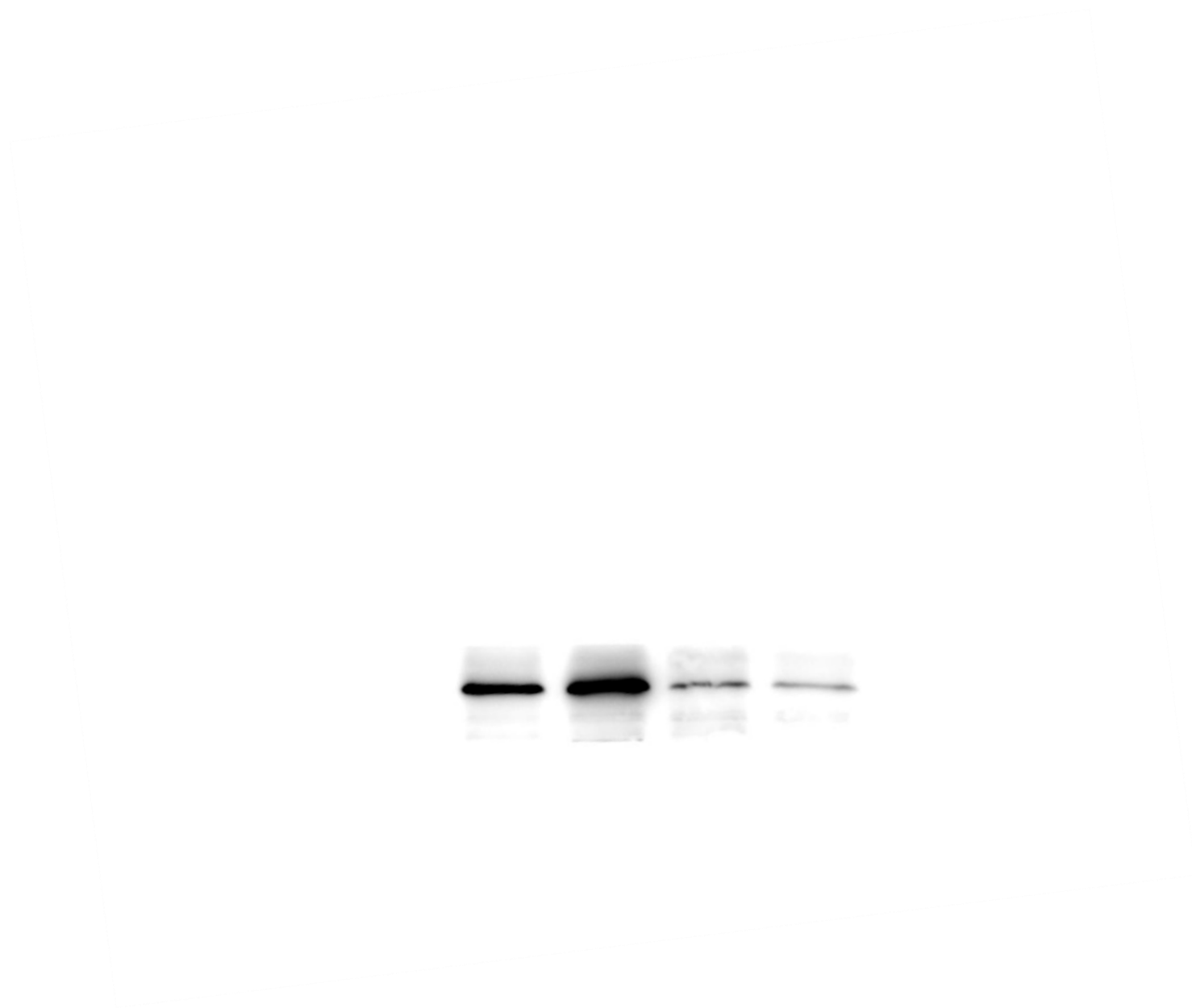

FIG6

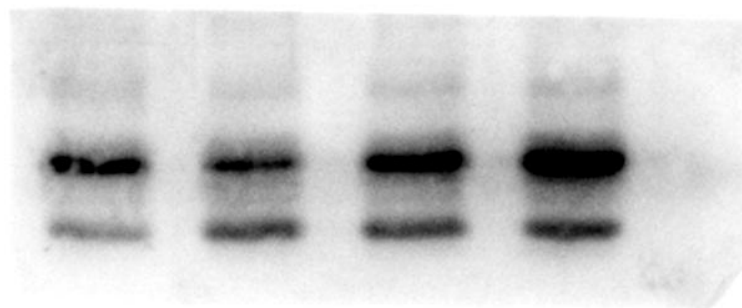

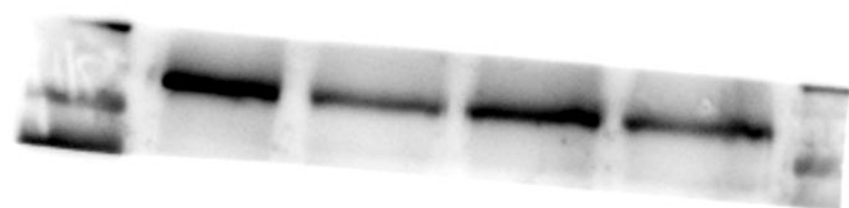

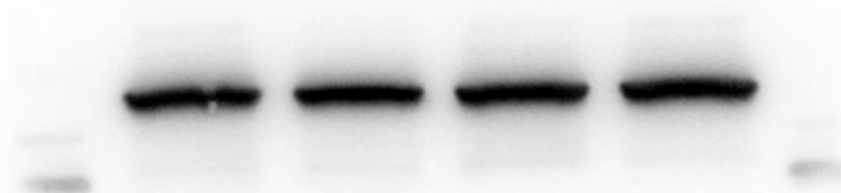

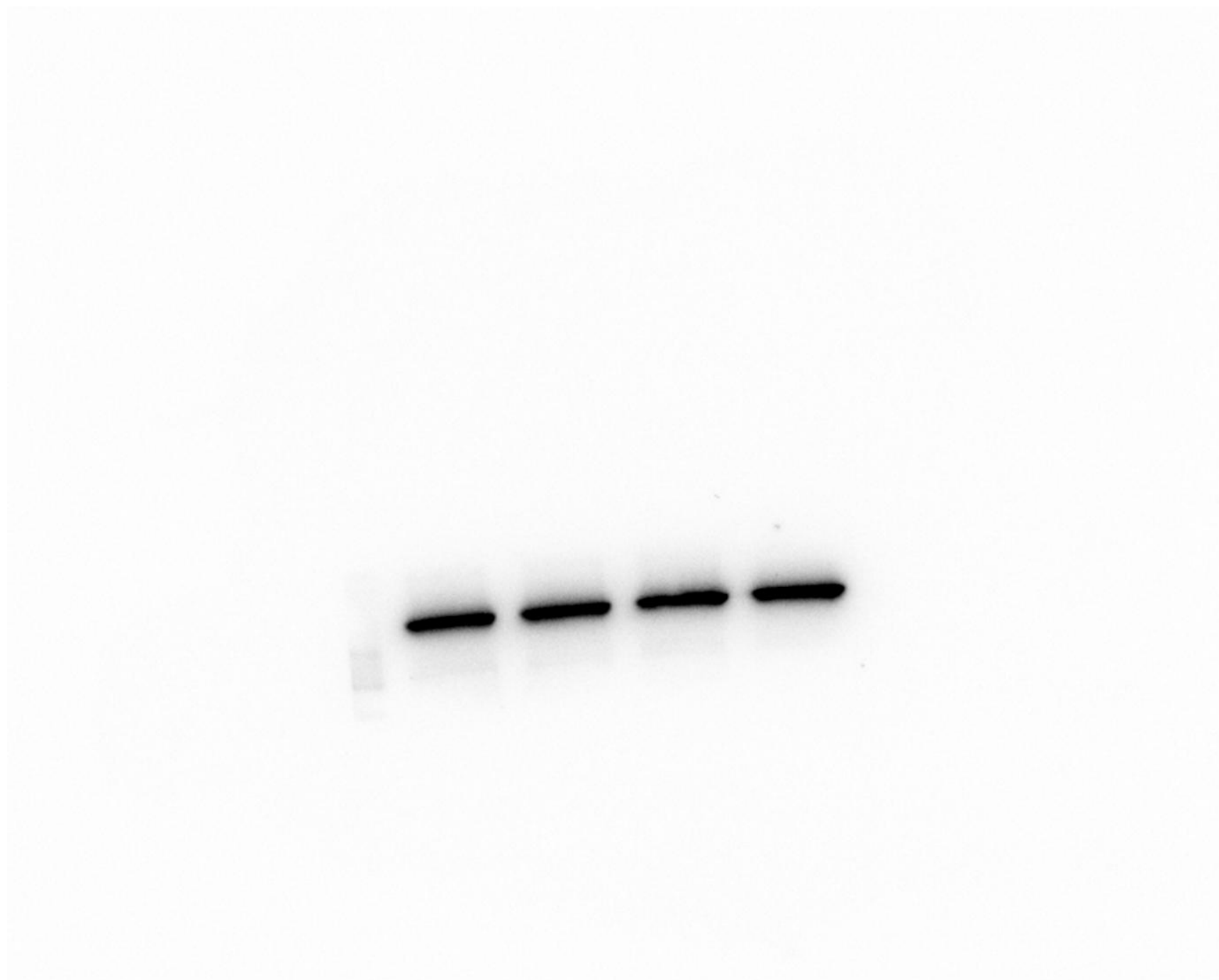

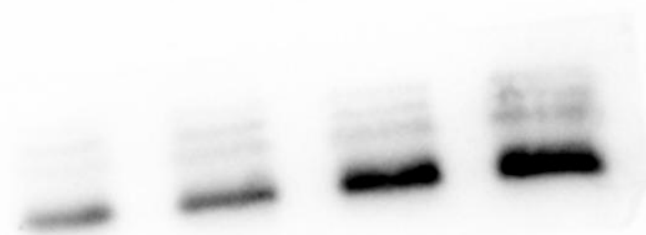

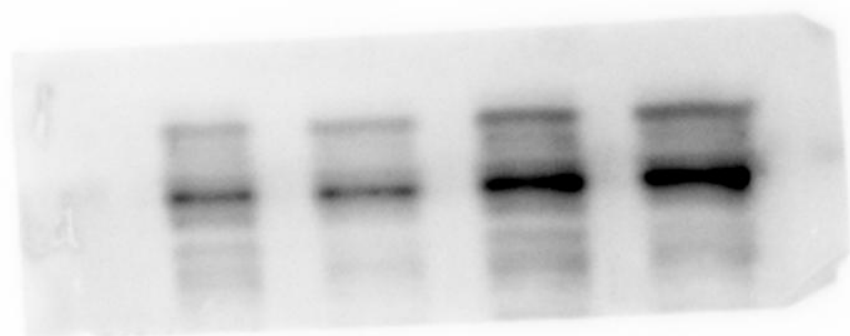

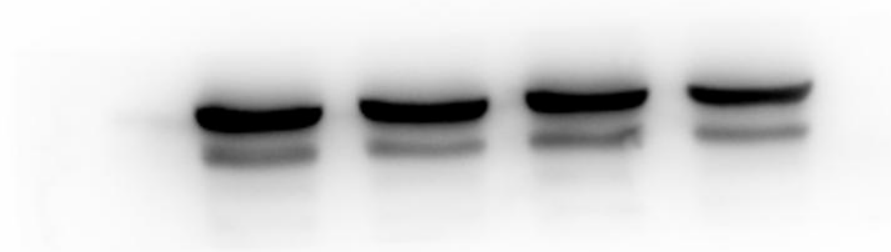

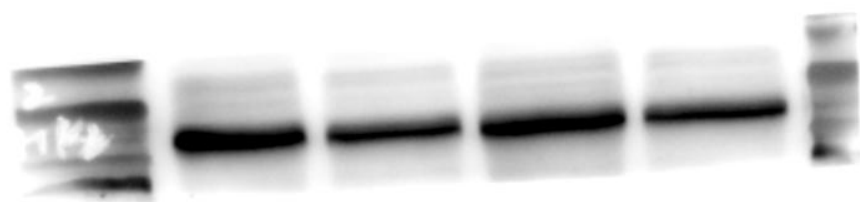

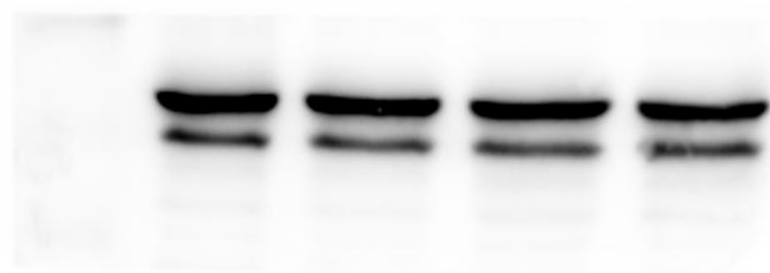

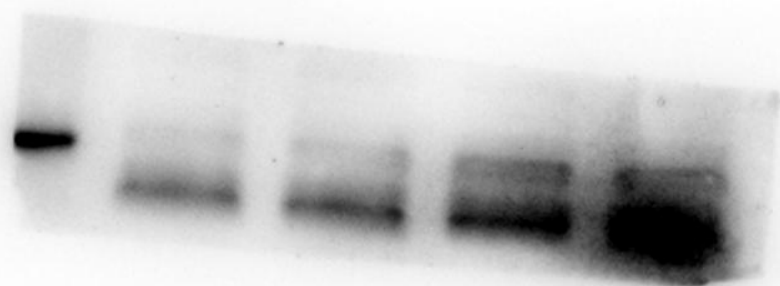

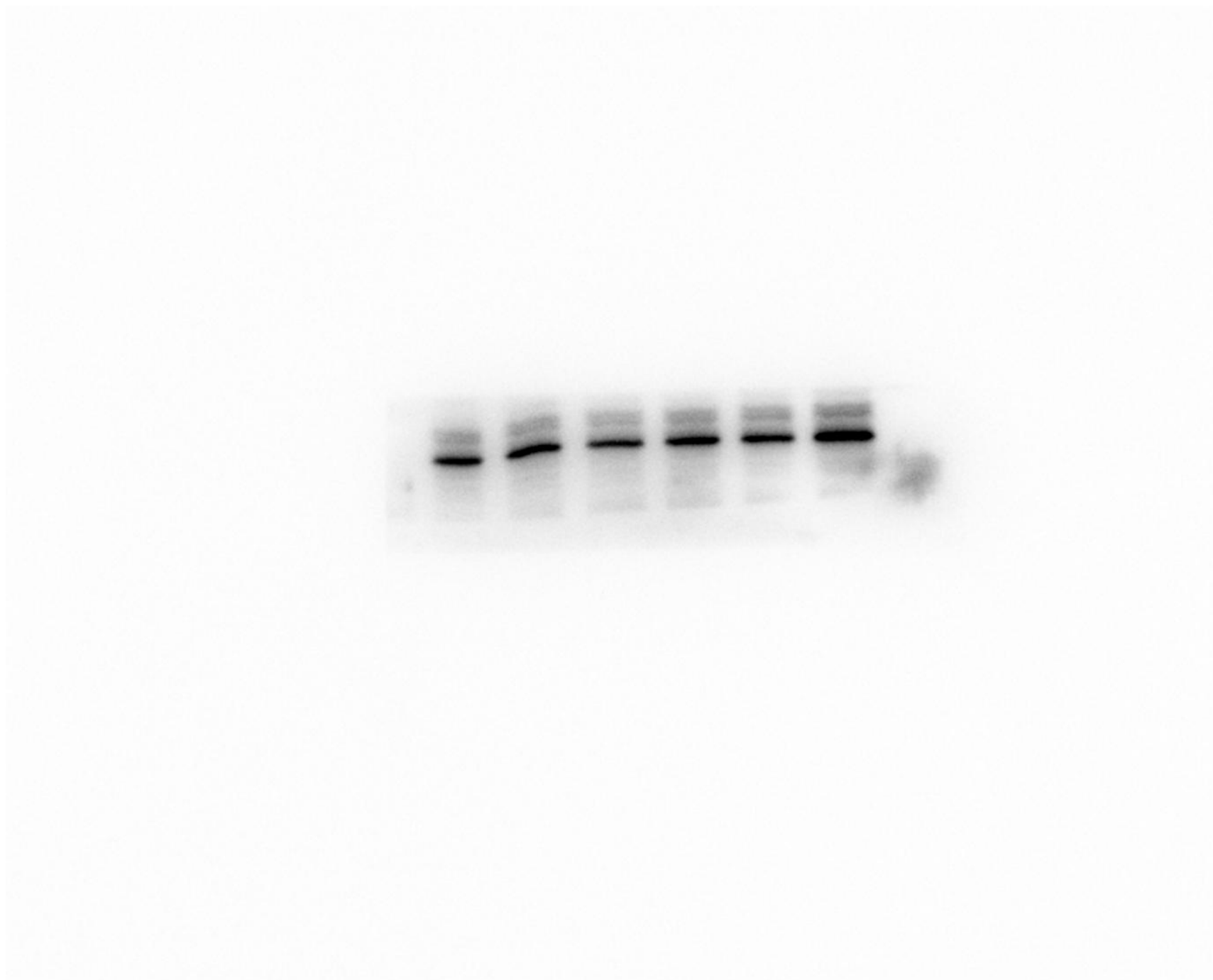

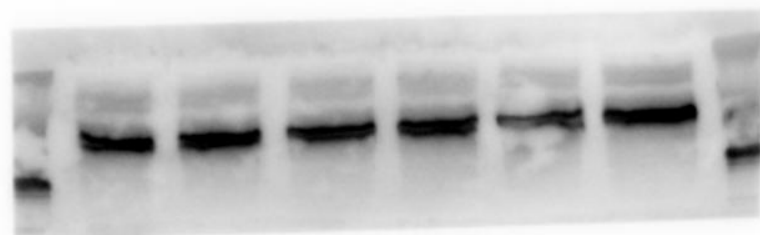

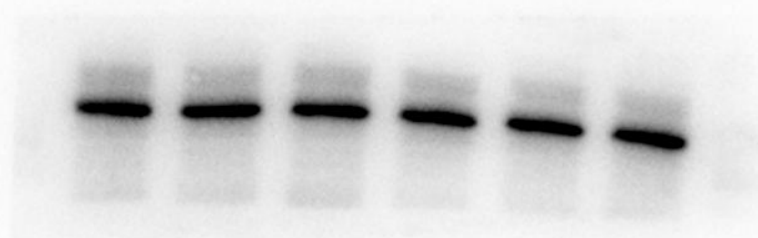

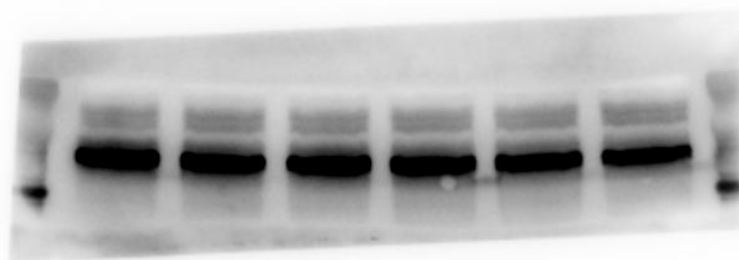

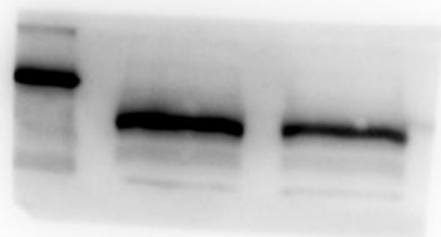

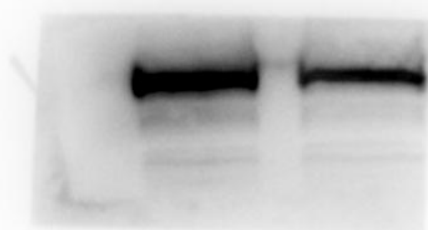

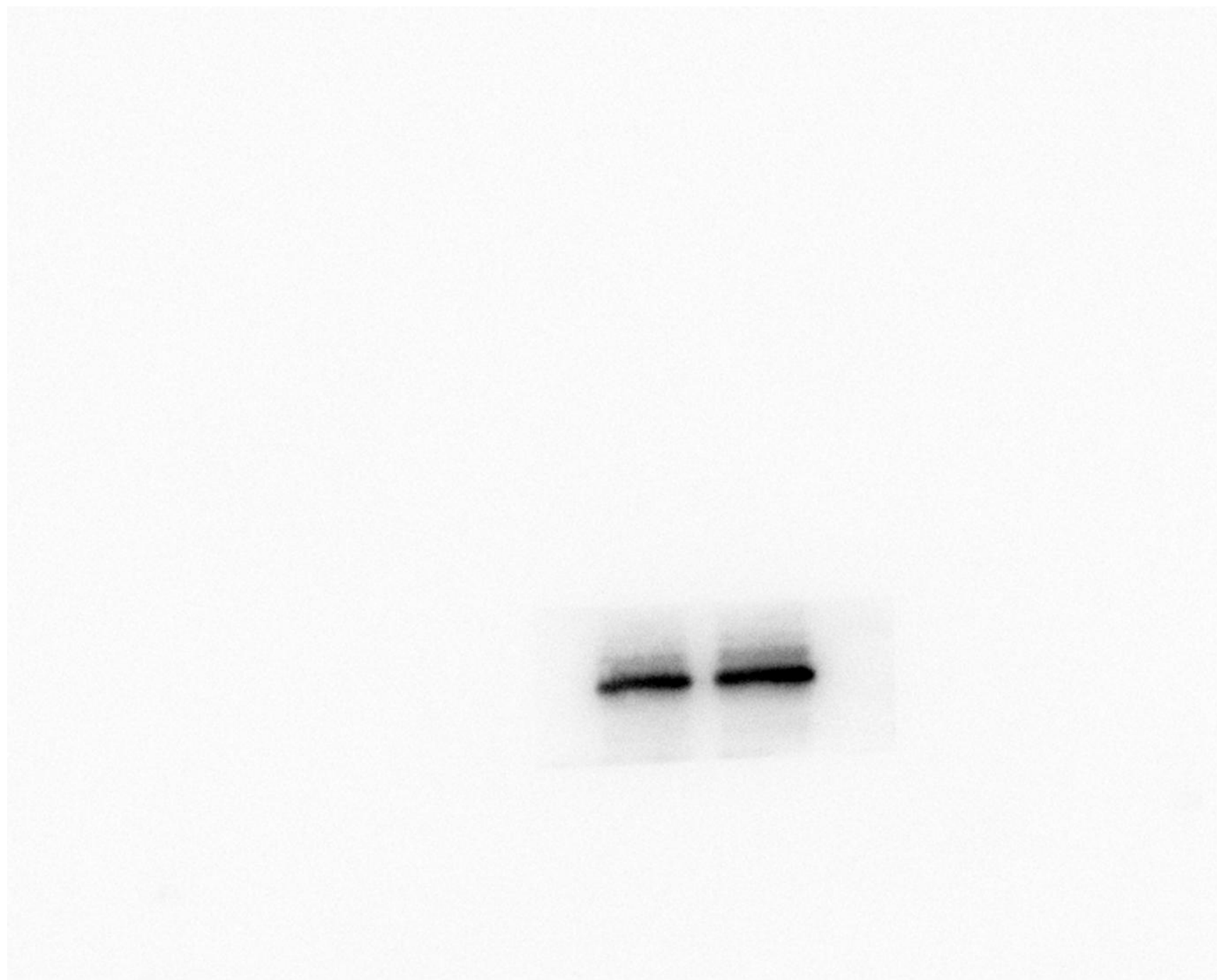

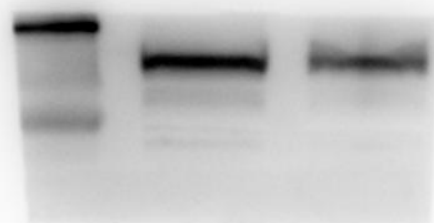

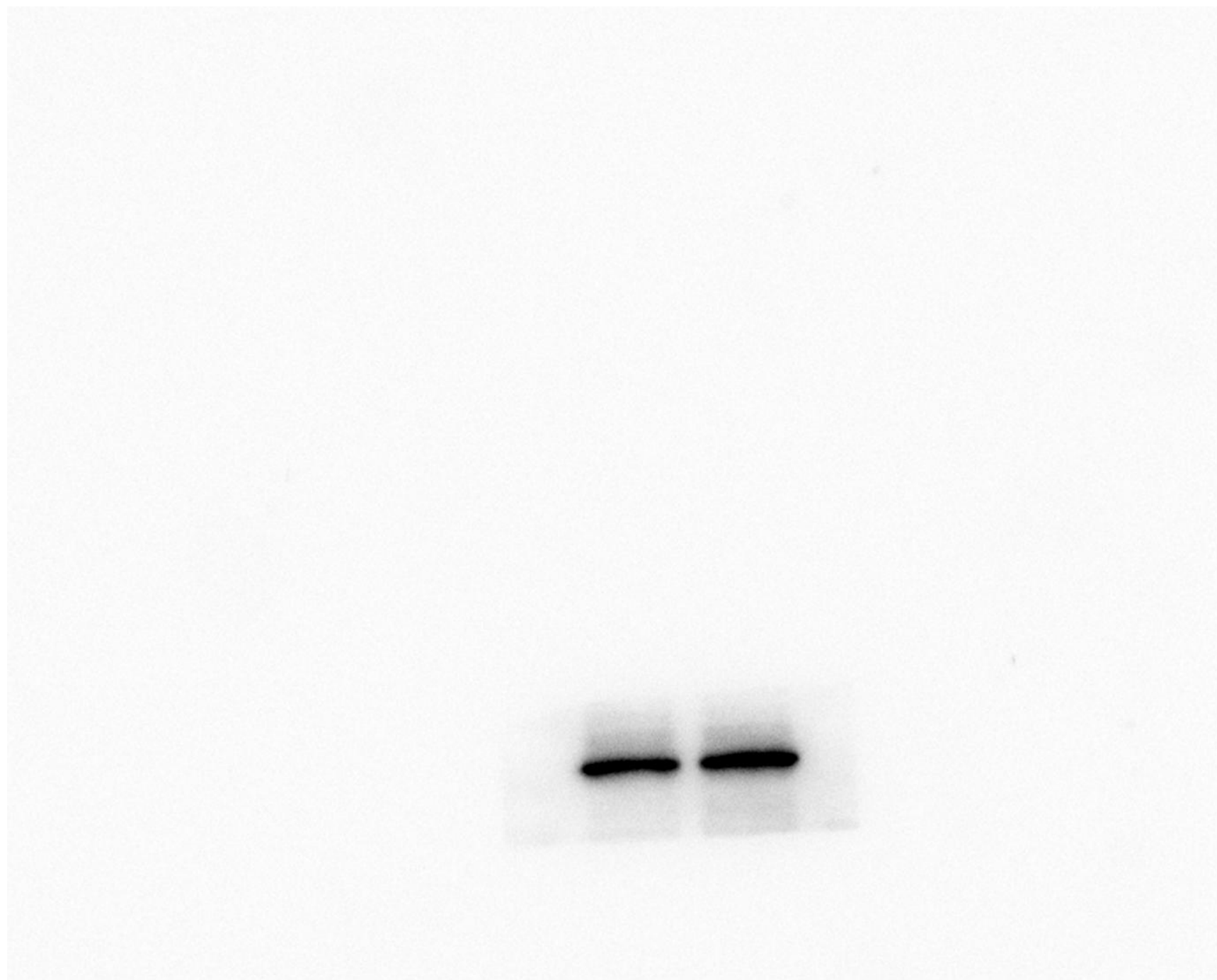

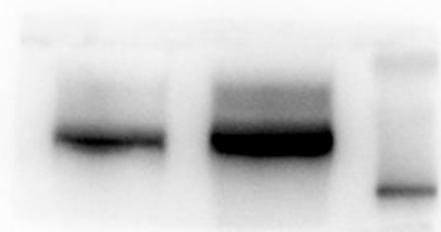

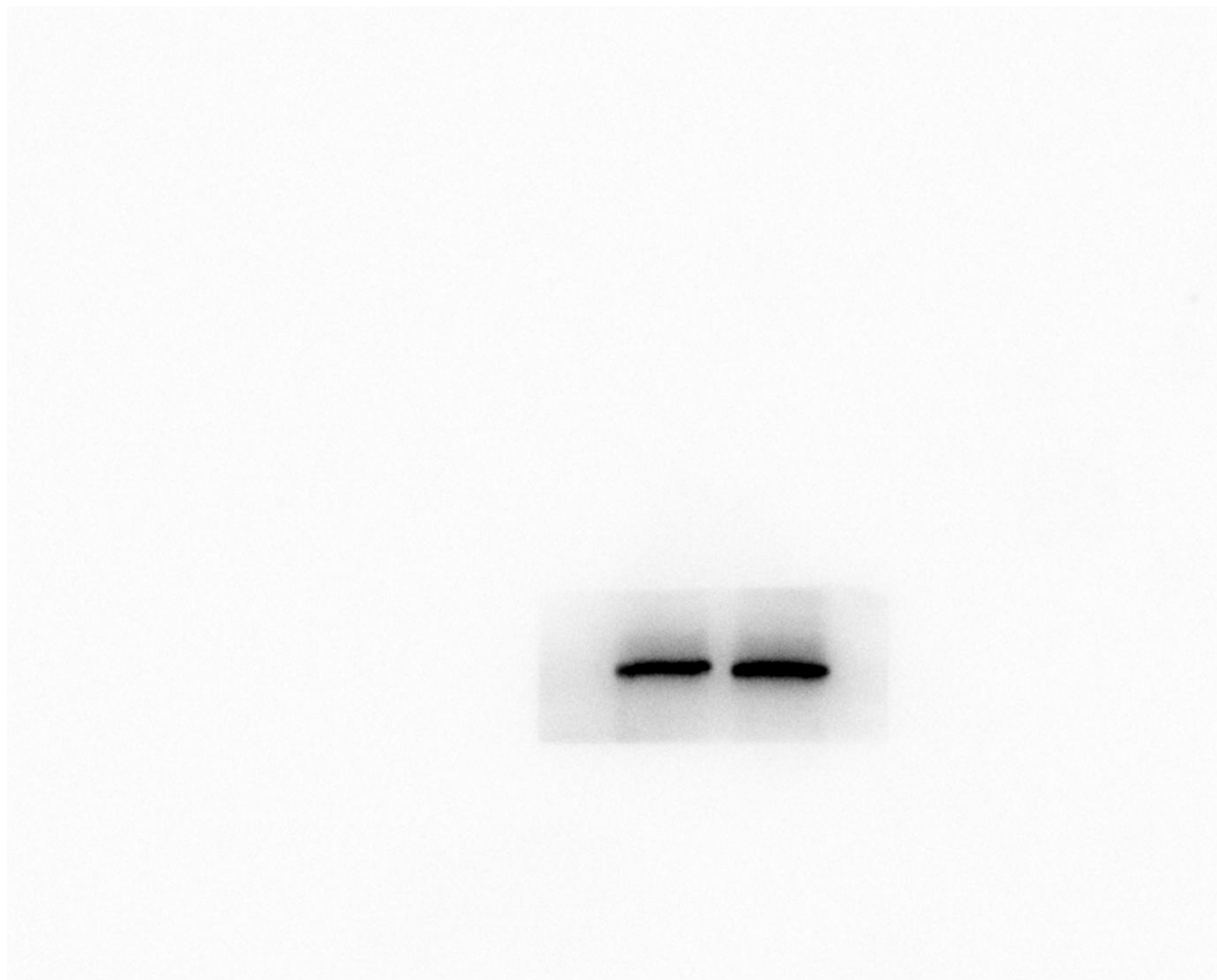

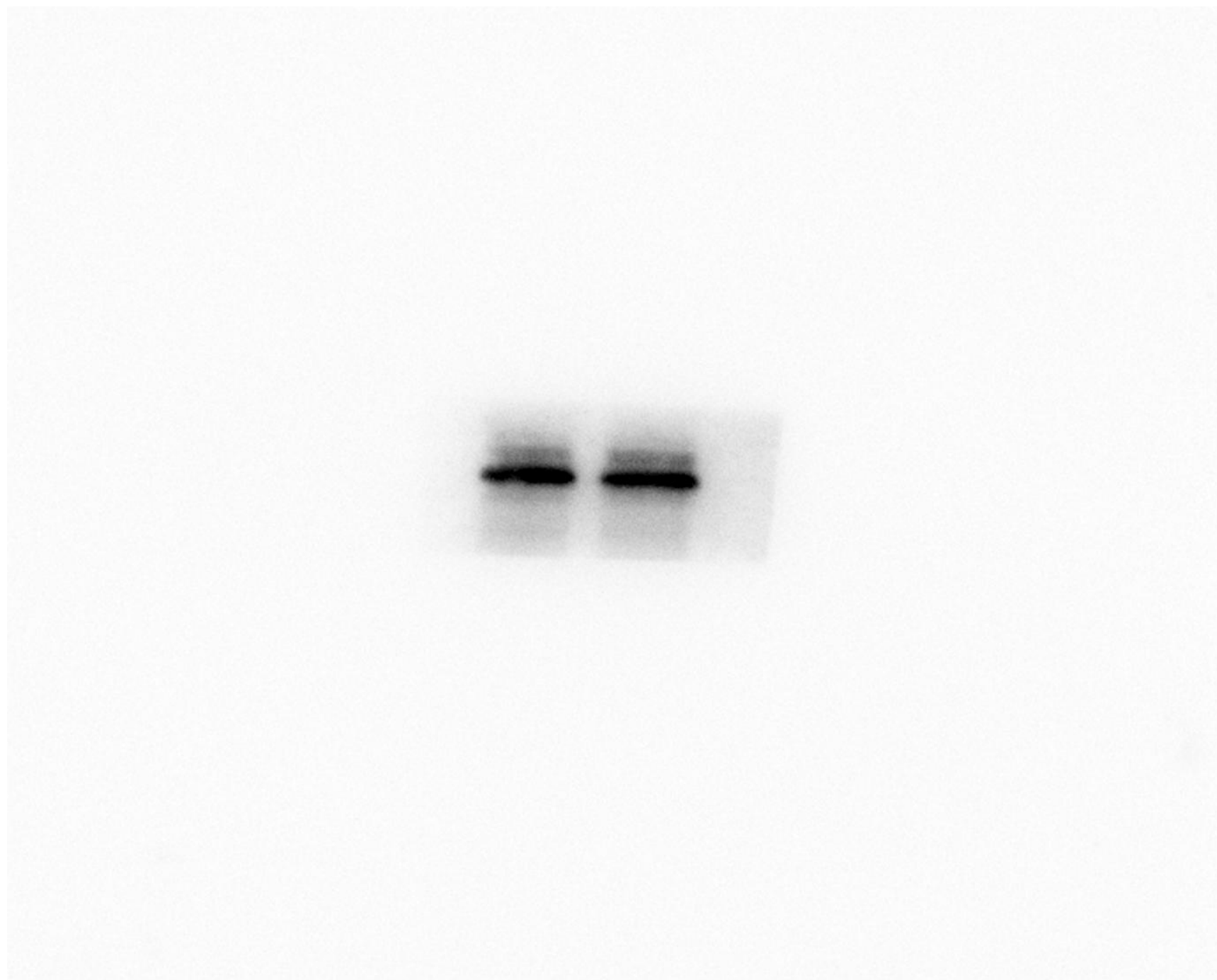

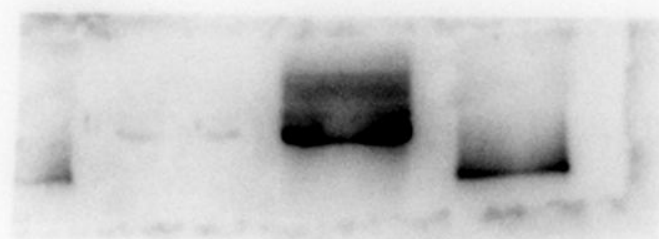

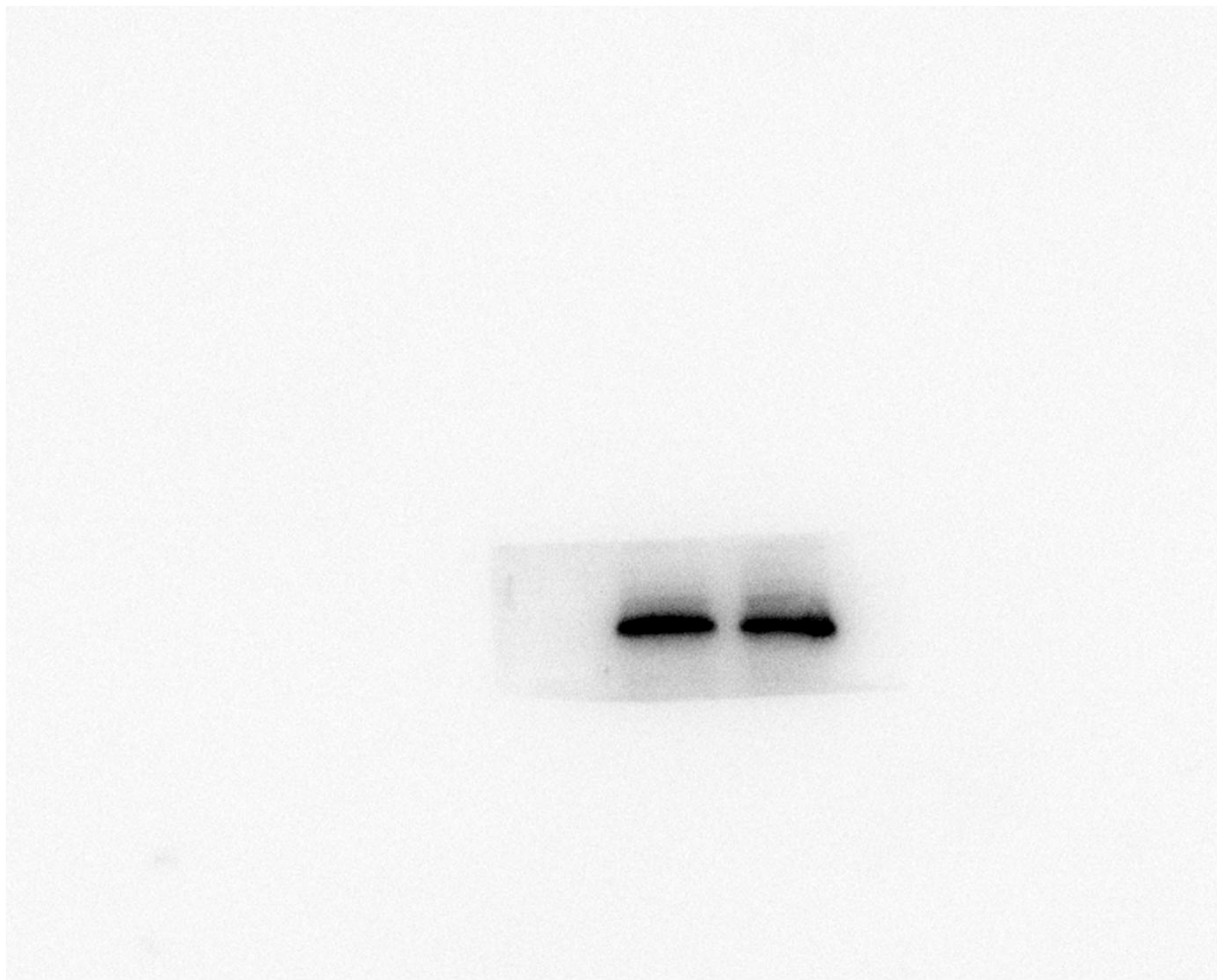

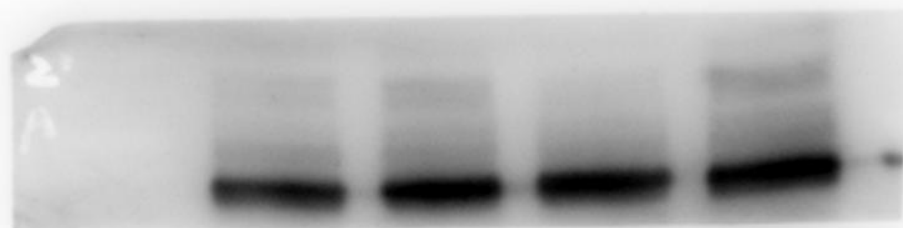

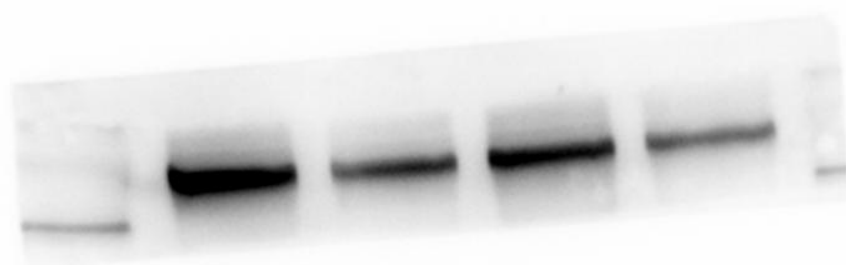

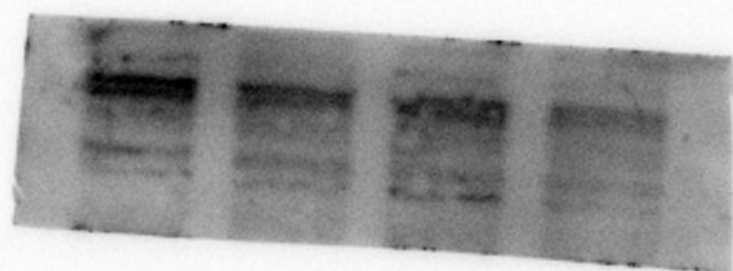

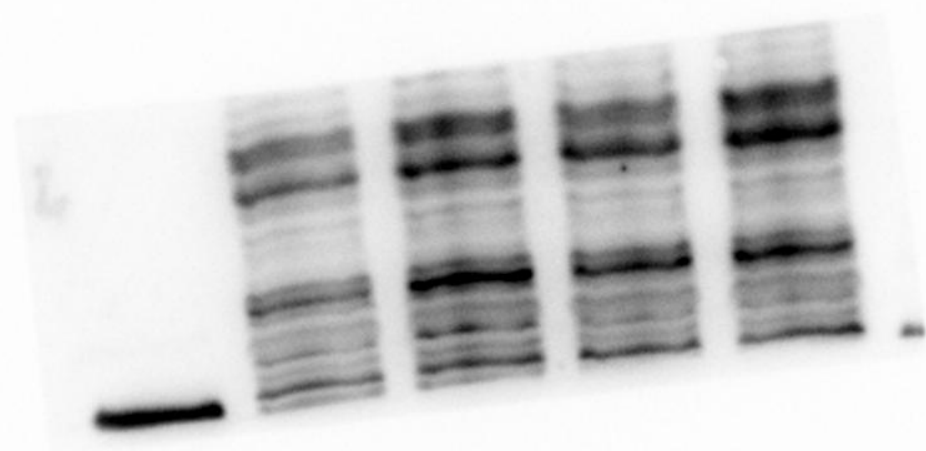

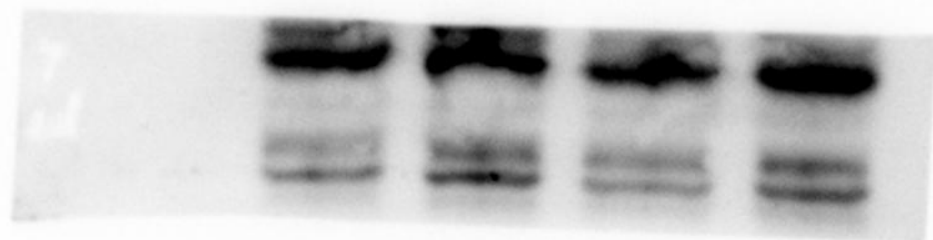

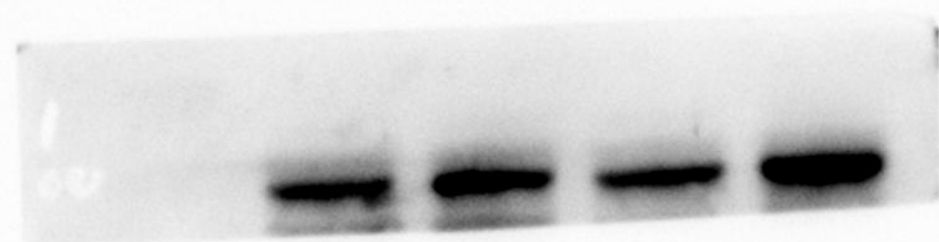

S. FIG3

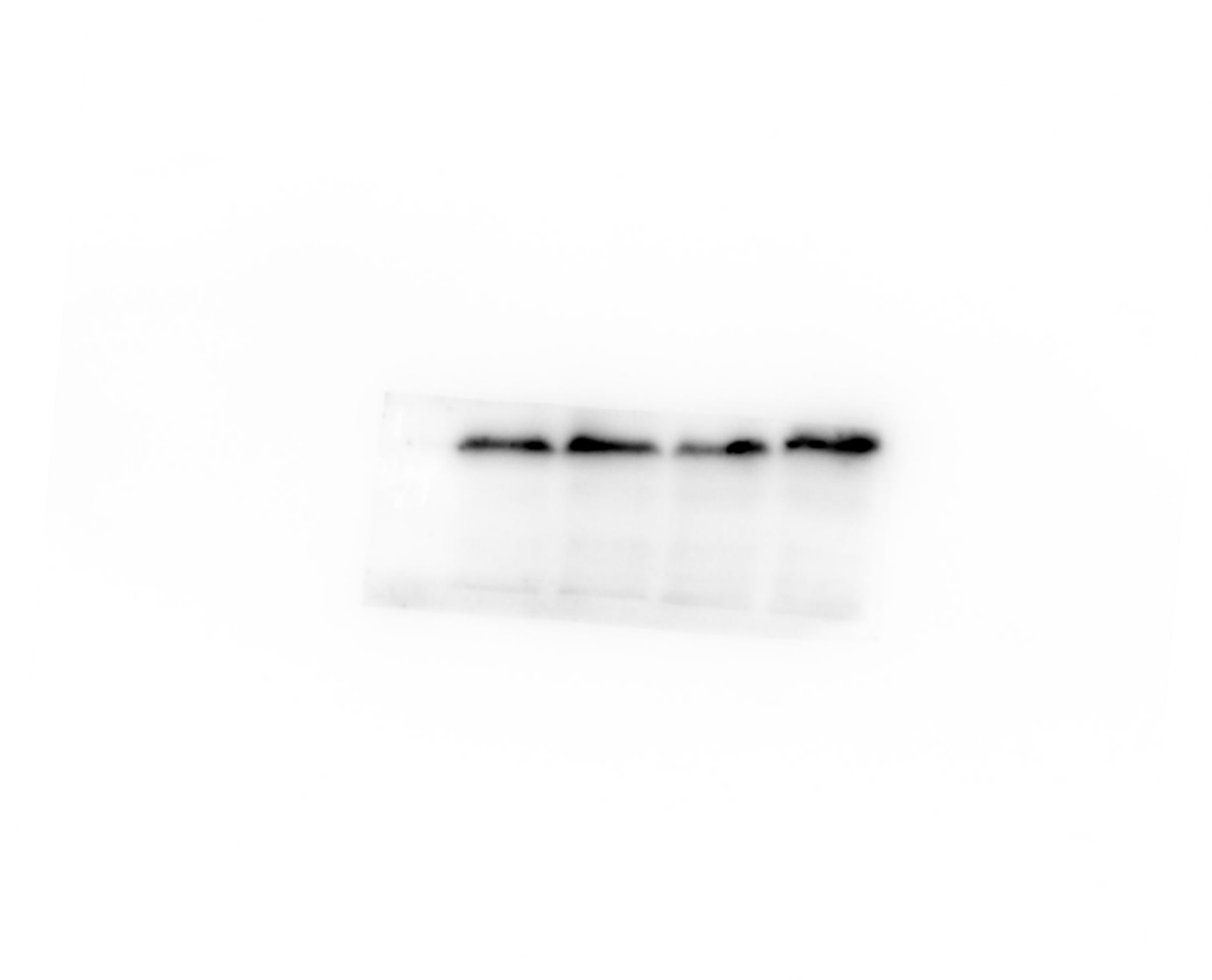

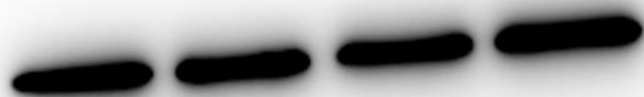

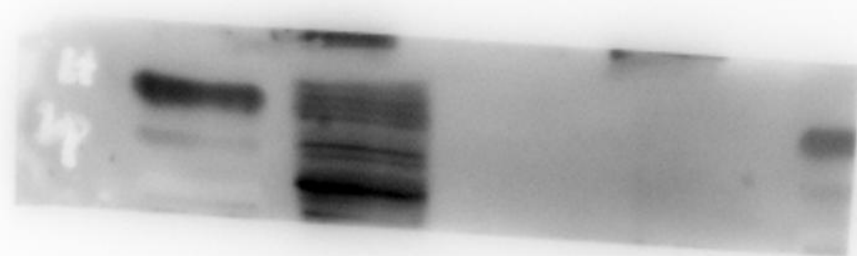

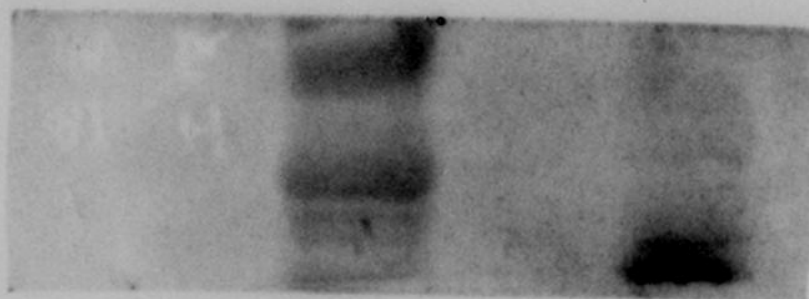

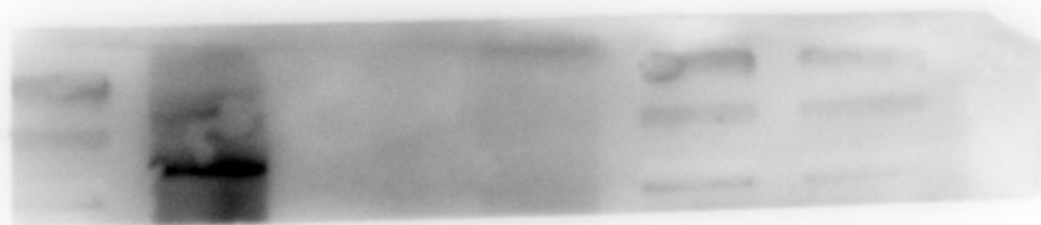

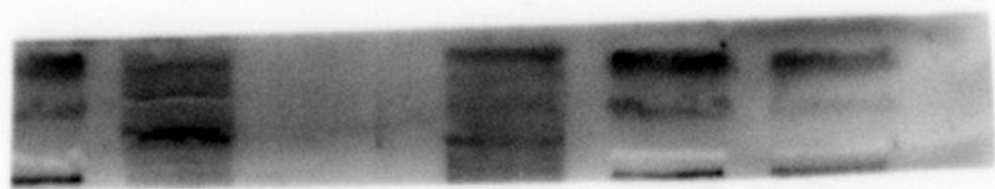

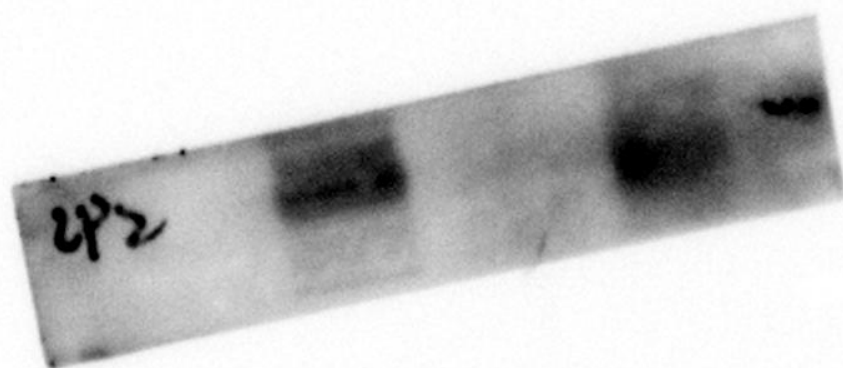

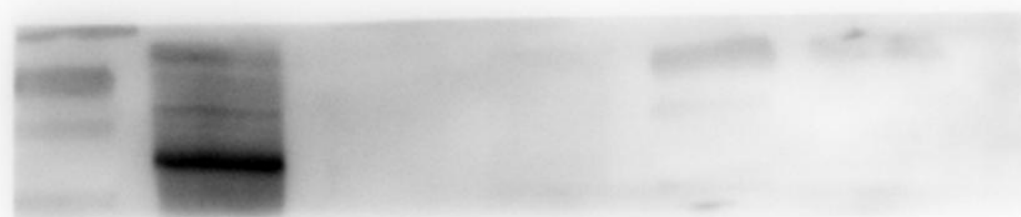

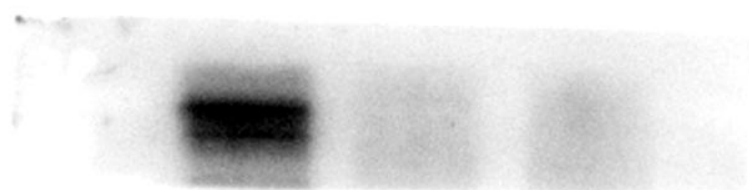

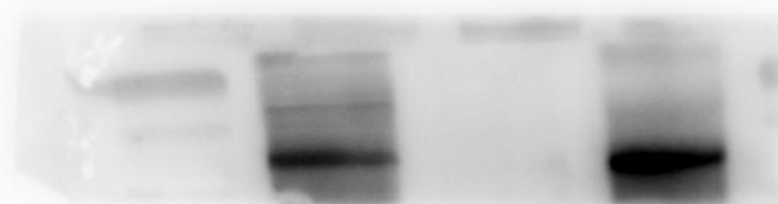

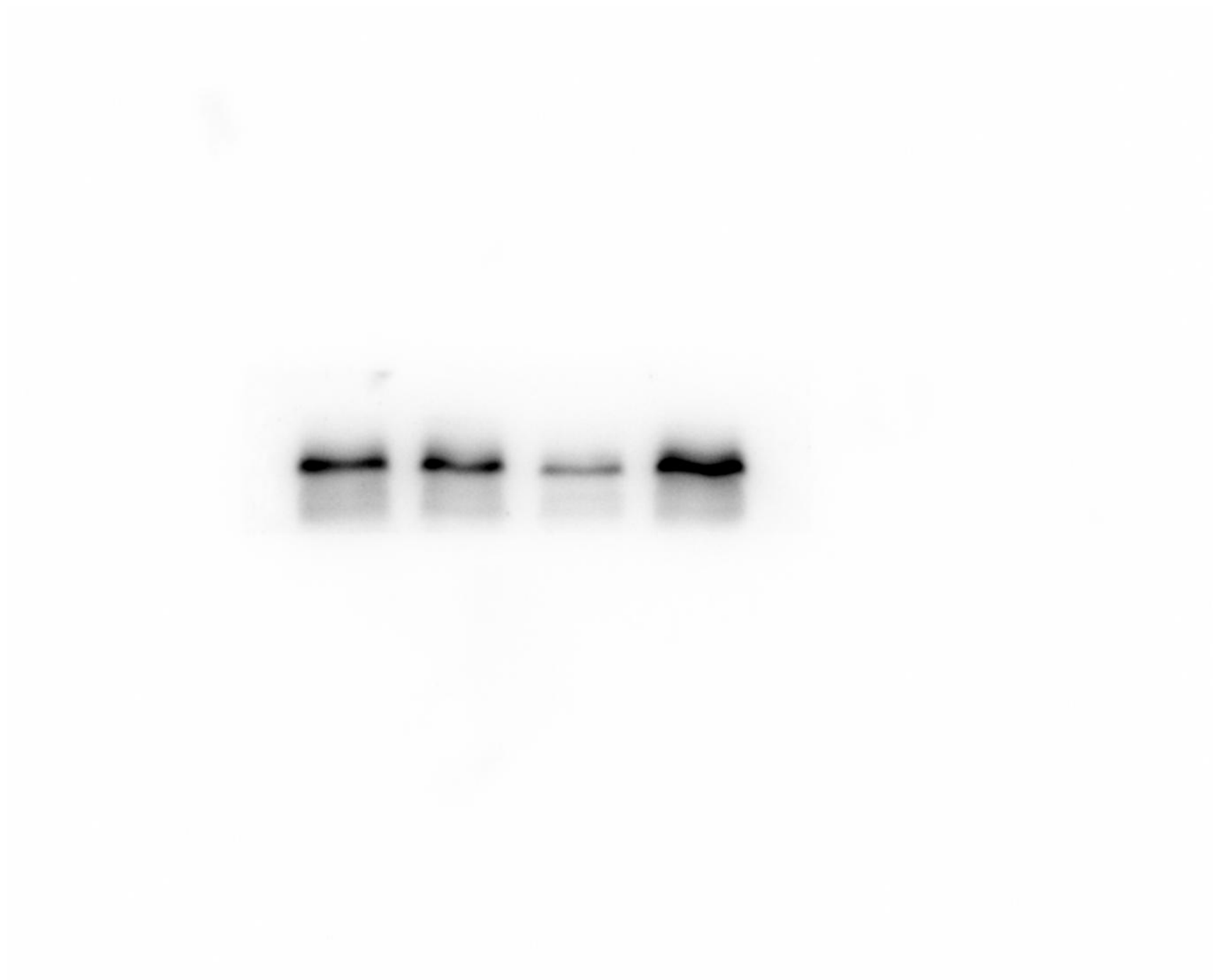

— — — —

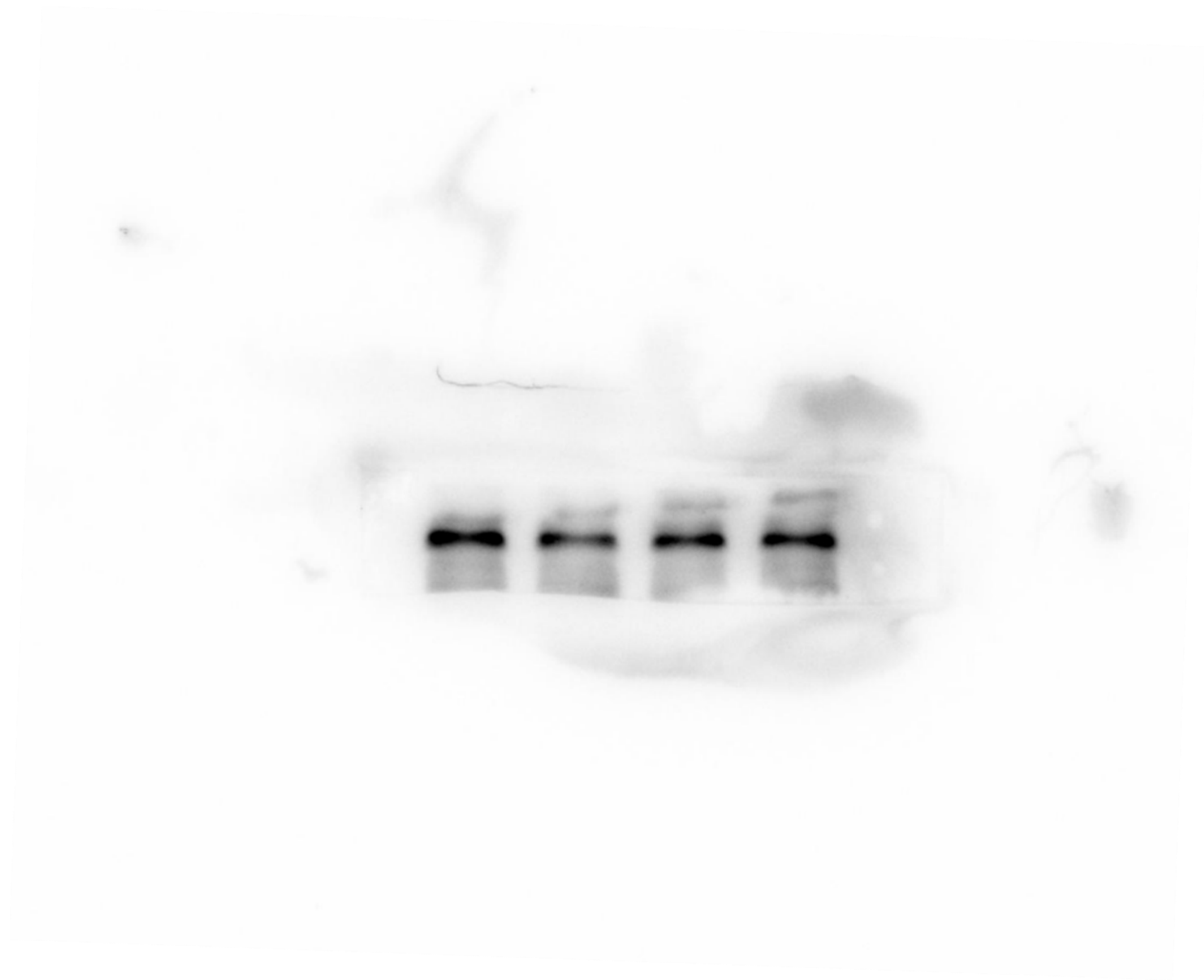

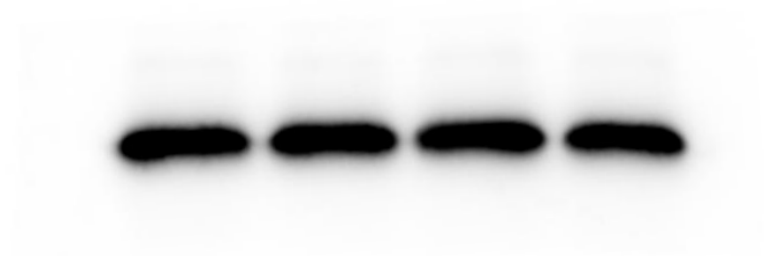

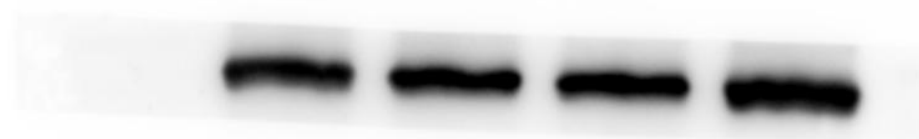

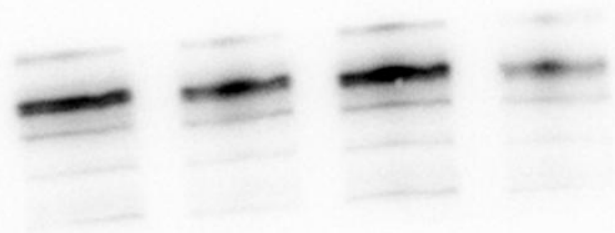

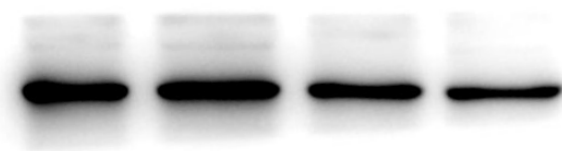

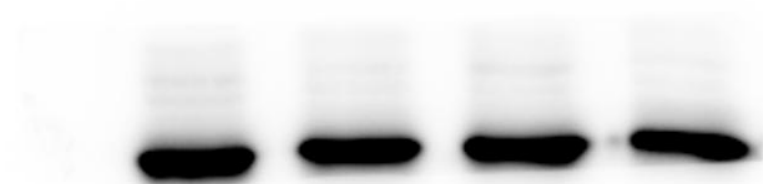

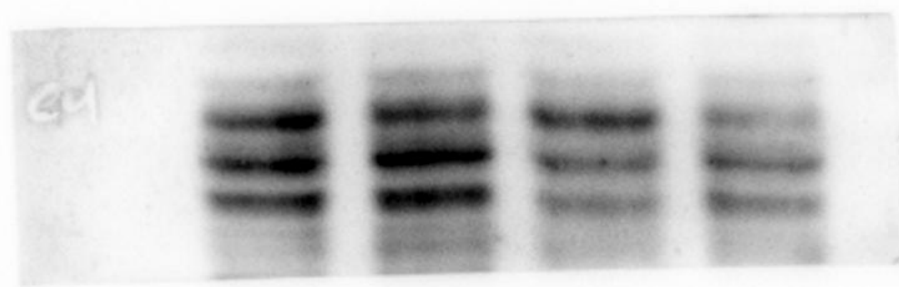

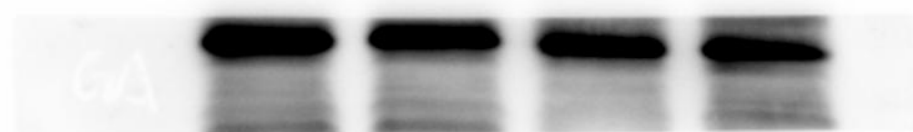

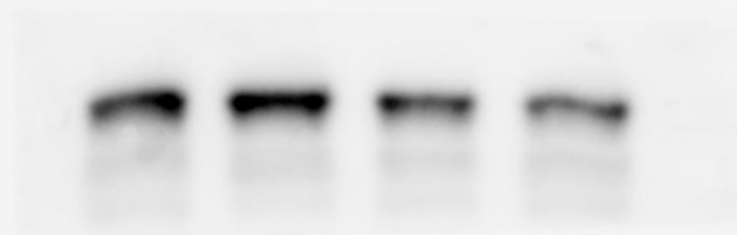

Supplement: Supplementary file 21 — Original Western Blots [file 41419_2023_6009_MOESM21_ESM.pdf]
