# Supplementary material for: Hexokinase 2 confers radio-resistance in hepatocellular carcinoma by promoting autophagy-dependent degradation of AIMP2
Source: Cell Death Dis. 2023 Aug 1;14(8):488. doi: 10.1038/s41419-023-06009-2 (PMC10390495; doi:10.1038/s41419-023-06009-2)
Supplement: Supplementary file 3 — Extended Figure3 (Figure3 merge file) [file 41419_2023_6009_MOESM3_ESM.pdf]

A

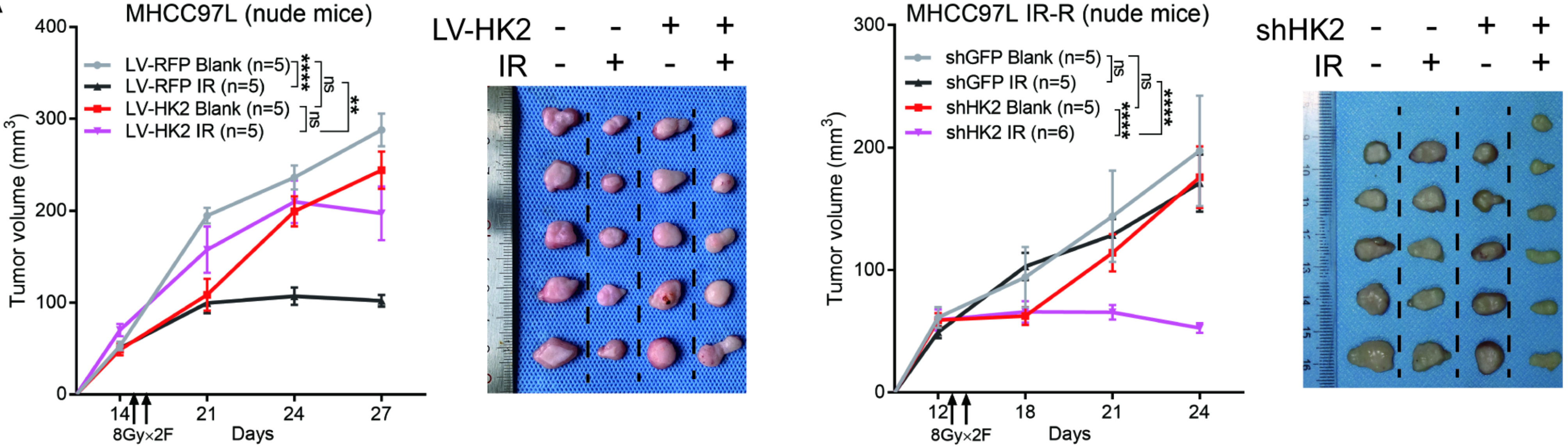
MHCC97L (nude mice)
Tumor volume (mm3)
400
300
200
100
0
LV-RFP Blank (n=5)
LV-RFP IR (n=5)
LV-HK2 Blank (n=5)
LV-HK2 IR (n=5)
****
ns
**
ns
14
21
24
27
8Gy×2F
Days
LV-HK2 - - + +
IR - + - +
MHCC97L IR-R (nude mice)
Tumor volume (mm3)
300
200
100
0
shGFP Blank (n=5)
shGFP IR (n=5)
shHK2 Blank (n=5)
shHK2 IR (n=6)
ns
ns
****
****
12
18
21
24
8Gy×2F
Days
shHK2 - - + +
IR - + - +

B

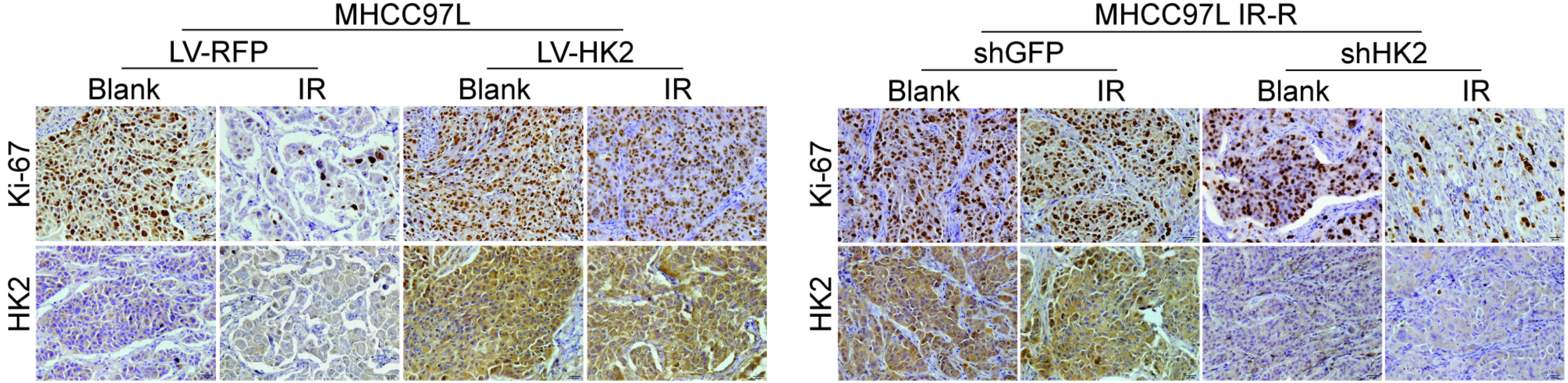

MHCC97L
LV-RFP
LV-HK2
Blank
IR
Blank
IR
Ki-67
HK2
MHCC97L IR-R
shGFP
shHK2
Blank
IR
Blank
IR
Ki-67
HK2

C

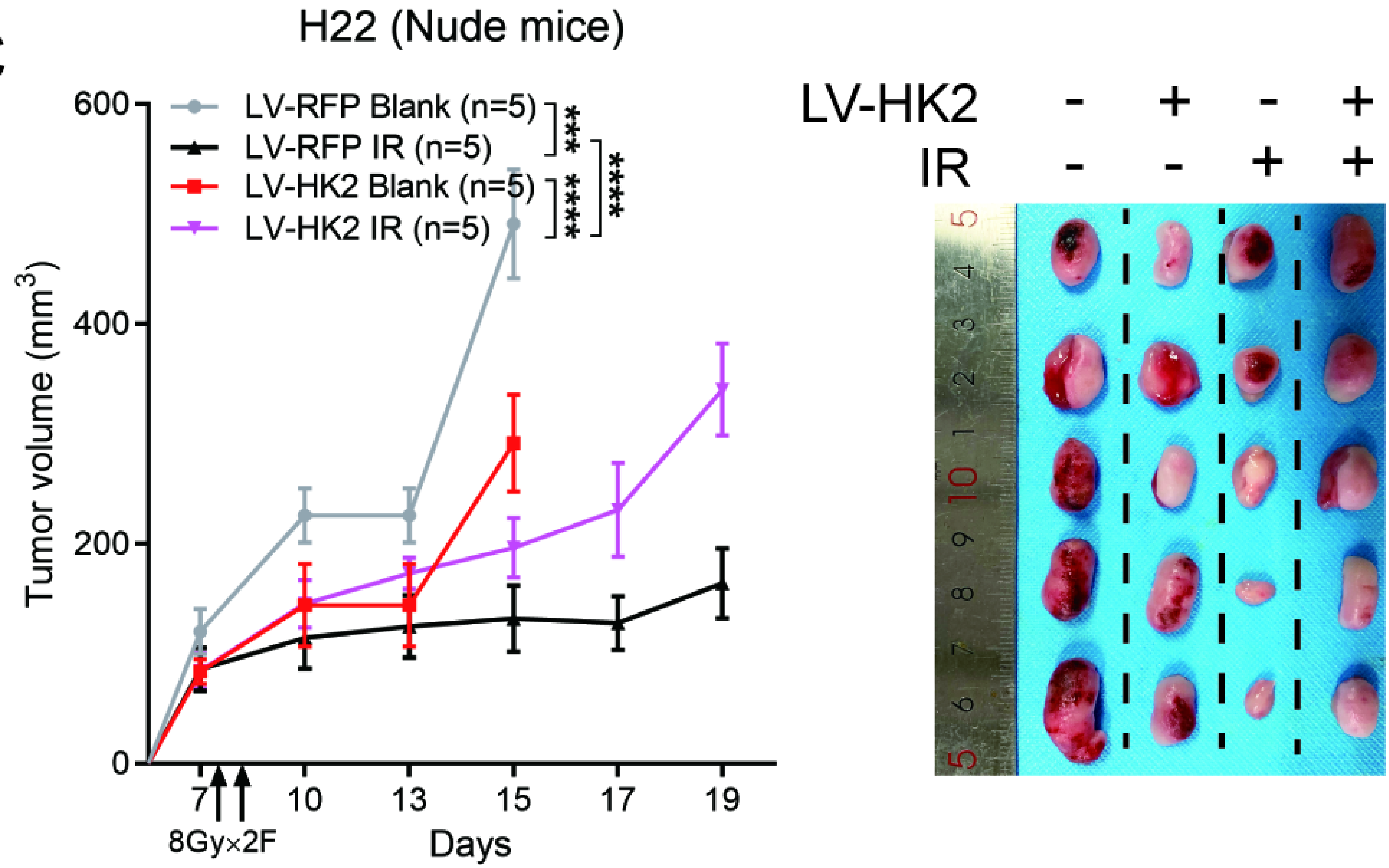

H22 (Nude mice)
LV-RFP Blank (n=5)
LV-RFP IR (n=5)
LV-HK2 Blank (n=5)
LV-HK2 IR (n=5)
***
****
****
Tumor volume (mm3)
600
400
200
0
7
10
13
15
17
19
8Gy×2F
Days
LV-HK2 - + - +
IR - - + +

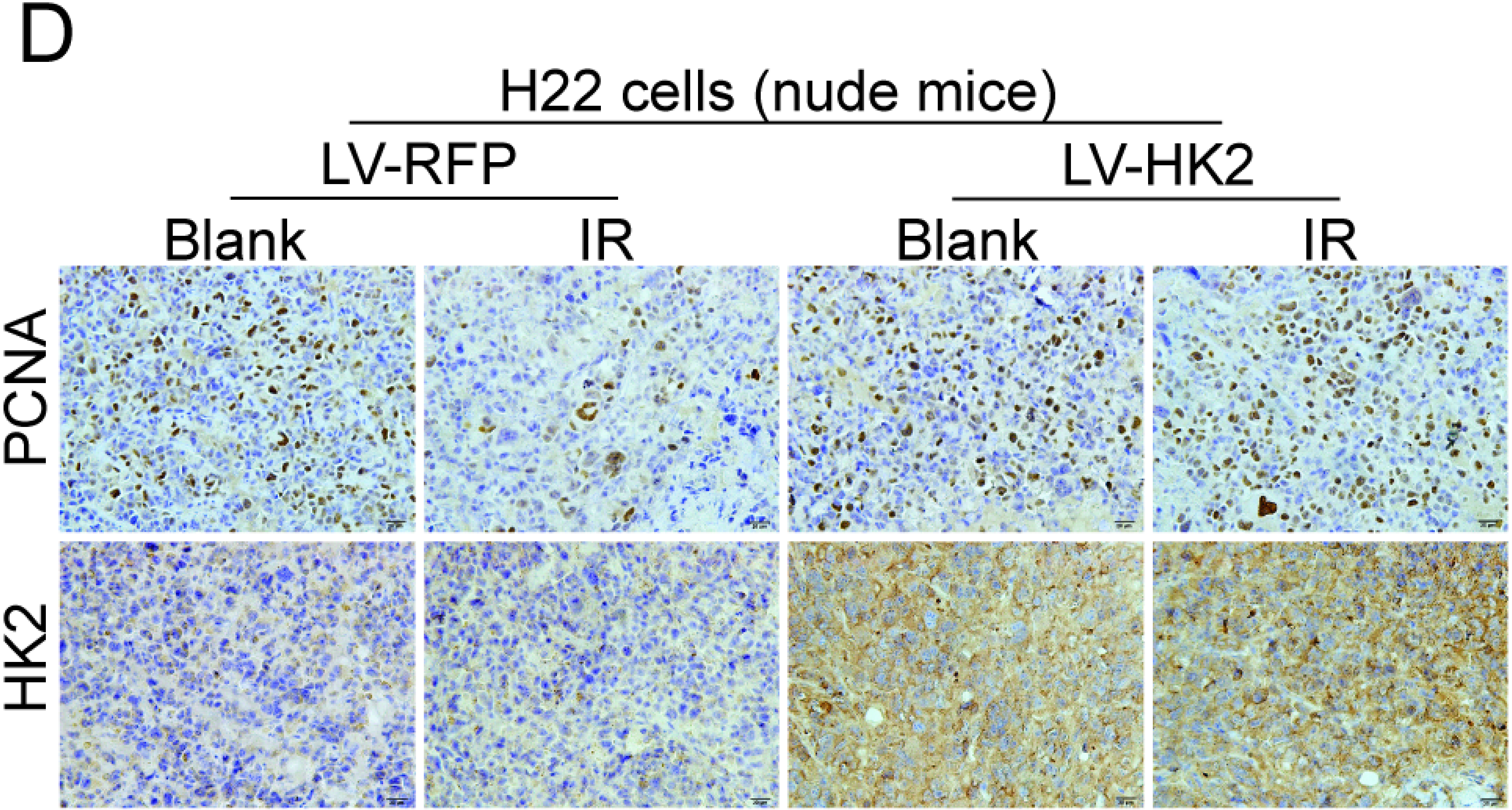

D
H22 cells (nude mice)
LV-RFP
LV-HK2
Blank
IR
Blank
IR
PCNA
HK2

E

H22 (C57BL/6 mice)

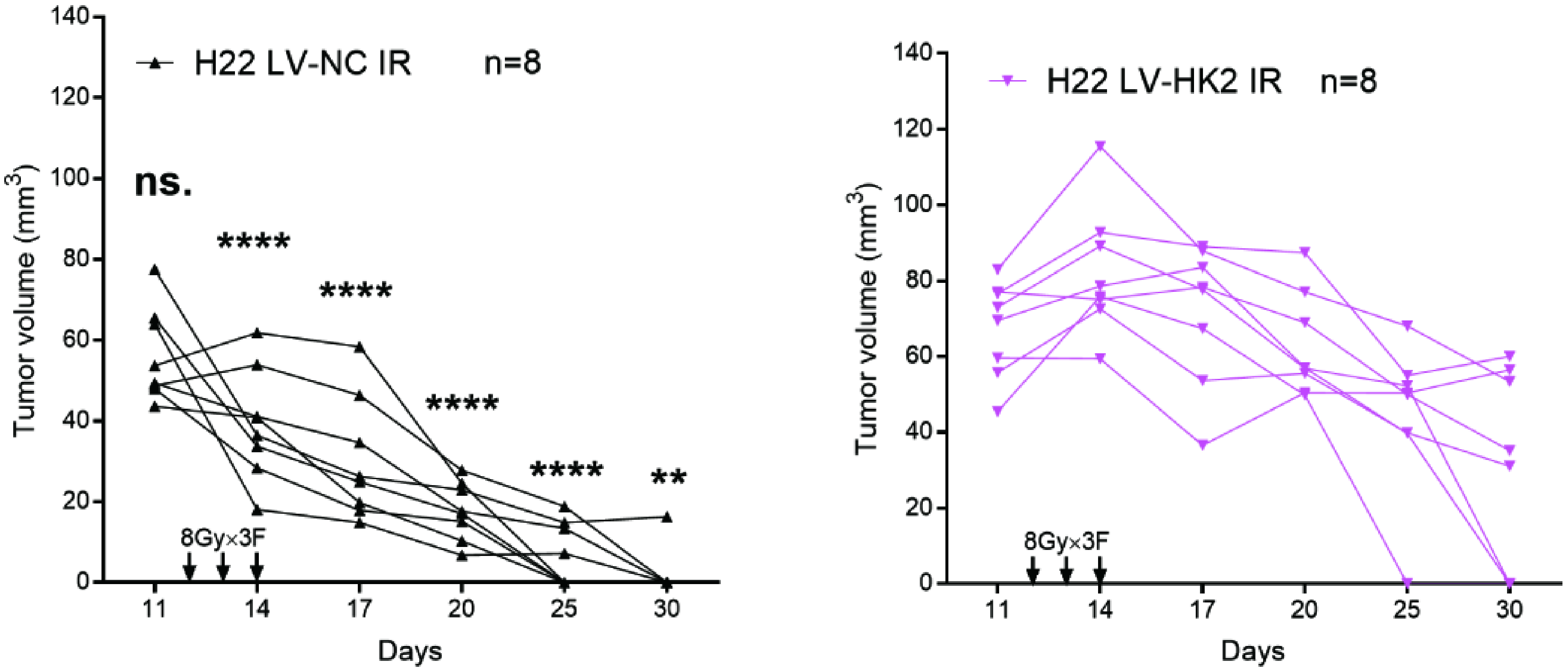

F

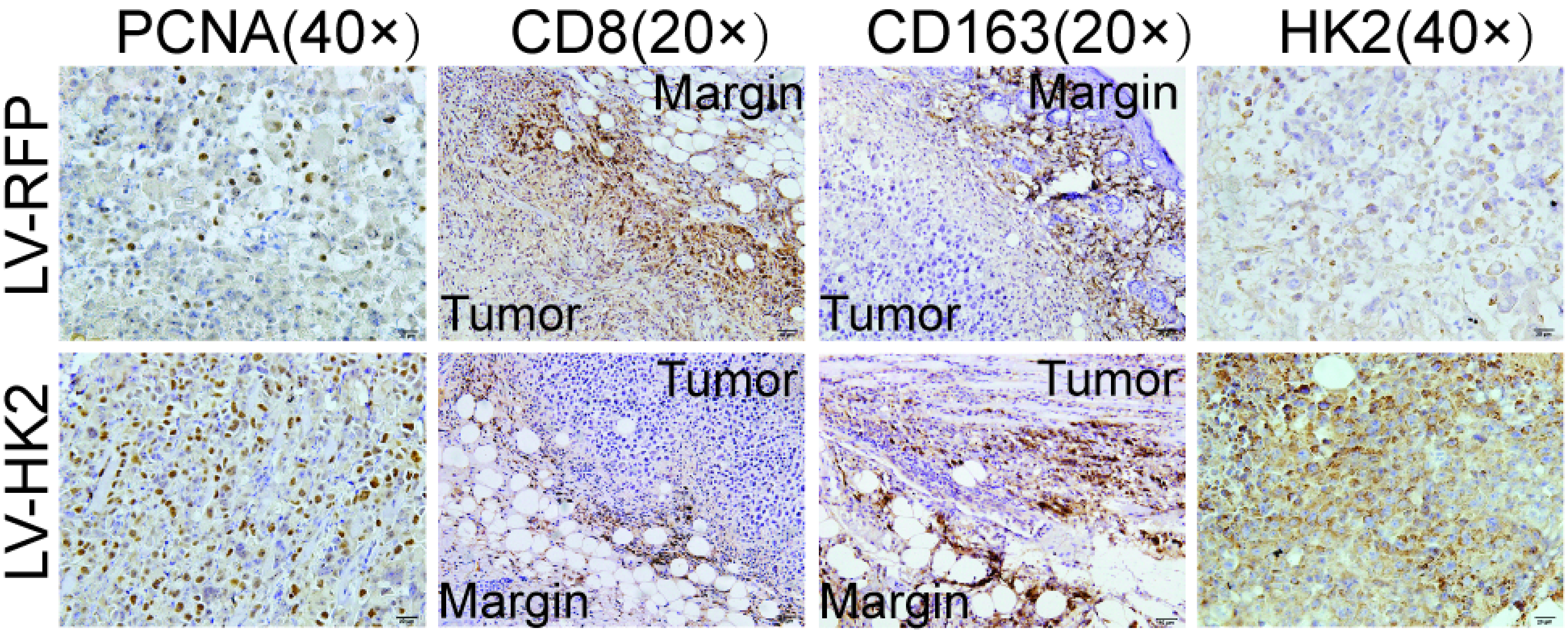
PCNA(40×)
CD8(20×)
CD163(20×)
HK2(40×)
LV-RFP
LV-HK2
Margin
Tumor
Tumor
Margin
Margin
Tumor
Tumor
Margin
