# Supplementary material for: Hexokinase 2 confers radio-resistance in hepatocellular carcinoma by promoting autophagy-dependent degradation of AIMP2
Source: Cell Death Dis. 2023 Aug 1;14(8):488. doi: 10.1038/s41419-023-06009-2 (PMC10390495; doi:10.1038/s41419-023-06009-2)
Supplement: Supplementary file 6 — Extended Figure6 (Figure6 merge file) [file 41419_2023_6009_MOESM6_ESM.pdf]

A

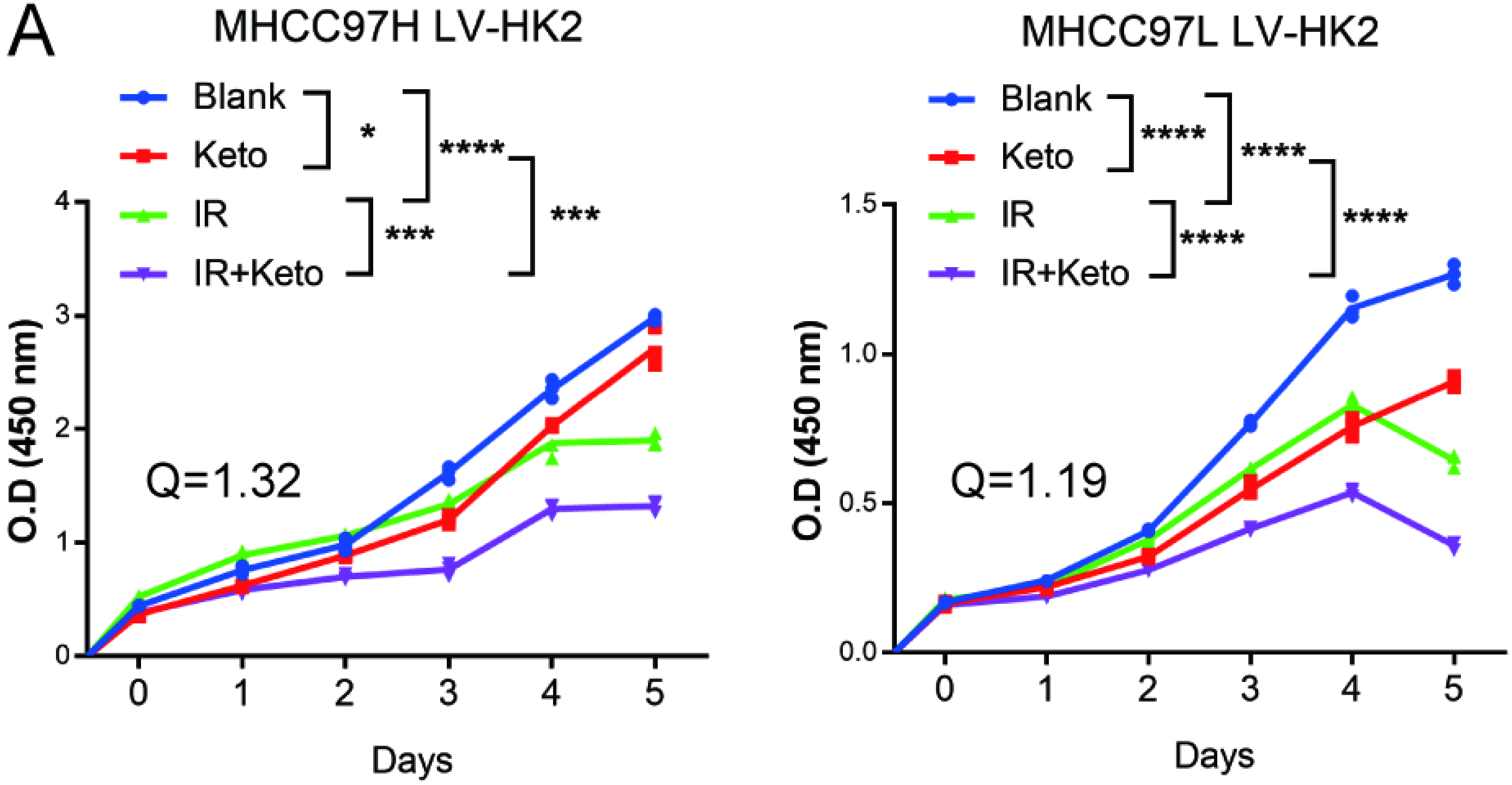

MHCC97H LV-HK2
Blank
Keto
IR
IR+Keto
*
****
***
***
Q=1.32
O.D (450 nm)
Days
0
1
2
3
4
5
MHCC97L LV-HK2
Blank
Keto
IR
IR+Keto
****
****
****
****
Q=1.19
O.D (450 nm)
Days
0.0
0.5
1.0
1.5

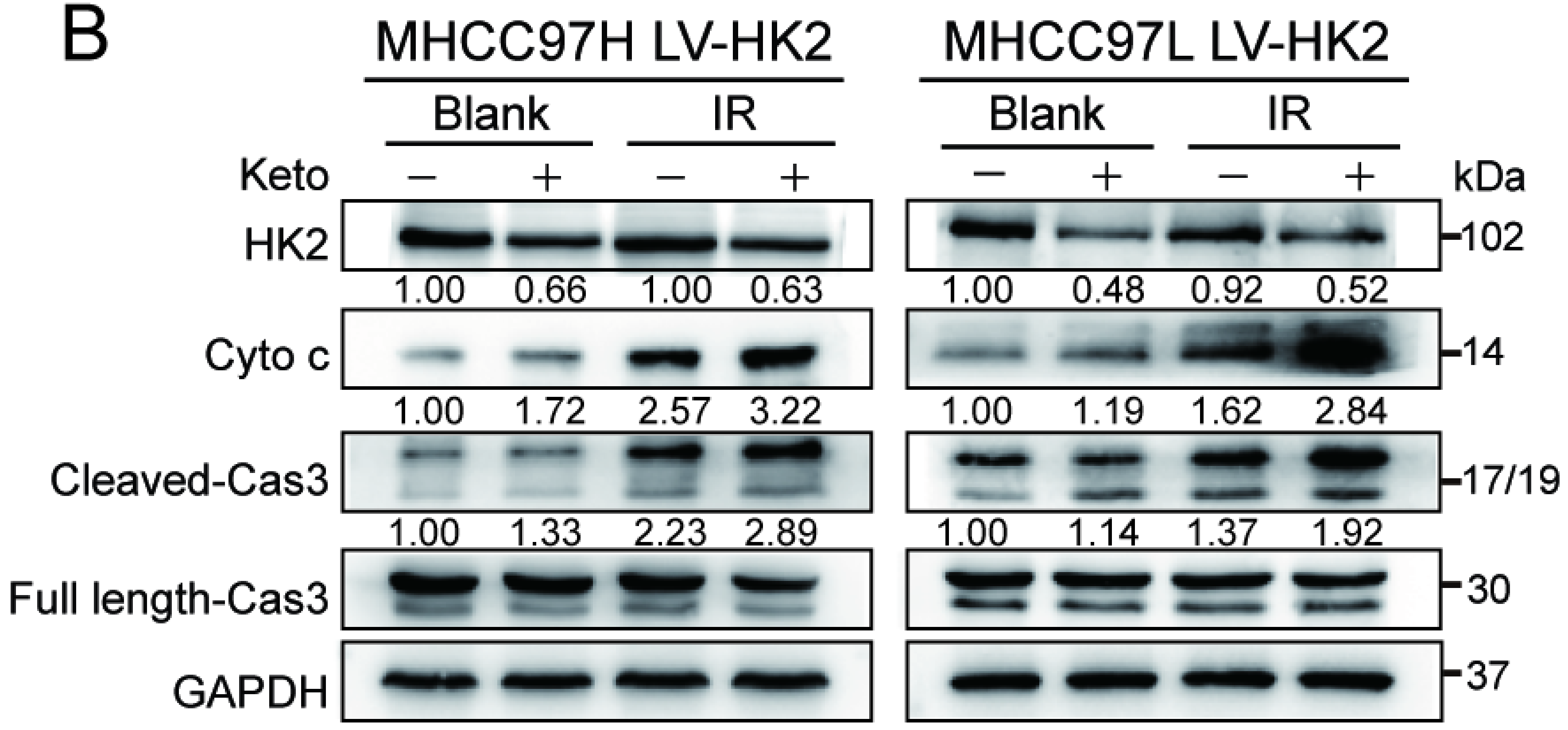
B
MHCC97H LV-HK2
MHCC97L LV-HK2
Blank
IR
Blank
IR
Keto
−
+
−
+
−
+
−
+
kDa
HK2
102
1.00 0.66 1.00 0.63
1.00 0.48 0.92 0.52
Cyto c
14
1.00 1.72 2.57 3.22
1.00 1.19 1.62 2.84
Cleaved-Cas3
17/19
1.00 1.33 2.23 2.89
1.00 1.14 1.37 1.92
Full length-Cas3
30
GAPDH
37

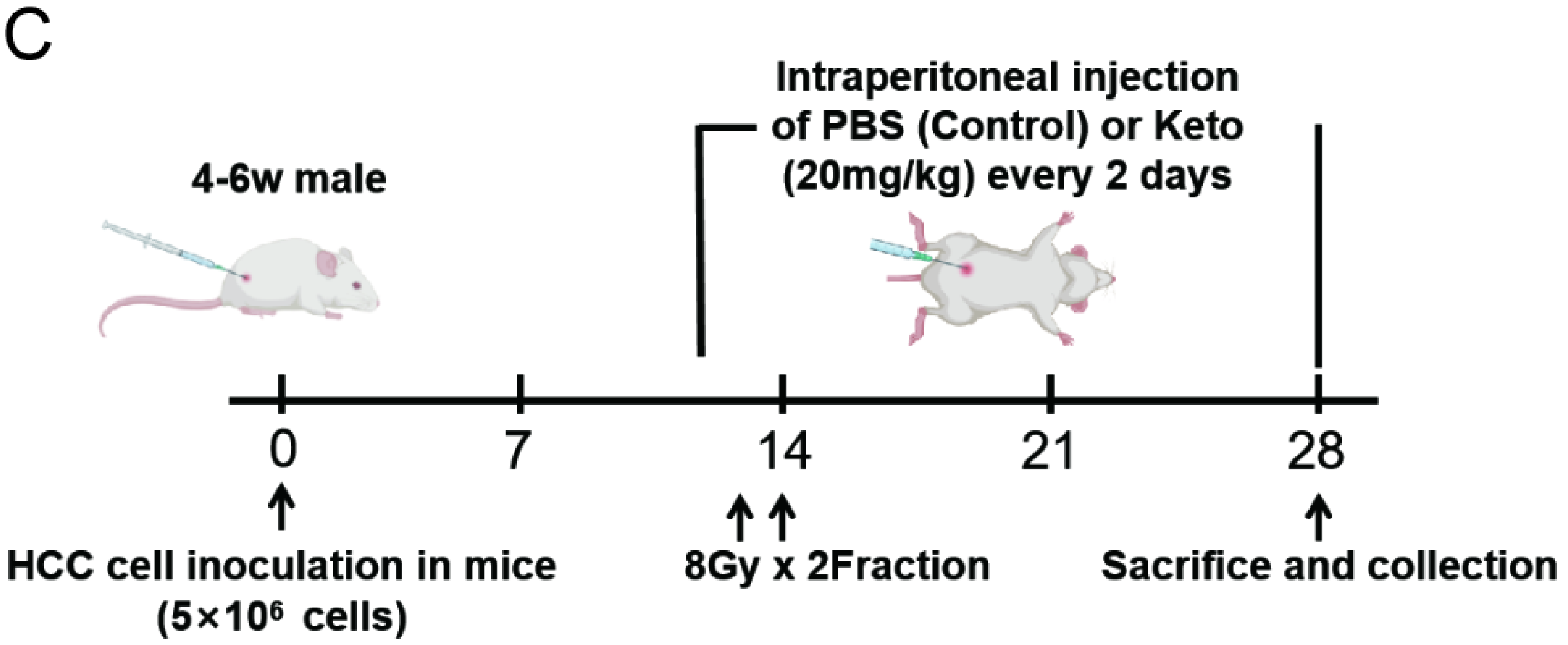
C
4-6w male
Intraperitoneal injection
of PBS (Control) or Keto
(20mg/kg) every 2 days
0
7
14
21
28
HCC cell inoculation in mice
(5×10⁶ cells)
8Gy x 2Fraction
Sacrifice and collection

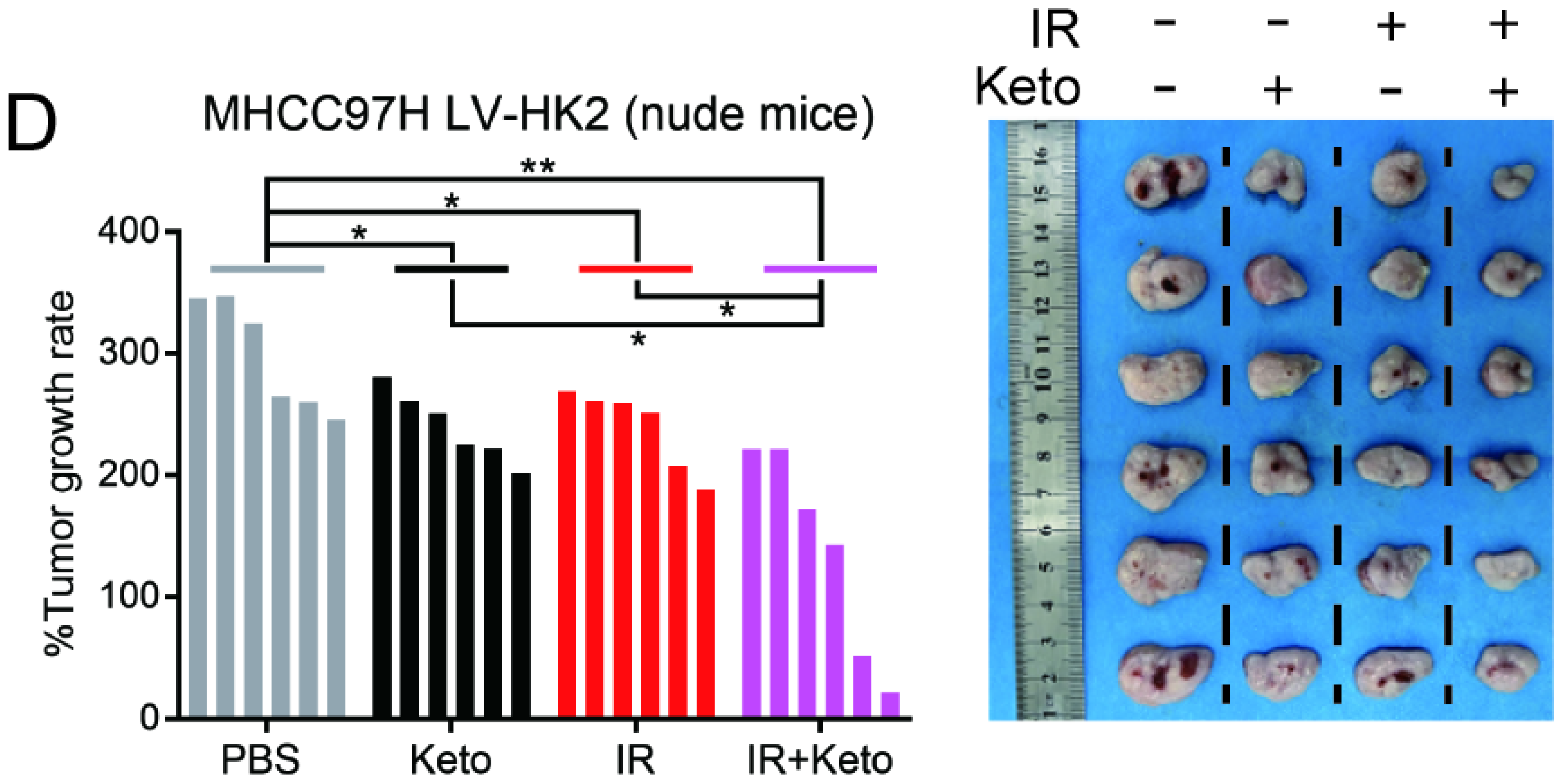

D
MHCC97H LV-HK2 (nude mice)
**
*
*
*
*
400
300
200
100
0
%Tumor growth rate
PBS
Keto
IR
IR+Keto
IR - - + +
Keto - + - +

E

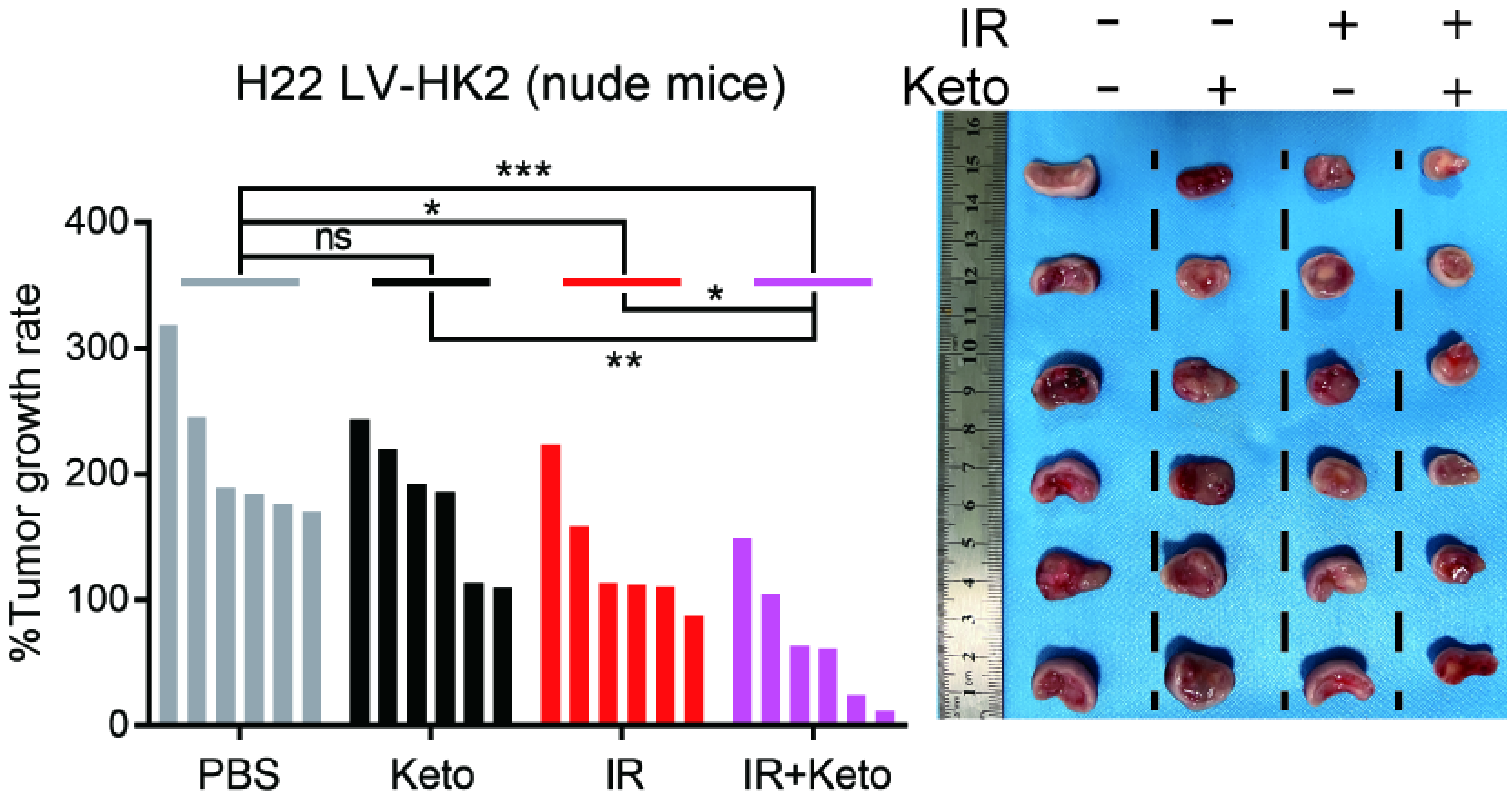
H22 LV-HK2 (nude mice)
%Tumor growth rate
400
300
200
100
0
***
*
ns
*
**
PBS
Keto
IR
IR+Keto
IR - - + +
Keto - + - +

F

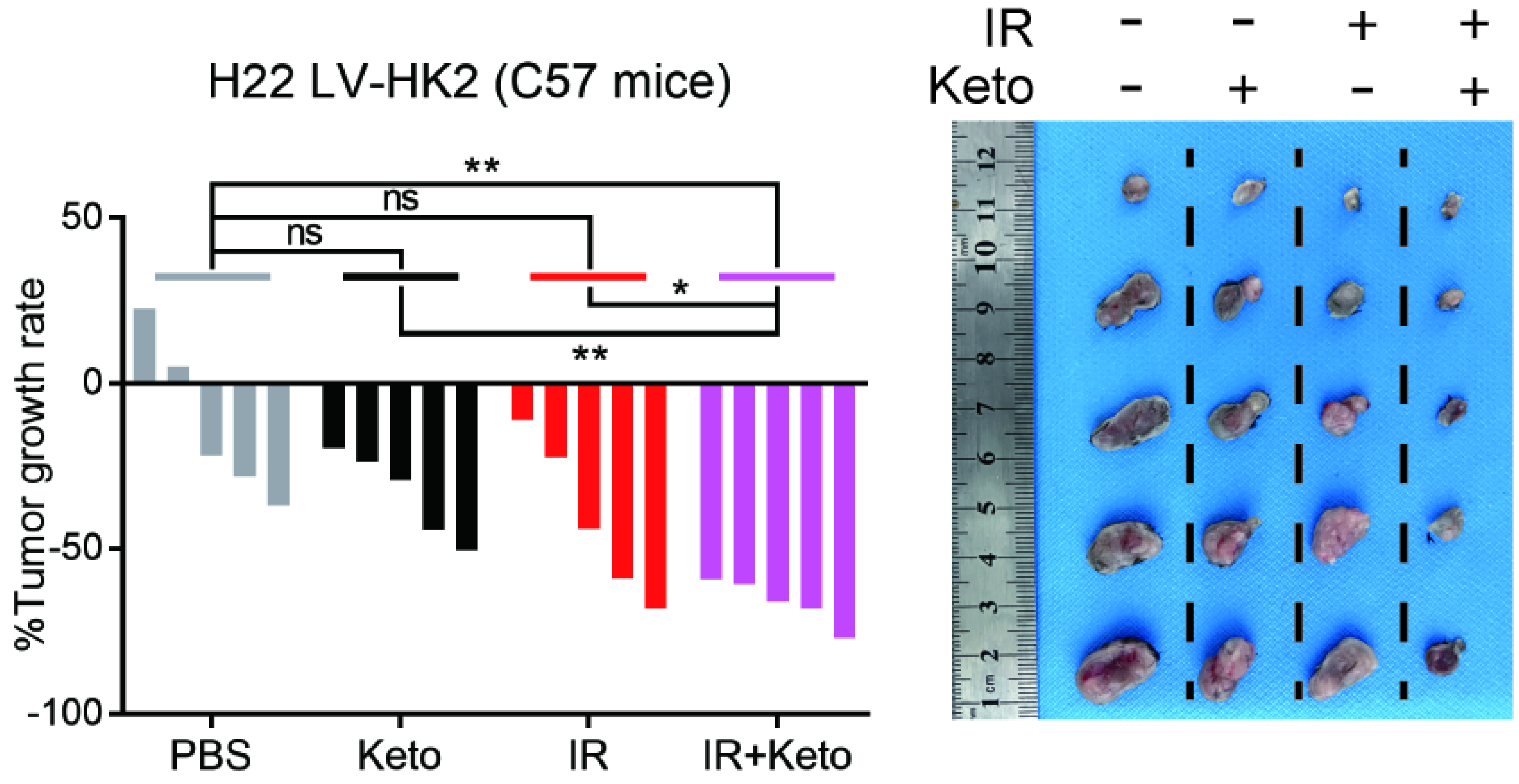
H22 LV-HK2 (C57 mice)
%Tumor growth rate
50
0
-50
-100
**
ns
ns
*
**
PBS
Keto
IR
IR+Keto
IR - - + +
Keto - + - +

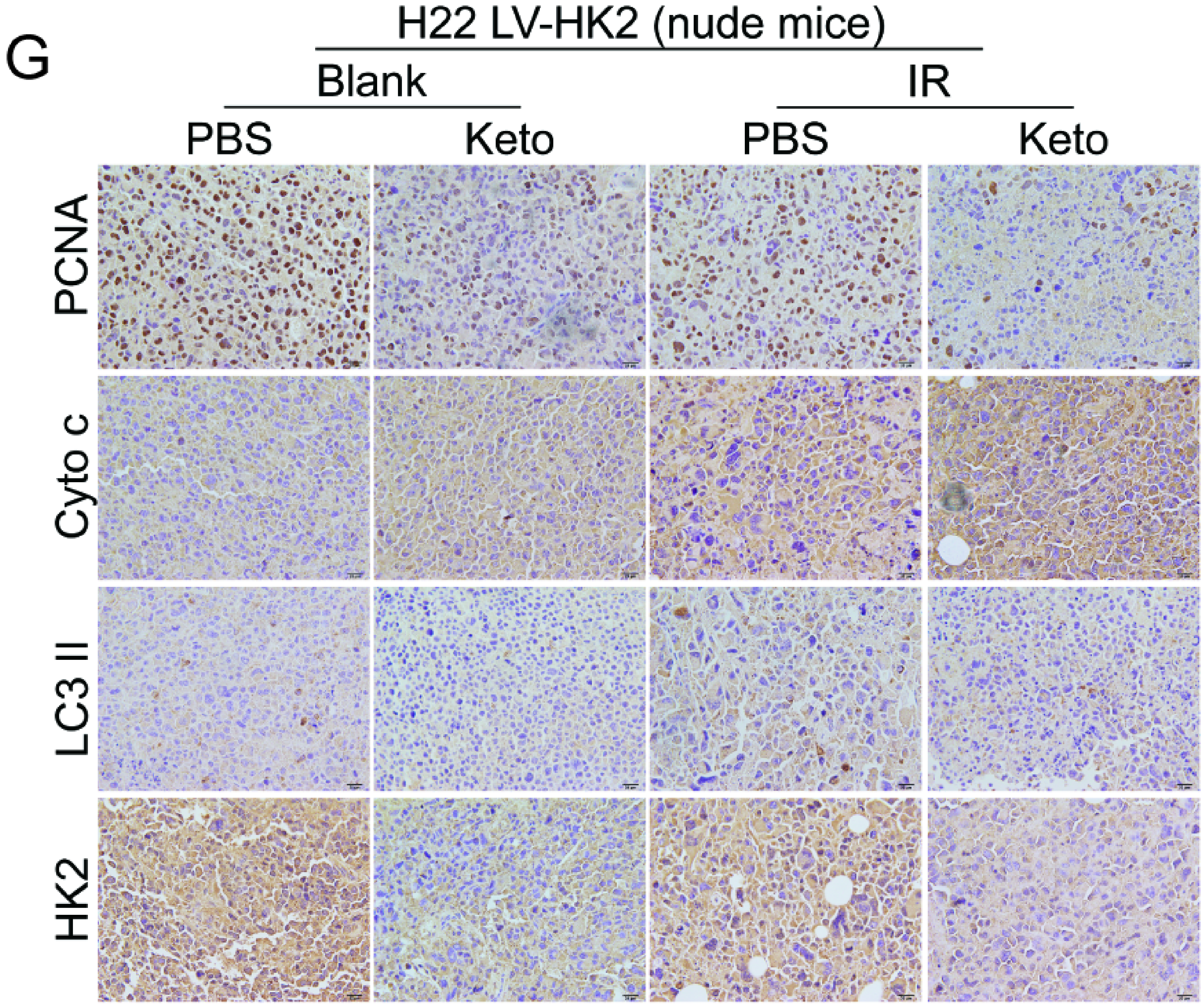
G
H22 LV-HK2 (nude mice)
Blank
IR
PBS
Keto
PBS
Keto
PCNA
Cyto c
LC3 II
HK2

H

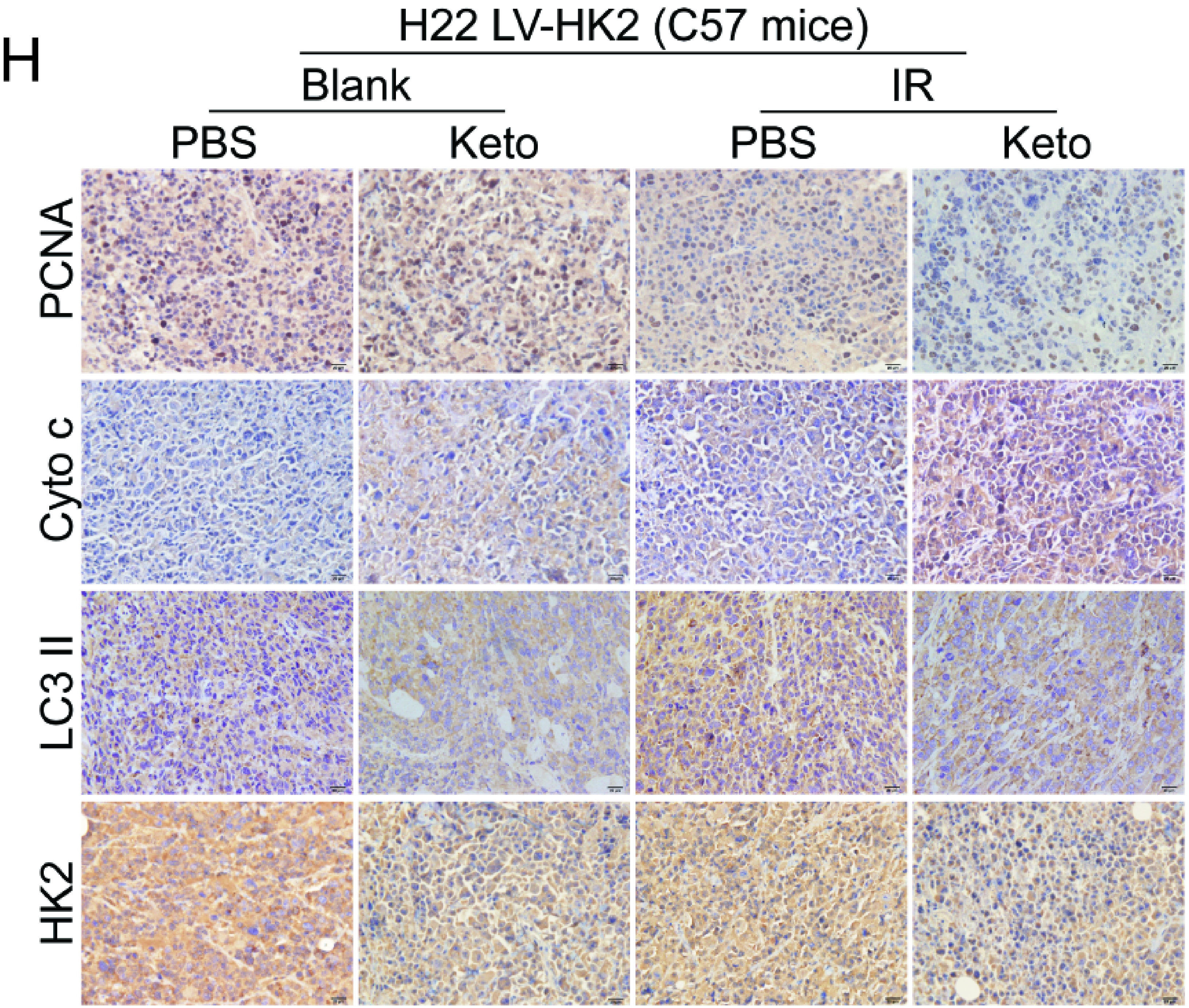

H22 LV-HK2 (C57 mice)
Blank
IR
PBS
Keto
PBS
Keto
PCNA
Cyto c
LC3 II
HK2

I

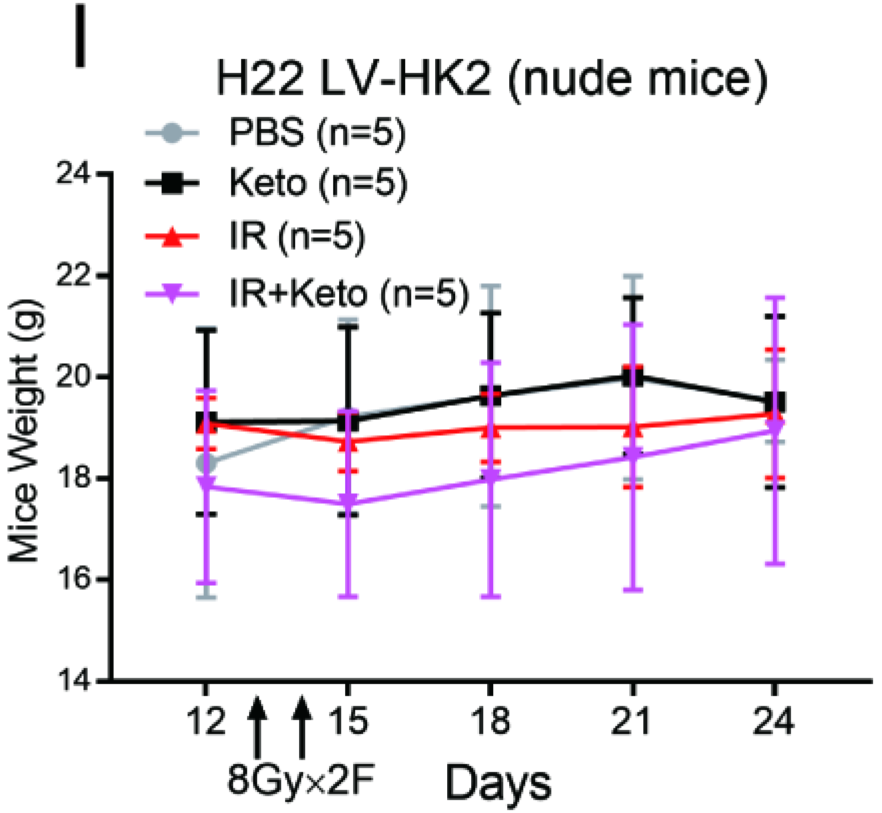
H22 LV-HK2 (nude mice)
PBS (n=5)
Keto (n=5)
IR (n=5)
IR+Keto (n=5)
Mice Weight (g)
24
22
20
18
16
14
12
15
18
21
24
8Gy×2F
Days

J

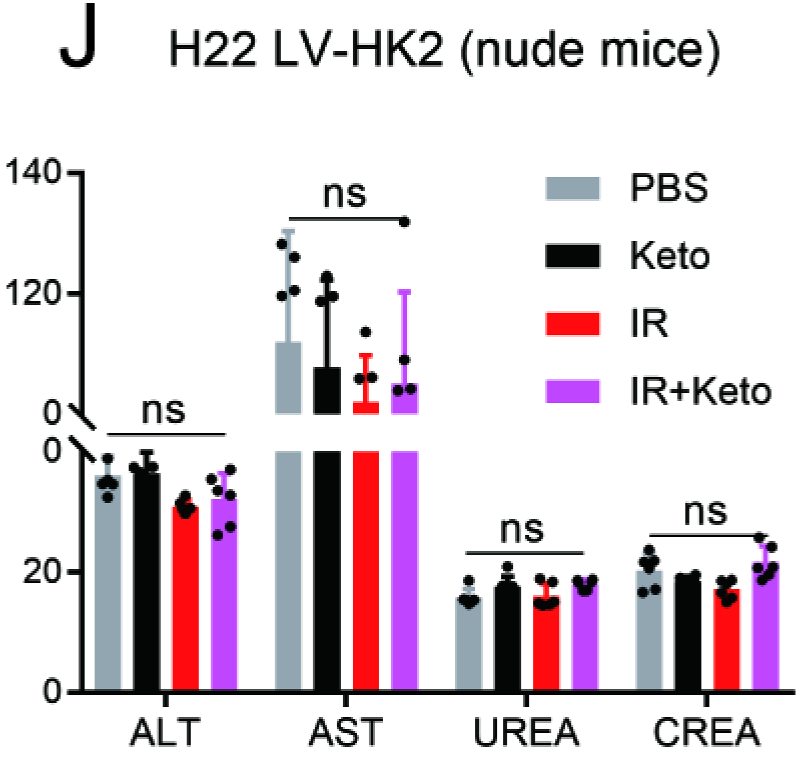
H22 LV-HK2 (nude mice)
140
120
0
0
20
0
ns
ns
ns
ns
PBS
Keto
IR
IR+Keto
ALT
AST
UREA
CREA

K

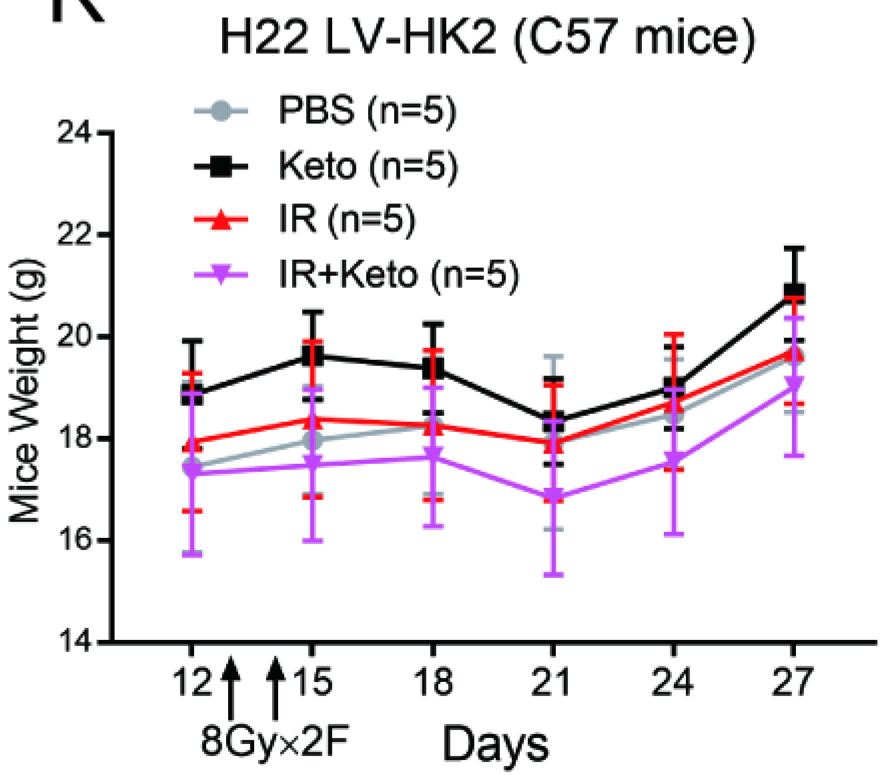

H22 LV-HK2 (C57 mice)
PBS (n=5)
Keto (n=5)
IR (n=5)
IR+Keto (n=5)
Mice Weight (g)
24
22
20
18
16
14
12
15
18
21
24
27
8Gy×2F
Days

L

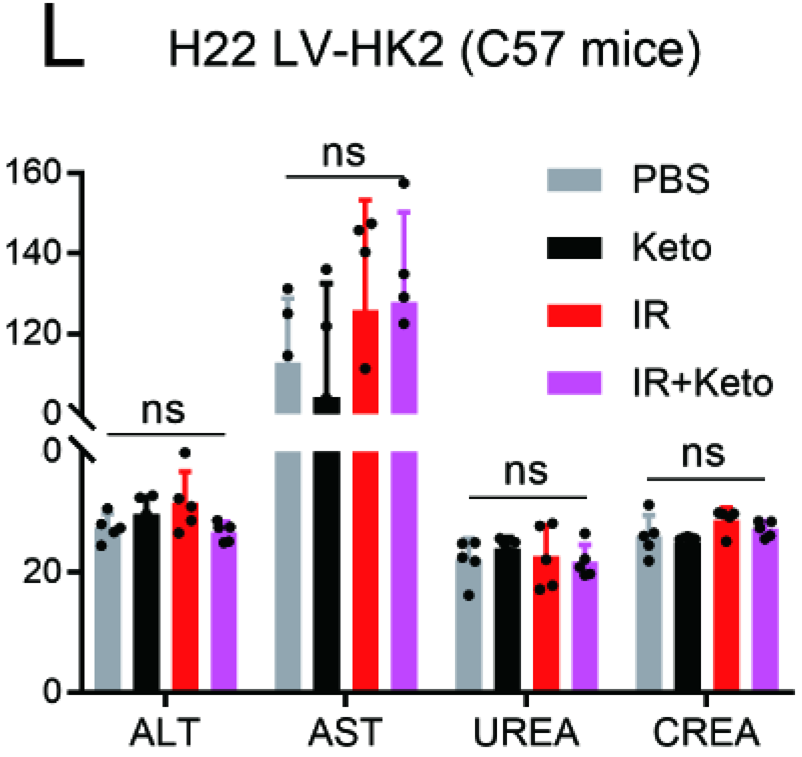

H22 LV-HK2 (C57 mice)
160
140
120
0
0
20
0
ns
ns
ns
ns
PBS
Keto
IR
IR+Keto
ALT
AST
UREA
CREA

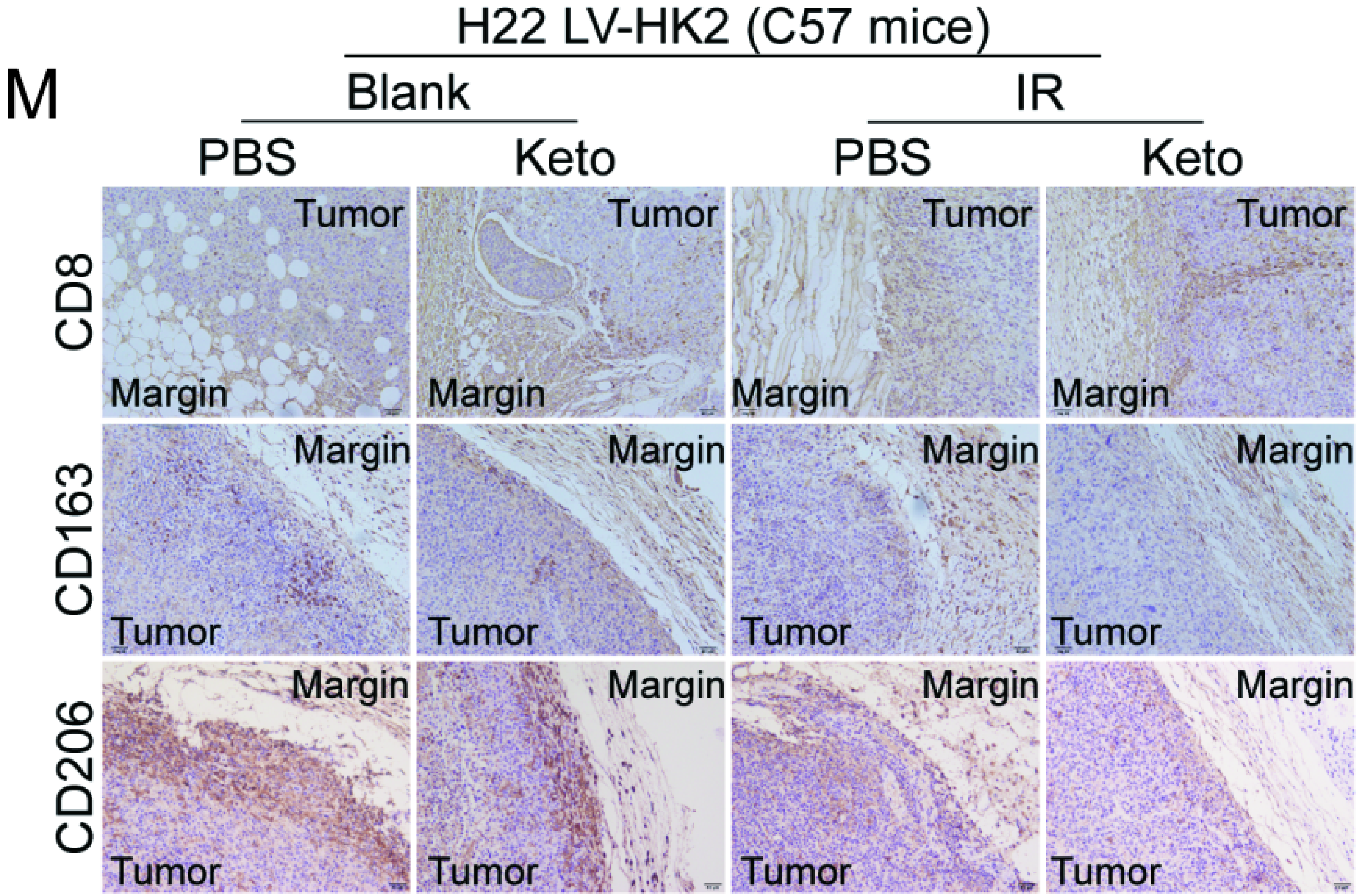
M
H22 LV-HK2 (C57 mice)
Blank
IR
PBS
Keto
PBS
Keto
CD8
CD163
CD206
Tumor
Margin

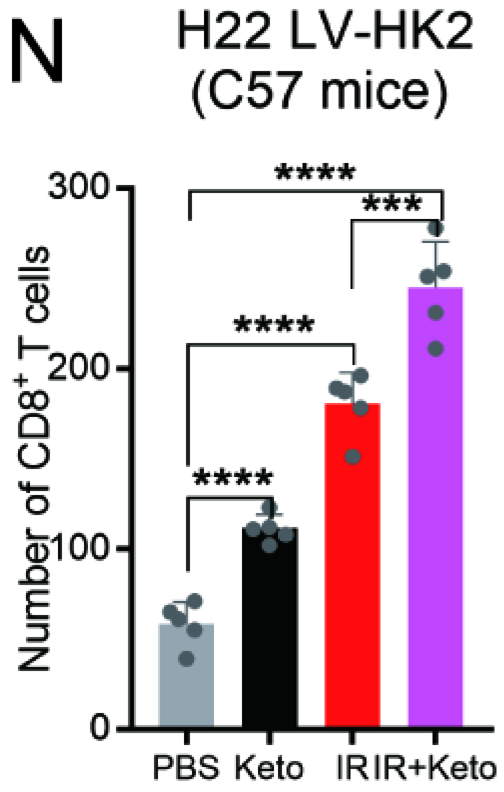
N
H22 LV-HK2
(C57 mice)
Number of CD8$^+$ T cells
300
200
100
0
****
***
****
****
PBS
Keto
IR
IR+Keto

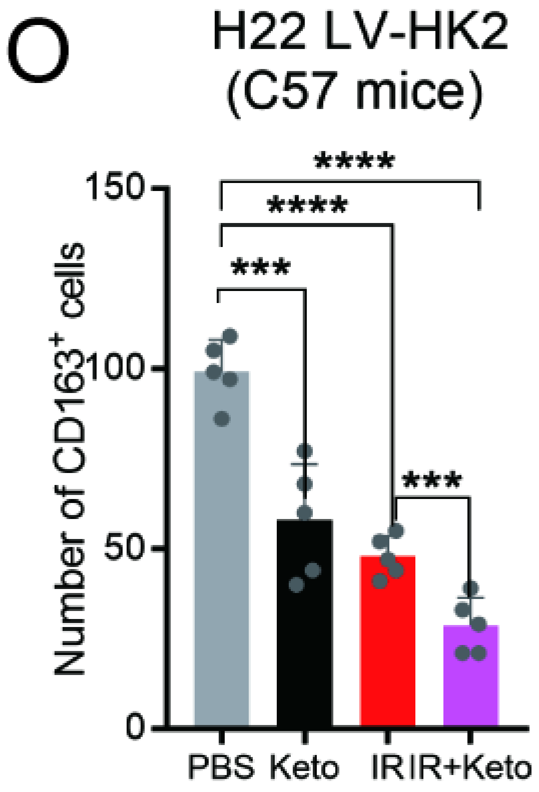
O
H22 LV-HK2
(C57 mice)
Number of CD163+ cells
150
100
50
0
****
****
***
***
PBS
Keto
IR
IR+Keto

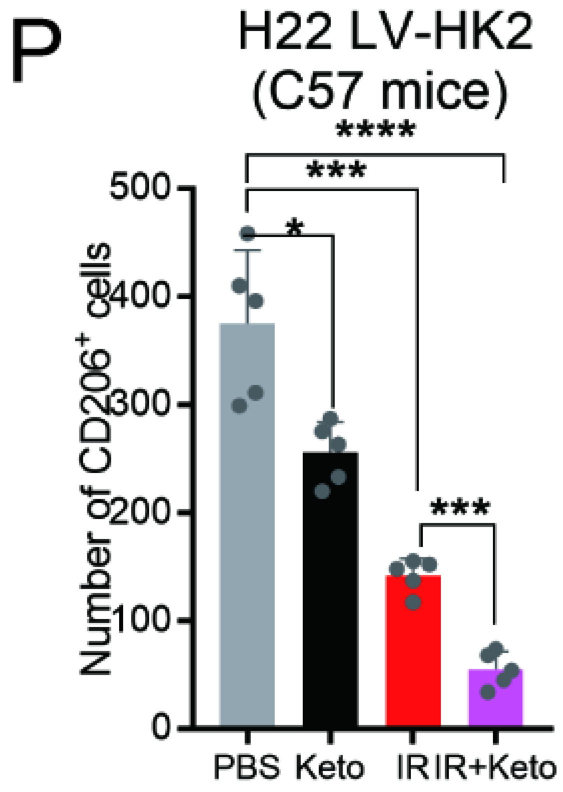
P
H22 LV-HK2
(C57 mice)
****
***
*
***
Number of CD206+ cells
500
400
300
200
100
0
PBS
Keto
IR
IR+Keto

Q

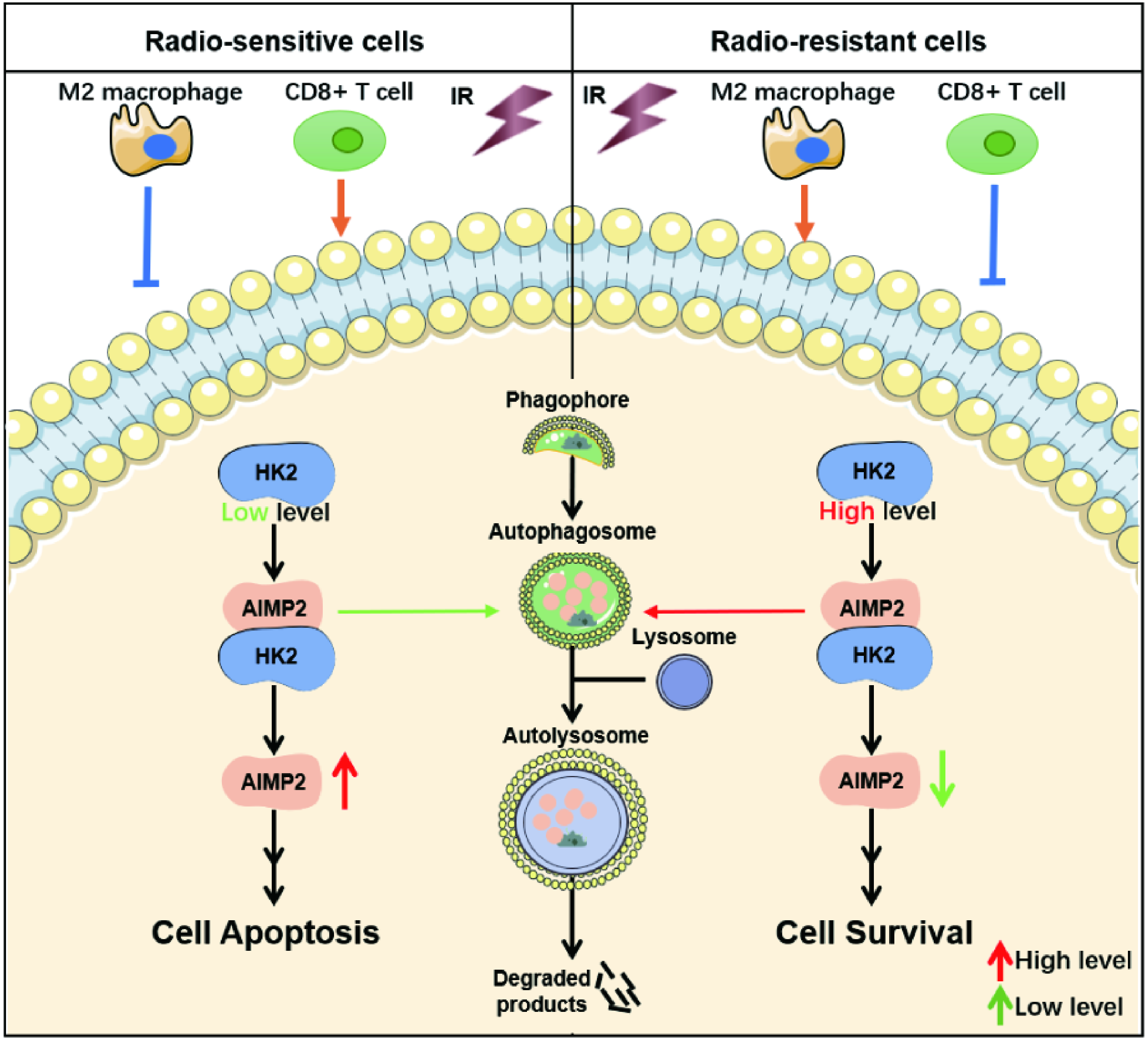
Radio-sensitive cells
Radio-resistant cells
M2 macrophage
CD8+ T cell
IR
IR
M2 macrophage
CD8+ T cell
Phagophore
HK2
Low level
AIMP2
HK2
AIMP2
Cell Apoptosis
Autophagosome
Lysosome
Autolysosome
Degraded products
HK2
High level
AIMP2
HK2
AIMP2
Cell Survival
High level
Low level
