# Supplementary material for: Hexokinase 2 confers radio-resistance in hepatocellular carcinoma by promoting autophagy-dependent degradation of AIMP2
Source: Cell Death Dis. 2023 Aug 1;14(8):488. doi: 10.1038/s41419-023-06009-2 (PMC10390495; doi:10.1038/s41419-023-06009-2)
Supplement: Supplementary file 7 — Supplementary Figure [file 41419_2023_6009_MOESM7_ESM.docx]

# Hexokinase 2 Confers Radio-resistance on Hepatocellular Carcinoma by Targeting AIMP2

**Supplementary Figures**


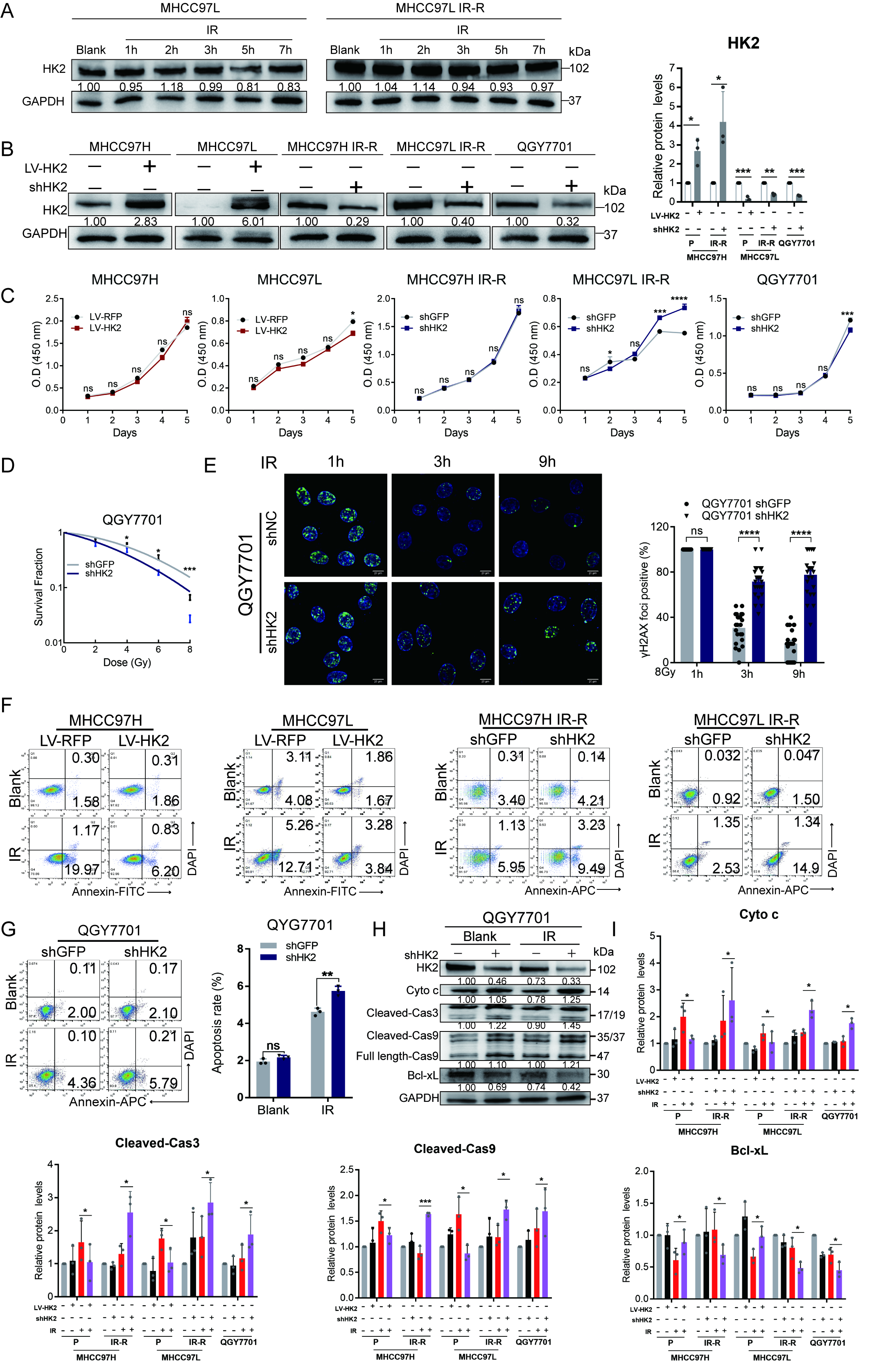


**Supplement. Fig. 1 HK2’s role in proliferation and IR-induced apoptosis. A** Western blotting of HK2 in radio-resistant HCC cells at indicated time point upon 6 Gy radiation. **B** Western blotting analysis and quantification were used to identify the transfection efficiencies, respectively (n = 3). **C** CCK8 assays in radio-sensitive and radio-resistant HCC cells without radiation (n = 3). **D** Colony formation assays of innate radio-resistant cells QGY7701 (shGFP&shHK2) after exposure to indicated doses of IR. Error bars are the SEM of at least three independent replicates. **E** Different HK2 level of QGY7701 were treated with 8 Gy radiation, then stained and quantified at the indicated times with antibodies to pH2AX-Ser139. Error bars are the SEM of at least ten independent replicates. Scale bar 20 μm. **F-G** Apoptosis in HCC cells with different HK2 level with or without 8 Gy radiation (n = 3). **H** Western blotting analysis of apoptosis-related proteins in QGY7701 (shGFP&shHK2) with or without 8 Gy radiation. **I** Western blotting quantification of apoptosis-related proteins in indicated cell lines (n = 3). **p* < . 05, ***p* < . 01, ****p* < . 001, *****p* < . 0001, based on Students’ t test.

**
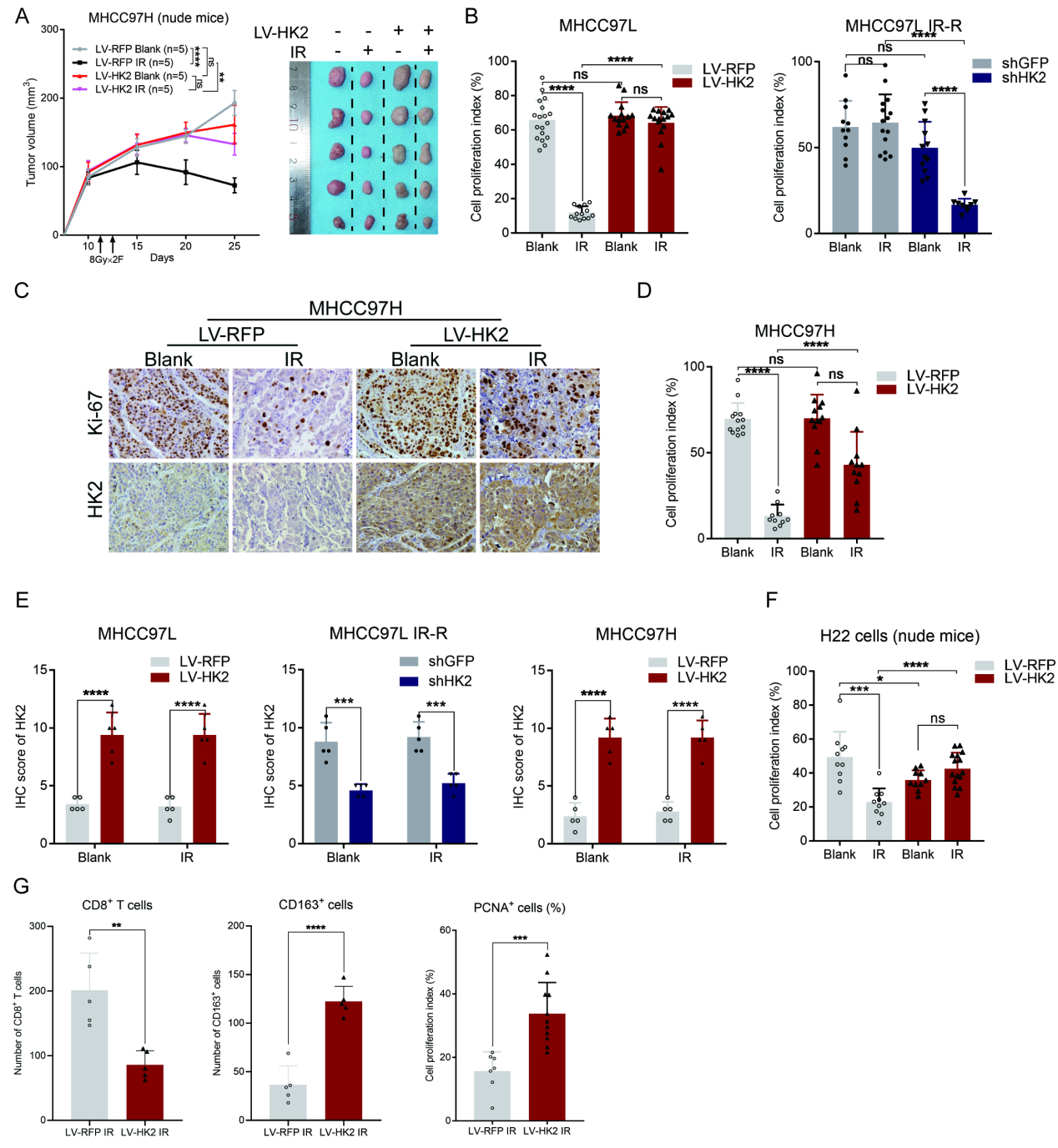
**

**Supplement. Fig. 2 HK2’ s role in radio-resistance gene in vivo. A** Subcutaneous xenograft nude mice model to twice 8 Gy radiation responsiveness among MHCC97H with different HK2 status. Tumor growth curves and images upon necropsy were presented (n = 5). **B** Representative IHC quantification of Ki-67 in MHCC97L and MHCC97L IR-R groups. Error bars are the SEM of at least ten independent replicates. **C** Representative IHC staining Ki-67 and HK2 in MHCC97H group. Scale bar 20 μm. **D** Representative IHC quantification of Ki-67 in the MHCC97H group. Error bars are the SEM of at least ten independent replicates. **E** Representative IHC quantification of HK2 in indicated groups (n = 5). **F** Representative IHC quantification of PCNA in H22 nude mice group. Error bars are the SEM of at least ten independent replicates. **G** Representative IHC quantification of PCNA, CD8 and CD163 in the formalin-fixed tumor sections from indicated treatment groups (n = 5). Data are represented as mean ± SEM. **p* < . 05, ***p* <0. 01，****p* <0. 001，*****p* <0. 0001, based on Student’s t test.

**
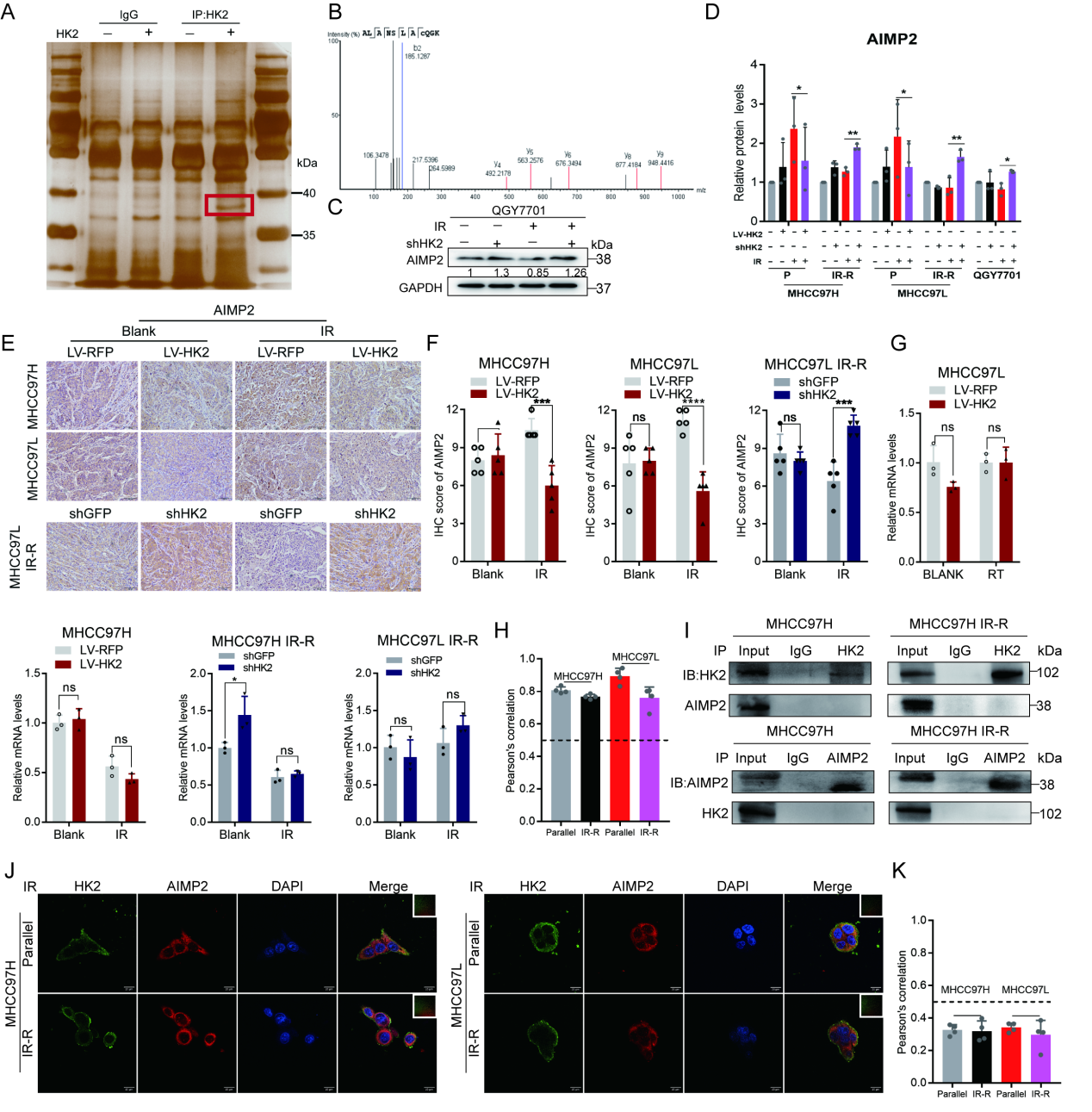
**

**Supplement. Fig. 3 HK2 complexes with AIMP2 in protein and space level. A** Sliver staining of MHCC97H (LV-RFP&LV-HK2) after 8 Gy radiation, identified the specific band, cut it and send it to mass spectrometric analyse. **B** AIMP2 were the potential co-localized protein statistically. **C** Protein levels of AIMP2 in QGY7701 with different HK2 expression status. **D** Quantification of AIMP2 protein level in indicated cell lines. Error bars are the SEM of at least three independent replicates. **E-F** Representative IHC staining and qualification of AIMP2 in the formalin-fixed tumor sections from blank and IR groups (n = 5). Scale bar 20 μm. **G** mRNA level of AIMP2 in different HK2 status cells (n = 3). **H** Qualification of the co-localization of HK2 (green) and AIMP2 (red) in MHCC97H, MHCC97L, MHCC97H IR-R, MHCC97L IR-R post 8 Gy radiation (n = 4). **I** Co-IP of HK2 and AIMP2 in MHCC97H and MHCC97H IR-R without IR. **J-K** IF and Qualification of the co-localization of HK2 (green) and AIMP2 (red) in MHCC97H, MHCC97L, MHCC97H IR-R, MHCC97L IR-R without IR (n = 4). Data are represented as mean ± SEM. **p* < . 05, ***p* <0. 01，****p* <0. 001，*****p* <0. 0001, based on Student’s t test.


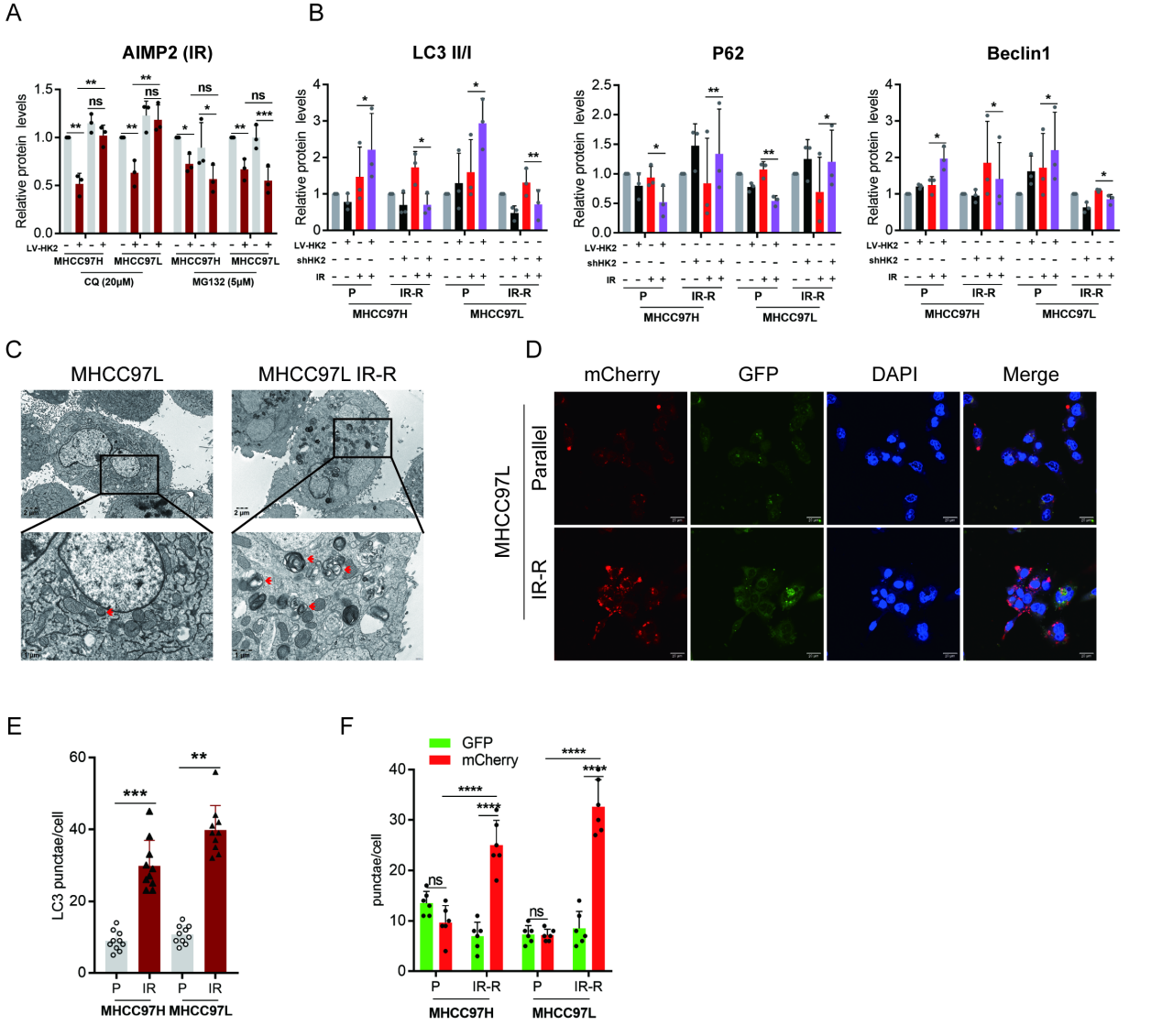


**Supplement. Fig. 4 HK2 regulates lysosomal-dependent autophagy of AIMP2.**

**A** AIMP2 quantification of MG-132 (20 μM, 24 h) and CQ (5 μM, 24 h) treatment in MHCC97H and MHCC97L cells with or without HK2 overexpression post 8 Gy radiation, respectively (n = 3). **B** Quantification of LC3 II/I, P62 and Beclin1 in indicated cell lines, respectively (n = 3). **C** TEM of indicating cells post 8Gy radiation after 48h. Error bars are the SEM of at least ten independent replicates. **D-F** IF analysis and qualification of autophagosomes in indicated post-IR cells which transfected with plasmid expressing mCherry-GFP-LC3 II. Error bars are the SEM of at least five independent replicates. Scale bar 20 μm. Data are represented as mean ± SEM. **p* < . 05, ***p* <0. 01，****p* <0. 001，*****p* <0. 0001, based on Student’s t test.

**
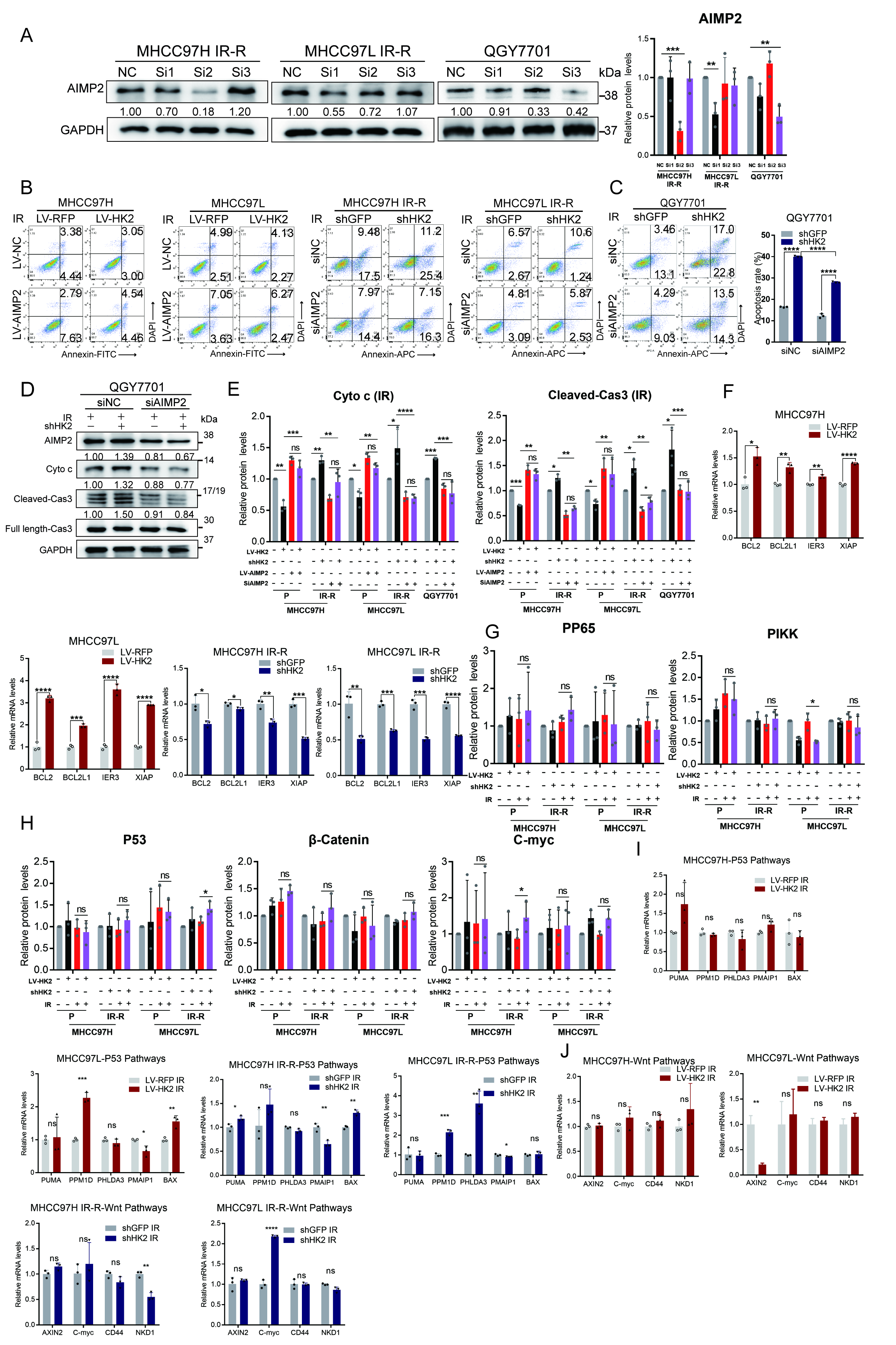
**

**Supplement. Fig. 5 The function role of HK2 is dependent on AIMP2. A** Screening and quantification of the most efficient siRNA segments which transfected into MHCC97H IR-R , MHCC97L IR-R and QGY7701 (n = 3). **B** Images of apoptosis in HCC cells with different HK2 and AIMP2 level post 8 Gy radiation, respectively (n = 3). **C** Representative images and statistics of apoptosis in QGY7701 with different HK2 and AIMP2 status after 8 Gy radiation, respectively (n = 3). **D** Western blotting of apoptosis related protein in QGY7701 (shGFP&shHK2). **E** Quantification of Cyto c, Cleaved-Cas3 protein level in indicated cell lines (n = 3). **F** mRNA level of NF-κb related apoptosis-associated gene in different HK2 status cells after 8Gy radiation (n = 3). **G** Quantification of PP65, PIKK, P53, β-Catenin, C-myc protein level in indicated cell lines, respectively (n = 3). **I-J** mRNA level of P53 and Wnt pathways in indicated cell lines (n = 3). Data are represented as mean ± SEM. **p* < . 05, ***p* <0. 01，****p* <0. 001，*****p* <0. 0001.

**
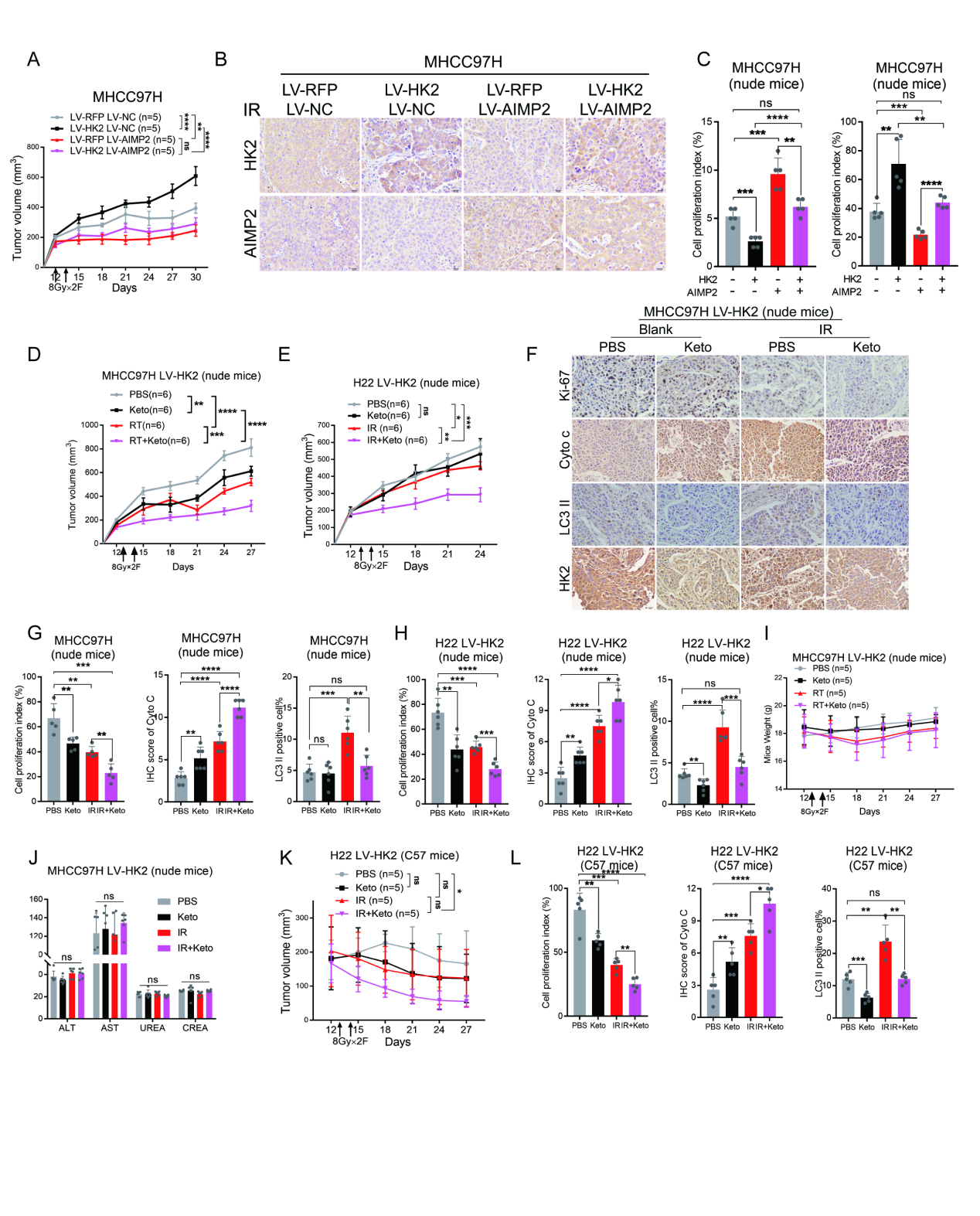
**

**Supplement. Fig. 6 Pharmacological inhibition of HK2 significantly alleviates its radio-resistant effect A** Tumor growth of indicated group was presented (n = 5). **B** Representative IHC stain of HK2 and AIMP2 were presented. **C** Quantification of Cyto c and Ki-67 in the indicated treatment group (n = 5). **D-E** Tumor growth curve of subcutaneous xenograft models in nude mice with indicated cells and treatments, each groups contain 6 mouse. **F-G** Representative IHC staining and qualification of Ki-67, Cyto c, LC3 II and HK2 in the formalin-fixed tumor sections from nude mice with MHCC97H LV-HK2 groups (n = 6). Scale bar 20 μm. **H** Qualification of PCNA, Cyto c and LC3 II in the formalin-fixed tumor sections from nude mice with MHCC97H LV-HK2 groups (n = 6), Scale bar 20 μm. **I-J** Mice weight and indicators of mice liver and kidney in nude mice with MHCC97H LV-HK2 groups (n = 6). The units of ALT and AST are U/L. The units of UREA and CREA are Mmol/L and μmol/L, respectively. **K** Tumor growth curve of subcutaneous xenograft models in C57 mice with H22 LV-HK2 and treatments, each groups contain 5 mouse. **L** Qualification of PCNA, Cyto c, LC3 II in the formalin-fixed tumor sections from C57 mice with H22 LV-HK2 groups (n = 5). Scale bar 20 μm. Data are represented as mean ± SEM. **p* < . 05, ***p* <0. 01，****p* <0. 001，*****p* <0. 0001.
