# Supplementary material for: Hexokinase 2 confers radio-resistance in hepatocellular carcinoma by promoting autophagy-dependent degradation of AIMP2
Source: Cell Death Dis. 2023 Aug 1;14(8):488. doi: 10.1038/s41419-023-06009-2 (PMC10390495; doi:10.1038/s41419-023-06009-2)
Supplement: Supplementary file 8 — Supplementary Table 1 [file 41419_2023_6009_MOESM8_ESM.docx]

**Hexokinase 2 Confers Radio-resistance in Hepatocellular Carcinoma by Promoting Autophagy-dependent Degradation of AIMP2**

**Supplementary Table 1. Primers for qPCR**

| **Gene** | **Forward** | **Reverse** |
| --- | --- | --- |
| HK2 | GAGCCACCACTCACCCTACT | CCAGGCATTCGGCAATGTG |
| GAPDH | ACCACAGTCCATGCCATCAC | TCCACCACCCTGTTGCTGTA |
| β-ACTIN | CATGTACGTTGCTATCCAGGC | CTCCTTAATGTCACGCACGAT |
| AIMP2 | GGTTTGCGTTGATCACAATG | AGTTGAAGGCAGCAGTCGAT |
| AXIN2 | CAAACTCATCGCTTGCTTTTT | CACTTACTTTTTCTGTGGGGAAG |
| NKD1 | GCTGAGCGTGTCTCTCAACA | AGGAGTGGATCGGGAGACAG |
| CD44 | TCCACATGGAATACACCTGC | CAAGTTTTGGTGGCACACAG |
| C-Myc | CGACGAGACCTTCATCAAAAAC | CTTCTCTGAGACGAGCTTGG |
| PUMA | TGTCGATGCTGCTCTTCTTG | GTGTGGAGGAGGAGGAGTGG |
| PPM1D | ATGTATGTAGCTCACGTAGGTG | CTCCTTCTAACAGGTCCATTGT |
| PHLDA3 | ACATCTACTTCACGCTGGTG | CTGCTGGTTCTTGAACTTGAC |
| BCL2 | GGTGGGGTCATGTGTGTGG | CGGTTCAGGTACTCAGTCATCC |
| BCL2L1 | GAGCTGGTGGTTGACTTTCTC | TCCATCTCCGATTCAGTCCCT |
| IER3 | CAGCCGCAGGGTTCTCTAC | GATCTGGCAGAAGACGATGGT |
| XIAP | ACCGTGCGGTGCTTTAGTT | TGCGTGGCACTATTTTCAAGATA |
| BAX | CCCGAGAGGTCTTTTTCCGAG | CCAGCCCATGATGGTTCTGAT |
