# Supplementary material for: Hexokinase 2 confers radio-resistance in hepatocellular carcinoma by promoting autophagy-dependent degradation of AIMP2
Source: Cell Death Dis. 2023 Aug 1;14(8):488. doi: 10.1038/s41419-023-06009-2 (PMC10390495; doi:10.1038/s41419-023-06009-2)
Supplement: Supplementary file 19 — Extended Supplementary Figure5 (Supplementary Figure5 merge file) [file 41419_2023_6009_MOESM19_ESM.pdf]

A

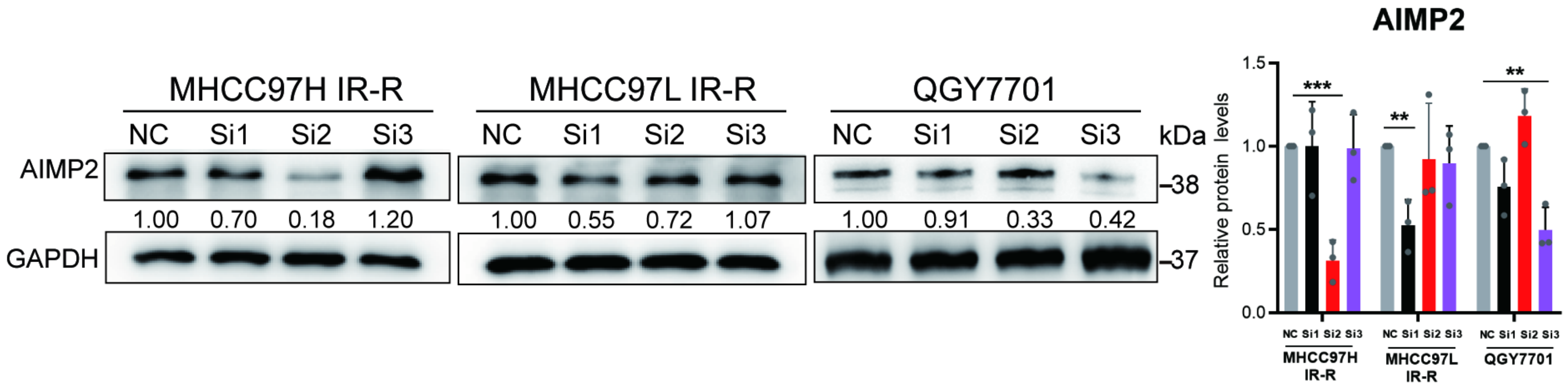
MHCC97H IR-R
MHCC97L IR-R
QGY7701
NC Si1 Si2 Si3
kDa
AIMP2
GAPDH
1.00 0.70 0.18 1.20
1.00 0.55 0.72 1.07
1.00 0.91 0.33 0.42
38
37
AIMP2
Relative protein levels
1.5
1.0
0.5
0.0
***
**
**
NC Si1 Si2 Si3
MHCC97H IR-R
MHCC97L IR-R
QGY7701

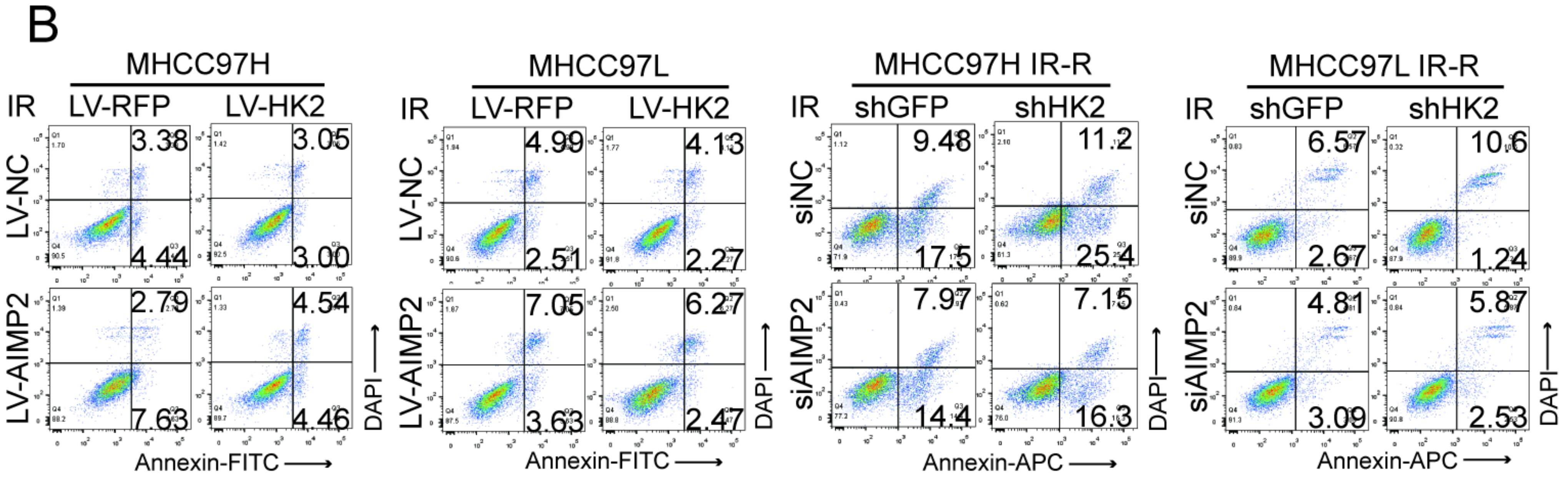
B
MHCC97H
IR
LV-RFP
LV-HK2
LV-NC
LV-AIMP2
3.38
4.44
3.05
3.00
2.79
7.63
4.54
4.46
Annexin-FITC
DAPI
MHCC97L
IR
LV-RFP
LV-HK2
LV-NC
LV-AIMP2
4.99
2.51
4.13
2.27
7.05
3.63
6.27
2.47
Annexin-FITC
DAPI
MHCC97H IR-R
IR
shGFP
shHK2
siNC
siAIMP2
9.48
17.5
11.2
25.4
7.97
14.4
7.15
16.3
Annexin-APC
DAPI
MHCC97L IR-R
IR
shGFP
shHK2
siNC
siAIMP2
6.57
2.67
10.6
1.24
4.81
3.09
5.87
2.53
Annexin-APC
DAPI

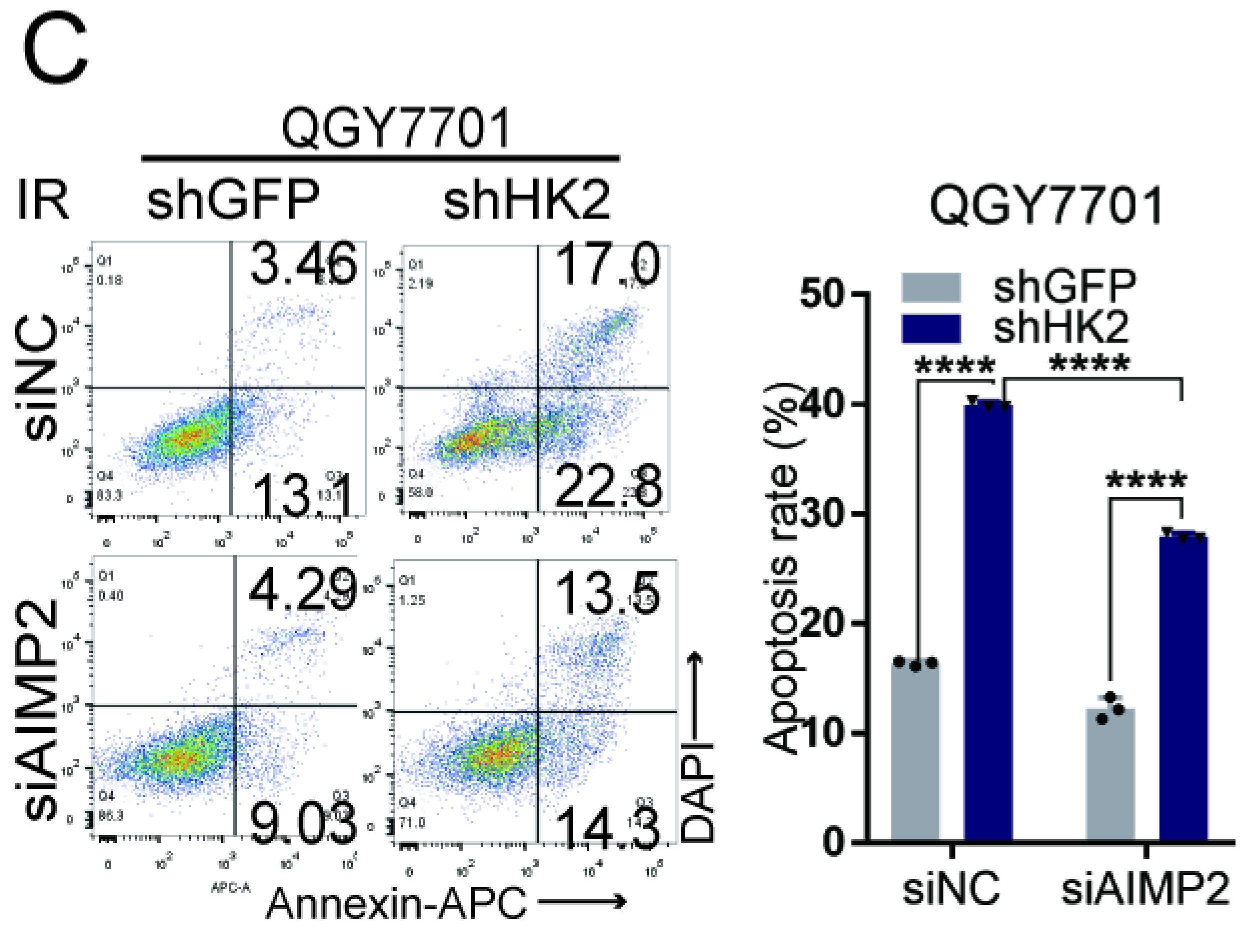
C
QGY7701
IR
shGFP
shHK2
siNC
siAIMP2
3.46
13.1
17.0
22.8
4.29
9.03
13.5
14.3
Annexin-APC
DAPI
QGY7701
shGFP
shHK2
Apoptosis rate (%)
****
****
****
siNC
siAIMP2

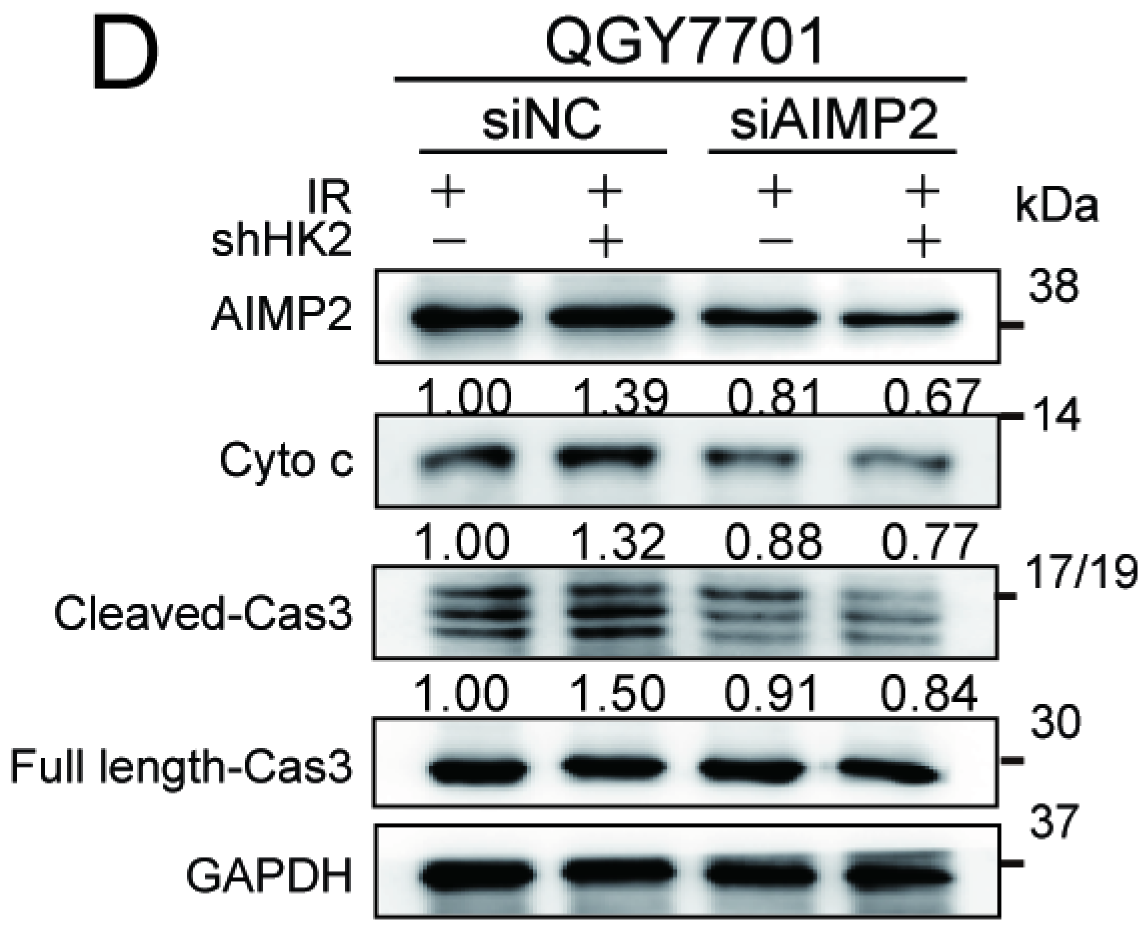
D
QGY7701
siNC
siAIMP2
IR
shHK2
kDa
AIMP2
38
1.00 1.39 0.81 0.67
14
Cyto c
1.00 1.32 0.88 0.77
17/19
Cleaved-Cas3
1.00 1.50 0.91 0.84
30
Full length-Cas3
37
GAPDH

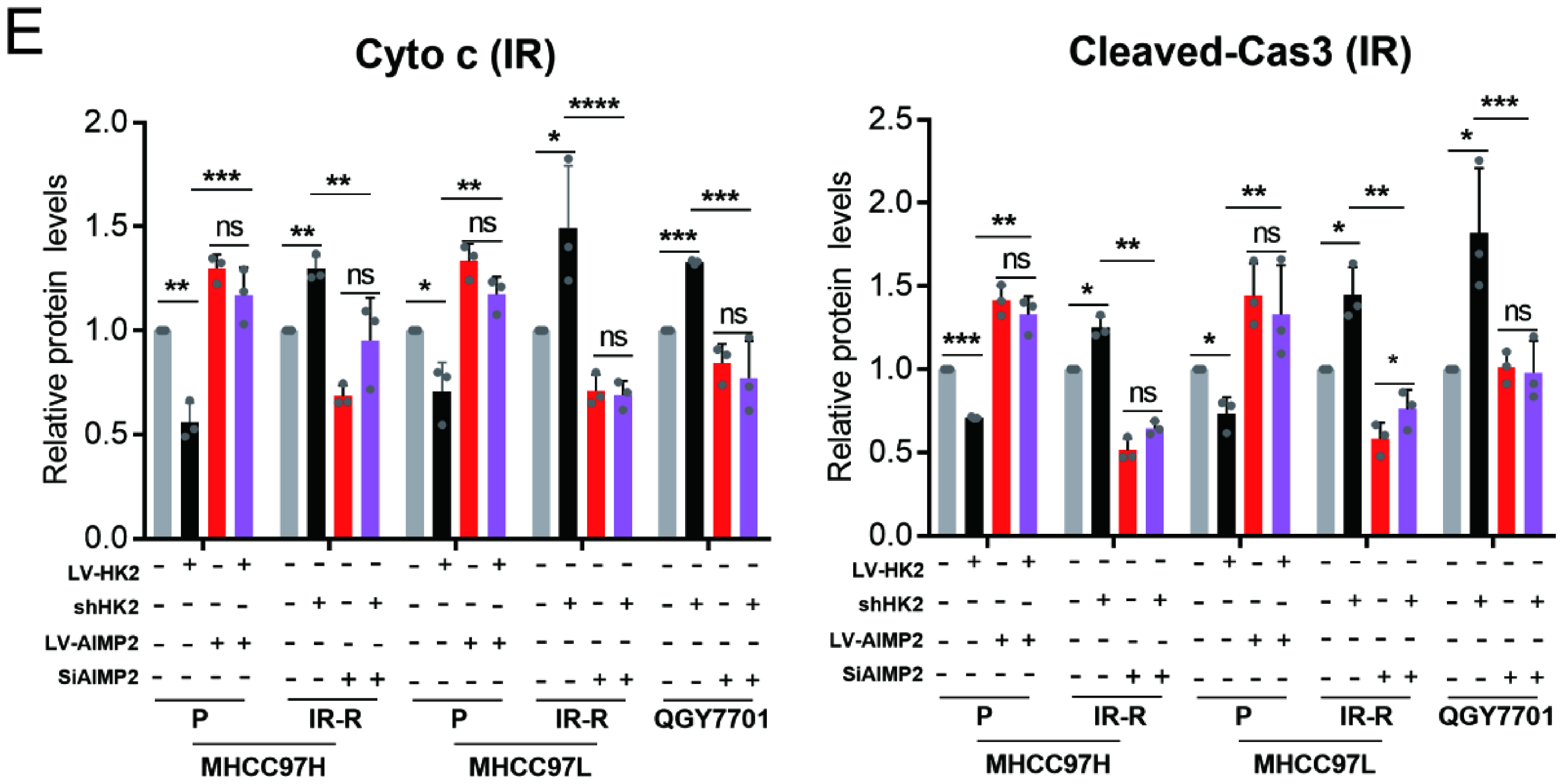
E
Cyto c (IR)
Cleaved-Cas3 (IR)
Relative protein levels
LV-HK2
shHK2
LV-AIMP2
SiAIMP2
P
IR-R
QGY7701
MHCC97H
MHCC97L

F

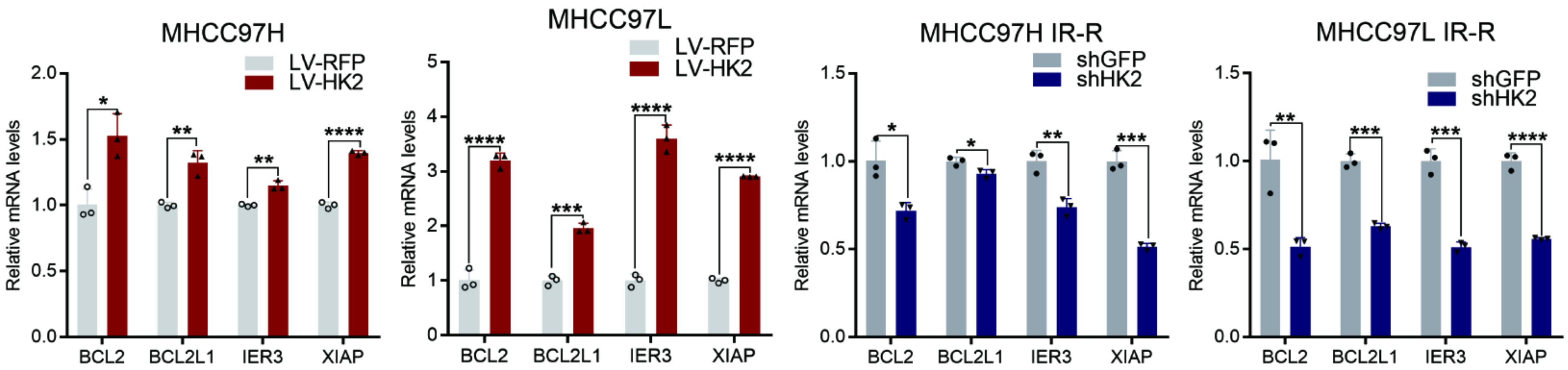
MHCC97H
LV-RFP
LV-HK2
Relative mRNA levels
BCL2
BCL2L1
IER3
XIAP
MHCC97L
MHCC97H IR-R
shGFP
shHK2
MHCC97L IR-R

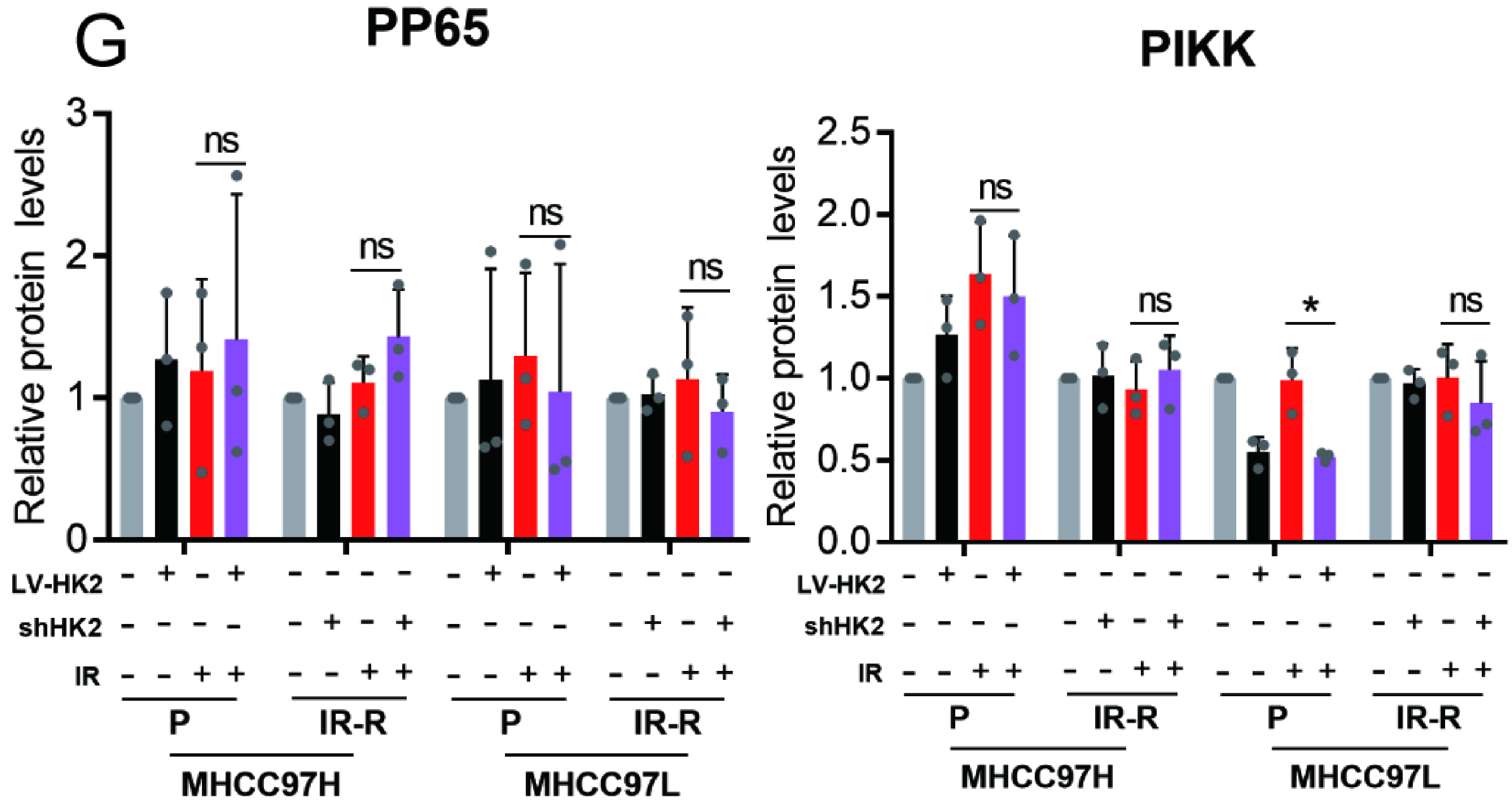

G
PP65
Relative protein levels
3
2
1
0
ns
ns
ns
ns
LV-HK2
shHK2
IR
P
IR-R
P
IR-R
MHCC97H
MHCC97L
PIKK
Relative protein levels
2.5
2.0
1.5
1.0
0.5
0.0
ns
ns
*
ns
LV-HK2
shHK2
IR
P
IR-R
P
IR-R
MHCC97H
MHCC97L

H

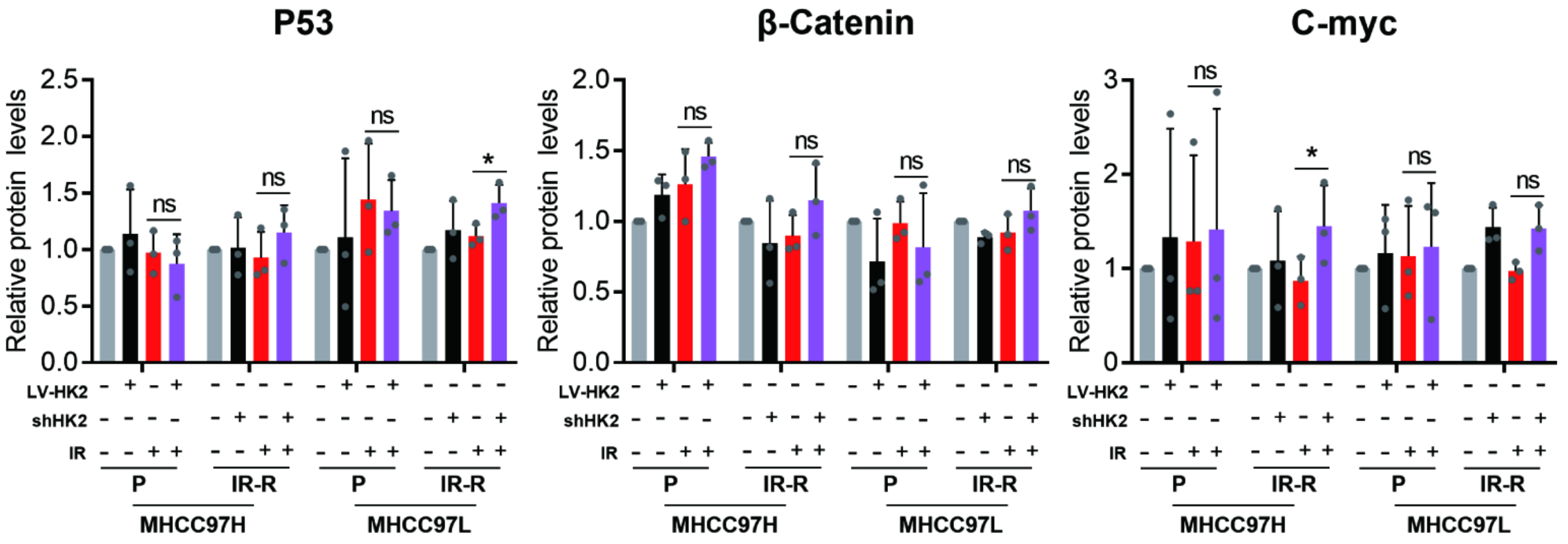
P53
β-Catenin
C-myc
Relative protein levels
ns
*
LV-HK2
shHK2
IR
P
IR-R
MHCC97H
MHCC97L

I

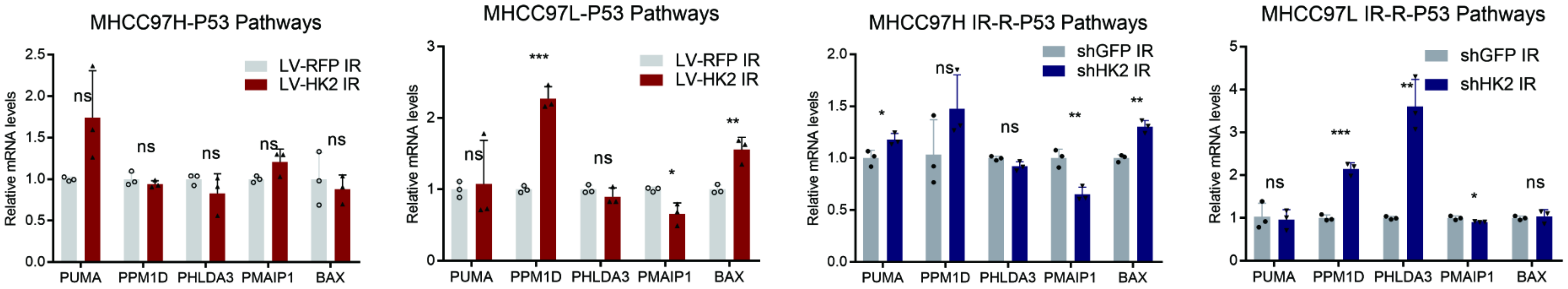

MHCC97H-P53 Pathways
LV-RFP IR
LV-HK2 IR
Relative mRNA levels
PUMA
PPM1D
PHLDA3
PMAIP1
BAX
MHCC97L-P53 Pathways
LV-RFP IR
LV-HK2 IR
MHCC97H IR-R-P53 Pathways
shGFP IR
shHK2 IR
MHCC97L IR-R-P53 Pathways
shGFP IR
shHK2 IR

J

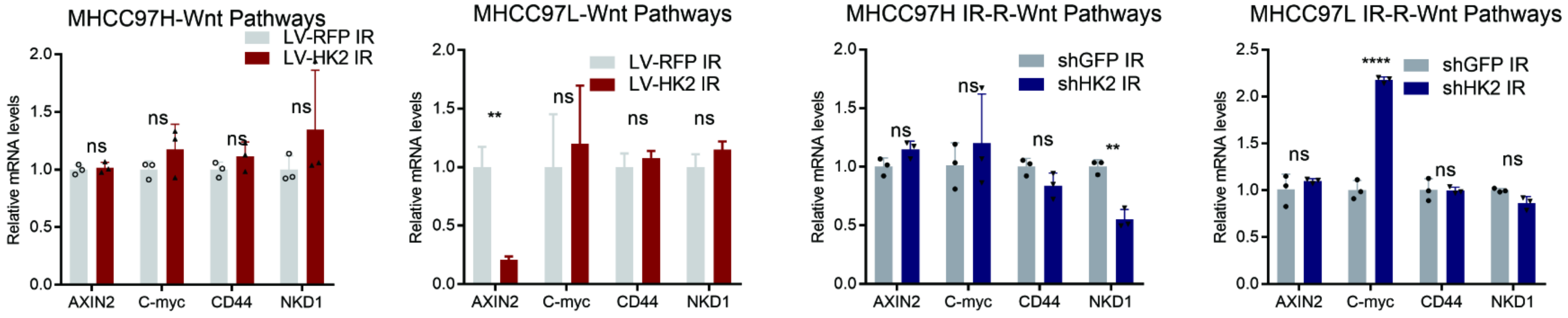

MHCC97H-Wnt Pathways
LV-RFP IR
LV-HK2 IR
Relative mRNA levels
2.0
1.5
1.0
0.5
0.0
ns
ns
ns
ns
AXIN2
C-myc
CD44
NKD1
MHCC97L-Wnt Pathways
LV-RFP IR
LV-HK2 IR
Relative mRNA levels
**
ns
ns
ns
AXIN2
C-myc
CD44
NKD1
MHCC97H IR-R-Wnt Pathways
shGFP IR
shHK2 IR
Relative mRNA levels
ns
ns
ns
**
AXIN2
C-myc
CD44
NKD1
MHCC97L IR-R-Wnt Pathways
shGFP IR
shHK2 IR
Relative mRNA levels
2.5
****
ns
ns
ns
AXIN2
C-myc
CD44
NKD1
