# Supplementary material for: Hexokinase 2 confers radio-resistance in hepatocellular carcinoma by promoting autophagy-dependent degradation of AIMP2
Source: Cell Death Dis. 2023 Aug 1;14(8):488. doi: 10.1038/s41419-023-06009-2 (PMC10390495; doi:10.1038/s41419-023-06009-2)
Supplement: Supplementary file 20 — Extended Supplementary Figure6 (Supplementary Figure6 merge file) [file 41419_2023_6009_MOESM20_ESM.pdf]

A

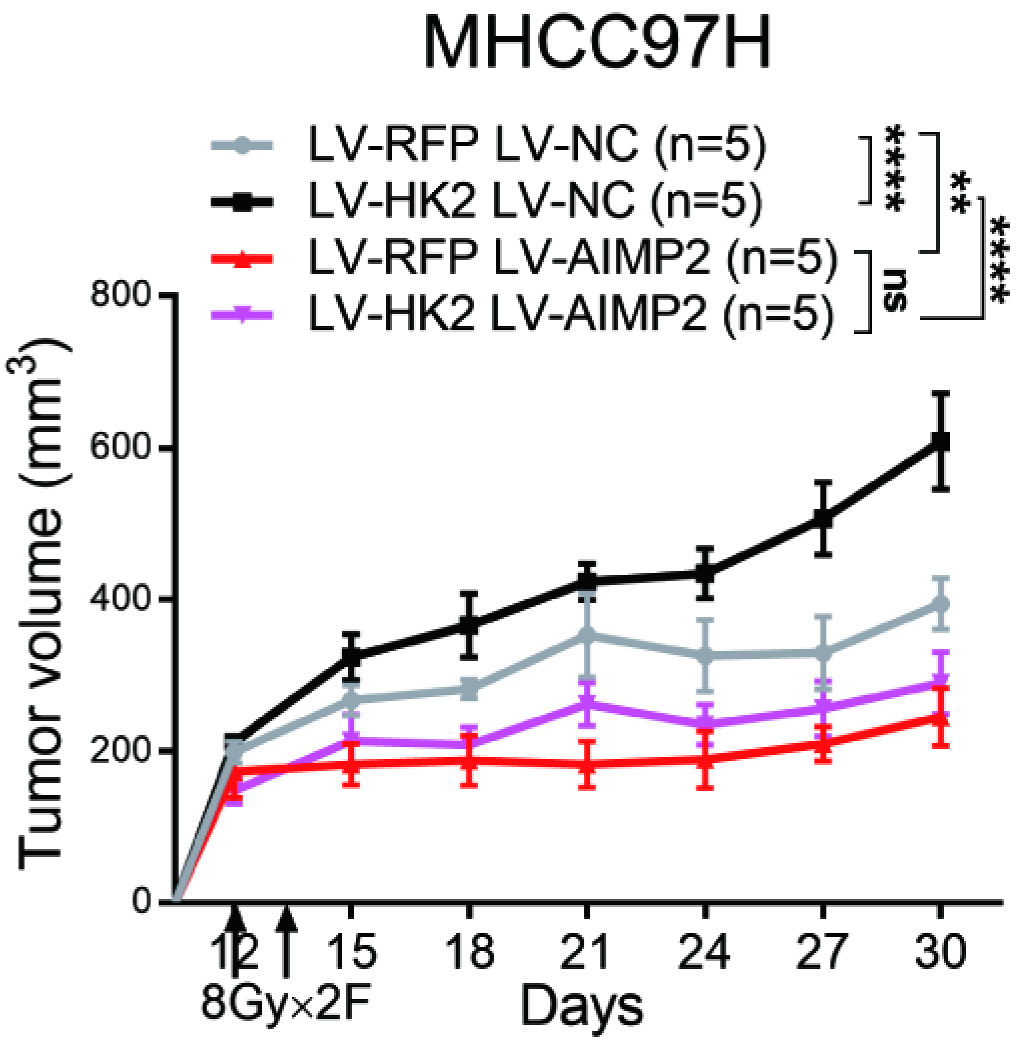

MHCC97H
LV-RFP LV-NC (n=5)
LV-HK2 LV-NC (n=5)
LV-RFP LV-AIMP2 (n=5)
LV-HK2 LV-AIMP2 (n=5)
****
**
ns
****
Tumor volume (mm3)
800
600
400
200
0
12
15
18
21
24
27
30
8Gy×2F
Days

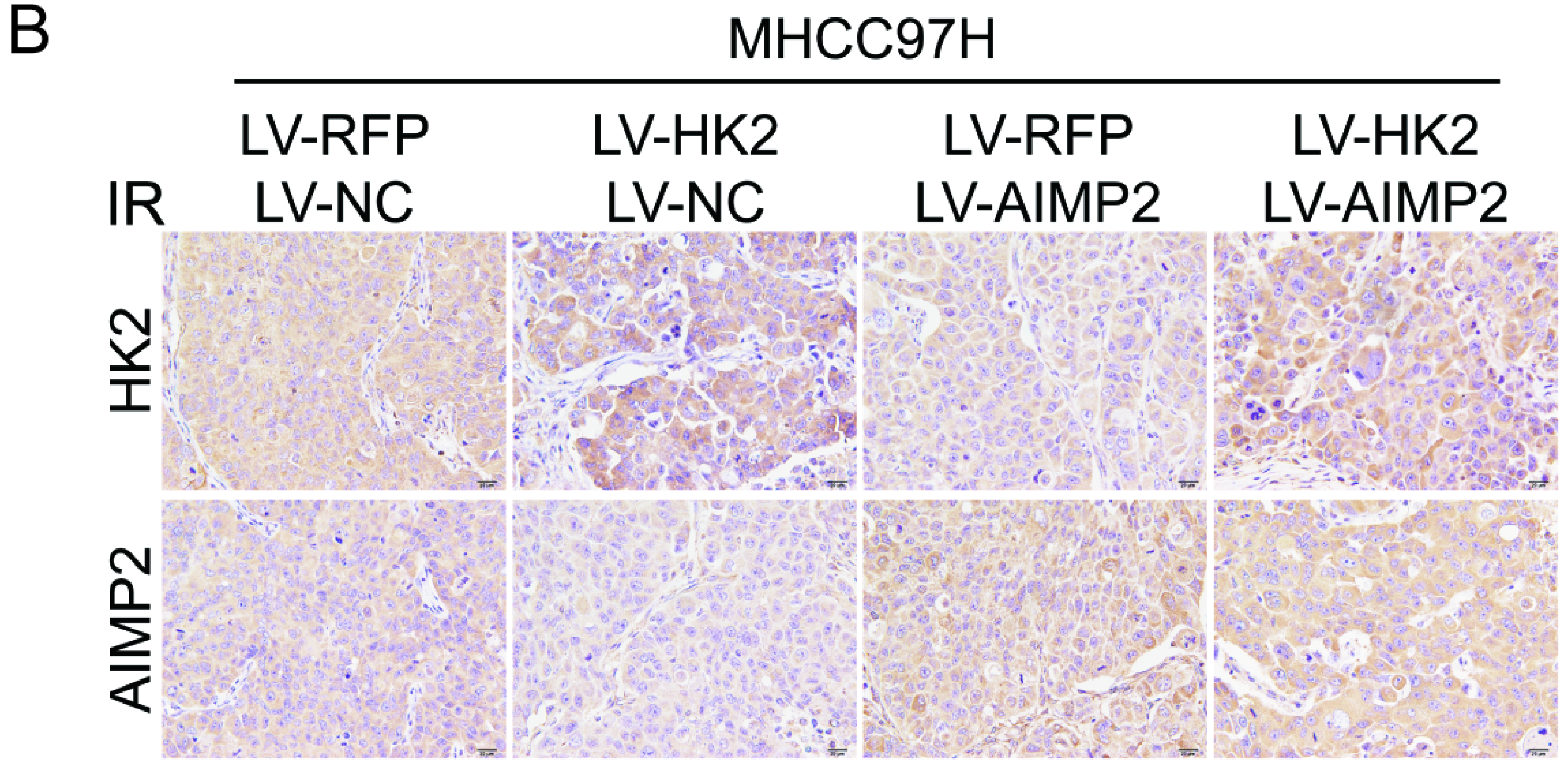

B
MHCC97H
IR
LV-RFP
LV-NC
LV-HK2
LV-NC
LV-RFP
LV-AIMP2
LV-HK2
LV-AIMP2
HK2
AIMP2

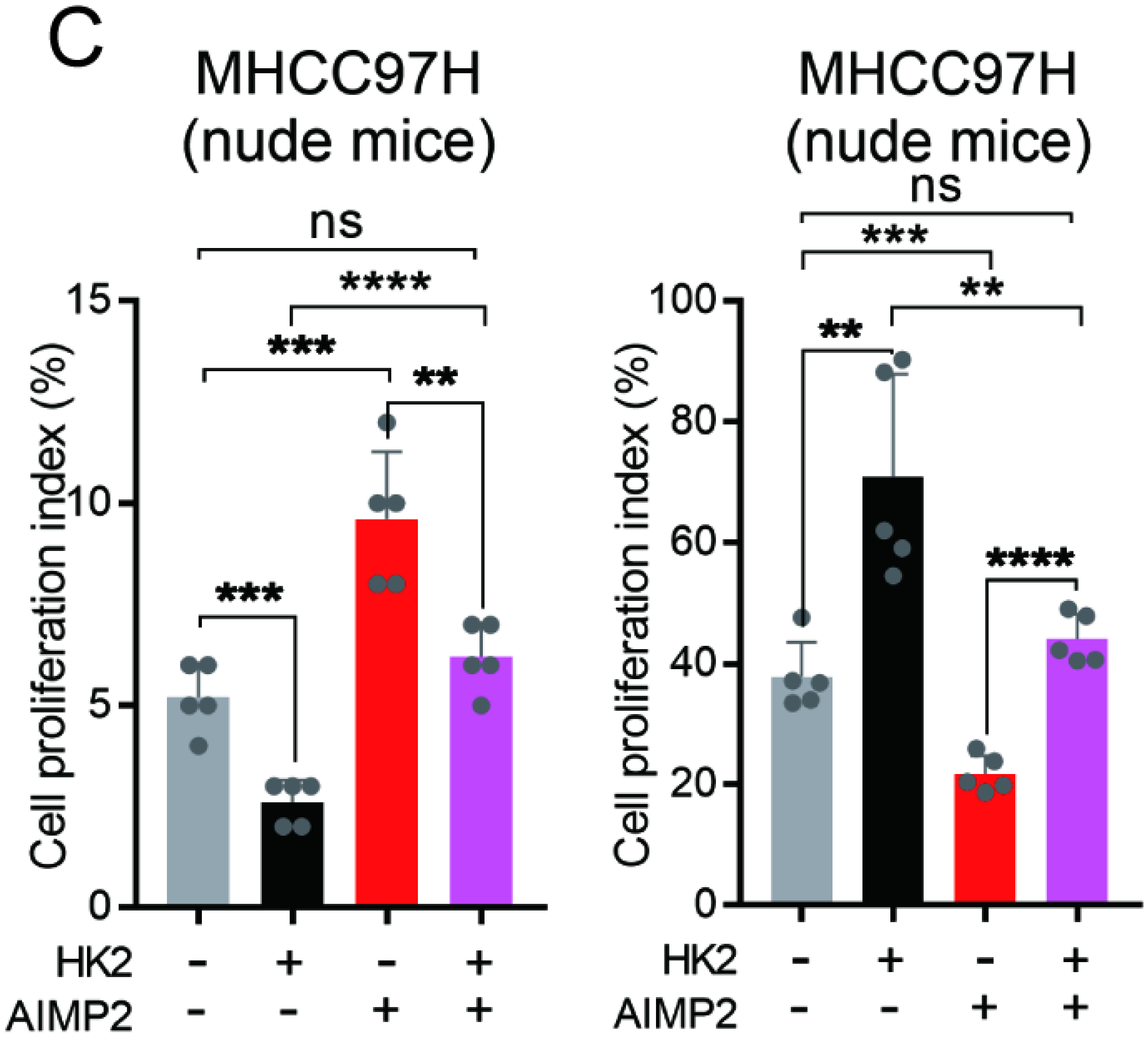
C
MHCC97H
(nude mice)
ns
****
***
**
***
Cell proliferation index (%)
0
5
10
15
HK2 - + - +
AIMP2 - - + +
MHCC97H
(nude mice)
ns
***
**
**
****
Cell proliferation index (%)
0
20
40
60
80
100
HK2 - + - +
AIMP2 - - + +

D

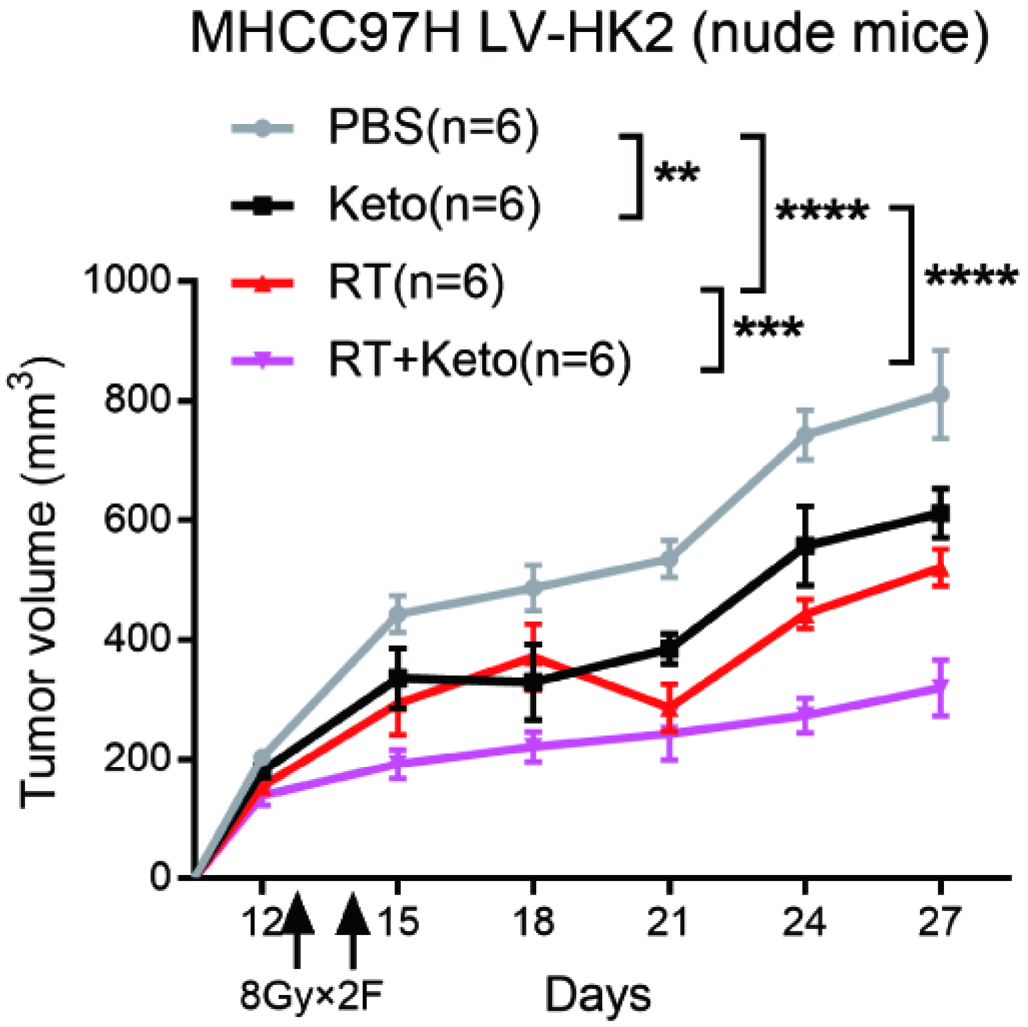
MHCC97H LV-HK2 (nude mice)
PBS(n=6)
Keto(n=6)
RT(n=6)
RT+Keto(n=6)
**
****
***
****
Tumor volume (mm3)
1000
800
600
400
200
0
12
15
18
21
24
27
8Gy×2F
Days

E

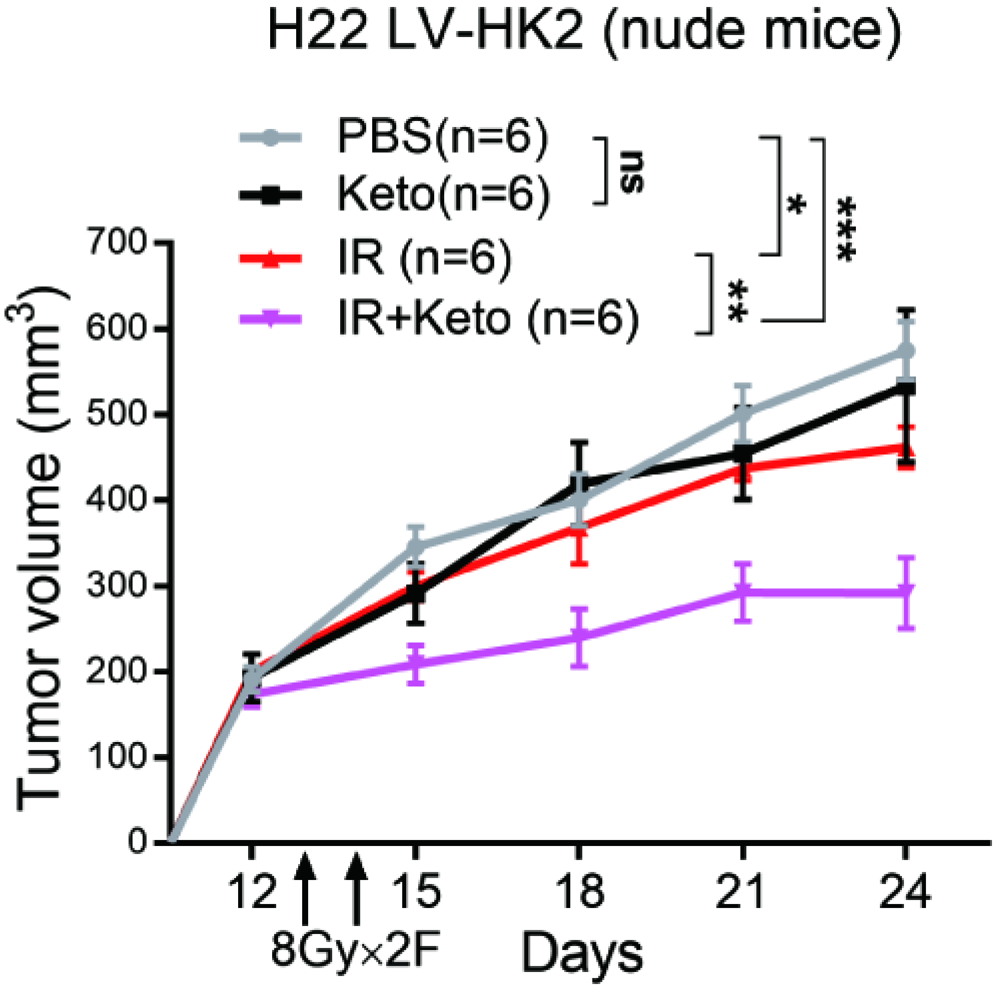

H22 LV-HK2 (nude mice)
PBS(n=6)
Keto(n=6)
IR (n=6)
IR+Keto (n=6)
ns
*
**
***
700
600
500
400
300
200
100
0
Tumor volume (mm3)
12
15
18
21
24
8Gy×2F
Days

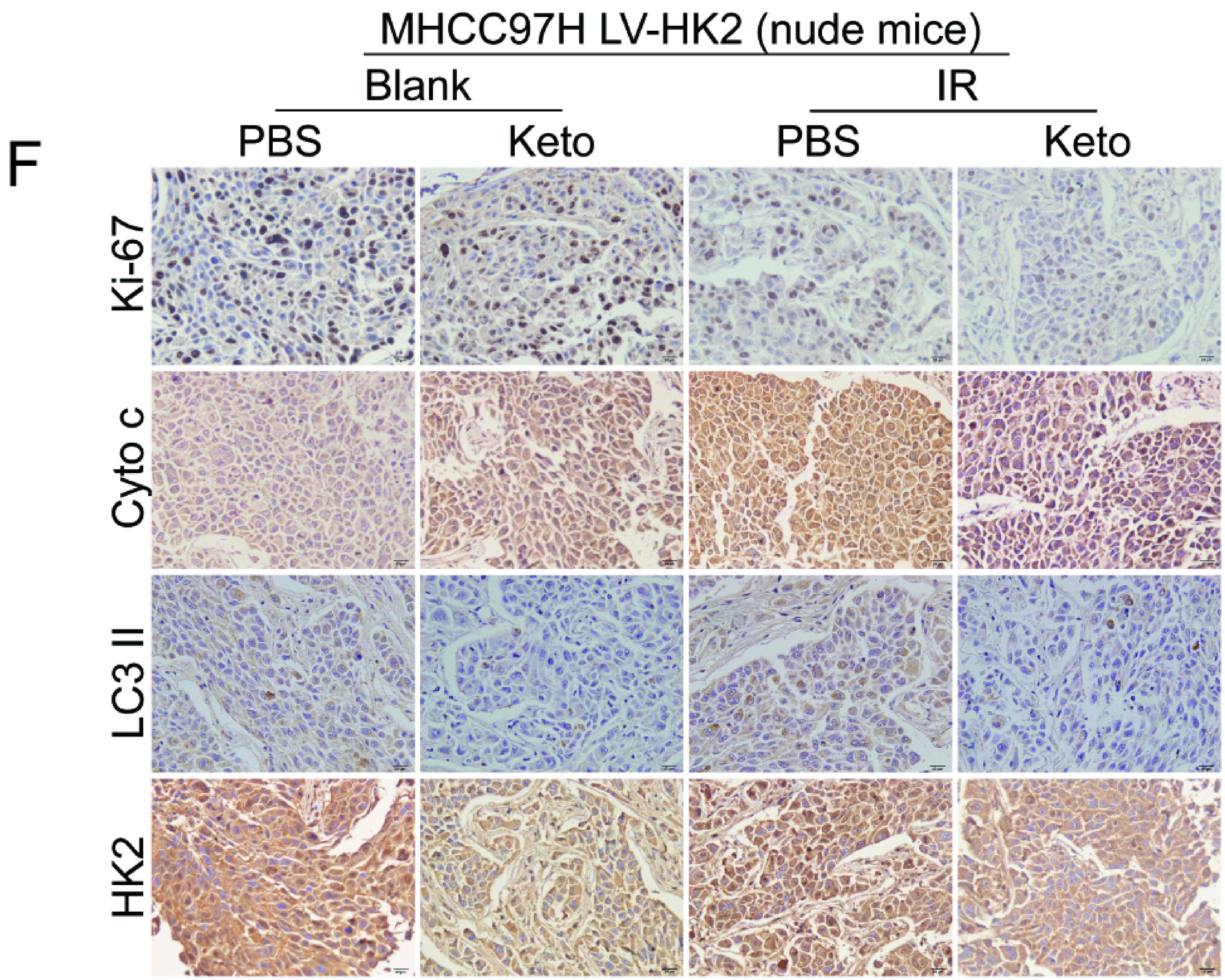
F
MHCC97H LV-HK2 (nude mice)
Blank
IR
PBS
Keto
PBS
Keto
Ki-67
Cyto c
LC3 II
HK2

G

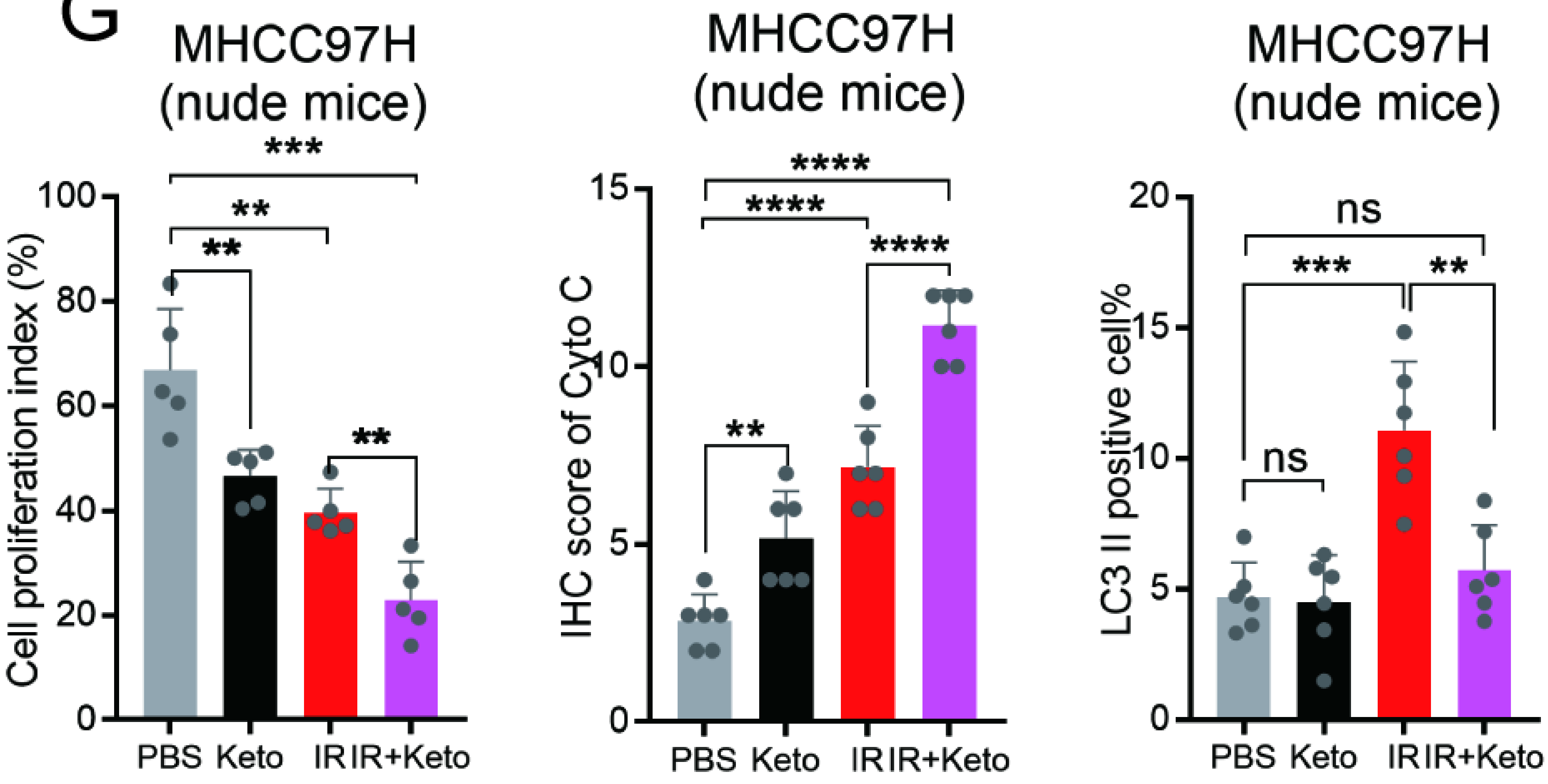
MHCC97H
(nude mice)
Cell proliferation index (%)
100
80
60
40
20
0
***
**
**
**
PBS
Keto
IR
IR+Keto
MHCC97H
(nude mice)
IHC score of Cyto C
15
10
5
0
****
****
****
**
PBS
Keto
IR
IR+Keto
MHCC97H
(nude mice)
LC3 II positive cell%
20
15
10
5
0
ns
***
**
ns
PBS
Keto
IR
IR+Keto

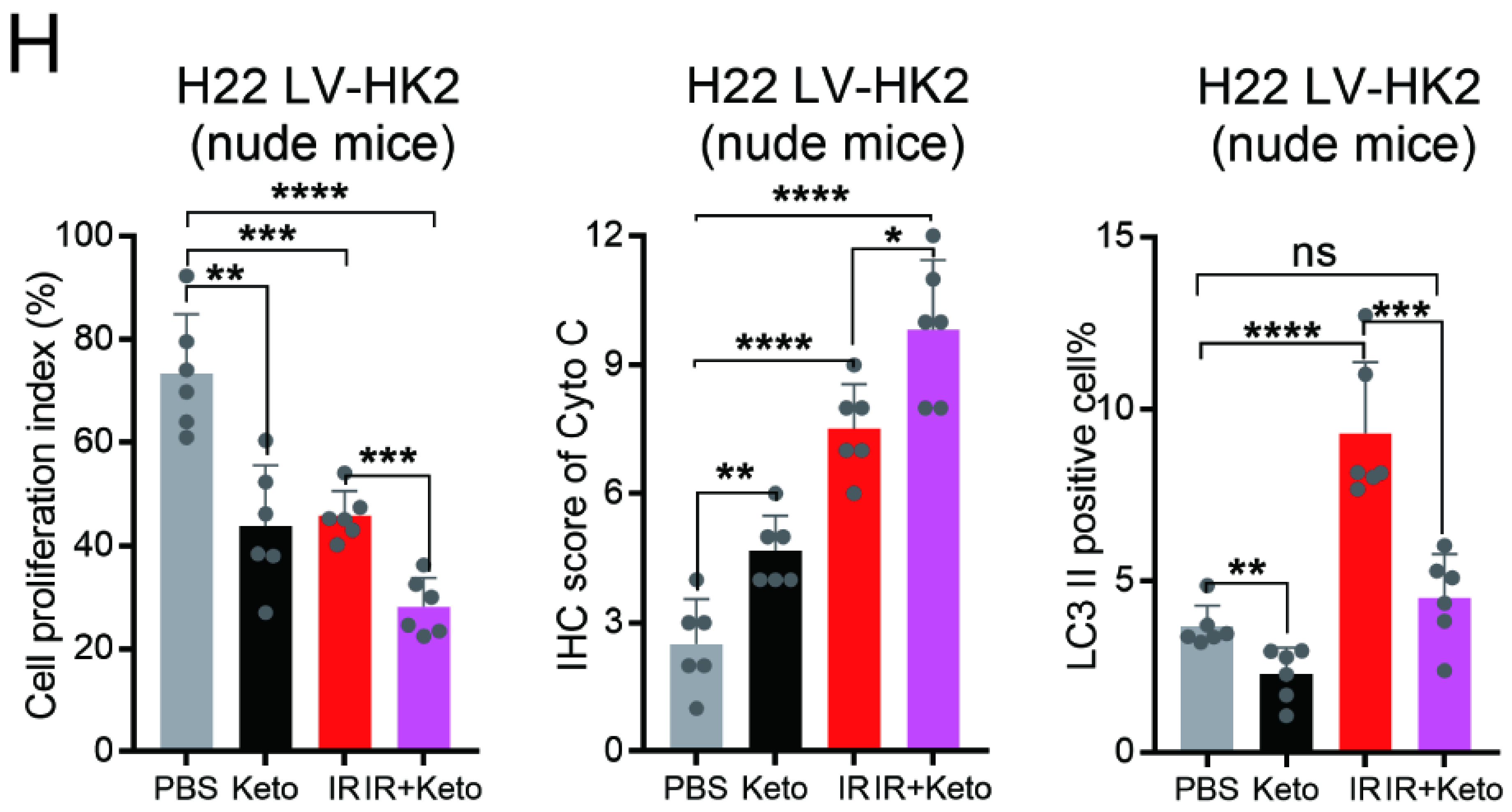

H
H22 LV-HK2
(nude mice)
Cell proliferation index (%)
100
80
60
40
20
0
PBS
Keto
IR
IR+Keto
****
***
**
***
H22 LV-HK2
(nude mice)
IHC score of Cyto C
12
9
6
3
0
PBS
Keto
IR
IR+Keto
****
*
****
**
H22 LV-HK2
(nude mice)
LC3 II positive cell%
15
10
5
0
PBS
Keto
IR
IR+Keto
ns
****
***
**

I

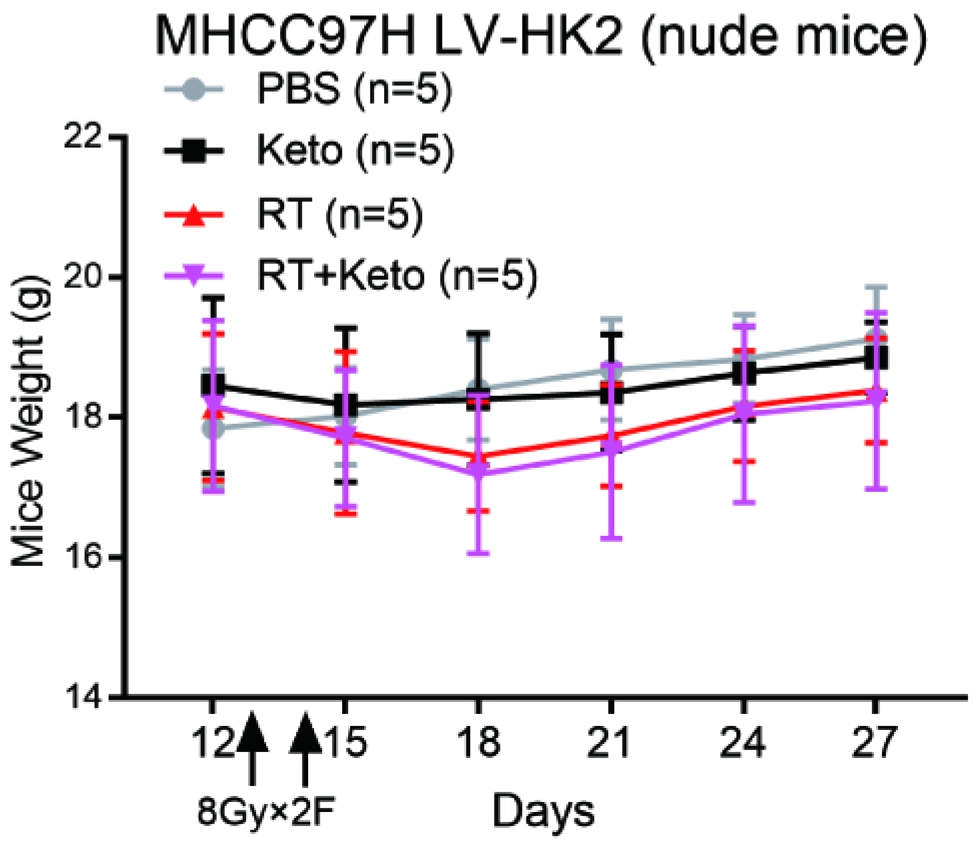

MHCC97H LV-HK2 (nude mice)
PBS (n=5)
Keto (n=5)
RT (n=5)
RT+Keto (n=5)
Mice Weight (g)
22
20
18
16
14
12
15
18
21
24
27
8Gy×2F
Days

J

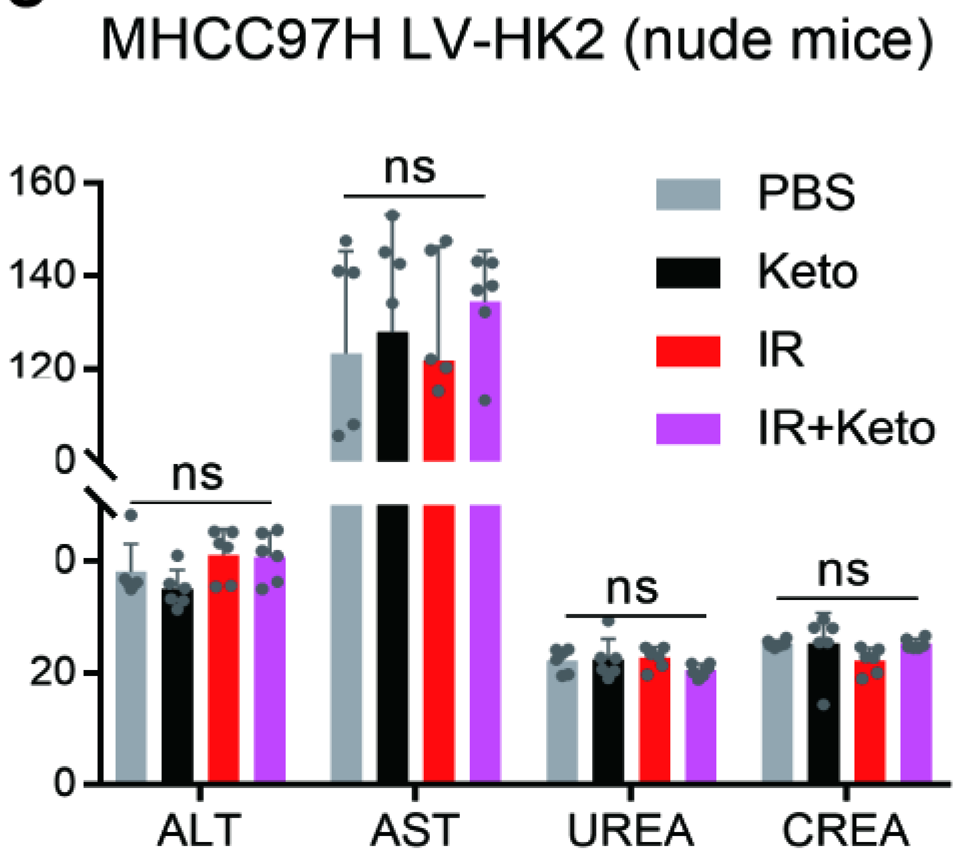
MHCC97H LV-HK2 (nude mice)
160
140
120
0
0
20
0
ns
ns
ns
ns
PBS
Keto
IR
IR+Keto
ALT
AST
UREA
CREA

K

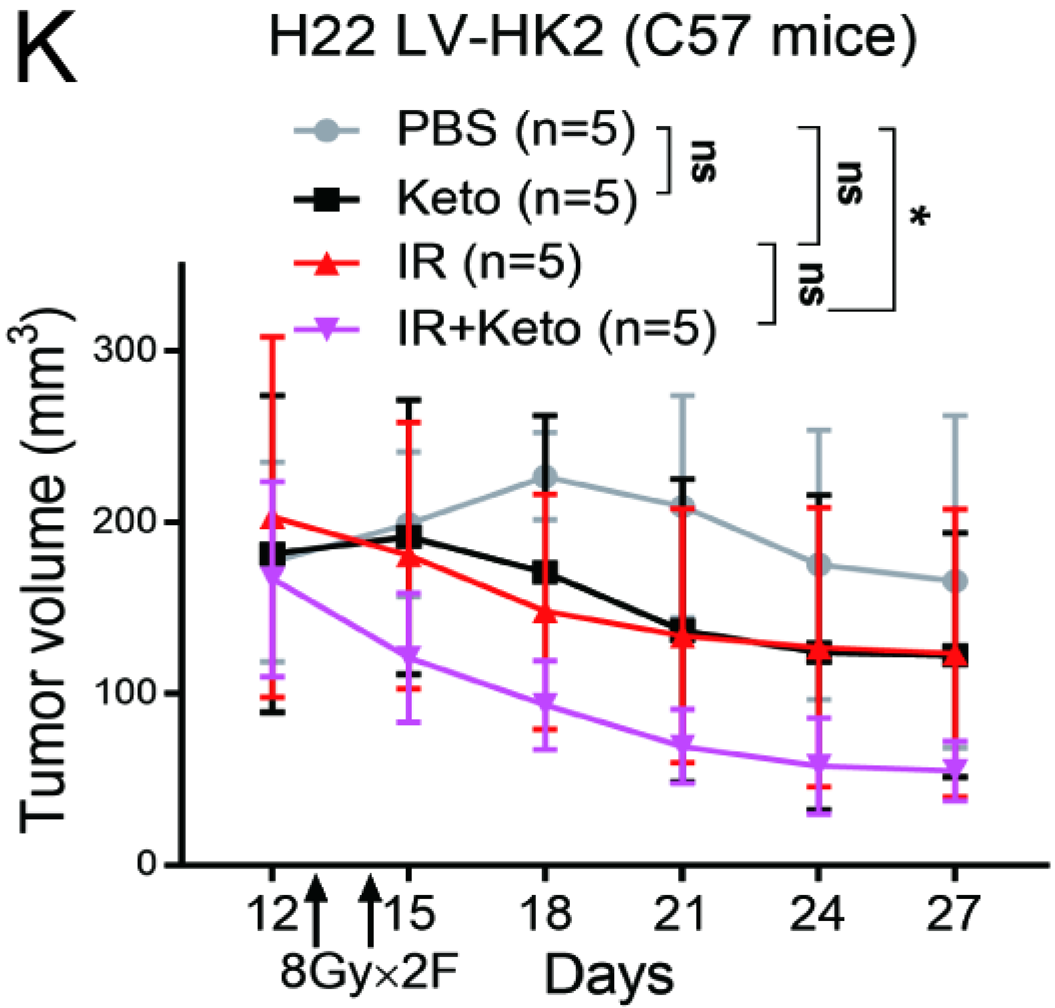

H22 LV-HK2 (C57 mice)
PBS (n=5)
Keto (n=5)
IR (n=5)
IR+Keto (n=5)
ns
ns
ns
*
Tumor volume (mm3)
300
200
100
0
12
15
18
21
24
27
8Gy×2F
Days

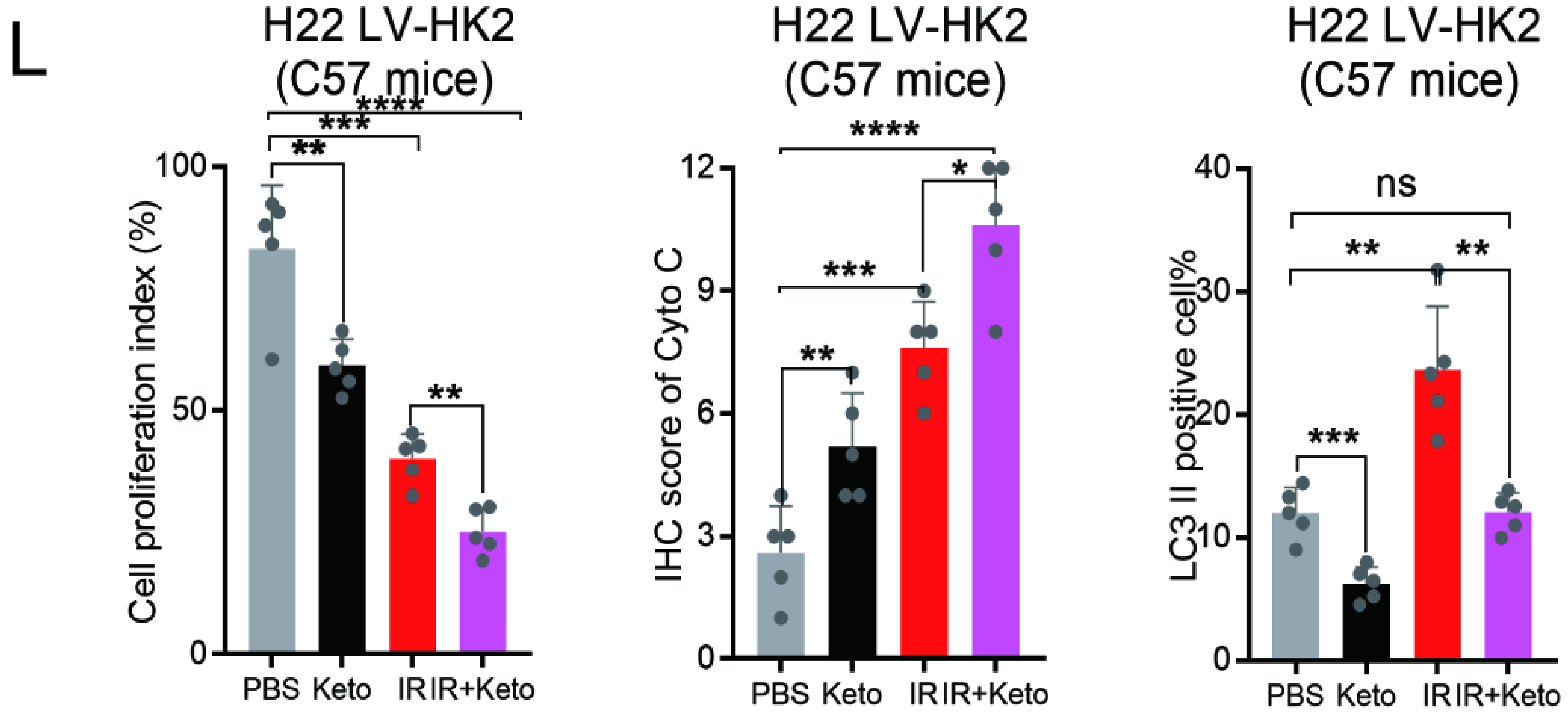
L
H22 LV-HK2 (C57 mice)
Cell proliferation index (%)
100
50
0
**
***
****
**
PBS
Keto
IR
IR+Keto
H22 LV-HK2 (C57 mice)
IHC score of Cyto C
12
9
6
3
0
****
*
***
**
PBS
Keto
IR
IR+Keto
H22 LV-HK2 (C57 mice)
LC3 II positive cell%
40
30
20
10
0
ns
**
**
***
PBS
Keto
IR
IR+Keto
